# Identification of novel potential hypoxia-inducible factor-1α inhibitors through machine learning and computational simulations

**DOI:** 10.3389/fchem.2025.1585882

**Published:** 2025-05-12

**Authors:** Yuxiang He, Shuning Diao, Shengzhen Hou, Taiying Li, Wenhui Meng, Jinping Zhang

**Affiliations:** ^1^ First Clinical Medical College, Shandong University of Traditional Chinese Medicine, Jinan, China; ^2^ Third Department of Infectious Diseases, The Fourth People’s Hospital of Zibo, Zibo, China; ^3^ Affiliated Hospital of Shandong University of Traditional Chinese Medicine, Jinan, China

**Keywords:** hypoxia-inducible factor-1α, virtual screening, machine learning, molecular docking, molecular dynamics simulation

## Abstract

**Introduction:**

Hypoxia-inducible factor-1α (HIF-1α) has become a significant therapeutic target for breast cancer and other cancers by regulating the expression of downstream genes such as erythropoietin, thereby improving cell survival in hypoxic conditions.

**Methods:**

We jointly applied a multistage screening system encompassing machine learning, molecular docking, and molecular dynamics simulations to conduct virtual screening of the “Traditional Chinese Medicine Monomer Library” for potential HIF-1α inhibitors. The virtual screening was conducted in three sequential stages, applying the following selection criteria sequentially: an activity prediction score greater than or equal to 0.8, a stronger binding affinity, and an MM-PBSA binding free energy lower than the reference compound.

**Results and Discussion:**

We retrieved 361 compounds with HIF-1α inhibitory activity data from the ChEMBL database for the construction and evaluation of machine learning models. Among the six constructed models, the random forest model based on RDKit molecular descriptor with the optimal comprehensive performance was employed for virtual screening. Ultimately, four compounds were selected for binding mode analyses and 100 ns molecular dynamics simulations. The results showed that the compounds Arnidiol and Epifriedelanol exhibit the most stable interactions with the HIF-1α protein, which can serve as potential HIF-1α inhibitors for future investigations.

## 1 Introduction

HIF-1 is a heterodimeric protein consisting of the HIF-1α subunit, which is regulated by oxygen levels, and the constitutively expressed HIF-1β subunit. The HIF-1β subunit plays a critical role in forming the HIF-1 heterodimer, whereas the HIF-1α subunit is primarily responsible for regulating the activity of HIF-1 (Graham and Presnell, 2017). Both subunits contain a basic helix-loop-helix (bHLH) motif and a Per-ARNT-Sim (PAS) structural domain, which are responsible for binding DNA as well as forming heterodimers. Unlike HIF-1β, HIF-1α also contains an oxygen-dependent degradation structural domain (ODDD) and two transactivation domains (TADs), namely, the N-terminal active domain (N-TAD) and the C-terminal active domain (C-TAD) (Ziello et al., 2007). Under normal oxygen levels, the factor inhibitor of hypoxia-inducible factor 1 (FIH-1) hydroxylates C-TAD, inhibiting its interaction with the transcriptional co-activators CPB and p300. Proline hydroxylase-2 (PHD-2) catalyzes the hydroxylation of ODDD, leading to the degradation of the HIF-1α protein after ubiquitination (Ke and Costa, 2006). In contrast, at lower oxygen levels, the activities of FIH-1 and PHD-2 are correspondingly decreased. The structurally stable HIF-1α protein translocates to the nucleus and forms a heterodimer with HIF-1β. The HIF-1 heterodimer interacts with transcriptional co-activators and binds to hypoxia-responsive elements (HREs) of downstream genes, enhancing their expression (Wenger et al., 2005). The regulation of HIF-1 enhances the expression of downstream genes such as erythropoietin (EPO) and vascular endothelial growth factor (VEGF). Cells can adapt to hypoxic conditions through increasing oxygen delivery, reducing oxygen consumption, or activating anaerobic metabolic pathways (Albadari et al., 2019).

Cancer has emerged as a significant public health issue worldwide. According to the “Global Cancer Statistics 2022” released by the International Agency for Research on Cancer (IARC) in 2024,there were approximately 20 million newly diagnosed cancer cases and 9.7 million cancer-related deaths worldwide in 2022 (Bray et al., 2024). Chemotherapy and radiotherapy constitute the principal treatment modalities for cancer. However, these therapies are associated with significant adverse effects, such as radiation-induced damage, gastrointestinal toxicity, and myelosuppression. Consequently, there is an imperative necessity to develop innovative pharmaceuticals for oncological therapy. Research has found that HIF-1α is overexpressed in various cancers, such as brain, breast, and oropharyngeal cancers, promoting the proliferation and metastasis of cancer cells (Semenza, 2003). For example, HIF-1α promotes the proliferation of endothelial cells and tumor angiogenesis by mediating the VEGFR-1/VEGF/VEGFR-2 autocrine signaling pathway, which enhances the survival of cancer cells under hypoxic conditions (Liao and Johnson, 2007). Consequently, the development of HIF-1α targeted inhibitors is of great significance for the treatment of cancer.

Computer-aided drug design (CADD) is a multidisciplinary technology that utilizes methods such as docking and virtual screening, Quantitative Structure-Activity Relationship (QSAR), and pharmacophore modeling to simulate and predict the interactions between drug molecules and their biological targets (Niazi and Mariam, 2023). Compared to conventional drug discovery methods, CADD employs accurate computations to predict the interactions between targets and pharmaceuticals, improving the selectivity of pharmaceuticals while reducing research and development expenses and time. With the help of CADD technology, researchers have successfully developed notable pharmaceuticals such as dorzolamide, saquinavir, and imatinib. It can be said that CADD has become an important driving force in the field of drug development.

Machine learning constitutes an essential branch of artificial intelligence. In machine learning, computers analyze training data to learn its patterns and regularities. Based on these patterns and regularities, algorithm models are constructed and employed to perform predictions or decisions regarding unknown data. In the field of drug discovery, in contrast to traditional CADD approaches such as molecular docking and pharmacophore models, machine learning algorithms (MLAs) possess distinctive advantages in aspects like the screening of key features and can score or classify hit compounds in large databases more effectively (Crampon et al., 2022; Parvatikar et al., 2023). Therefore, in recent years, the joint application of ML algorithms and CADD methods has achieved many satisfactory results in drug discovery (Patel et al., 2020). For instance, Narendra et al. employed multiple machine learning models for virtual screening to identify selective human ALDH1A1 inhibitors. They constructed and jointly applied SVM and RF models for virtual screening of three databases. Subsequently, they performed molecular docking, ADMET analysis, and molecular dynamics simulation on the screened compounds, and 10 selective ALDH1A1 inhibitors were finally identified (Narendra et al., 2021). Jingyu Zhu et al. constructed a naive Bayes classification model and combined methods such as molecular docking and bioactivity evaluation to conduct virtual screening of the ChemDiv database. Eventually, they identified an efficacious inhibitor of PI3Kγ, namely, JN-K13 (Zhu et al., 2022).

Currently, virtual screening studies of potential inhibitors targeting HIF-1α are predominantly based on molecular docking or pharmacophore modeling methods. For example, Yadav PK et al. used a 3D-QSAR-based pharmacophore model method, screening ZINC02121040 as a promising candidate for HIF-1α inhibitors (Yadav et al., 2023). Latha MS et al. used the molecular docking method, identifiying ten potential molecule inhibitors, such as ZINC04280532, that can inhibit the activity of HIF-1 heterodimer (Latha and Saddala, 2017). As previously stated, these methods have certain limitations in the screening process of critical features and may not adequately identify HIF-1α inhibitors. Therefore, it is necessary to jointly apply machine learning and CADD methods to conduct a more comprehensive discovery and exploration of HIF-1α inhibitors.

**FIGURE 1:**
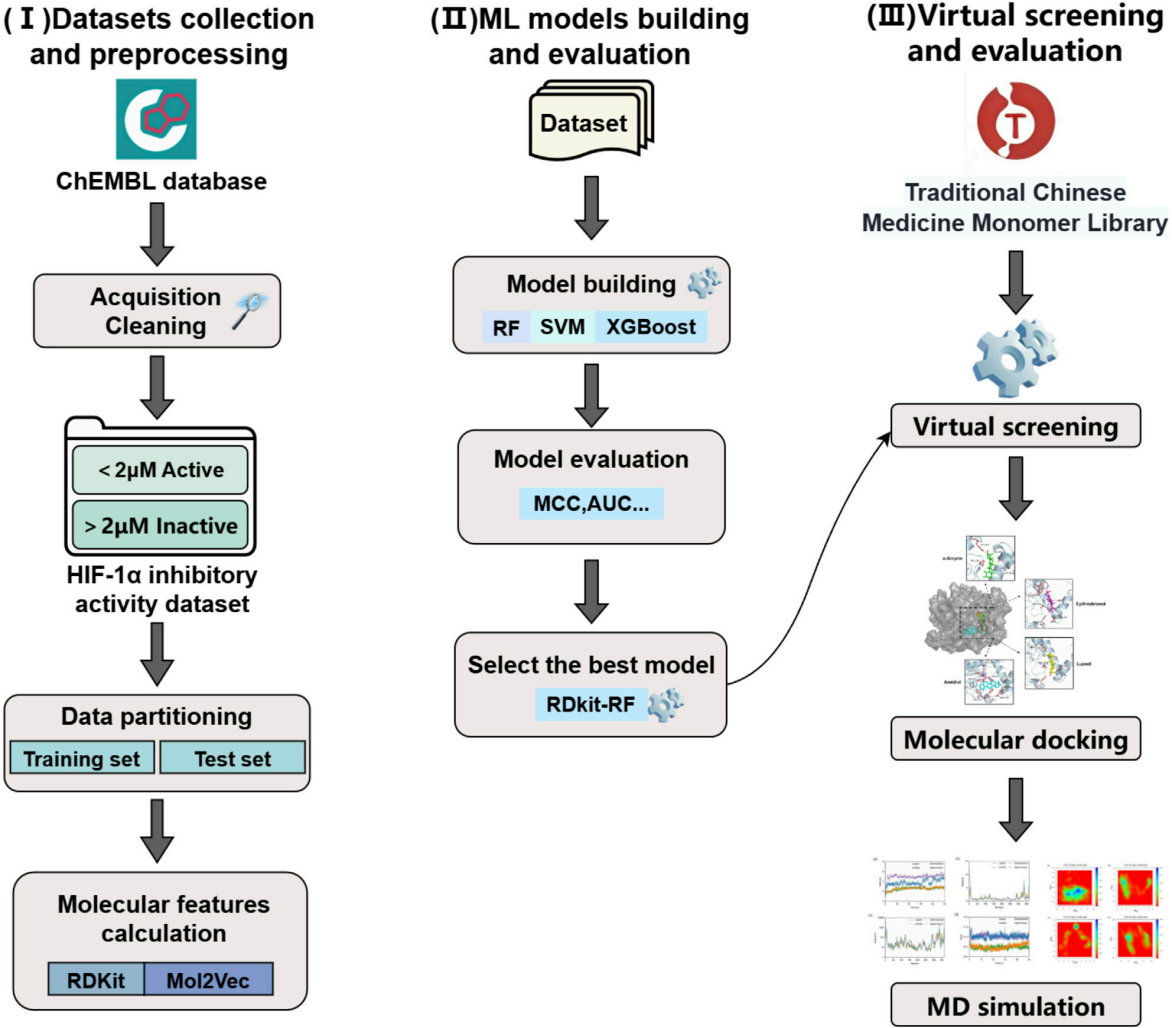
Workflow for the virtual screening of HIF-1α inhibitors based on machine learning methods.

In our study, we constructed a joint virtual screening workflow based on machine learning, molecular docking and molecular dynamics simulation. Firstly, we computed two types of molecular features, namely, RDKit and Mol2Vec, for the compounds in the training set. Subsequently, we established six machine learning models utilizing three algorithms, namely, RF, SVM, and XGBoost. After the evaluation of model performance, the optimal model was utilized to screen the “Traditional Chinese Medicine Monomer Library” of TargetMol. The active compounds obtained from the screening were subjected to molecular docking with HIF-1α, and compounds with stronger binding energies were further selected for molecular dynamics simulations. The MM-PBSA binding free energy was calculated through molecular dynamics simulations to assess the interaction mechanism and stability during the dynamic process, so as to thoroughly explore potential HIF-1α inhibitors (Figure 1).

## 2 Material and methods

### 2.1 Data preprocessing

ChEMBL is a large-scale bioactive molecule database containing 2.43 million compounds (Zdrazil et al., 2024). A total of 576 compounds with recorded inhibitory activity data against HIF-1α were retrieved from the ChEMBL database (ChEMBL ID-CHEMBL4261). The activity data of compounds in ChEMBL were mainly obtained from the scientific literature, the PubChem bioassay and the BindingDB database, etc. Then we selected compounds with binding type “B”, removed compounds with uncertain IC_50_ values or multiple IC_50_. After cleaning the data, we ultimately obtained 361 compounds. With 2 μM as the threshold for activity classification, 129 compounds with an IC_50_ value ≤ 2 μM were classified as “active”, and 232 compounds with an IC_50_ value > 2 μM were classified as “inactive”. Then the data were normalized using the StandaedScaler method. By removing the mean and scaling to unit variance, the original data were transformed into data conforming to the standard normal distribution. Finally, the dataset was partitioned into training and test sets at a ratio of 4:1.

### 2.2 Molecular features calculation

Molecular descriptors are mathematical or logical representations of molecular properties based on numeric values or standardized experimental results (Grisoni et al., 2018). RDKit molecular descriptors are among the most commonly utilized molecular descriptors, capable of representing molecular attributes from multiple perspectives such as physicochemical properties, topological properties, and pharmaceutical properties. Mol2Vec is an unsupervised machine learning technique based on natural language processing (NLP). It compares the substructure of a molecule based on Mrogan’s algorithm to a “word” and the whole molecule to a “sentence” (Jaeger et al., 2018). We employed the Morgan algorithm to calculate the feature vectors of molecular substructures. Subsequently, the feature vectors of all the substructures are aggregated to form the molecular composite vector, which is the Mol2Vec descriptor of the molecule. In this study, we utilized the Scikit-learn library in Python to generate 208 RDKit 2D molecular descriptors for each data point. Meanwhile, we established a 300-dimensional molecular structure vector descriptor for each data point via the Mol2Vec method. The one with the optimal performance was finally selected for the establishment of the machine learning model.

### 2.3 Machine learning models building

In this study, we used the Scikit-learn library of Python to build three machine learning models based on the RDKit and Mol2Vec molecular descriptors, including random forests (RF), support vector machines (SVM), and extreme gradient boosting trees (XGBoost). Moreover, we performed hyperparameter tuning by Bayesian optimization methods.

Random Forest (RF) comprises an ensemble of numerous decision trees (Breiman, 2001). For each decision tree, the RF algorithm randomly selects a portion of the data from the original dataset with replacement as the training set. A certain number of features are randomly chosen from all features, and an optimal feature is further selected based on strategies such as information gain as the splitting basis for a node. The node proceeds to split until the maximum depth is reached or no further splitting is feasible. Each tree will make a prediction for classification problems. The consensus outcome after voting by all decision trees is the prediction of the random forest. Hence, the hyperparameters employed by RF include “max_depth”, “min_samples_leaf”, and “n_estimators.”

Support Vector Machine (SVM) is a commonly utilized supervised machine learning algorithm primarily employed for classification issues. The SVM algorithm constructs a hyperplane to distinguish two classes of data points with the maximum possible margin. A hard margin can be used for fully linearly separable data, while a soft margin can be adopted for approximately linearly separable data (Evgeniou and Pontil, 2001). For non-linear data, kernel functions are employed to transform it into linear data in a high-dimensional space. Hence, SVM possesses two hyperparameters, namely, “C” and “gamma.”

Extreme Gradient Boosting Tree (XGBoost) is a scalable tree-boosting machine learning system based on the gradient boosting algorithm (Chen and Guestrin, 2016). XGBoost aims to improve the overall predictive performance of the model by building numerous weak learners. The essence of XGBoost is the iterative training of decision trees. In each iteration, a new tree is trained based on the residuals of the previous round of modeling. The residuals are gradually diminished through iterations to optimize the prediction model. Consequently, the hyperparameters utilized by XGBoost encompass “colsample_bytree”, “gamma”, “learning_rate”, “max_depth”, “min_child_weight”, “n_estimators”, and “subsample.”

### 2.4 Evaluation of machine learning models

Model performance evaluation is a necessary step in machine learning, which can accurately quantify the predictive and generalization abilities of models, thus guiding us to select effective and reliable models in practical applications. We use machine learning models constructed from the training set to perform pre-screening tests on the test set. The performance of each model is evaluated through a ten-fold cross-validation method utilizing metrics including accuracy, precision, recall, F1 score, Matthews Correlation Coefficient (MCC), and Area Under the Curve (AUC). The optimal model was chosen for virtual screening of HIF-1α inhibitors. The formulae for each metric were as follows:

$$\text{Accuracy}=\frac{TN+TP}{TN+TP+FN+FP}$$

$$\text{Precision}=\frac{TP}{TP+FP}$$

$$\text{Recall}=\frac{TP}{TP+FN}$$

$$F1\text{ score}=\frac{2\times Percision\times Recall}{Precision+Recall}$$

$$\text{MCC}=\frac{TP\times TN-FP\times FN}{\sqrt{\left(TP+FP\right)\times \left(TP+FN\right)\times \left(TN+FP\right)\times \left(TN+FN\right)}}$$

TP (True Positives) and TN (True Negatives) respectively represent the quantities of correctly classified HIF-1α inhibitors and non-inhibitors. FP (False Positives) indicates the number of HIF-1α non-inhibitors that are wrongly predicted as inhibitors. FN (False Negatives) indicates the number of HIF-1α inhibitors that are wrongly predicted as non-inhibitors. MCC is a performance evaluation metric that integrates the data of TP, TN, FP, and FN, and can reliably reflect the predictive performance of binary classification models (Chicco and Jurman, 2020). AUC is another evaluation metric reflecting the effectiveness of machine learning models, with a value range from 0 to 1. The closer it is to 1, the stronger the classification ability of the model is. AUC > 0.9 can be regarded as the model having excellent predictive performance (Jiang et al., 2020).

### 2.5 SHAP analysis

SHAP is an interpretable artificial intelligence method based on the Shapley value of cooperative game theory (Salih et al., 2025). In the SHAP method, all possible combinations of features are termed as coalitions. It calculates the Shapley values for each feature in the model by computing the average marginal contributions of a feature in all possible coalitions, representing the influence weight of that feature on the model’s predictive outcomes. Each row in the figure represents a feature, and all features are arranged from top to bottom according to their feature importance, which is quantified by the average absolute value of Shapley. Each point in a row represents a sample under that feature, with higher feature values corresponding to redder sample points and lower values to bluer ones.

### 2.6 Molecular docking

The 3D cocrystal structure of HIF-1α (PDB ID: 1H2K) was retrieved from the Protein Data Bank (https://www.rcsb.org/). Water molecules, metal ions, and ligands in the cocrystal structure were removed utilizing the molecular visualization software PyMOL. All hydrogen atoms were added to the protein receptor and all small molecule ligands utilizing the AutoDock Tools software. Information such as rotatable bonds was verified. Finally, the protein receptor and small molecule ligands were saved in the PDBQT format, followed by molecular docking conducted with AutoDock Vina software.

AutoDock Vina is a high-performance computational program for molecular docking and virtual screening that utilizes new scoring functions, efficient optimization algorithms, and multi-threading technology (Trott and Olson, 2010). We employed the AutoDock Vina software for semi-flexible docking, allowing for conformational changes in the ligand while maintaining the receptor’s rigidity. The docking region was centered at coordinates X = 39.356, Y = 45.945, Z = 36.489, with a grid box of 40 Å × 40 Å × 40 Å established. The grid spacing and exhaustiveness value were respectively set to 0.375 and 10. We selected compounds with lower docking scores for further investigation and utilized PyMOL to visualize the docking results, thereby analyzing the interactions between the ligand and HIF-1α.

### 2.7 Molecular dynamics simulations

To gain a deeper understanding of the dynamic behavior and interaction mechanisms of molecular systems, we performed molecular dynamics simulations on the protein-ligand complex system using the Amber software and ff19SB force field (Salomon-Ferrer et al., 2013). Firstly, we generated the topology and coordinate files of proteins and ligands by the tleap program. The TIP3P water model with a buffering distance of 10 A was used to solvate the system. Sodium ions or chloride ions were added to balance the net charge of the system. After completing the assembly of the system, we performed energy minimization emoloying the steepest descent method and the conjugate gradient method to optimize the stability of the system. The constant-volume system was slowly heated to 300 K under weak confinement. The Berendsen barostat was used to control the pressure at 1atm, and the Langevin thermostat was used to control the temperature at 300 K. The system was equilibrated under isobaric-isothermal (NPT) ensemble for 1 ns, followed by a 100 ns production phase of molecular dynamics simulation. The Particle Mesh Ewald (PME) algorithm was employed to calculate long-range electrostatic interactions. The cutoff distance for non-bonded interactions was set to 10 Å. The SHAKE algorithm was utilized to constrain the high-frequency vibrations of hydrogen atoms. After the simulation, we analyzed the simulation trajectory for root-mean-square deviation (RMSD), root-mean-square fluctuation (RMSF), radius of gyration (Rg) and B-factors (temperature factors), drew the dynamic cross-correlation matrices and free energy landscapes by the ptraj program.

The RMSD reflects the degree of deviation of the system’s overall conformation from its initial conformation at a specific time. Higher RMSD values in the complex system indicate greater deviations. When the RMSD values stabilize, the system conformation ceases to change, indicating that the simulation system has reached equilibrium. The RMSF characterizes the fluctuation amplitude of individual atoms in the complex system relative to their average positions. Residues with higher RMSF values exhibit greater flexibility, while those with lower RMSF values demonstrate stronger structural rigidity. The B-factor, also known as the temperature factor, reflects the extent to which atoms in a complex system deviate from their positions due to vibration or thermal disturbance. Lower B-factor values of amino acid residues are more conducive to protein stability. The Rg serves as a critical parameter for evaluating structural compactness. A smaller Rg value indicates a more confined distribution of atoms around the center of mass, corresponding to greater spatial compactness of the structure. Dynamical cross-correlation matrix and free energy landscape further characterize the correlations between distinct residues and the relative free energy differences among various conformational states, respectively.

## 3 Result

### 3.1 Chemical information diversity analysis

**FIGURE 2:**
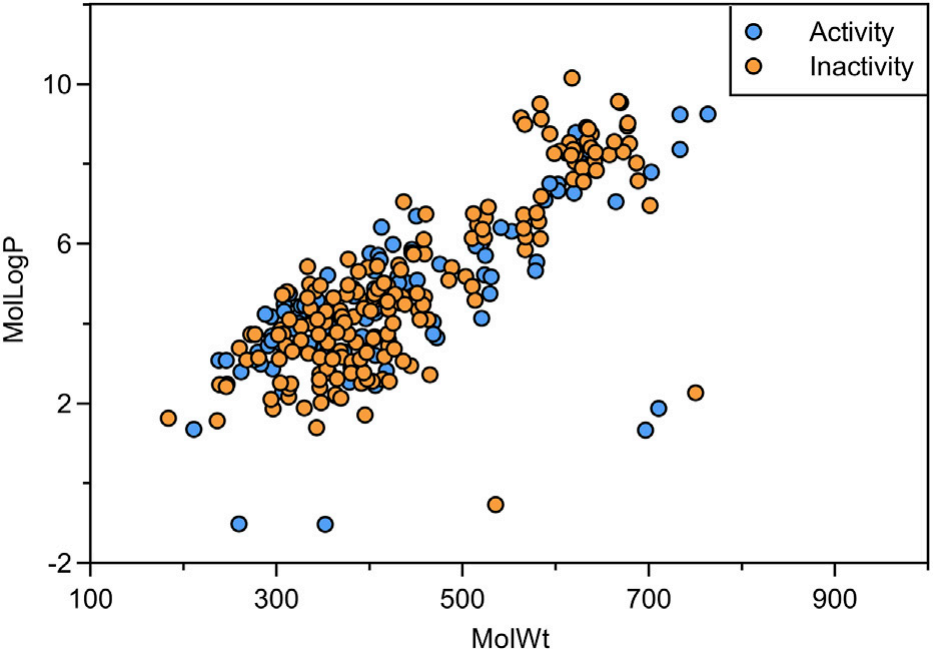
Scatter distribution plot of molecular weight and LogP for active and inactive data.

Sample diversity is an important factor affecting the prediction accuracy of machine learning models. Chemically diverse datasets can reduce sample bias, enhance the generalization capability of models, prevent overfitting, and thereby improve the predictive accuracy on novel data. In our study, we analyzed the molecular weight and logP of the compounds in the dataset. The molecular weight distribution of the compounds ranges from 184.198 to 763.909, and the distribution of logP ranges from −1.026 to 10.155 (Figure 2). It demonstrates that the compounds in the dataset have an excellent chemical space distribution and are suitable for the establishment of machine learning models.

### 3.2 Model performance

**FIGURE 3:**
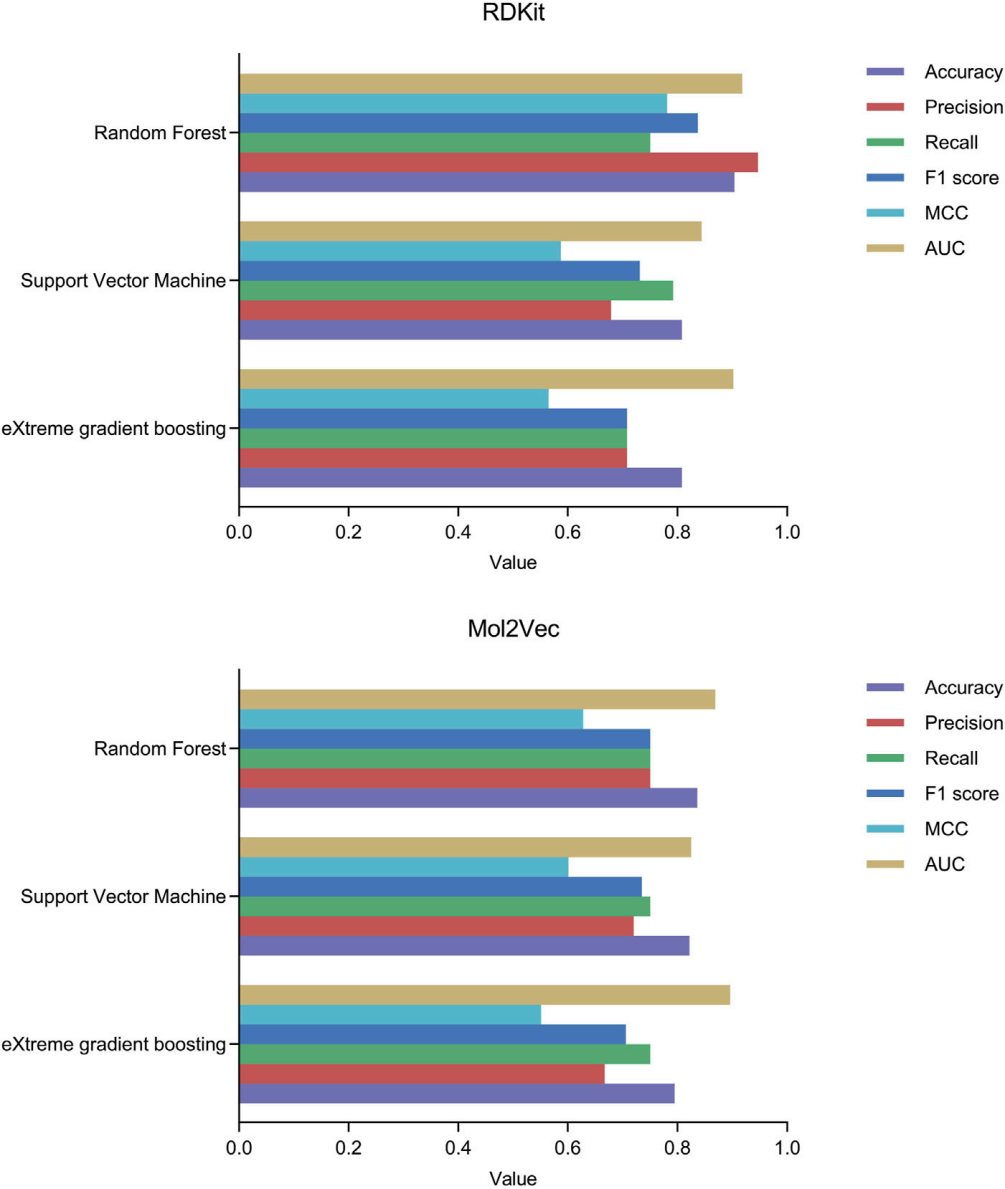
The performance of machine learning classification models under ten-fold cross-validation.

We constructed three machine learning classification models, namely, RF, SVM, and XGBoost, based on molecular descriptors and Mol2Vec features. We employed the Bayesian optimization algorithm to perform hyperparameter tuning and identified the best hyperparameter combinations for the six models, as detailed in Supplementary Material. Subsequently, we conducted a ten-fold cross-validation on the test set to assess the performance of the machine learning models based on metrics including accuracy, precision, recall, F1 score, MCC, and AUC. The performance metrics data of all models are presented in Table 1. Among them, MCC and AUC are prioritized as performance evaluation metrics for binary classification models (Chicco and Jurman, 2020; Ling et al., 2003). The results show that the RF model based on RDKit molecular descriptors has an MCC value of 0.781, which significantly surpasses the other five models, demonstrating the superior performance of the RDKit-RF model in binary classification. Additionally, the model exhibits an AUC value of 0.918, which is the highest among all models, suggesting that it possesses excellent prediction performance for HIF-1α inhibitors. Despite the recall value of the RDKit-RF model being 0.75, the disparity with the RDKit-SVM model with the highest recall value is relatively minimal, and it is comparable to the other four models. Moreover, the RDKit-RF model also possesses significantly superior accuracy, precision values, and F1 score (Figure 3). Considering all factors, we concluded that the RDKit-RF model exhibited the best overall performance and was most suitable for virtual screening of HIF-1α inhibitors.

**TABLE 1:** Performance evaluation of each machine learning model.

Model	Accuracy	Precision	Recall	F1 score	MCC	AUC
RDkit-RF	0.904	0.947	0.750	0.837	0.781	0.918
RDkit -SVM	0.808	0.679	0.792	0.731	0.587	0.844
RDkit -XGBoost	0.808	0.708	0.708	0.708	0.565	0.902
Mol2Vec-RF	0.836	0.750	0.750	0.750	0.628	0.869
Mol2Vec -SVM	0.822	0.720	0.750	0.735	0.601	0.825
Mol2Vec -XGBoost	0.795	0.667	0.750	0.706	0.551	0.896

### 3.3 SHAP analysis

**FIGURE 4:**
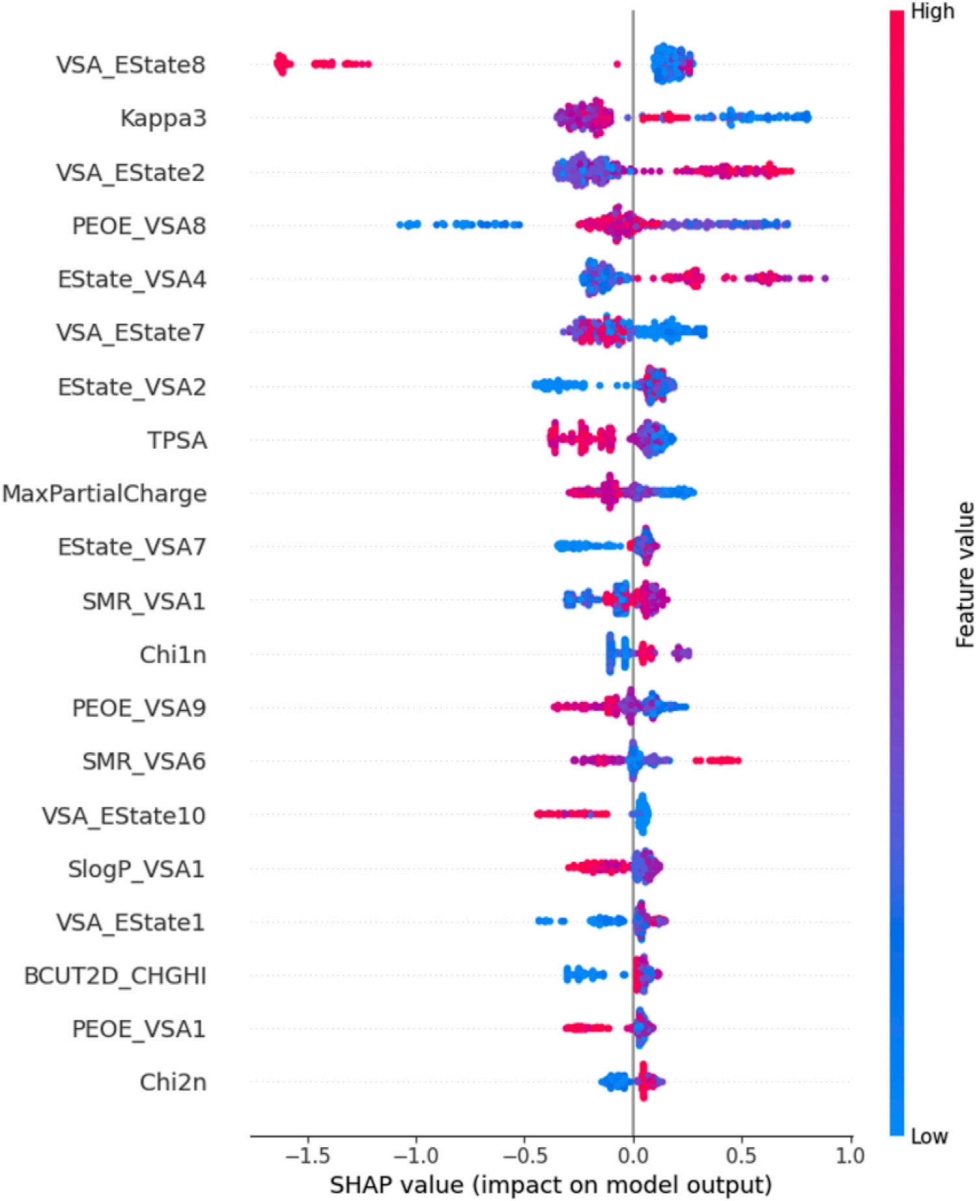
Scatter diagram of variables in SHAP analysis.

We performed SHAP analysis on all the features utilized in the machine learning model (Figure 4). Through SHAP analysis, we can determine the degree of influence each feature exerts on the prediction outcomes. For instance, the VSA_Estate8 descriptor belongs to the EState-VSA (Electrostatic State-Valence State Analysis) hybrid descriptor, which denotes atoms or atomic groups with an electronic state value ranging from 6.45 to 7.00. This descriptor is the feature that has the greatest impact on the model’s predictive outcomes. The Chi2n descriptor characterizes the electronic properties and structural information of a molecule, representing the number of paths of length 2 within the molecule. Therefore, this descriptor is the least important feature for the prediction outcomes. Further analysis revealed that the red sample points with high values of the VSA_Estate8 feature have negative SHAP values, indicating a negative influence of VSA_Estate8 on the activity prediction outcomes. In contrast, Chi2n exerts a slightly positive influence on the activity prediction outcomes.

### 3.4 Virtual screening

#### 3.4.1 Prediction score for inhibitory activity

**FIGURE 5:**
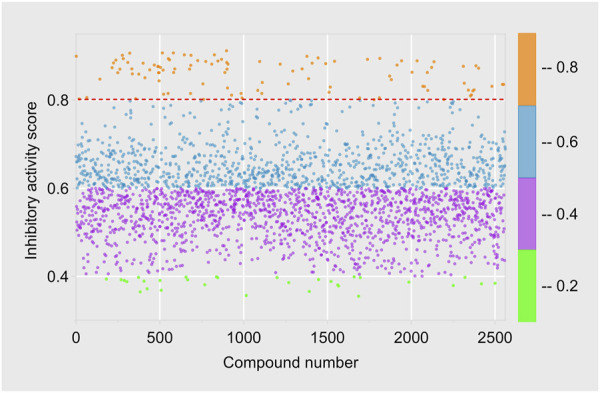
The distribution range of prediction scores of RDKit-RF model.

The Traditional Chinese Medicine Monomer Library developed by Topscience company encompasses 2,910 monomer compounds derived from traditional Chinese medicine sources, including various structural types like flavonoids and alkaloids. After excluding some compounds that failed to meet the criteria, we utilized the optimal machine learning model RDkit-RF to predict the probability of inhibitory activity of 2,560 compounds in the Traditional Chinese Medicine Monomer Library, as detailed in Supplementary Material. Using a cutoff threshold of “Active” = 0.8, 2,456 compounds were categorized as “Inactivity”, with “Active” values ranging from 0.355 to 0.799; 104 compounds were classified as “Activity”, with “Active” values ranging from 0.800 to 0.917 (Figure 5).

#### 3.4.2 Binding affinity

We conducted molecular docking analyses on 104 compounds with “active” values greater than 0.8 against HIF-1α (PDB ID: 1H2K), and computed their affinity, as detailed in Supplementary Material. The results show that the affinity of the 104 compounds range from −9.035 to −5.838 kcal/mol, indicating that all compounds form significant interactions with the target protein. To improve the precision of virtual screening and thoroughly explore potential HIF-1α inhibitors, we further selected 8 compounds with affinity ranging from −9.035 to −8.505 kcal/mol (Table 2) for 10 ns molecular dynamics simulations to calculate their MM-PBSA binding free energy.

**TABLE 2:** The eight compounds with the lowest binding affinities.

Compound	PubChem CID	Structure	Binding affinity
β-Amyrin	73145	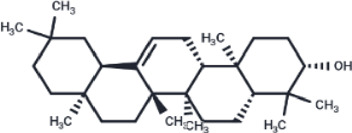	−9.035
Arnidiol	10478550	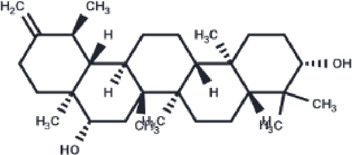	−8.886
alpha-Amyrin	73170	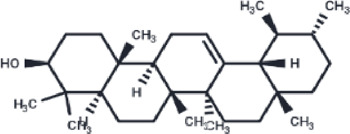	−8.802
Soyasapogenol B	115012	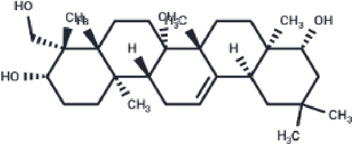	−8.734
Neoruscogenin	9910474	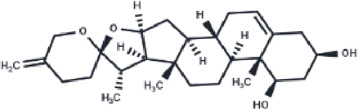	−8.640
Lupenone	92158	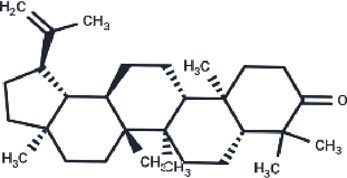	−8.626
Epifriedelanol	119242	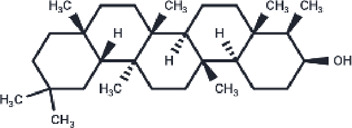	−8.626
Lupeol	259846	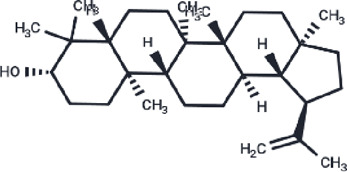	−8.505

#### 3.4.3 MM-PBSA binding free energy

In the 10 ns molecular dynamics simulation, we employed the MM-PBSA method to calculate the binding free energy of these eight compounds. The results are presented in Table 3. The total binding free energy of the eight compounds ranges from −16.5178 to −27.2463 kcal/mol. Among them, the compound Epifriedelanol exhibits the lowest binding free energy of −27.2463 ± 2.2615 kcal/mol, indicating the strongest binding affinity to the HIF-1α target. Further analysis indicated that van der Waals forces, electrostatic energy, and non-polar solvation energy contribute favorably to the binding of the compound with the target protein. Van der Waals forces are the primary contributors to the total binding free energy. In contrast, polar solvation energy is not conducive to their binding. The established potent HIF-1α inhibitor, 7-Hydroxyneolamellarin A (PubChem CID:24179494), served as the reference compound, with a binding free energy of −19.5983 ± 3.4421 kcal/mol. Compared to the reference compound, the compounds epifriedelanol, arnidiol, alpha-amyrin, and lupeol exhibited lower binding free energy. Therefore, we proposed that these four compounds are the most probable HIF-1α inhibitors and are suitable candidates for further docking analysis and molecular dynamics simulation studies.

**TABLE 3:** The MM-PBSA binding free energies of the selected compounds and the reference compound.

Compound	van der Waals	Electrostatic	Polar solvation	Non-polar solvation	Delta total
β-Amyrin	−24.8296 ± 3.0824	−1.9171 ± 2.5689	12.3711 ± 2.9900	−3.0817 ± 0.2327	−17.4573 ± 2.9240
Arnidiol	−28.9238 ± 3.1297	−26.7377 ± 6.7507	36.0393 ± 3.1439	−3.4469 ± 0.1674	−23.0690 ± 6.0784
alpha-Amyrin	−32.9273 ± 2.8387	−2.8381 ± 3.2600	17.3814 ± 3.4603	−4.0435 ± 0.1614	−22.4275 ± 2.9015
Soyasapogenol B	−30.1631 ± 3.7572	−13.3866 ± 6.9211	29.2832 ± 6.1601	−3.3842 ± 0.2792	−17.6507 ± 3.2998
Neoruscogenin	−34.8140 ± 3.0997	−26.2047 ± 5.4528	45.4834 ± 5.2613	−4.0078 ± 0.1471	−19.5431 ± 3.6187
Lupenone	−24.5069 ± 3.6751	−3.2034 ± 2.2221	14.3452 ± 3.0921	−3.1527 ± 0.4063	−16.5178 ± 2.3276
Epifriedelanol	−34.6658 ± 2.3512	−0.5926 ± 1.4122	11.9477 ± 3.0692	−3.9356 ± 0.2320	−27.2463 ± 2.2615
Lupeol	−32.1686 ± 3.1916	−2.5593 ± 1.3048	16.8127 ± 4.3160	−3.7719 ± 0.2131	−21.6870 ± 3.8124
7-Hydroxylamellarin A	−29.1419 ± 3.6769	−16.5952 ± 7.7053	29.7008 ± 6.1931	−3.5619 ± 0.2274	−19.5983 ± 3.4421

**FIGURE 6:**
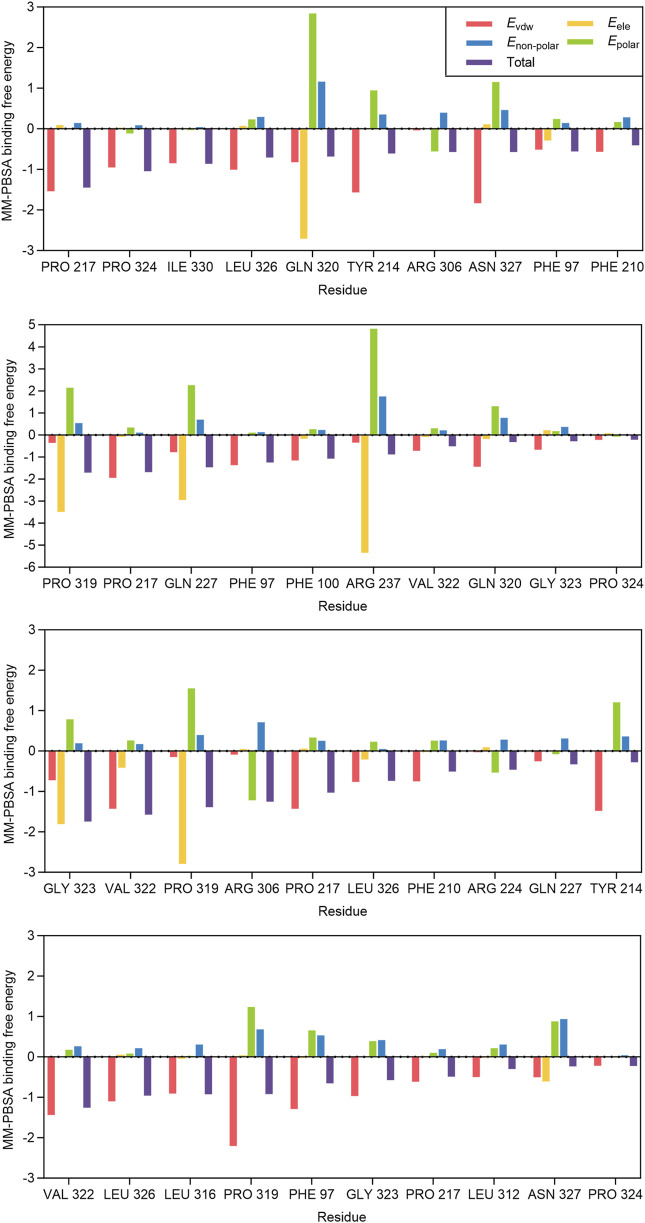
Residue contributions to the binding free energy of alpha-Amyrin, Arnidiol, Epifriedelanol, and Lupeol.

We further calculated the contribution of each residue to the MM-PBSA binding free energy of epifriedelanol, arnidiol, alpha-amyrin, and lupeol. Figure 6 displays, from top to bottom, the ten residues with the largest contributions to the binding free energy for Alpha-Amyrin, Arnidiol, Epifriedelanol, and Lupeol. We found that PRO 217 was the only residue that made a significant contribution to the binding free energy of all four compounds. Additionally, residues such as VAL 322, LEU 326, PRO 319, PHE 97, and GLY 323 demonstrated significant contributions to the binding free energy of three of the compounds. These residues represent noteworthy hotspots. Remarkably, there were a considerable number of high-contributing residues in the 310–340 region in all four compounds. Subsequent molecular dynamics simulations indicated that this region was highly flexible and related to the function of the active site. These residues were consistent with dynamic functional characteristics of the active region. Furthermore, a certain number of high-contribution residues were identified outside this region. Although molecular docking did not identify direct interactions between these two types of residues and the ligands, the calculation results of the contribution of residues to the binding free energy suggested that such residues might stabilize the overall conformation of the protein through long-range van der Waals forces or reduce the exposure of hydrophobic interfaces to decrease the loss of solvation entropy, thereby enhancing the ligand affinity in coordination with the directly binding residues. These findings demonstrated the necessity of combining multiple computational methods in elucidating the complex protein-ligand interaction mechanism and provided a new perspective for precise drug design.

### 3.5 Binding mode analysis

**FIGURE 7:**
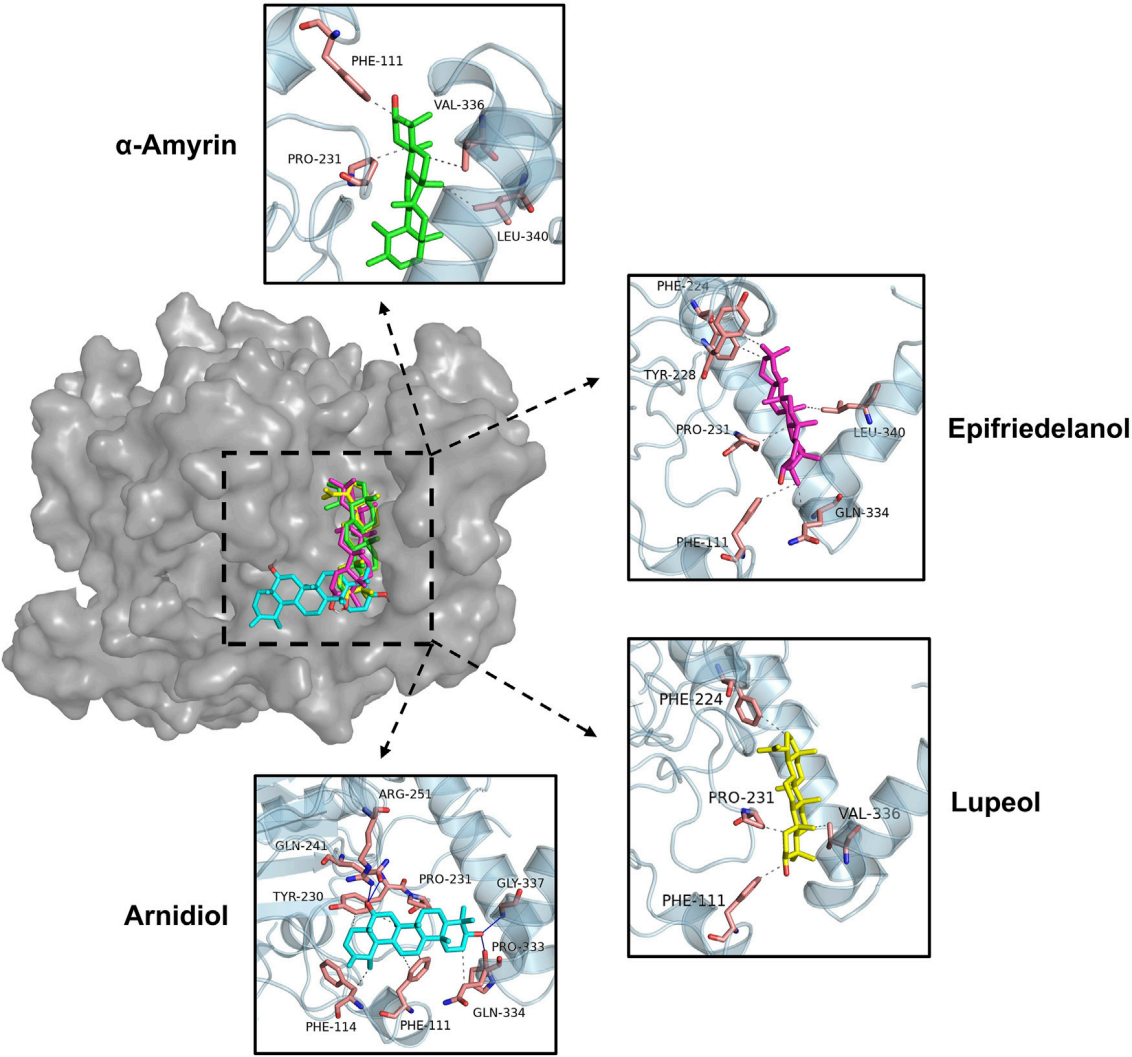
3D binding modes of four potential compounds with HIF-1α.

Figure 7 illustrated the intermolecular interactions between the four aforementioned potential HIF-1α inhibitors and the target protein. Table 4 further presents the bond lengths and bond angles of the interactions. Except for Arnidiol, the other three compounds docked into the binding pocket with similar orientations. Arnidiol forms seven hydrophobic interactions with residues PHE111, PHE114, TYR230, PRO231, and GLN334 through its saturated hydrocarbon chain, with bond lengths of 3.6, 3.51, 3.69, 3.87, 3.73, 3.44, and 3.62 Å. Additionally, it forms four hydrogen bond interactions with GLN241, ARG251, PRO333, and GLY337 through the hydroxyl group, with bond lengths of 3.0, 2.92, 3.09, and 2.72 Å and bond angles of 98.9, 141.9, 106.5, and 138.4. Research by Jeffrey et al. indicates that bond length and bond angle are critical factors determining hydrogen bond strength. Strong hydrogen bonds exhibit donor-acceptor distances of 2.2–2.5 Å, moderate-strength bonds range from 2.5–3.2 Å, while weak hydrogen bonds span 3.2–4.0 Å. Hydrogen bond strength increases when the bond angle approaches 180°, as this linear arrangement maximizes orbital overlap and electrostatic interactions between the donor and acceptor. Effective hydrogen bonds typically demonstrate angles exceeding 120° (Jeffrey, 1997). Specifically, the guanidyl group of ARG-251 exhibits strong donor characteristics and forms highly complementary electrostatic interactions with the hydroxyl group of Arnidiol, leading to a hydrogen bond with a short bond length (2.92 Å) and near-linear geometry (141.9°). GLY-337, lacking a side chain, exhibits high backbone conformational flexibility. This allows the formation of a hydrogen bond with the shortest bond length (2.72 Å) and a relatively ideal bond angle (138.4°). These two bonds exhibit characteristics of strong hydrogen bonds, significantly enhancing compound-target binding affinity. Although the hydrogen bonds formed between Arnidiol and PRO-333 and GLN-241 exhibit bond lengths within the moderate-strength range, their bond angles deviate from linearity to varying degrees. Consequently, these interactions are weaker and primarily contribute to the stabilization of the protein-ligand complex through synergistic and auxiliary effects.

**TABLE 4:** Analysis of the interaction between four compounds and HIF-1α.

Compound	Interaction type	Residue	Bond length	Bond angle
Arnidiol	Hydrophobic interaction	PHE-111	3.60	145.8
		PHE-111	3.51	82.6
		PHE-114	3.69	156.5
		TYR-230	3.87	109.9
		TYR-230	3.73	131.4
		PRO-231	3.44	119.7
		GLN-334	3.62	144.0
	Hydrogen bond	GLN-241	3.00	98.9
		ARG-251	2.92	141.9
		PRO-333	3.09	106.5
		GLY-337	2.72	138.4
Epifriedelanol	Hydrophobic interaction	PHE-111	3.92	88.3
		PHE-224	3.72	92.4
		TYR-228	3.54	158.9
		PRO-231	3.38	81.7
		GLN-334	3.96	163.8
		LEU-340	3.85	134.8
Alpha-Amyrin	Hydrophobic interaction	PHE-111	3.60	122.3
		PRO-231	3.33	103.1
		VAL-336	3.87	89.0
		LEU-340	3.94	111.4
Lupeol	Hydrophobic interaction	PHE-111	3.75	124.2
		PHE-224	3.88	93.0
		PRO-231	3.34	90.3
		VAL-336	3.79	85.2

Among the four compounds, Arnidiol establishes the highest number of hydrophobic interactions with the HIF-1α target protein. Furthermore, it is distinguished by its distinctive ability to establish hydrogen bond interactions with the protein. The distinctive interaction profile might be attributed to its distinct docking posture. The remaining three compounds demonstrate solely hydrophobic interactions with the target protein via saturated hydrocarbon chains. Specifically, Epifriedelanol forms six hydrophobic interactions with the residues PHE111, PHE224, TYR228, PRO231, GLN334, and LEU340. Alpha-Amyrin establishes four hydrophobic interactions with PHE111, PRO231, VAL336, and LEU340. Lupeol also establishes four hydrophobic interactions, targeting residues PHE111, PHE224, PRO231, and VAL336. The last two compounds exhibit the fewest interactions with the HIF-1α target protein, corresponding to the lowest binding affinity to the target, which aligns with the results of the previous MM-PBSA binding free energy calculations.

We observed that the dihydroxy groups of Arnidiol form strong hydrogen bonds with the ARG-251 and GLY-337 residues of the target protein, characterized by short bond lengths and nearly linear bond angles, while forming slightly weaker hydrogen bonds with the GLN-241 and PRO-333 residues. The cooperative effects of this multi-hydrogen-bond network reduce the compound’s conformational freedom to stabilize its conformation. Arnidiol’s optimal docking conformation enables better adaptation to the binding pocket of the target protein, facilitating the formation of intermolecular interactions and enhancing binding affinity. In contrast, the other three compounds possess only monohydroxy structures. Minor local conformational changes could easily disrupt their hydrogen bonds without compensatory alternative sites, while also compromising the maintenance of hydrophobic interactions. Therefore, introducing hydroxyl groups to construct multi-hydrogen-bond networks should be considered in future lead compound optimization efforts. Additionally, methoxy (-OCH_3_) or amino (-NH_2_) groups can be introduced at the ortho or para positions of hydroxyl groups. These electron-donating substituents enhance the polarity of the O-H bond through resonance effects, thereby augmenting the hydrogen bond donor capacity of the hydroxyl group. Alternatively, replacing hydroxyl groups with guanidinyl moieties—which possess three more polarized N-H bonds—could simultaneously strengthen hydrogen bonding and target multiple residues to form hydrogen bond networks. Without affecting the binding space, hydrophobic groups such as methyl groups can be introduced when necessary to counterbalance the increased polar solvation energy arising from hydrogen-bond optimization. These structure-based drug design approaches are expected to further enhance compound binding affinity with HIF-1α and optimize the activity of lead compounds.

Furthermore, it is also essential to focus on critical topological structures and binding sites associated with HIF-1α functional activity in molecular modeling studies. HIF-1α comprises a total of 826 amino acids, with its N-terminal basic region serving as the DNA-binding domain that interacts with hypoxia response elements (HREs) in downstream target genes. Subsequent HLH and PAS domains constitute the HIF-1β binding sites, mediating the formation of heterodimers (Xu et al., 2022). The central ODD domain (401-600), which contains the N-terminal transcriptional activation domain (N-TAD, 531-575), serves as the VHL protein binding site. The Pro402 and Pro564 residues within this domain undergo hydroxylation by oxygen-dependent prolyl hydroxylases (PHDs), while Lys532 is acetylated by acetyltransferase-1 (ARD-1), both processes being critically involved in the VHL-mediated ubiquitination degradation pathway. The C-terminal transcriptional activation domain (786-826) serves as the transcriptional coactivator binding site, with its Asn803 residue directly participating in the recruitment of p300/CBP coactivators (Wu et al., 2021). These key binding sites and functional residues also provide a theoretical foundation for developing HIF-1α-targeted inhibitors.

In previous research, Yadav PK et al. generated a compound with the highest potential as an HIF-1α inhibitor via the side-chain hopping approach based on the outcome of pharmacophore model screening. The molecular docking results demonstrated that this compound interacted with residues TYR102, TYR145, GLN147, THR196, HIS199, ASP201, and LYS214 of the HIF-1α protein (Yadav et al., 2023). Latha MS et al. conducted a virtual screening study for HIF-1 inhibitors based on molecular docking, discovering residues ARG14, LYS17, MET18, TYR21, ASN34, VAL35, SER36, and ARG46 as key interacting residues with potential inhibitors (Latha and Saddala, 2017). Intriguingly, the potential HIF-1α inhibitors identified through our innovative multi-level virtual screening system presented a distinct interaction pattern from previous studies. This unique interaction pattern provided a novel perspective that contributed to the exploration and optimization of potential HIF-1α inhibitors.

### 3.6 Molecular dynamics simulations

**FIGURE 8:**
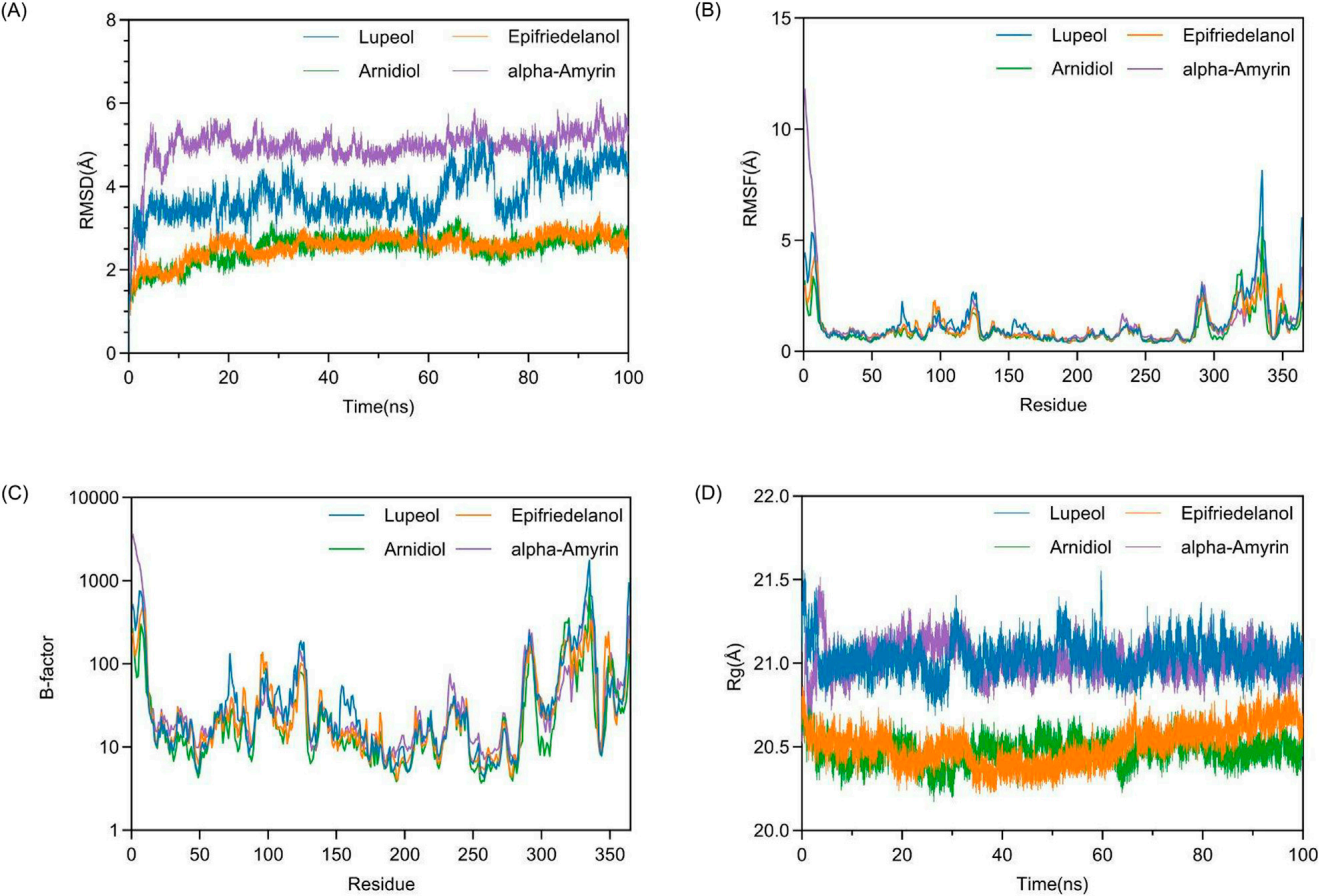
Analysis of structural stability and dynamics in molecular dynamics simulations: **(A)** RMSD, **(B)** RMSF **(C)** B-factor, **(D)** Rg.

To achieve a comprehensive understanding and predict the interactions and dynamic alterations of the complex system at the molecular level, we performed 100 ns molecular dynamics simulations on four protein-ligand complexes using the Amber software. The results are shown in Figure 8. In Figure 8, the images located in the upper left, upper right, lower left, and lower right are respectively Figures 8A–D.

#### 3.6.1 Dynamic stability and conformational flexibility

Firstly, we assessed the conformational stability of complex systems during molecular dynamics simulation by measuring the Root-Mean-Square Deviation (RMSD). In Figure 8A, we observed that at the onset of the simulation, the complex system transitioned from its initial conformation towards an equilibrium state, with all complex systems exhibiting a certain degree of fluctuation. After 5 ns, the RMSD values of the four complex systems commenced stabilization. Between 30 ns and 40 ns, the Lupeol-HIF1α complex system experienced a minor fluctuation of approximately 1 Å, which was within the acceptable range and could be considered as the system remaining in equilibrium. In the final 10 ns of the simulation, all complex systems had reached dynamic equilibrium, demonstrating that the 100 ns molecular dynamics simulation was sufficient. For a protein with a defined PDB structure, the RMSD values of the complexes it forms being below 3 Å are considered acceptable (Kufareva and Abagyan, 2012). Compared to other systems, the Arnidiol-HIF1α and Epifriedelanol-HIF1α complex systems exhibited lower average RMSD values, with 2.51 Å and 2.56 Å, respectively. This indicated that the degree of deviation from the initial conformation of these two systems was minimal, and the overall structure was the most stable. This finding was also consistent with the MM-PBSA binding free energy calculations presented in the previous section.

Previous studies have indicated that the RMSF fluctuation of a complex not exceeding 2 Å is regarded as within an acceptable range (Rahimi et al., 2023). The calculation of RMSF in Figure 8B illustrates that the RMSF variation trends of the main chains of all complexes are broadly consistent. The residues at the N-terminal and C-terminal ends of the protein lack stable interactions, such as internal hydrophobic interactions, thereby resulting in larger RMSF values. Additionally, the residues 310–340 in proximity to the active site also exhibit elevated RMSF values. The high fluctuations of these residues may be associated with the function of the active site. In Figure 8C, the computational analysis of B-factor showed that all complex systems exhibit two distinct regions with high B-factor values, corresponding to areas with high RMSF values. Future research can focus on selectively optimizing non-active site residues with high B-factor values to enhance the thermodynamic stability of the complexes while preserving their functionality.

We subsequently assessed the structural compactness of the complex systems by computing the Rg. The calculation of Rg in Figure 8D illustrates that during the initial 2 ns of the simulation, the Rg values of all protein-ligand complexes exhibited fluctuations to varying degrees. After transitioning to the equilibrium state, the Rg values of the four complex systems remained relatively stable throughout the simulation without significant structural contraction or expansion. In comparison, the Rg values of the Arnidiol-HIF1α and Epifriedelanol-HIF1α complex systems exhibited smaller fluctuations and lower average values of 20.47 Å and 20.50 Å, respectively, indicating a more compact overall structure.

**FIGURE 9:**
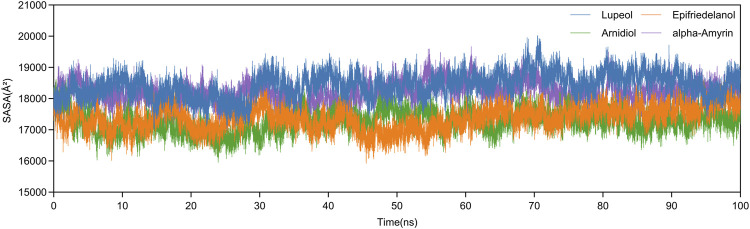
The changes in SASA of four complexes during the molecular dynamics simulation process.

Additionally, we analyzed the SASA variations of four complexes during molecular dynamics simulations. As shown in Figure 9, all systems exhibited SASA fluctuation trends consistent with Rg values throughout the 100ns simulations, maintaining stability before simulation termination. The mean SASA values of Arnidiol-HIF-1α and Epifriedelanol-HIF-1α are 17269.5 Å^2^ and 17376.4 Å^2^ respectively, which are significantly lower than those of the other two complexes. This indicates these two complexes possess minimal solvent-exposed surface area with the most compact structural arrangements. Collectively, these molecular dynamics simulation results consistently demonstrate the tight and stable binding capabilities of Arnidiol and Epifriedelanol with HIF-1α.

**FIGURE 10:**
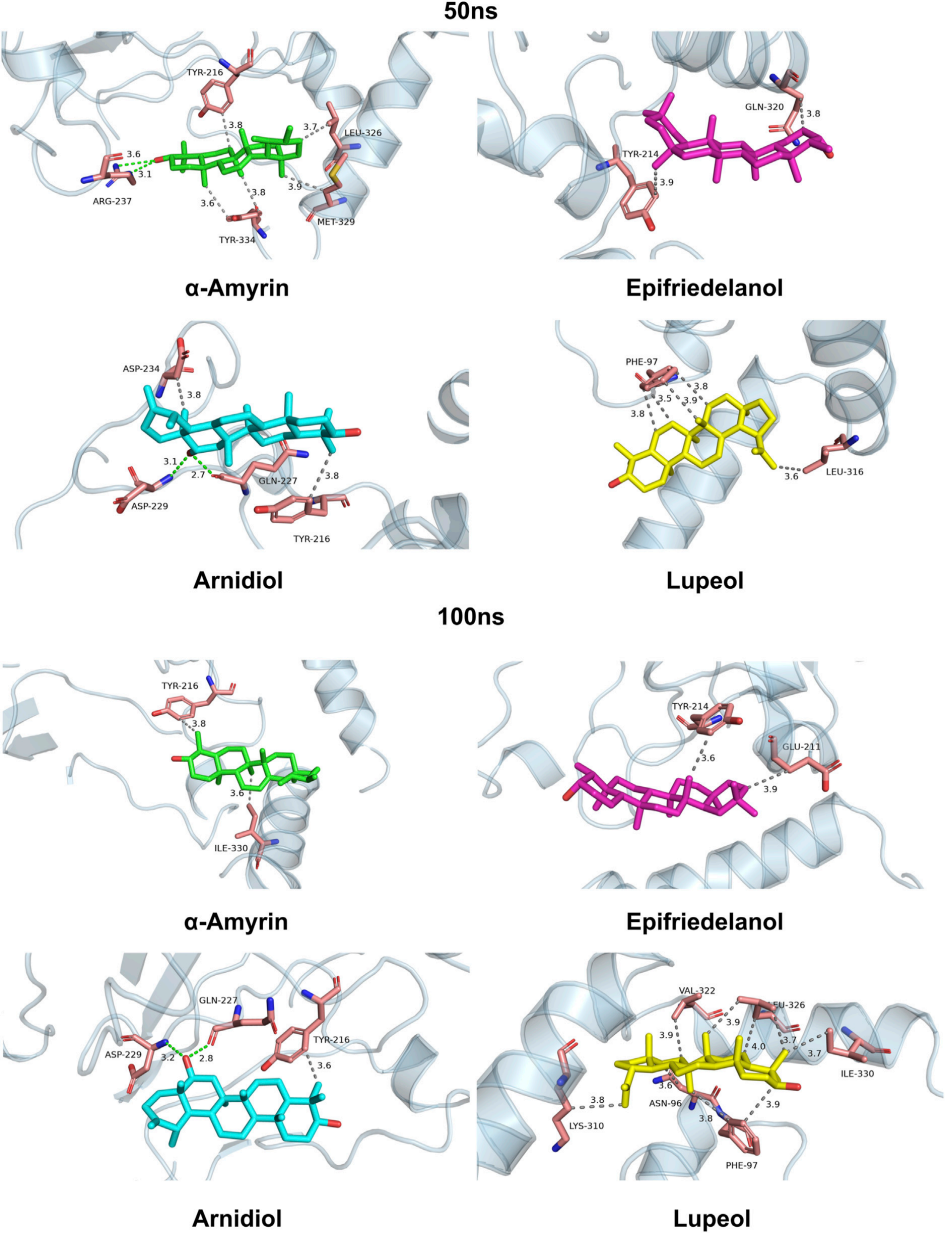
Binding modes of four complexes at 50 ns and 100 ns during molecular dynamics simulations.

To investigate the dynamic protein-ligand interaction patterns, we also visualized the binding modes of four complexes at 50 ns and 100 ns time points during molecular dynamics simulations in Figure 10. Our findings revealed that compound Arnidiol demonstrates remarkable advantages. The hydrogen-bond network formed with residues GLN-227 and ASP-229 remained stable throughout the simulation, while hydrophobic interactions optimized from residues TYR-216 and ASP-234 to retain only the critical anchor residue TYR-216, indicating highly converged binding modes. This combination of dominant polar interactions and streamlined hydrophobic interactions suggests that Arnidiol establishes an energy-stable and specific binding with HIF-1α, emerging as a reliable drug candidate with clear optimization potential in pharmaceutical design.

#### 3.6.2 Dynamical cross-correlation matrix

**FIGURE 11:**
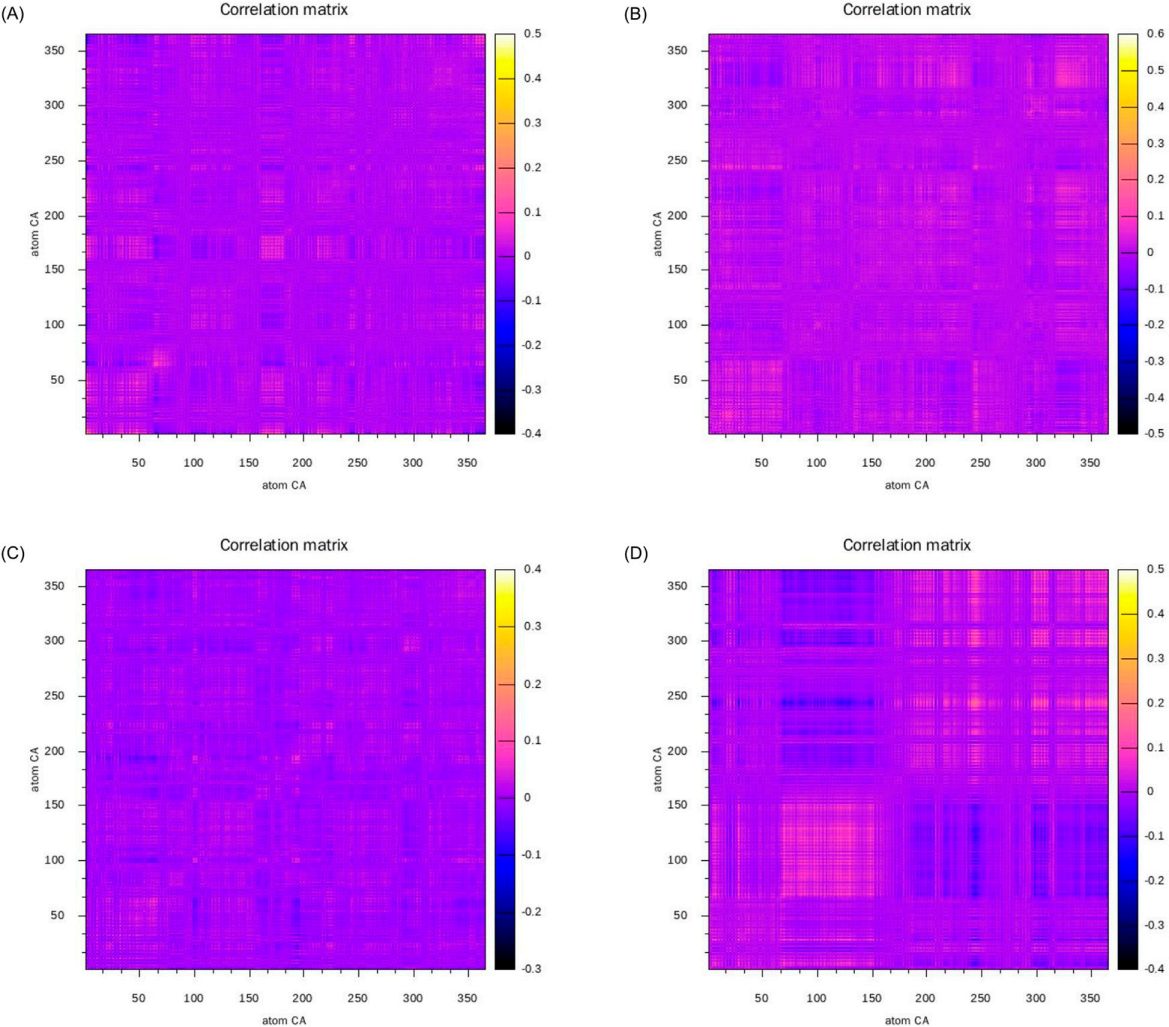
Dynamic cross-correlation matrices of the complexes: **(A)** alpha-Amyrin, **(B)** Arnidiol **(C)** Epifriedelanol, **(D)** Lupeol.

To investigate the correlations between different residues during the simulation process and comprehend the dynamic characteristics and functions of proteins, we constructed the dynamical cross-correlation matrix (DCCM) of four complex systems. Subsequent to aligning the protein structures, we computed the deviation vectors of each residue’s α-carbon atom relative to its average position. Based on the deviation vectors, the covariance matrices and correlation coefficients were further calculated to obtain the DCCM diagrams of the four complex systems as shown in Figure 11. In Figure 11, the images located in the upper left, upper right, lower left, and lower right are respectively Figures 11A–D. Positively correlated motions between residues are represented in yellow and orange. Negatively correlated motions are depicted in blue and black. Uncorrelated motions are indicated in purple. At the diagonal position, the same α-carbon atom exhibits the highest self-correlation, indicating that its own motion pattern remains completely consistent throughout the time series. The correlations on both sides of the diagonal usually indicate that adjacent residues form a tight secondary structure such as α-helix, β-sheet, or β-turn.

The correlated motions of active site residues are crucial for the execution of protein function. Specifically, in the Arnidiol-HIF1α complex, residues 320–340 located near the active site demonstrated markedly positive correlated motions. In the Epifriedelanol-HIF1α system, the residues near the active site manifested certain correlated motions, albeit with slightly diminished intensity compared to Arnidiol. In the Lupeol-HIF1α system, the residues in proximity to the active site also exhibited a regular trend of correlated motion. Residues 66–150 displayed significantly positive correlated motions with each other, while also exhibiting significantly negative correlated motions with residues 166–250 and 295–364. Residues 166–364 also had interrelated positive correlated motions among themselves. Meanwhile, we observed that the adjacent residues flanking the diagonal in the above three systems showed significant correlations, indicating a tight and stable secondary structure. In contrast, no notable correlated motions are detected near the active site residues in the alpha-amyrin complex system. Moreover, the distribution of positive and negative correlated motion regions was comparatively disordered, suggesting lower structural stability. This may account for its higher mean RMSD.

#### 3.6.3 Free energy landscape

**FIGURE 12:**
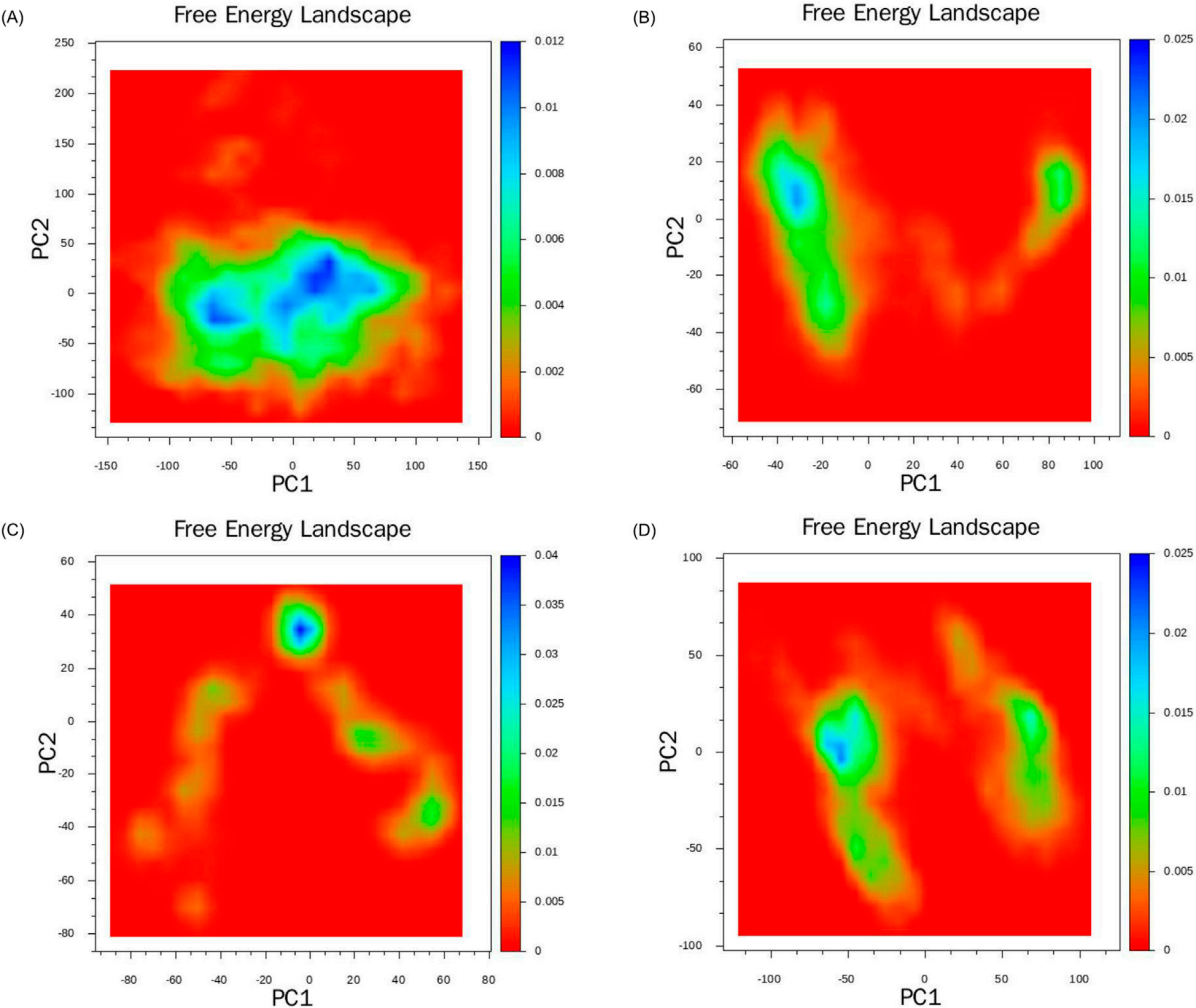
Free energy landscapes of the complexes: **(A)** alpha-Amyrin, **(B)** Arnidiol **(C)** Epifriedelanol, **(D)** Lupeol.

Based on the data from molecular dynamics simulations, we initially computed the covariance matrix of the spatial coordinates. Using the two most significant eigenvectors, PC1 and PC2, obtained from the covariance matrix decomposition as the coordinate axes, we constructed the free energy landscapes for the four complex systems. In Figure 12, the images located in the upper left, upper right, lower left, and lower right are respectively Figures 12A–D. The blue region denotes areas of higher energy, namely, energy barriers. The red region, on the other hand, denotes areas of lower energy, namely, energy wells. In all four complex systems. We observed several widely significant energy wells in all four complex systems, indicating that the four ligands can strongly bind to the target protein and form multiple stable complex conformations. Compared to other systems, the Lupeol-HIF1α complex exhibits a degree of minor relative free energy differences among its energy wells, indicating the existence of distinct stable conformations. Meanwhile, the transitions between stable conformations require overcoming two significant energy barriers of varying magnitudes. Therefore, the corresponding RMSD trajectory in Figure 8A displays two moderate fluctuations at 60 ns and 80 ns. The Arnidiol-HIF1α complex also exhibits two minor energy barriers, with the corresponding RMSD trajectory showing slight fluctuations at 10 ns and 25 ns, respectively. The Epifriedelanol-HIF-1α complex presents a significant energy barrier and several scattered minor energy barriers. Consequently, the RMSD trajectory exhibits noticeable fluctuations between 5 ns and 10 ns, succeeded by several subtle oscillations. The Arnidiol-HIF1α and Epifriedelanol-HIF1α complexes exhibit the lowest energy wells with the widest ranges, indicating the most stable complex conformations. The alpha-Amyrin complex system possesses an extensive and sizable energy barrier, indicative of higher conformational instability. This is consistent with the mean RMSD value observed in Figure 8A.

## 4 Discussion

The multi-level virtual screening strategy of “machine learning - molecular docking - molecular dynamics simulation” adopted in this study successfully achieved the virtual screening and mechanism analysis of HIF-1α inhibitors, verifying the efficiency and reliability of combining artificial intelligence with classical CADD methods for drug discovery. In this study, we separately computed 208 RDKit molecular descriptors and 300-dimensional Mol2Vec molecular structure vector descriptors for the training and test datasets. Based on these two molecular features, six distinct machine learning models were constructed by three machine learning algorithmic: random forest (RF), support vector machine (SVM), and extreme gradient boosting (XGBoost). In the subsequent model performance evaluation, the random forest model based on RDKit molecular descriptors (RDKit-RF) exhibited superior performance, with an AUC value of 0.918 and an MCC value of 0.781. Consequently, we employed the RDKit-RF model to conduct virtual screening of the Traditional Chinese Medicine Monomer Library developed by Topscience, identifying 104 compounds with “active” values exceeding 0.8 for molecular docking. Based on the results of molecular docking, we further selected eight compounds with docking scores below −8.5 kcal/mol and computed their MM-PBSA binding free energies. Taking the known effective HIF-1α inhibitor, 7-Hydroxylamellarin A, as a reference, the total binding free energies of the compounds Epifriedelanol, Arnidiol, alpha-Amyrin, and Lupeol were lower than that of the reference compound 7-Hydroxylamellarin A (−19.5983 ± 3.4421 kcal/mol). Among them, the MM-PBSA binding free energy of the compound Epifriedelanol was the lowest, at −27.2463 ± 2.2615 kcal/mol. We considered Epifriedelanol and the other three compounds to be the most likely HIF-1α inhibitors, and conducted docking interaction analysis and 100 ns molecular dynamics simulations on them. We found that the potential HIF-1α inhibitors screened by the multi-level virtual screening system combining machine learning and CADD methods exhibited different target protein interaction patterns compared to previous studies. This may provide a new perspective for the exploration and design of HIF-1 α inhibitors. The results of molecular dynamics simulations indicate that the compounds Arnidiol and Epifriedelanol form the most stable conformations with the HIF-1α protein, holding promise as potential HIF-1α inhibitors.

In the future, we will integrate data from multiple databases such as ChEMBL and Pubchem to enrich the sample diversity of our research. Additionally, we will focus on building a fusion system of deep learning models and existing machine learning frameworks to fully explore the three-dimensional binding features in molecular graph data and reduce the potential false positive rate of existing algorithms. We also plan to incorporate multiple molecular docking algorithms including Glide and AutoDock Vina, establishing a multi-dimensional scoring system to overcome potential biases associated with single-algorithm approaches, On the basis of verifying the effectiveness of current targeted inhibitors, we will also extend the screening strategy to key targets such as PHD2 and FIH-1 in the HIF-1α pathway to explore the possibility of developing multi-target inhibitors or agonists. Meanwhile, we will also evaluate the value of compounds in HIF-1α-related disease such as ischemic cardiovascular disease, chronic inflammation and autoimmune diseases to further enhance the application value of this study.

## 5 Conclusion

In this study, we employed a three-tier innovative virtual screening system integrating machine learning, molecular docking, and molecular dynamics simulations to comprehensively screen potential HIF-1α-targeting inhibitors from the traditional chinese medicine monomer database containing 2,560 natural compounds developed by Topscience. Through sequential evaluation via machine learning model scoring, binding affinity analysis, and MM-PBSA binding free energy calculations, four compounds demonstrating theoretical superiority over the known HIF-1α inhibitor 7-hydroxy amiraprotein A were identified. Subsequent molecular dynamics simulations ultimately revealed two most potent potential HIF-1α inhibitors: Arnidiol and Epifriedelanol. In future, these compounds hold promise for advancing more effective therapeutic strategies in cancer treatment after further experimental validation.

## Data Availability

The original contributions presented in the study are included in the article/Supplementary Material, further inquiries can be directed to the corresponding author.
