# Supplementary material for: Identification of novel potential hypoxia-inducible factor-1α inhibitors through machine learning and computational simulations
Source: Front Chem. 2025 May 12;13:1585882. doi: 10.3389/fchem.2025.1585882 (PMC12104167; doi:10.3389/fchem.2025.1585882)
Supplement: Supplementary file 1 [file Table1.docx]

Supplementary Material

# Supplementary Tables

**
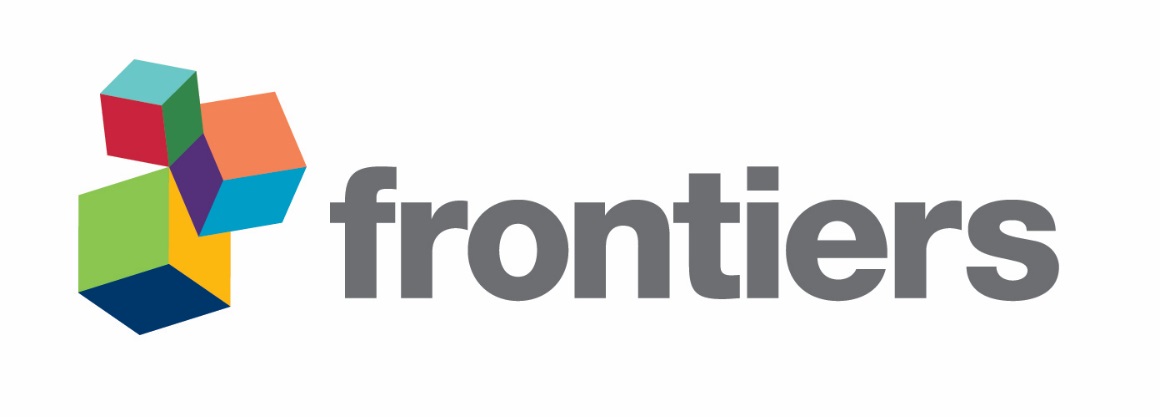
**

**Supplementary Table 1.**The Optimal Hyperparameter Combinations of the RF Model

| Model | max_depth | min_samples_leaf | n_estimators |
| --- | --- | --- | --- |
| RDkit-RF | 8 | 3 | 256 |
| Mol2Vec-RF | 13 | 5 | 255 |

**Supplementary Table 2.** The Optimal Hyperparameter Combinations of the SVM Model

| Model | C | Gamma |
| --- | --- | --- |
| RDkit -SVM | 3 | 0.01 |
| Mol2Vec -SVM | 100 | 0.001 |

**Supplementary Table 3.** The Optimal Hyperparameter Combinations of the XGBoost

| Model | colsample_bytree | gamma | learning_rate | max_ depth | min_child_weight | n_estimators | subsample |
| --- | --- | --- | --- | --- | --- | --- | --- |
| RDkit -XGBoost | 0.95 | 0.06 | 0.07 | 4 | 2 | 80 | 0.95 |
| Mol2Vec-XGBoost | 0.96 | 0.09 | 0.09 | 4 | 2 | 84 | 0.94 |

**Supplementary Table 4.** Compounds inhibitory activity scores based on the RDKit-RF model

| smiles | active |
| --- | --- |
| COc1ccc(/C=C/c2cc(O)cc(O[C@@H]3O[C@H](CO)[C@@H](O)[C@H](O)[C@H]3O)c2)cc1 | 0.672479046 |
| C=C(C)[C@@H]1CC[C@]2(CO)CC[C@]3(C)[C@H](CC[C@@H]4[C@@]5(C)CC[C@H](O)C(C)(C)[C@@H]5CC[C@]43C)[C@@H]12 | 0.900066406 |
| O=C(/C=C/c1ccccc1)c1ccccc1 | 0.659681362 |
| C[N+](C)(C)C[C@@H](O)CC(=O)[O-] | 0.558892425 |
| COc1cc(-c2cc(=O)c3c(O)c([C@@H]4O[C@H](CO)[C@@H](O)[C@H](O)[C@H]4O)c(O)cc3o2)ccc1O | 0.523489858 |
| O[C@H]1[C@H]2c3cc4c(cc3CN3CCC(=C[C@@H]1O)[C@H]23)OCO4 | 0.504905159 |
| COc1cc(/C=C/C(O)=C/C(=O)CCc2ccc(O)c(OC)c2)ccc1O | 0.621419096 |
| COc1ccc(C[C@@H]2COc3c(C)c(O)c(C)c(O)c3C2=O)cc1 | 0.600702806 |
| C=C(C)[C@@H]1CC=C(CO)CC1 | 0.552081478 |
| COC(=O)c1c(C)cc(OC(=O)c2c(C)cc(O)c(C=O)c2O)c(C)c1O | 0.543715596 |
| C/C1=C\CC(C)(C)/C=C/C(=O)/C(C)=C/CC1 | 0.661211151 |
| CCCCCCCCCCCCCCCCCCCCCCO | 0.516348055 |
| Oc1cc(O)cc(/C=C/c2ccc(O)cc2O)c1 | 0.69847354 |
| Nc1ccn([C@@H]2O[C@H](CO)[C@@H](O)[C@@H]2O)c(=O)n1 | 0.667053991 |
| COc1cc(O)c(CC=C(C)C)c2c1C(=O)C[C@@H](c1ccc(O)cc1)O2 | 0.607786849 |
| Nc1ncnc2c1ncn2[C@@H]1O[C@H](CO)[C@@H](O)[C@@H]1O | 0.671111893 |
| C[C@@H]1O[C@@H](O[C@@H]2[C@@H](O)[C@H](OCCc3ccc(O)c(O)c3)O[C@H](CO)[C@H]2OC(=O)/C=C/c2ccc(O)c(O)c2)[C@H](O)[C@H](O)[C@H]1O | 0.492995943 |
| CC1(C)[C@@H]2CC[C@@]1(C)[C@H](O)C2 | 0.512554996 |
| C=C(C)[C@@H]1CC[C@]2(C(=O)O)CC[C@]3(C)[C@H](CC[C@@H]4[C@@]5(C)CCC(=O)C(C)(C)[C@@H]5CC[C@]43C)[C@@H]12 | 0.80295868 |
| CCCCCCCCOC(=O)c1cc(O)c(O)c(O)c1 | 0.675569075 |
| COc1cc(O)c(CC=C(C)C)c(O)c1C(=O)/C=C/c1ccc(O)cc1 | 0.611214655 |
| Nc1ncnc2c1ncn2[C@@H]1O[C@H](COP(=O)(O)O)[C@@H](O)[C@H]1O.O | 0.663099401 |
| C[C@H]1CCC/C=C/[C@@H]2C[C@H](O)C[C@H]2[C@H](O)/C=C/C(=O)O1 | 0.648764358 |
| C/C=C(\C)C(=O)O | 0.614580127 |
| O=C1c2cccc(O)c2C(=O)c2c(O)cc(CO)cc21 | 0.571635591 |
| O=Cc1cc(O)c(O)c(O)c1 | 0.511042413 |
| Cc1nccc2c1[nH]c1cc(O)ccc12 | 0.509754029 |
| Cc1nccc2c1[nH]c1ccccc12 | 0.518015505 |
| CCCn1c(=O)c2[nH]c(C3CCCC3)nc2n(CCC)c1=O | 0.547416159 |
| CC(C)=CCc1c(O)ccc2ccc(=O)oc12 | 0.700999505 |
| O=C(/C=C/c1ccc(O)c(O)c1)O[C@H](Cc1ccc(O)c(O)c1)C(=O)O | 0.633861246 |
| COc1cc(/C=C/c2ccccc2)oc(=O)c1 | 0.667415465 |
| O=C1Nc2ccccc2C1=O | 0.481008289 |
| COc1ccc(C(C)=O)c(O)c1 | 0.524058184 |
| COc1ccc2ccc(=O)oc2c1CC=C(C)C | 0.695750102 |
| C[C@H](CC(=O)C[C@@H](C)[C@H]1C[C@H](O)[C@@]2(C)C3=C(C(=O)C[C@]12C)[C@@]1(C)CC[C@H](O)C(C)(C)[C@@H]1C[C@@H]3O)C(=O)O | 0.747038362 |
| CN1CCc2cc3c(cc2C(=O)Cc2ccc4c(c2C1)OCO4)OCO3 | 0.536432203 |
| O=C(O)/C=C\c1cc2ccoc2cc1O[C@@H]1O[C@H](CO)[C@@H](O)[C@H](O)[C@H]1O | 0.715858498 |
| CC(=O)NCCc1cnc[nH]1 | 0.680617818 |
| NCCc1c[nH]cn1 | 0.611900224 |
| COc1c(O)cc2oc(-c3ccc(O)c(O)c3)cc(=O)c2c1O | 0.639075722 |
| COc1ccc([C@@H]2CC(=O)c3c(O)cc(O)cc3O2)cc1O | 0.588669138 |
| COc1cccc(O)c1-c1cc(=O)c2c(O)c(OC)c(OC)c(OC)c2o1 | 0.557607841 |
| C=C1C(=O)O[C@@H]2C[C@@]3(C)CCC[C@H](C)C3=C[C@H]12 | 0.630979772 |
| COc1cc(O[C@@H]2O[C@H](CO[C@@H]3O[C@H](CO)[C@@H](O)[C@H](O)[C@H]3O)[C@@H](O)[C@H](O)[C@H]2O)c2c(O)c3c(=O)cc(C)oc3cc2c1 | 0.441299053 |
| C[C@H]1C(=O)O[C@@H]2CCN3CC=C(COC(=O)[C@](C)(O)[C@]1(C)O)C23 | 0.51970625 |
| O=C1C(=C(O)C=Cc2ccc(O)cc2)C(O)=C(C2OC(CO)C(O)C(O)C2O)C(=O)C1(O)C1OC(CO)C(O)C(O)C1O | 0.419602426 |
| O=C1CCC[C@@H]2[C@H]3CCC[N+]4([O-])CCC[C@@H](CN12)[C@@H]34 | 0.651251961 |
| CC=C1[C@H]2C=C(C)C[C@]1(N)c1ccc(=O)[nH]c1C2 | 0.580468098 |
| C=CC(C)(O)CCC=C(C)C | 0.558308682 |
| C=CCc1cc(OC)c(OC)c(OC)c1 | 0.619469025 |
| Cc1ccc(C(C)C)cc1O | 0.54469055 |
| C=C[C@@H]1C2=CCOC(=O)C2=CO[C@H]1O[C@@H]1O[C@H](CO[C@@H]2O[C@H](CO)[C@@H](O)[C@H](O)[C@H]2O)[C@@H](O)[C@H](O)[C@H]1O | 0.496163216 |
| CC(C)=CCC1=CC(=O)c2ccccc2C1=O | 0.639586144 |
| CC(C)=CCc1c(O)cc(O)c2c(=O)cc(-c3ccc(O)cc3)oc12 | 0.551898822 |
| C=CC(C)(O)CCC=C(C)CCC=C(C)C | 0.666349073 |
| CC(C)c1ccc(C(=O)O)cc1 | 0.629270945 |
| O=C(/C=C/c1ccc(O[C@@H]2O[C@H](CO)[C@@H](O)[C@H](O)[C@H]2O)cc1)c1ccc(O)cc1O | 0.684019315 |
| COc1ccc(/C=C/C(=O)c2ccc(O)cc2)c(OC)c1 | 0.728436656 |
| COc1cc(/C=C/CO)ccc1O | 0.527069682 |
| COC1=C2CC(C)=c3c(C(C)=O)c(OC)c(O)c4c3c2c2c(cc(OC)c3c(OC)cc(O)c4c32)C(=O)C1=O | 0.405990267 |
| CSC(=S)NCc1c[nH]c2ccccc12 | 0.666333477 |
| COC1=CC(=O)c2ccccc2C1=O | 0.489256671 |
| Oc1cccc(O)c1O | 0.496467805 |
| CC(=O)O[C@H]1CC[C@]2(C)[C@H]3C(=O)C=C4[C@@H]5[C@@H](C)[C@H](C)CC[C@]5(C)CC[C@@]4(C)[C@]3(C)CC[C@H]2C1(C)C | 0.806106001 |
| COc1cccc(OC)c1C(=O)O | 0.575844477 |
| COC(=O)C1=CCC23CCC(C(C)(C=CC=C(C)C(=O)O)OC2=O)C3(OC(C)=O)CC1 | 0.556007492 |
| COc1ccc(C(O)CNC(=O)/C=C/c2ccccc2)cc1 | 0.631092419 |
| COc1cc(/C=C/CO)ccc1O[C@@H]1O[C@H](CO)[C@@H](O)[C@H](O)[C@H]1O | 0.634017548 |
| CC(C)=CCC1=C(O)C(=O)c2ccccc2C1=O | 0.615899498 |
| CC(=O)c1ccc2c(c1)C=CC(C)(C)O2 | 0.449937864 |
| O=C1C=C(O)C(=O)c2ccccc21 | 0.43327024 |
| CC(C)=CCOc1ccc2ccc(=O)oc2c1 | 0.707335697 |
| CCCC[C@@H]1OC(=O)C2=CCCC[C@H]21 | 0.596602285 |
| C[C@H]1CC[C@@]2(O)C(C)(C)[C@@H]3CC[C@@]2(C)[C@@H]1C3 | 0.687327592 |
| Cc1cc(=O)oc2cc(O)cc(O)c12 | 0.519005226 |
| O=C1c2ccccc2C(=O)c2c(O)ccc(O)c21 | 0.555261769 |
| COC(=O)/C=C\c1ccc(O)cc1 | 0.606645153 |
| COc1cc(OC)c2c(=O)cc(-c3ccc(OC)c(OC)c3)oc2c1 | 0.583073468 |
| COc1cc2c(cc1OC)C1Cc3ccc4c(c3CN1CC2)OCO4 | 0.486596381 |
| OCCc1ccc(O)c(O)c1 | 0.538441865 |
| COc1c(O)cc2oc3cc(O)c(CC=C(C)C)c(O)c3c(=O)c2c1CC=C(C)C | 0.577506793 |
| COc1c(O)cc2c(c1O)[C@@H]1O[C@H](CO)[C@@H](O)[C@H](O)[C@H]1OC2=O | 0.708816118 |
| Cc1ccc(/C=C/C(=O)O)cc1 | 0.573077098 |
| CCOC(=O)c1ccc(O)cc1 | 0.594705661 |
| COC(=O)C1=CC[C@@]23CC[C@@H]([C@@](C)(/C=C/C=C(\C)C(=O)O)OC2=O)[C@@]3(O)CC1 | 0.626309131 |
| O=C(O)c1ccccc1 | 0.633535535 |
| CC(=O)O[C@H]1Cc2cc3c(cc2[C@H]2N(C)Cc4c(ccc5c4OCO5)[C@@]12C)OCO3 | 0.526340867 |
| CCCCCCCCCCC(=O)O | 0.662907197 |
| CC1(C)C=Cc2cc3ccc(=O)oc3cc2O1 | 0.58993144 |
| COc1cc2c(cc1O)CCN1Cc3c(ccc(OC)c3OC)CC21 | 0.489974102 |
| CC1=CC=C(C(C)C)CC1 | 0.582441218 |
| O=c1c(O[C@@H]2O[C@H](CO)[C@H](O)[C@H](O)[C@H]2O)c(-c2ccc(O)c(O)c2)oc2cc(O)cc(O)c12 | 0.501438229 |
| COn1cc(CC#N)c2ccccc21 | 0.514649884 |
| CC(C)=CCC/C(C)=C\CO | 0.599266201 |
| CCOC(=O)c1ccc(O)c(OC)c1 | 0.548864989 |
| COc1cc2c(cc1O)[C@@H]1Cc3ccc(OC)c(OC)c3CN1CC2 | 0.479259817 |
| CC(C)=CCc1c(O)cc(O)c2c(=O)c(-c3ccc(O)cc3O)coc12 | 0.574739366 |
| CC(=O)c1c(O)ccc2ccc(=O)oc12 | 0.538335607 |
| c1ccc2c(c1)C[C@H]1NCCc3cc4c(c-2c31)OCO4 | 0.502560395 |
| COc1cc(/C=C/c2cc(O)cc(O[C@@H]3O[C@H](CO)[C@@H](O)[C@H](O)[C@H]3O)c2)ccc1O | 0.646897555 |
| C=CCc1ccc(OC(C)=O)c(OC)c1 | 0.665396398 |
| O=C1c2ccccc2C(=O)c2c(O)c(O)cc(O)c21 | 0.558215331 |
| COc1cc(C=O)cc(O)c1O | 0.475942133 |
| CN1Cc2c(ccc3c2OCO3)-c2ccc3cc4c(cc3c21)OCO4 | 0.502556473 |
| C[C@@H]1CC[C@H]2C(C)(C)[C@H]3C[C@]12CC[C@@]3(C)O | 0.643546164 |
| COc1cc(-c2c3c(c(O)c4cc5c(cc24)OCO5)COC3=O)cc(OC)c1OC | 0.608776629 |
| COc1ccc2c(c1O)-c1c(O)c(OC)cc3c1[C@H](C2)[N+](C)(C)CC3 | 0.507284648 |
| COc1cccc(CCc2cc(O)cc(OC)c2)c1 | 0.610011345 |
| CC1(C)C(=O)[C@]2(C)CC[C@H]1C2 | 0.528406303 |
| CC(=O)O[C@@]12CCC(C)=CC[C@]13CC[C@H]2[C@](C)(/C=C/C=C(\C)C(=O)O)OC3=O | 0.572545244 |
| COc1cc(O)c(C(C)=O)c(OC)c1 | 0.561762131 |
| COc1ccc(-c2coc3cc(OC)ccc3c2=O)cc1 | 0.611231065 |
| COc1cc(C[C@H]2COC(=O)[C@]2(O)Cc2ccc(O)c(OC)c2)ccc1O | 0.638285037 |
| Oc1ccc(/C=C/c2cc(O)cc(O)c2)cc1 | 0.657553522 |
| COc1ccc(/C=C/c2cc(O)cc(O)c2)cc1O | 0.639472679 |
| CC(C)CNC(=O)/C=C/C=C/c1ccc2c(c1)OCO2 | 0.653333364 |
| O=Cc1ccc(CO)o1 | 0.599906689 |
| CC1=CCC2C(C1)C2(C)C | 0.544314072 |
| COc1cccc(C=O)c1OC | 0.565207042 |
| CCCCCCCCCCCCCC(=O)OCC(O)CO | 0.529313382 |
| O=c1cc(-c2ccc(O)c(-c3c(O)cc(O)c4c(=O)cc(-c5ccc(O)cc5)oc34)c2)oc2cc(O)cc(O)c12 | 0.408994967 |
| CC(=O)O[C@@H]1C[C@@H]2CC[C@@]1(C)C2(C)C | 0.593097597 |
| C=CCCCCCCCCC(=O)O | 0.630719624 |
| CCCCCCCCCCCCCCCCCCCCC | 0.507088489 |
| COc1c2ccccc2nc2occc12 | 0.468023069 |
| C/C=C/C=C/C(=O)O | 0.633874198 |
| C=CC(C)(O)CC/C=C(\C)CCC=C(C)C | 0.666349073 |
| CC(C)(C)c1cc(O)cc(C(C)(C)C)c1 | 0.587769729 |
| O=c1cc(-c2ccc(O)cc2)oc2c([C@@H]3O[C@H](CO)[C@@H](O)[C@H](O)[C@H]3O)c(O)c([C@@H]3OC[C@H](O)[C@H](O)[C@H]3O)c(O)c12 | 0.419842236 |
| C=C[C@H]1[C@H](O[C@@H]2O[C@H](CO)[C@@H](O)[C@H](O)[C@H]2O)OC=C2C(=O)N3CCc4c([nH]c5ccccc45)[C@@H]3C[C@H]21 | 0.5020199 |
| CC(=O)O[C@]1(C)CC[C@@]23C[C@@H]1C(C)(C)[C@@H]2CC[C@H]3C | 0.710512468 |
| COc1ccc2c(c1OC)CN(C)c1c-2ccc2cc3c(cc12)OCO3 | 0.518462188 |
| C=CC(=C)CC/C=C(\C)CCC=C(C)C | 0.62088002 |
| CC1(C)C=CC2=C(O1)C(=O)c1ccccc1C2=O | 0.557207981 |
| CC(C)=CCc1c(O)cc2occ(-c3ccc(O)cc3)c(=O)c2c1O | 0.555631912 |
| CC(C)CC1NC(=O)C(Cc2ccc(O)cc2)NC1=O | 0.572957525 |
| C/C=C/c1ccc(OC(C)=O)c(OC)c1 | 0.628804797 |
| COc1cc(O)c2c(c1)O[C@H](c1ccc(O)cc1)CC2=O | 0.636998091 |
| CC(C)=CCC/C(C)=C/CO | 0.599266201 |
| COc1ccc(C=O)cc1OC | 0.569368654 |
| C[C@@H]1O[C@@H](Oc2cc(O)c3c(=O)c(O)c(-c4ccc(O)c(O)c4)oc3c2)[C@H](O)[C@H](O)[C@H]1O | 0.51549413 |
| CC(=O)O[C@H]1CC[C@]2(C)[C@H]3CC=C4[C@@H]5CC(C)(C)CC[C@]5(C)CC[C@@]4(C)[C@]3(C)CC[C@H]2C1(C)C | 0.848062382 |
| CC[C@@]1(O)C(=O)OCc2c1cc1n(c2=O)Cc2cc3ccccc3nc2-1 | 0.596316459 |
| CC(C)=CCCC(C)=CCCC(C)=CCCC=C(C)CCC=C(C)CCC=C(C)C | 0.517882842 |
| CC(C)(O)[C@@H]1Cc2c(ccc3ccc(=O)oc23)O1 | 0.624641186 |
| COc1ccccc1C=O | 0.529365119 |
| CC1=CCC(=O)O1 | 0.571126786 |
| C=C1C/C(=C/C)C(=O)O[C@@H]2CCN3CC=C(COC(=O)[C@]1(C)O)[C@H]23 | 0.502911191 |
| Cc1c(C)c2c(c(C)c1O)CC[C@@](C)(CCC[C@H](C)CCC[C@H](C)CCCC(C)C)O2 | 0.624063506 |
| C/C=C/c1ccc(OC)c(OC)c1 | 0.554650803 |
| CC(=O)Oc1ccc2c(C)cc(=O)oc2c1 | 0.68230643 |
| COc1cc(/C=C/COC(=O)/C=C/c2ccc(O)c(OC)c2)ccc1O | 0.652461219 |
| C=C[C@@]1(C)Cc2occ(C)c2C[C@@H]1C(=C)C | 0.587785395 |
| O=c1cc(-c2ccccc2)c2c(O)cc(O)cc2o1 | 0.639378336 |
| C/C=C/C#CC#C/C=C/c1ccco1 | 0.596886356 |
| COc1c(O)cc2oc3cc4c(c(O)c3c(=O)c2c1CC=C(C)C)CCC(C)(C)O4 | 0.570021462 |
| COc1ccc(/C=C/c2cc(O)cc(O)c2)cc1 | 0.596097226 |
| COC(=O)C1=CO[C@@H](O[C@@H]2O[C@H](CO)[C@@H](O)[C@H](O)[C@H]2O)[C@H]2[C@@H]1[C@H](O)C[C@]2(C)O | 0.703228852 |
| C=C(C)[C@H]1Cc2c(ccc3c2O[C@@H]2COc4cc(OC)c(OC)cc4[C@@H]2C3=O)O1 | 0.537225887 |
| O=C(O)c1cccnc1 | 0.678940836 |
| NCCS(=O)(=O)O | 0.610742496 |
| CC(C)=CCCC1(C)C=Cc2c(O)c3c(c(CC=C(C)C)c2O1)OC12C(=CC4CC1C(C)(C)OC2(C/C=C(\C)C(=O)O)C4=O)C3=O | 0.549416969 |
| O=c1c(O)c(-c2ccc(O)c(O)c2)oc2cc(O)cc(O)c12 | 0.589994917 |
| O=C(/C=C/C=C/c1ccc2c(c1)OCO2)N1CCCCC1 | 0.547193702 |
| COc1cc(C=CCO)cc(OC)c1OC1OC(CO)C(O)C(O)C1O | 0.625404242 |
| O=C(O)[C@H](O)Cc1ccc(O)c(O)c1 | 0.606720774 |
| O=C(O[C@@H]1O[C@@H]2COC(=O)c3cc(O)c(O)c(O)c3-c3c(cc(O)c(O)c3O)C(=O)O[C@@H]([C@@H]1O)[C@H]2O)c1cc(O)c(O)c(O)c1 | 0.433045321 |
| CCCCCCCCCCCCCCCC(=O)NCc1ccccc1 | 0.526101775 |
| O=C(O)c1ccccc1O | 0.578419913 |
| C[C@@H]1O[C@@H](O[C@H]2[C@H](Oc3cc(O)c4c(=O)cc(-c5ccc(O)cc5)oc4c3)O[C@H](CO)[C@@H](O)[C@@H]2O)[C@H](O)[C@H](O)[C@H]1O | 0.450129373 |
| CC1(C)CCC2=C(O1)c1ccccc1C(=O)C2=O | 0.550226642 |
| COc1cc(/C=C/C(=O)CC(=O)/C=C/c2ccc(O)cc2)ccc1O | 0.554492081 |
| COc1cc(C(=O)OCCCCNC(=N)N)cc(OC)c1O.Cl | 0.741798275 |
| C=C1C(=O)O[C@H]2[C@H]1CC/C(C)=C/CC[C@@]1(C)O[C@@H]21 | 0.621903559 |
| O=c1cc(-c2ccc(O)cc2)oc2cc(O)cc(O)c12 | 0.617530744 |
| O=C(O)c1cc(O)c2c(c1)C(=O)c1cccc(O)c1C2=O | 0.603435794 |
| C[C@@H]1O[C@@H](O[C@H]2[C@H](Oc3cc(O)c4c(c3)O[C@H](c3ccc(O)cc3)CC4=O)O[C@H](CO)[C@@H](O)[C@@H]2O)[C@H](O)[C@H](O)[C@H]1O | 0.447205791 |
| COc1ccc2cc3[n+](cc2c1OC)CCc1cc2c(cc1-3)OCO2 | 0.481513605 |
| COc1cc([C@@H]2c3cc4c(cc3C[C@H]3COC(=O)[C@@H]32)OCO4)cc(OC)c1OC | 0.53731738 |
| Nc1ncnc2c1ncn2[C@@H]1O[C@H](CO)C[C@H]1O | 0.67326407 |
| COc1cc2c(cc1O)[C@@H](Cc1ccc(O)c(Oc3ccc(C[C@@H]4c5cc(OC)c(OC)cc5CCN4C)cc3)c1)N(C)CC2 | 0.393917549 |
| CC(C)=CCc1c(O)ccc(C(=O)/C=C/c2ccc(O)cc2)c1O | 0.600809436 |
| O=C1c2c(O)cc(O)cc2O[C@H](c2ccc(O)c(O)c2)[C@H]1O | 0.618513797 |
| C[n+]1cc2c3c(ccc2c2ccc4cc5c(cc4c21)OCO5)OCO3.[Cl-] | 0.489598933 |
| COc1cc([C@@H]2c3cc4c(cc3[C@@H](O[C@@H]3O[C@@H]5CO[C@@H](C)O[C@H]5[C@H](O)[C@H]3O)[C@H]3COC(=O)[C@H]23)OCO4)cc(OC)c1O | 0.474161354 |
| O=c1c(O[C@@H]2O[C@@H](CO)[C@H](O)[C@H]2O)c(-c2ccc(O)cc2)oc2cc(O)cc(O)c12 | 0.613960212 |
| O=C1c2c(O)cc(O)cc2O[C@H](c2cc(O)c(O)c(O)c2)[C@H]1O | 0.598644556 |
| COc1c(OC2OC(C(=O)O)C(O)C(O)C2O)cc(O)c2c(=O)cc(-c3ccccc3)oc12 | 0.552718524 |
| COc1cc(C[C@H]2C(=O)OC[C@@H]2Cc2ccc(OC)c(OC)c2)ccc1O | 0.578139866 |
| COc1ccc(-c2oc3cc(O)cc(O)c3c(=O)c2O)cc1 | 0.641319096 |
| COc1ccc2cc1Oc1ccc(cc1)C[C@H]1c3c(cc4c(c3Oc3cc5c(cc3OC)CCN(C)[C@@H]5C2)OCO4)CCN1C | 0.405627868 |
| C=C(C)C(=O)O[C@H]1CC2=C[C@H](C/C(C)=C\[C@H]3OC(=O)C(=C)[C@H]13)OC2=O | 0.551017798 |
| O=C1/C(=C/c2ccc(O)c(O)c2)Oc2cc(O)ccc21 | 0.652825364 |
| COC(=O)C1=CO[C@@H](O)[C@@H]2C(CO)=CC[C@H]12 | 0.686473217 |
| N[C@@H](C=O)[C@@H](O)[C@H](O)[C@H](O)CO | 0.629931379 |
| CCCCCCCCCCCC1=C(O)C(=O)C=C(O)C1=O | 0.47217146 |
| O=C(/C=C/c1ccc(O)c(O)c1)c1ccc(O)cc1O | 0.671589624 |
| COc1c(O)cc(O)c2c(=O)cc(-c3ccccc3)oc12 | 0.584211595 |
| CC1=C[C@@H]2C(=O)[C@]3(C=C(C)[C@H](O)[C@@]3(O)[C@@H]1O)[C@H](C)C[C@@H]1[C@H]2C1(C)C | 0.697756972 |
| CCCCC1OC(=O)C2=CCCCC21 | 0.596602285 |
| Nc1ncnc2c1ncn2C1OC(CO)C(O)C1O | 0.671111893 |
| COc1cc2c(c(OC)c1OC)-c1c(cc3c(c1OC)OCO3)CC(C)C(C)(O)C2 | 0.512255893 |
| C=C(CC[C@@H](C(=O)O)[C@H]1[C@H](O)C[C@@]2(C)C3=CC[C@H]4C(C)(C)[C@@H](OC(C)=O)CC[C@]4(C)C3=CC[C@]12C)C(C)C | 0.715309178 |
| CC(=O)[C@H]1CC[C@H]2[C@@H]3CC=C4C[C@@H](O)CC[C@]4(C)[C@H]3CC[C@]12C | 0.679733063 |
| COc1ccc2c(c[n+](C)c3c4cc5c(cc4ccc23)OCO5)c1OC | 0.499346995 |
| COc1cc(/C=C/c2ccc(O)c(O)c2)cc(OC)c1 | 0.677797526 |
| Cn1nnc2c(C(N)=O)ncn2c1=O | 0.61237405 |
| CCOC(=O)c1ccc(O)c(O)c1 | 0.560292525 |
| COc1ccc2[nH]cc(CCNC(C)=O)c2c1 | 0.636909357 |
| C=CC(C)(C)c1cc(/C=C/C(=O)c2ccc(O)cc2)c(OC)cc1O | 0.621204764 |
| COc1cc(CNC(=O)CCCC/C=C/C(C)C)ccc1O | 0.585378176 |
| C[n+]1cc2c3c(ccc2c2ccc4cc5c(cc4c21)OCO5)OCO3 | 0.469116004 |
| COc1ccc(/C=C/c2cc(OC)cc(=O)o2)cc1 | 0.676500877 |
| COC1=C[C@@H]2[C@@H]3Cc4ccc(OC)c(O)c4[C@]2(CCN3C)CC1=O.Cl | 0.621964621 |
| CC1(C)CC[C@]2(C(=O)O)CC[C@]3(C)C(=CC[C@@H]4[C@@]5(C)CC[C@H](O)C(C)(C)C5CC[C@]43C)[C@H]2C1 | 0.873847271 |
| C/C=C(/C)C(=O)O[C@H]1[C@@H]2[C@H](C)C(=O)O[C@@H]2C[C@@H](C)[C@@H]2C=CC(=O)[C@]21C | 0.593550555 |
| C=C1C(=O)[C@@]23[C@H](O)C[C@@H]4C(C)(C)C(=O)CC[C@@]4(C)[C@@H]2CC[C@@H]1[C@H]3OC(C)=O | 0.68473633 |
| CC(=O)c1c(O)c(C)c(O)c2c1OC1=CC(=O)C(C(C)=O)C(=O)C12C | 0.524832507 |
| COc1cc(C(C)=O)ccc1O | 0.50475847 |
| O=C(O)[C@H]1O[C@@H](Oc2cc3oc(-c4ccccc4)cc(=O)c3c(O)c2O)[C@H](O)[C@@H](O)[C@@H]1O | 0.555204521 |
| OC[C@H]1O[C@@H](Cc2cc(O)cc(/C=C/c3ccc(O)cc3)c2)[C@H](O)[C@@H](O)[C@@H]1O | 0.603704117 |
| O=c1c(O)c(-c2ccccc2)oc2cc(O)cc(O)c12 | 0.619748875 |
| C[C@H]1[C@H](C)CC[C@]2(C(=O)O)CC[C@]3(C)C(=CC[C@@H]4[C@@]5(C)C[C@@H](O)[C@H](O)C(C)(C)[C@@H]5CC[C@]43C)[C@H]12 | 0.884498144 |
| CCCCC/C=C/C(=O)CCc1ccc(O)c(OC)c1 | 0.666160197 |
| CC(=O)[C@H]1[C@H]2O[C@]23[C@@H]2CC[C@@H]4C[C@@H](O)CC[C@]4(C)[C@H]2CC[C@]3(C)[C@H]1c1ccc(=O)oc1 | 0.68526724 |
| CC1CCC2N(C1)CC1C3CC4C(CC(=O)C5CC(O)CCC54C)C3CCC1C2(C)O | 0.783051254 |
| C[C@@H]1O[C@@H](Oc2c(-c3ccc(O)c(O)c3)oc3cc(O)cc(O)c3c2=O)[C@H](O)[C@H](O)[C@H]1O | 0.499780774 |
| COc1cc(/C=C/C(=O)CC(=O)/C=C/c2ccc(O)c(OC)c2)ccc1O | 0.560952955 |
| C=C[C@@]1(C)CC(=O)[C@]2(O)[C@]3(C)[C@H]([C@H](O)[C@H](OC(C)=O)[C@@]2(C)O1)C(C)(C)CC[C@@H]3O | 0.621267301 |
| C[C@H](CC(=O)C[C@@H](C)[C@H]1C[C@H](O)[C@@]2(C)C3=C(C(=O)C[C@]12C)[C@@]1(C)CCC(=O)C(C)(C)C1C[C@@H]3O)C(=O)O | 0.739067285 |
| CC(O)(CC(=O)O)CC(=O)O | 0.560788629 |
| CC(Cc1ccc(O)c(O)c1)C(C)Cc1ccc(O)c(O)c1 | 0.474465076 |
| Cc1cc(O)c(C(=O)CCC(C)C)c(=O)o1 | 0.680024245 |
| C[C@H]1COC2=C1C(=O)C(=O)c1c2ccc2c1CCCC2(C)C | 0.506926496 |
| COc1cc(O)c2c(=O)cc(-c3ccc(OC)c(-c4c(O)cc(O)c5c(=O)cc(-c6ccc(O)cc6)oc45)c3)oc2c1 | 0.444963636 |
| NC[C@H](O)c1ccc(O)c(O)c1 | 0.506631225 |
| C[C@H](C[C@H](O)[C@@H](O)C(C)(C)O)C1=C2C[C@H](O)[C@H]3[C@@]4(C)CCC(=O)C(C)(C)[C@@H]4CC[C@]3(C)[C@@]2(C)CC1 | 0.891316996 |
| C=C1C(=O)O[C@@H]2C[C@@]3(C)CCCC(=C)[C@@H]3C[C@H]12 | 0.634663408 |
| COc1cc2c3cc1Oc1c(OC)c(OC)cc4c1[C@@H](Cc1ccc(O)c(c1)Oc1ccc(cc1)C[C@@H]3N(C)CC2)N(C)CC4.Cl.Cl | 0.427390506 |
| Cl.NCC(=O)CCC(=O)O | 0.618427251 |
| O=c1ccc2ccc(O)c(O)c2o1 | 0.470450208 |
| COc1cccc2c1C(=O)c1c(O)c3c(c(O)c1C2=O)C[C@@](O)(C(=O)CO)C[C@@H]3O[C@H]1C[C@H](N)[C@H](O)[C@H](C)O1.Cl | 0.447244406 |
| Oc1ccc2c(c1)OC[C@]1(O)Cc3cc(O)c(O)cc3[C@H]21 | 0.556149501 |
| O=C1c2cccc(O)c2C(=O)c2c(O)cccc21 | 0.563313971 |
| C[C@H]1[C@@H](O)O[C@@H]2O[C@@]3(C)CC[C@H]4[C@H](C)CC[C@@H]1[C@@]24OO3 | 0.641559814 |
| COC(=O)[C@H]1[C@H]2C[C@@H]3c4[nH]c5cc(OC)ccc5c4CCN3C[C@H]2C[C@@H](OC(=O)c2cc(OC)c(OC)c(OC)c2)[C@@H]1OC | 0.432413012 |
| COc1ccc(/C=C/C(=O)c2ccc(OC)cc2)cc1 | 0.703934244 |
| CC1CCC2(NC1)OC1CC3C4CCC5CC(O)CCC5(C)C4CCC3(C)C1C2C | 0.862724466 |
| O=c1c(-c2ccc(O)cc2)coc2cc(O)cc(O)c12 | 0.624819473 |
| O=C(/C=C/c1ccc(O)cc1)CC(=O)/C=C/c1ccc(O)cc1 | 0.584324439 |
| O=c1cc(-c2ccc(O)cc2)oc2cc(O)c(O)c(O)c12 | 0.607956841 |
| COC(=O)/C=C/c1ccc(O)c(OC)c1 | 0.661233568 |
| Cc1cc(O)c2c(c1)C(=O)c1cc(O)cc(O)c1C2=O | 0.541425791 |
| C1=C2CCCN[C@H]2[C@@H]2C[C@H]1[C@H]1CCCCN1C2 | 0.573990059 |
| COc1ccc(-c2cc(=O)c3c(OC)c(OC)c(OC)c(OC)c3o2)cc1OC | 0.506186645 |
| O=C1C[C@@H](c2ccccc2)Oc2cc(O)cc(O)c21 | 0.603076666 |
| CC(=O)Oc1ccccc1C(=O)O | 0.555380023 |
| O=c1cc(-c2ccc(O)c(O)c2)oc2cc(O)cc(O)c12 | 0.617524347 |
| CC(=O)OC(C)(C)/C=C/C(=O)[C@](C)(O)[C@H]1[C@H](O)C[C@@]2(C)[C@@H]3CC=C4[C@@H](C=C(O)C(=O)C4(C)C)[C@]3(C)C(=O)C[C@]12C | 0.657765276 |
| COc1ccc([C@@H]2CC(=O)c3c(O)cc(O[C@@H]4O[C@H](CO[C@@H]5O[C@@H](C)[C@H](O)[C@@H](O)[C@H]5O)[C@@H](O)[C@H](O)[C@H]4O)cc3O2)cc1O | 0.453706764 |
| CC1CCC2(NC1)OC1CC3C4CCC5CC(O)CCC5(C)C4CCC3(C)C1C2C.Cl | 0.870469349 |
| CC(C)C12OC1C1OC13C1(C)CCC4=C(COC4=O)C1CC1OC13C2=O | 0.583000246 |
| COc1cc2c(c(OC)c1OC)-c1ccc(OC)c(=O)cc1C(NC(C)=O)CC2 | 0.532901509 |
| O=c1cc(-c2ccc(O)cc2)oc2c([C@@H]3OC[C@H](O)[C@H](O)[C@H]3O)c(O)c([C@@H]3O[C@H](CO)[C@@H](O)[C@H](O)[C@H]3O)c(O)c12 | 0.418865674 |
| CC(=O)OC(C)(C)C=CC(=O)C(C)(O)C1C(O)CC2(C)C3CC=C4C(CC(O)C(=O)C4(C)C)C3(C)C(=O)CC12C | 0.68439288 |
| COc1c(O)cc2oc(-c3ccccc3)cc(=O)c2c1O | 0.572520811 |
| C[C@@H]1O[C@@H](OC[C@H]2O[C@@H](Oc3c(-c4ccc(O)c(O)c4)oc4cc(O)cc(O)c4c3=O)[C@H](O)[C@@H](O)[C@@H]2O)[C@H](O)[C@H](O)[C@H]1O | 0.413134157 |
| COc1cc2c(c(OC)c1OC)-c1c(cc3c(c1OC)OCO3)C[C@@H](C)[C@@H](C)C2 | 0.510007464 |
| COc1ccc2cc1Oc1ccc(cc1)C[C@H]1c3cc(c(OC)cc3CCN1C)Oc1c(O)c(OC)cc3c1[C@@H](C2)N(C)CC3 | 0.392376592 |
| COc1ccc(-c2cc(=O)c3c(O)cc(O)cc3o2)cc1 | 0.610552542 |
| COc1ccc(-c2cc(=O)c3c(O)c(OC)c(O)cc3o2)cc1OC | 0.575533656 |
| COc1cc(/C=C/C(=O)N2CCC=CC2=O)cc(OC)c1OC | 0.565218373 |
| OCC(O)C(O)C(O)CO | 0.581867809 |
| COc1ccc(-c2oc3c(CC=C(C)C)c(O)cc(O)c3c(=O)c2O)cc1 | 0.600156201 |
| C=C(C)[C@@H]1CC[C@]2(C(=O)O)CC[C@]3(C)[C@H](CC[C@@H]4[C@@]5(C)CC[C@H](O)C(C)(C)[C@@H]5CC[C@]43C)[C@@H]12 | 0.902206477 |
| O=C(O[C@@H]1Cc2c(O)cc(O)cc2O[C@@H]1c1cc(O)c(O)c(O)c1)c1cc(O)c(O)c(O)c1 | 0.536369054 |
| CC1=C(O)C(=O)C=C2C1=CC=C1[C@@]2(C)CC[C@@]2(C)[C@@H]3C[C@](C)(C(=O)O)CC[C@]3(C)CC[C@]12C | 0.672152008 |
| Cn1c(=O)c2[nH]cnc2n(C)c1=O | 0.604648662 |
| O=C1OC(c2ccc(O)cc2)Cc2cccc(O)c21 | 0.660445272 |
| C=C1C(=O)O[C@H]2C[C@@H](C)[C@@H]3C=CC(=O)[C@@]3(C)[C@@H](OC(C)=O)[C@H]12 | 0.52500786 |
| CC1(C)C[C@@H](O)[C@]2(C)CC[C@]3(C)C(=CC[C@@H]4[C@@]5(C)CC[C@H](O)[C@](C)(CO)[C@@H]5CC[C@]43C)[C@@H]2C1 | 0.894526531 |
| COc1c(CC=C(C)C)c(O)cc2oc(=O)c(-c3ccc(O)cc3O)cc12 | 0.6091012 |
| COc1cc2c(c(OC)c1OC)-c1c(cc(OC)c(OC)c1OC)CC(C)(O)C(C)C2 | 0.507255034 |
| Oc1cc(O)cc(/C=C/c2ccc(O)c(O)c2)c1 | 0.677420423 |
| O=c1c(O)c(-c2cc(O)c(O)c(O)c2)oc2cc(O)cc(O)c12 | 0.608719963 |
| C=CCc1ccc(O)c(-c2cc(CC=C)ccc2O)c1 | 0.579745369 |
| COc1cc2c(cc1OC)[C@@H](Cc1ccc(O)c(Oc3cc4c(cc3OC)CCN(C)[C@@H]4Cc3ccc(O)cc3)c1)N(C)CC2 | 0.390967443 |
| COc1ccc(-c2cc(=O)c3c(O)c(OC)c(OC)cc3o2)cc1 | 0.605055106 |
| CC(C)[C@]12CC(=O)[C@H](C)[C@H]1C2 | 0.564976893 |
| O=c1c(O)c(-c2ccc(O)cc2)oc2cc(O)cc(O)c12 | 0.643158385 |
| COC1=C[C@@H]2[C@@H]3Cc4ccc(OC)c(O)c4[C@]2(CCN3C)CC1=O | 0.578146274 |
| Oc1cc(O)c2c(c1)O[C@H](c1cc(O)c(O)c(O)c1)[C@H](O)C2 | 0.565078832 |
| COc1cc(CCC(=O)CC(=O)CCc2ccc(O)c(OC)c2)ccc1O | 0.527988394 |
| CC[C@@H]1CN2CC[C@@]3(C(=O)Nc4ccccc43)[C@@H]2C[C@@H]1/C(=C\OC)C(=O)OC | 0.521786019 |
| COc1cc(/C=C/c2cc(O)cc(O)c2)ccc1O | 0.635566429 |
| COc1cc2c(cc1OC)[C@@H]1C(=O)c3ccc4c(c3O[C@@H]1CO2)C=CC(C)(C)O4 | 0.571349715 |
| O=C(O[C@@H]1Cc2c(O)cc(O)cc2O[C@@H]1c1ccc(O)c(O)c1)c1cc(O)c(O)c(O)c1 | 0.580164834 |
| CC1=C2[C@@H](OC1=O)[C@H](O)C13[C@H]4C[C@@H](C(C)(C)C)C15[C@@H](OC(=O)[C@@H]5O)O[C@@]23C(=O)O4 | 0.594675101 |
| CC(C)(O)CCC(O)C(C)(O)C1CCC2(O)C3=CC(=O)C4CC(O)C(O)CC4(C)C3CCC12C | 0.767618309 |
| COc1ccc(-c2coc3cc(O)cc(O)c3c2=O)cc1 | 0.624849273 |
| COc1cc([C@H]2Oc3cc([C@H]4Oc5cc(O)cc(O)c5C(=O)[C@@H]4O)ccc3O[C@@H]2CO)ccc1O | 0.527343346 |
| O=c1cc(-c2ccccc2)oc2cc(OC3OC(COC4OC(CO)C(O)C(O)C4O)C(O)C(O)C3O)c(O)c(O)c12 | 0.43506949 |
| COc1ccc(C[C@@H]2c3cc(Oc4cc(C[C@H]5c6cc(OC)c(OC)cc6CCN5C)ccc4O)c(OC)cc3CCN2C)cc1 | 0.387829335 |
| CC(C)(C)[C@]1(O)C[C@@H]2OC(=O)C[C@@]23C(=O)O[C@@H]2OC(=O)[C@H](O)[C@]213 | 0.583435917 |
| C=C1C(=O)O[C@H]2C[C@@H](C)[C@@H]3[C@@H](OC(C)=O)C[C@H](O)[C@@]3(C)[C@@H](OC(C)=O)[C@H]12 | 0.582265361 |
| COc1cc2c(cc1OC)-c1c(C)c3ccc(OC)c(OC)c3c[n+]1CC2 | 0.477380367 |
| COc1cc(/C=C/c2ccc(O)cc2)cc(OC)c1 | 0.647769269 |
| C=CCc1ccc(O)c(-c2ccc(O)c(CC=C)c2)c1 | 0.572804546 |
| C[C@H]1[C@H](C)CC[C@]2(C(=O)O)CC[C@]3(C)C(=CC[C@@H]4[C@@]5(C)CC[C@H](O)C(C)(C)C5CC[C@]43C)[C@H]12 | 0.886060644 |
| COc1c2ccoc2cc2oc(=O)ccc12 | 0.557013905 |
| Oc1cc(O)cc(/C=C/c2ccccc2)c1 | 0.604183467 |
| C=C[C@@](C)(/C=C/c1ccc(O)cc1)CCC=C(C)C | 0.633253308 |
| O=C(/C=C/c1ccc(O)cc1)c1ccc(O)cc1O | 0.679748991 |
| C[C@H]1OC(Oc2c(-c3ccc(O)cc3)oc3cc(O)cc(O)c3c2=O)[C@@H](O)[C@@H](O)[C@@H]1O | 0.569228068 |
| C=C1C(=O)[C@]23C[C@H]1C[C@@H](O)[C@H]2[C@@]12CO[C@@H](O)[C@@H]1C(C)(C)CC[C@@H]2OC3=O | 0.643638886 |
| C=C1CC[C@@H]2[C@](C)(CO)[C@H](O)CC[C@@]2(C)[C@@H]1C/C=C1/C(=O)OC[C@H]1O | 0.65658649 |
| O=C1CCCC2C3CCCN4CCCC(CN12)C34 | 0.642430102 |
| CC1OC(OC2CC(O)C3(CO)C4C(O)CC5(C)C(C6=CC(=O)OC6)CCC5(O)C4CCC3(O)C2)C(O)C(O)C1O.O.O.O.O.O.O.O.O | 0.71441363 |
| COc1cc2c(cc1O)C[C@H]1c3c(cc(O)c(OC)c3-2)CCN1C | 0.495435881 |
| C[C@H](C[C@H](O)[C@H]1OC1(C)C)C1=C2C[C@H](O)[C@H]3[C@@]4(C)CCC(=O)C(C)(C)[C@@H]4CC[C@]3(C)[C@@]2(C)CC1 | 0.907205716 |
| C[C@@]1(C(=O)O)CC[C@]2(C)CC[C@]3(C)C4=CC(=O)c5c(cc(O)c(O)c5C=O)[C@]4(C)CC[C@@]3(C)[C@@H]2C1 | 0.599930666 |
| C[C@@H]1CC[C@@]2(OC1)O[C@H]1C[C@H]3[C@@H]4CC[C@H]5C[C@@H](O)[C@H](O)C[C@]5(C)[C@H]4CC[C@]3(C)[C@H]1[C@@H]2C | 0.798278027 |
| CC(C)=CCC/C(C)=C/Cc1cc(-c2coc3cc(O)ccc3c2=O)ccc1O | 0.574704908 |
| COc1cc(-c2cc(=O)c3c(O)c(OC)c(O)cc3o2)ccc1O | 0.622905701 |
| CC1=C2C[C@H]3[C@@H](CC=C4C[C@@H](O)CC[C@@]43C)[C@@H]2CC2O[C@@H]3C[C@H](C)CN[C@H]3[C@@H](C)[C@@H]12 | 0.760402171 |
| CC1=C2C(=O)[C@H]3[C@@H](CC=C4C[C@@H](O)CC[C@@]43C)[C@@H]2CC[C@]12O[C@@H]1C[C@H](C)CN[C@H]1[C@H]2C | 0.776685615 |
| C[C@@H]1O[C@@H](Oc2ccc(CN=C=S)cc2)[C@H](O)[C@H](O)[C@H]1O | 0.770774603 |
| COc1cc([C@H]2OC[C@H]3[C@@H]2CO[C@@H]3c2ccc(O)c(OC)c2)ccc1O | 0.536178633 |
| COc1cc(O)c(CC=C(C)C)c(O)c1C(=O)C=Cc1ccc(O)cc1 | 0.611214655 |
| CC(=O)c1c(O)c(C)c(O)c2c1OC1=CC(=O)C(C(C)=O)C(=O)[C@@]12C | 0.524832507 |
| CC1=C2[C@H]3O[C@]34CC[C@@H]3C(C)(C)CCC[C@@]3(C)[C@@H]4[C@H]3O[C@@]23OC1=O | 0.695407256 |
| OCC1OC(OCCc2ccc(O)cc2)C(O)C(O)C1O | 0.679543709 |
| C[C@@H]1CC[C@]2(C(=O)O)CC[C@]3(C)C(=CC[C@@H]4[C@@]5(C)CC[C@H](O)[C@@](C)(CO)[C@@H]5CC[C@]43C)[C@@H]2[C@]1(C)O | 0.871139051 |
| CC1CCC2(OC1)OC1CC3C4CCC5CC(OC6OC(CO)C(O)C(O)C6OC6OC(CO)C(O)C(O)C6O)CCC5(C)C4CCC3(C)C1C2C | 0.708170232 |
| C=C1CC[C@@]2(OC1)O[C@H]1C[C@H]3[C@@H]4CC=C5C[C@@H](O)C[C@@H](O)[C@]5(C)[C@H]4CC[C@]3(C)[C@H]1[C@@H]2C | 0.859713625 |
| COc1ccc(-c2cc(=O)c3c(O)cc(O[C@@H]4O[C@H](CO[C@@H]5O[C@@H](C)[C@H](O)[C@@H](O)[C@H]5O)[C@@H](O)[C@H](O)[C@H]4O)cc3o2)cc1O | 0.428663599 |
| O=c1[nH]c(=O)c2nc[nH]c2[nH]1 | 0.608214951 |
| COc1cc2oc(-c3ccc(O[C@@H]4O[C@H](CO)[C@@H](O)[C@H](O)[C@H]4O)cc3)cc(=O)c2c(O)c1OC | 0.510597577 |
| Cn1c(=O)c2[nH]cnc2n(C)c1=O.Cn1c(=O)c2[nH]cnc2n(C)c1=O.NCCN | 0.586104021 |
| Cn1c(=O)c2c(ncn2CC(O)CO)n(C)c1=O | 0.688722618 |
| COc1cc2oc(-c3ccc(O)cc3)cc(=O)c2c(O)c1[C@@H]1O[C@H](CO)[C@@H](O)[C@H](O)[C@H]1O | 0.558075198 |
| Cn1c(=O)c2c(ncn2CC(=O)O)n(C)c1=O | 0.681677369 |
| COc1cc2c(cc1O)C[C@@H]1NCCc3cc(OC)c(O)c-2c31 | 0.507686849 |
| C=C(CC[C@@H](C)[C@H]1CC[C@H]2C3=C(C(=O)C[C@]12C)[C@@]1(C)CCC(=O)[C@@H](C)[C@@H]1CC3)C(C)C(=O)O | 0.710847946 |
| Oc1cc(O)c2c(c1)O[C@H](c1ccc(O)c(O)c1)[C@H](O)[C@H]2c1c(O)cc(O)c2c1O[C@H](c1ccc(O)c(O)c1)[C@H](O)C2 | 0.438105926 |
| C[C@]12C(=O)C=CCC1=CC[C@@H]1[C@@H]2CC[C@]2(O)C(=O)O[C@@]3(C)[C@H]4C[C@]5(C)[C@@H](CO[C@@]16O[C@@]32[C@H]5C6=O)C(=O)O4 | 0.6611594 |
| O=c1cc(-c2ccc(O)cc2)oc2cc(O)ccc12 | 0.621674408 |
| CC(C)=CCOc1c2occc2cc2ccc(=O)oc12 | 0.646183325 |
| COc1cc2c(cc1OC)-c1cc3ccc(OC)c(OC)c3c[n+]1CC2 | 0.483603865 |
| CC1=C2C(=O)O[C@@H](c3ccoc3)[C@]2(C)CCC1 | 0.617266097 |
| O=C1NCCc2c1[nH]c1ccccc21 | 0.518909987 |
| COc1cc2oc(=O)ccc2cc1O[C@@H]1O[C@H](CO)[C@@H](O)[C@H](O)[C@H]1O | 0.674910944 |
| C=CCS[S+]([O-])CC=C | 0.630638833 |
| COc1cc2c(cc1OC)[C@@H]1Cc3ccc(OC)c(OC)c3C[N@@+]1([O-])CC2 | 0.512984165 |
| CC1(C)CCC2(C)CCC3(C)C(=CCC4C5(C)CCC(=O)C(C)(C)C5CCC43C)C2C1 | 0.803299628 |
| COc1ccc([C@H]2O[C@@H](c3ccc(OC)c(OC)c3)[C@@H](C)[C@@H]2C)cc1OC | 0.501103329 |
| CC1(C)CCC2=C(O1)C(=O)c1ccccc1C2=O | 0.545632341 |
| O=C[C@@](O)(COC(=O)c1cc(O)c(O)c(O)c1)[C@H](O)[C@H](O)COC(=O)c1cc(O)c(O)c(O)c1 | 0.524070245 |
| CC(C)c1ccc2c(c1)CC[C@H]1[C@](C)(CO)CCC[C@]21C | 0.550841602 |
| CCCCCC1=CC(=O)C=C(OC)C1=O | 0.601290047 |
| COC(=O)C=Cc1ccc(O)c(OC)c1 | 0.661233568 |
| COc1cc(/C=C/C(=O)O[C@H]2C[C@@](O)(C(=O)O)C[C@H](O)[C@@H]2O)ccc1O | 0.621203801 |
| CC(C)=CCc1c(O)cc2oc3cc(O)c(O)c(CCC(C)(C)O)c3c(=O)c2c1O | 0.554678932 |
| CC(C)c1ccc2c(c1)CC[C@H]1[C@](C)(C(=O)O)CCC[C@]21C | 0.617489337 |
| O=C1c2ccccc2C(=O)c2c(O)cc(O)cc21 | 0.554401238 |
| COc1cc(O[C@@H]2O[C@H](CO)[C@@H](O)[C@H](O)[C@H]2O)c2c(O)c(C(C)=O)c(C)cc2c1 | 0.684838175 |
| CC(C)c1c(O)ccc2c1CC[C@H]1C(C)(C)CCC[C@]21C | 0.620359175 |
| C=C1C(=O)O[C@@H]2C[C@]3(C)[C@@H](CCC(C)=O)[C@@H]3C[C@H]12 | 0.613768503 |
| COc1cc(C[C@@H]2COC(=O)[C@@]2(O)Cc2ccc(O)c(OC)c2)ccc1O | 0.638285037 |
| COc1cc2ccc(=O)oc2c(OC)c1O[C@@H]1O[C@H](CO)[C@@H](O)[C@H](O)[C@H]1O | 0.657816876 |
| C=C(C)[C@H](CC=C(C)C)Cc1c(O)cc(OC)c2c1O[C@H](c1ccc(O)cc1OC)CC2=O | 0.515063928 |
| COc1ccc(-c2oc3cc(OC)cc(O)c3c(=O)c2O)cc1 | 0.62107979 |
| O=C1c2c(O)cc(O)cc2O[C@H](c2ccc(O)cc2)[C@@H]1[C@@H]1C(=O)c2c(O)cc(O)cc2O[C@H]1c1ccc(O)cc1 | 0.397944393 |
| CC1(C)CC[C@]2(C(=O)O)CC[C@]3(C)C(=CC[C@@H]4[C@@]5(C)C[C@@H](O)[C@H](O)[C@@](C)(CO)[C@@H]5CC[C@]43C)[C@@H]2[C@@H]1O | 0.859294756 |
| O=C(OCc1ccccc1)c1ccccc1 | 0.701733891 |
| CC1=CC(=O)[C@@H](O)[C@]2(C)[C@H]3[C@@]4(O)OC[C@@]35[C@@H](C[C@@H]12)OC(=O)[C@H](O)[C@@]5(O)[C@@H](C)[C@H]4O | 0.613503548 |
| COc1cc(CCN(C)C)c2ccc3ccccc3c2c1OC | 0.491862991 |
| C=C[C@H]1C[N@@]2CC[C@H]1C[C@H]2[C@H](O)c1ccnc2ccccc12 | 0.514125989 |
| COc1cc2c[n+](C)c3c4cc5c(cc4ccc3c2cc1OC)OCO5.[Cl-] | 0.493131876 |
| Cn1c(=O)c2c3c(ccc2c2ccc4cc5c(cc4c21)OCO5)OCO3 | 0.529346942 |
| COc1cc2oc3cc(O)c(OC)c(CC=C(C)C)c3c(=O)c2c(O)c1CC=C(C)C | 0.583386475 |
| O=C(CCc1ccc(O)c(O)c1)NCCCNCCCCNCCCNC(=O)CCc1ccc(O)c(O)c1 | 0.364744143 |
| C[C@]1(O)C[C@@H](O)[C@]2(O)C=CO[C@@H](O[C@@H]3O[C@H](CO)[C@@H](O)[C@H](O)[C@H]3O)C12 | 0.654405075 |
| COc1ccc([C@H]2OC[C@H]3[C@@H]2CO[C@H]3c2cc(OC)c(OC)c(OC)c2)cc1OC | 0.490526383 |
| COc1cc(/C=C/C(=O)c2ccc(O)cc2O)ccc1O | 0.675403551 |
| CC1=CC(=O)[C@@H](O)[C@]2(C)C3[C@@H](O)[C@H](O)[C@@]4(C)OC[C@@]35[C@@H](C[C@@H]12)OC(=O)[C@H](O)[C@]54O | 0.564387129 |
| CCCCCCCCCCCCCC1=C(O)C(=O)C=C(O)C1=O | 0.430022146 |
| CC(C)=CCc1cc(-c2cc(=O)c3cc(CC=C(C)C)c(O)cc3o2)ccc1O | 0.529004061 |
| O=C(/C=C/c1ccc(O)cc1)NCCc1ccc(O)cc1 | 0.698610696 |
| C[C@H]1[C@H](OC(=O)CCC(=O)O)O[C@@H]2O[C@@]3(C)CC[C@H]4[C@H](C)CC[C@@H]1[C@@]24OO3 | 0.652780972 |
| O=c1[nH]c2ccccc2o1 | 0.573468584 |
| Cl.Cl.O=C(C[C@H]1NCCC[C@@H]1O)Cn1cnc2ccccc2c1=O | 0.623160889 |
| CC1CCC(C(C)C)C(=O)C1 | 0.607719218 |
| CC(C)C12C=CC(C)(CC1)OO2 | 0.555574566 |
| C=C[C@H]1CN2CC[C@H]1C[C@@H]2[C@@H](O)c1ccnc2ccc(OC)cc12 | 0.537169007 |
| Oc1ccc2c(c1)OC[C@@H]1c3cc4c(cc3O[C@H]21)OCO4 | 0.496521511 |
| COC(=O)[C@@]12OC[C@]34[C@H]([C@@H](O)[C@@H]1O)[C@@]1(C)CC(=O)C(O)=C(C)[C@@H]1C[C@H]3OC(=O)[C@H](OC(=O)CC(C)C)[C@@H]24 | 0.59482796 |
| CC1=C2C3OC(=O)C(C)C3CCC2(C)C=CC1=O | 0.564223236 |
| C[C@H]1[C@H](C)CC[C@]2(C(=O)O)CC[C@]3(C)C(=CC[C@@H]4[C@@]5(C)C[C@@H](O)[C@H](O)[C@@](C)(CO)[C@@H]5CC[C@]43C)[C@H]12 | 0.884377652 |
| CC(C)=CCc1c(O)c(CC=C(C)C)c2oc3c(O)cccc3c(=O)c2c1O | 0.550986843 |
| C=C[C@H]1C[N@@]2CC[C@H]1C[C@@H]2[C@@H](O)c1ccnc2ccccc12 | 0.514125989 |
| C=CCc1ccc(O)c(OC)c1 | 0.542498552 |
| C=C[C@H]1CN2CC[C@H]1C[C@H]2[C@H](O)c1ccnc2ccc(OC)cc12 | 0.537169007 |
| C[C@H]1[C@H](O)O[C@@H]2O[C@@]3(C)CC[C@H]4[C@H](C)CC[C@@H]1[C@@]24OO3 | 0.641559814 |
| CC1(C)C=Cc2c(-c3c4c(c5ccccc5c3O)OC(C)(C)C=C4)c(O)c3ccccc3c2O1 | 0.389355968 |
| O=c1c2ccccc2ncn1C[C@]1(O)C[C@@H]2NCCC[C@@H]2O1 | 0.508109776 |
| COc1ccc(/C=C/C(=O)c2ccccc2O)cc1OC | 0.688236343 |
| O=c1cc(CO)occ1O | 0.60133675 |
| C[C@@]12C[C@@H](c3ccoc3)OC(=O)[C@@H]1CC[C@]1(C)[C@H]2[C@H]2C=C[C@]1(O)C(=O)O2 | 0.622166623 |
| CC1=C[C@H]2[C@@H](CC1)[C@H](C)CC[C@H]2[C@@H](C)C(=O)O | 0.691018209 |
| C=C(C)[C@@H]1CC[C@]2(C)CC[C@]3(C)[C@H](CC[C@@H]4[C@@]5(C)CCC(=O)C(C)(C)[C@@H]5CC[C@]43C)[C@@H]12 | 0.850970245 |
| COc1cc2c(cc1O)[C@@H]1Cc3ccc(OC)c(O)c3CN1CC2 | 0.477750472 |
| C[C@@H]1CC[C@H]2[C@@H](C)C(=O)O[C@@H]3O[C@]4(C)CC[C@@H]1[C@]32OO4 | 0.654259108 |
| COC1=CC(=O)C=C(OC)C1=O | 0.53411145 |
| CCO[C@H]1O[C@@H]2O[C@@]3(C)CC[C@H]4[C@H](C)CC[C@@H]([C@H]1C)[C@@]24OO3 | 0.636197501 |
| CC[C@@H]1[C@H]2CCCCN3[C@H]2[C@H](C[C@H]3[C@@H]2C[C@H](C)C(=O)O2)[C@@H]2[C@H]1OC(=O)[C@H]2C | 0.663473264 |
| COc1c2ccoc2c(OC)c2oc(=O)ccc12 | 0.612914277 |
| CC(=O)c1c(C)cc2cccc(O)c2c1O | 0.597320864 |
| CCC/C=C1\OC(=O)c2ccccc21 | 0.531742431 |
| CO[C@H]1O[C@@H]2O[C@@]3(C)CC[C@H]4[C@H](C)CC[C@@H]([C@H]1C)[C@@]24OO3 | 0.637109994 |
| C/C(=C\CO)CCC[C@H](C)CCC[C@H](C)CCCC(C)C | 0.57218583 |
| C[C@]1(O)C[C@@H](O)[C@@H]2C=CO[C@@H](O[C@@H]3O[C@H](CO)[C@@H](O)[C@H](O)[C@H]3O)[C@@H]21 | 0.661495631 |
| CCCCCCCCCCCCCCCC(=O)OC | 0.556865495 |
| CC(C)=CCc1c(-c2ccc(O)cc2O)oc2c([C@H]3C=C(C)C[C@@H](c4ccc(O)cc4O)[C@@H]3C(=O)c3ccc(O)cc3O)c(O)cc(O)c2c1=O | 0.371573915 |
| O=c1cc(-c2ccc(O)cc2)oc2cc(O)c([C@@H]3O[C@H](CO)[C@@H](O)[C@H](O)[C@H]3O)c(O)c12 | 0.564480925 |
| CCC/C=C1\OC(=O)c2ccccc21 | 0.531742431 |
| CCOC(=O)[C@@H](Cc1ccc(O)c(O)c1)OC(=O)/C=C/c1ccc(O)c(O)c1 | 0.558886655 |
| CC1OC(OC2C(OCCc3ccc(O)c(O)c3)OC(COC3OC(CO)C(O)C(O)C3O)C(O)C2OC(=O)C=Cc2ccc(O)c(O)c2)C(O)C(O)C1O | 0.412458989 |
| OC[C@H]1NC[C@H](O)[C@@H](O)[C@@H]1O | 0.608000002 |
| O=c1cc(-c2ccc(O)cc2)oc2cccc(O)c12 | 0.603843598 |
| COc1c(O)ccc(O)c1-c1cc(=O)c2c(O)cc(O)c(OC)c2o1 | 0.624351315 |
| CCCCCCCCCCCCCc1cccc(O)c1C(=O)O | 0.527979774 |
| O=C1CC(C(=O)O)c2c1oc(=O)c1cc(O)c(O)c(O)c21 | 0.621485534 |
| COc1cc(O)cc(CCc2ccc(OC)c(O)c2)c1 | 0.582204551 |
| COc1cc(C2OCC3C(c4ccc(OC5OC(CO)C(O)C(O)C5O)c(OC)c4)OCC23)ccc1OC1OC(CO)C(O)C(O)C1O | 0.408789491 |
| COc1ccc(CCNC(=O)/C=C/c2ccc(O)c(O)c2)cc1 | 0.689201682 |
| COc1c(/C=C/C(=O)c2ccc(O)cc2)ccc(O)c1CC=C(C)C | 0.592882893 |
| O=c1c(O[C@@H]2O[C@H](CO[C@@H]3O[C@H](CO)[C@@H](O)[C@H](O)[C@H]3O)[C@@H](O)[C@H](O)[C@H]2O)c(-c2ccc(O)cc2)oc2cc(O)cc(O)c12 | 0.437190546 |
| CC[C@H](CC[C@@H](C)[C@H]1CC[C@H]2[C@@H]3CC=C4C[C@@H](O[C@@H]5O[C@H](CO)[C@@H](O)[C@H](O)[C@H]5O)CC[C@]4(C)[C@H]3CC[C@]12C)C(C)C | 0.869211769 |
| COc1ccc(/C=C/C(=O)O)cc1 | 0.575784522 |
| O=c1c(O[C@@H]2O[C@H](CO)[C@@H](O)[C@H](O)[C@H]2O)c(-c2ccc(O)cc2)oc2cc(O[C@@H]3O[C@H](CO)[C@@H](O)[C@H](O)[C@H]3O)cc(O)c12 | 0.444411982 |
| O=C(C=Cc1ccc(O)c(O)c1)OC(Cc1ccc(O)c(O)c1)C(=O)O | 0.633861246 |
| NCCc1c[nH]c2ccc(O)cc12 | 0.508983903 |
| CCC(=O)c1c(O)cc(O)cc1O | 0.538069505 |
| COc1ccc(/C=C/C=O)cc1 | 0.588531926 |
| C[C@@H]1O[C@@H](O[C@H]2[C@H](Oc3cc(O)c4c(c3)O[C@H](c3ccc(O)c(O)c3)CC4=O)O[C@H](CO)[C@@H](O)[C@@H]2O)[C@H](O)[C@H](O)[C@H]1O | 0.450474816 |
| CN1Cc2c(ccc3c2OCO3)[C@@]2(C)[C@@H](O)Cc3cc4c(cc3[C@@H]12)OCO4 | 0.531540958 |
| CC1(C)CCC2(C(=O)O)CCC3(C)C(=CCC4C5(C)CCC(OC6OC(C(=O)OC7OC(CO)C(O)C(O)C7O)C(OC7OC(CO)C(O)C7O)C(O)C6O)C(C)(C)C5CCC43C)C2C1 | 0.745717293 |
| COc1cc2c(cc1OC)C1C(C)c3ccc(OC)c(OC)c3CN1CC2 | 0.491137985 |
| C[C@@H]1O[C@@H](Oc2cc(O)c3c(=O)c(O)c(-c4ccc(O)cc4)oc3c2O)[C@H](O)[C@H](O[C@@H]2O[C@H](CO)[C@@H](O)[C@H](O)[C@H]2O)[C@H]1O | 0.426953053 |
| CC(=O)O[C@H](CC=C(C)C)C1=CC(=O)c2c(O)ccc(O)c2C1=O | 0.519427619 |
| COc1cc(O)c(C(=O)c2ccccc2)c(O)c1 | 0.637658455 |
| [Cl-].c1c2c(cc3c1OCO3)-c1cc3cc4c(cc3c[n+]1CC2)OCO4 | 0.49542515 |
| COc1ccc2c3c1O[C@H]1C[C@@H](O)C=C[C@@]31CCN(C)C2 | 0.522209532 |
| COc1cc2ccc(=O)oc2cc1O | 0.546506261 |
| C[C@@H]1O[C@@H](c2c(O)c([C@@H]3O[C@H](CO)[C@@H](O)[C@H](O)[C@H]3O)c(O)c3c(=O)cc(-c4ccc(O)cc4)oc23)[C@H](O)[C@H](O)[C@H]1O | 0.431666361 |
| CC1(C)CCC2(C(=O)O)CCC3(CO)C(=CCC4C5(C)CC(O)C(OC6OC(CO)C(O)C(O)C6O)C(C)(C(=O)O)C5CCC43C)C2C1 | 0.643428038 |
| CC(C)=CCOc1c2ccoc2cc2oc(=O)ccc12 | 0.672036453 |
| CC(=O)O[C@H]1CC[C@@]2(C)C(CC[C@]3(C)[C@@H]2CC=C2[C@@H]4[C@@H](C)[C@H](C)CC[C@]4(C(=O)O)CC[C@]23C)C1(C)C | 0.785746153 |
| C[C@@H]1O[C@@H](Oc2cc(O)c3c(=O)c(O)c(-c4ccc(O)cc4)oc3c2O)[C@H](O)[C@H](O)[C@H]1O | 0.528817176 |
| COc1ccc2ccc(=O)oc2c1CC(=O)C(C)C | 0.71004482 |
| COc1cc2c(cc1O)CC[n+]1cc3c(OC)c(OC)ccc3cc1-2 | 0.483420336 |
| COc1ccc(-c2cc(=O)c3c(O)cc(OC4OC(COC5OC(C)C(O)C(O)C5O)C(O)C(O)C4O)cc3o2)cc1 | 0.440667269 |
| CN1C2=C3N=c4ccccc4=C3CCN2C(=O)c2ccccc21.Cl | 0.591901267 |
| COc1cc(O)c2c(=O)c3c(O[C@@H]4O[C@H](CO)[C@@H](O)[C@H](O)[C@H]4O)ccc(O)c3oc2c1 | 0.564959633 |
| CC1(C)CC[C@]2(C(=O)O)CC[C@]3(C)C(=CC[C@@H]4[C@@]5(C)C[C@@H](O)[C@H](O)[C@@](C)(CO)[C@@H]5CC[C@]43C)C2C1 | 0.880116289 |
| CC(C)[C@@H]1CC[C@@H](C)C[C@H]1O | 0.568555404 |
| CC1=C[C@H]2Cc3[nH]c(=O)ccc3[C@]3(C1)NCCC[C@H]23 | 0.596010213 |
| CC(C)(O[C@@H]1O[C@H](CO)[C@@H](O)[C@H](O)[C@H]1O)[C@H]1Cc2cc3ccc(=O)oc3cc2O1 | 0.681517904 |
| CCCc1cc(=O)c2ccccc2n1C | 0.58246204 |
| COc1cc2c3c(cc4ccccc4c3c1OC)N(C)CC2 | 0.51239152 |
| COc1cc(C=CC(=O)OCC[N+](C)(C)C)cc(OC)c1O | 0.684605308 |
| C/C=C(/C)C(=O)O[C@@H]1c2c(ccc3ccc(=O)oc23)OC(C)(C)[C@@H]1OC(C)=O | 0.572647236 |
| COc1cc2c(cc1OC)-c1cc3ccc4c(c3c[n+]1CC2)OCO4 | 0.478869374 |
| C/C=C1\C2C=C(C)CC1(N)c1ccc(=O)[nH]c1C2 | 0.580468098 |
| c1ccc2c(c1)CCO2 | 0.451249289 |
| COC(=O)C1=CO[C@@H](O[C@@H]2O[C@H](CO)[C@@H](O)[C@H](O)[C@H]2O)[C@@H]2[C@@H](C)[C@@H](O)C[C@H]12 | 0.692240959 |
| CCCCCCCCCCCCC(=O)OC | 0.650132209 |
| OC[C@@H]1CCCN2CCCC[C@H]12 | 0.579728217 |
| O=C(/C=C/c1ccc(O)c(O)c1)O[C@@H]1C[C@@](O)(C(=O)O)C[C@@H](O)[C@@H]1O | 0.61575665 |
| OC[C@H]1O[C@@H](Oc2cc(O)cc(/C=C/c3ccc(O[C@@H]4O[C@H](CO)[C@@H](O)[C@H](O)[C@H]4O)cc3O)c2)[C@H](O)[C@@H](O)[C@@H]1O | 0.476542224 |
| COc1cc2oc(-c3ccccc3)cc(=O)c2c(O)c1O | 0.599500522 |
| COc1cc(O)c2c(=O)cc(-c3ccc(OC)c(OC)c3)oc2c1 | 0.599535604 |
| COc1ccc(-c2oc3c4c(cc(O)c3c(=O)c2O)OC(C)(C)CC4)cc1 | 0.576337697 |
| CC(=O)Oc1cccc2c1C(=O)c1c(OC(C)=O)cc(C(=O)O)cc1C2=O | 0.504961184 |
| COc1ccc(-c2cc(=O)c3c(O)c(OC)c(O[C@@H]4O[C@H](CO[C@@H]5O[C@@H](C)[C@H](O)[C@@H](O)[C@H]5O)[C@@H](O)[C@H](O)[C@H]4O)cc3o2)cc1 | 0.412645834 |
| O=C(O)C1=CO[C@@H](O[C@@H]2O[C@H](CO)[C@@H](O)[C@H](O)[C@H]2O)[C@@H]2C(CO)=C[C@H](O)[C@H]12 | 0.648332571 |
| CC1CCC2(C(=O)O)CCC3(C)C(=CCC4C5(C)CC(O)C(O)C(C)(CO)C5C(O)CC43C)C2C1C | 0.876686869 |
| CC1CCCCCCCCCCCCC(=O)C1 | 0.614045773 |
| C=C(CC[C@@H](C(=O)O)[C@H]1CC[C@@]2(C)C3=C(CC[C@]12C)[C@@]1(C)CC[C@H](O)C(C)(C)[C@@H]1CC3)C(C)C | 0.876685176 |
| COc1ccc(-c2cc(=O)c3c(OC)cc(OC)c(OC)c3o2)cc1 | 0.564890452 |
| CC(C)(O)[C@@H]1CC[C@](C)([C@H]2[C@@H](O)C[C@@]3(C)[C@@H]4C[C@H](O)[C@H]5C(C)(C)[C@@H](O)CC[C@@]56C[C@@]46CC[C@]23C)O1 | 0.862663869 |
| C=C1C(=O)O[C@@H]2/C=C(\C)CC/C=C(\C)CC[C@@H]12 | 0.647025171 |
| CC(C)(O)[C@H]1CC[C@@](C)([C@H]2[C@@H](O)C[C@@]3(C)[C@@H]4C[C@H](O)[C@H]5C(C)(C)[C@@H](O)CC[C@@]56C[C@@]46CC[C@]23C)O1 | 0.862663869 |
| CC1(C)CC[C@]2(CO)CC[C@]3(C)C(=CC[C@@H]4[C@@]5(C)CC[C@H](O)C(C)(C)[C@@H]5CC[C@]43C)[C@@H]2C1 | 0.844312943 |
| COc1cc([C@H]2Oc3ccc([C@H]4Oc5cc(O)cc(O)c5C(=O)[C@@H]4O)cc3O[C@@H]2CO)ccc1O | 0.527064328 |
| O=c1c2ccccc2[nH]c2ccccc12 | 0.540451076 |
| OCCCc1ccc(O)cc1 | 0.543356283 |
| O=c1c(O)c(-c2ccc(O)c(O)c2)oc2c(O[C@@H]3O[C@H](CO)[C@@H](O)[C@H](O)[C@H]3O)c(O)cc(O)c12 | 0.513307776 |
| COc1ccc([C@@H]2OC[C@@H]3[C@H]2CO[C@H]3c2ccc(OC)c(OC)c2)cc1OC | 0.513088077 |
| C/C=C1/[C@H](O[C@@H]2O[C@H](CO)[C@@H](O)[C@H](O)[C@H]2O)OC=C(C(=O)OC)[C@H]1CC(=O)OCCc1ccc(O)c(O)c1 | 0.390685746 |
| COc1cc2c(cc1CC=C(C)C)C(=O)C[C@@H](c1ccc(O)cc1)O2 | 0.585783132 |
| C[C@H]1[C@H](C)CC[C@]2(CO)CC[C@]3(C)C(=CC[C@@H]4[C@@]5(C)CC[C@H](O)C(C)(C)[C@@H]5CC[C@]43C)[C@H]12 | 0.874679803 |
| O=c1c2cc(O)c(O)cc2oc2cc(O)cc(O)c12 | 0.594957595 |
| O=C1C[C@@H](c2ccc(O)cc2)Oc2cc(O)cc(O)c21 | 0.625509927 |
| CC1(C)C=Cc2c(ccc(-c3coc4cc(O)ccc4c3=O)c2O)O1 | 0.563174564 |
| CCCC(=O)c1c(O)c(Cc2c(O)c(Cc3c(O)c(C)c(OC)c(C(=O)CCC)c3O)c(O)c(C(=O)[C@@H](C)CC)c2O)c(O)c(C)c1OC | 0.368432376 |
| C[C@@H]1O[C@@H](O[C@H]2CC[C@]3(C=O)[C@H]4CC[C@]5(C)[C@@H](C6=CC(=O)OC6)CC[C@]5(O)[C@@H]4CC[C@]3(O)C2)[C@H](O)[C@H](O)[C@H]1O | 0.680224369 |
| CC(C)C1=CC2=CCC3[C@](C)(C(=O)O)CCC[C@]3(C)[C@H]2CC1 | 0.665548021 |
| COc1cc(O)cc2c1C(=O)C[C@@H](c1ccccc1)O2 | 0.599527469 |
| C/C=C(/C)C(=O)Oc1c(OC)c(OC)cc2c1-c1c(cc(OC)c(OC)c1OC)CC(C)(O)C(C)C2 | 0.481259965 |
| CCCCCC/C=C/CCCCCCCC(=O)O | 0.590570218 |
| CCCCCCCCCCCCCCCC(=O)NCCO | 0.546377724 |
| CCCCC[C@H](O)/C=C/C=C\CCCCCCCC(=O)O | 0.523209232 |
| CC(C)=CCC[C@@](C)(O)[C@H]1CC[C@]2(C)[C@@H]1[C@H](O)C[C@@H]1[C@@]3(C)CC[C@H](O)C(C)(C)[C@@H]3[C@@H](O)C[C@]12C | 0.907303429 |
| OCC=Cc1ccccc1 | 0.58709995 |
| COc1ccc(-c2cc(=O)c3c(O)cc(O)cc3o2)cc1-c1c(O)cc(O)c2c(=O)cc(-c3ccc(O)cc3)oc12 | 0.417144275 |
| O=c1cc(-c2ccccc2)oc2cc(O[C@@H]3O[C@H](CO)[C@@H](O)[C@H](O)[C@H]3O)c(O)c(O)c12 | 0.55735369 |
| CC1(C)CC[C@]2(C(=O)O)[C@H](O)C[C@]3(C)C(=CC[C@@H]4[C@@]5(C)CC[C@H](O)[C@@](C)(CO)[C@@H]5CC[C@]43C)[C@@H]2C1 | 0.868473635 |
| O=c1c(O)c(-c2ccc(O)c(O)c2)oc2cc(O)ccc12 | 0.627827967 |
| C/C=C(/CC[C@@H](C)[C@H]1CC[C@H]2[C@@H]3CC=C4CC(O)CC[C@]4(C)[C@H]3CC[C@]12C)C(C)C | 0.813613923 |
| COC(=O)[C@]12CCC(C)(C)C[C@H]1C1=CC[C@@H]3[C@@]4(C)CCC(=O)C(C)(C)[C@@H]4CC[C@@]3(C)[C@]1(C)CC2 | 0.782050012 |
| C=CC[S+]([O-])C[C@H](N)C(=O)O | 0.636378835 |
| CC1(C)C=Cc2c(ccc3c2OC[C@@H](c2ccc(O)cc2O)C3)O1 | 0.480961406 |
| COc1ccc(-c2oc3cc(OC)cc(OC)c3c(=O)c2OC)cc1 | 0.580456387 |
| CC(=O)CCc1ccc(O)cc1 | 0.538179394 |
| C/C=C/c1cc(OC)c2c(c1)[C@@H](C)[C@H](c1ccc3c(c1)OCO3)O2 | 0.461502059 |
| O=c1c(-c2ccc(O)cc2)coc2cc(O)ccc12 | 0.614766314 |
| COc1cc2c(cc1O)[C@H](Cc1ccc(O)cc1)NCC2.Cl | 0.563813819 |
| C[C@H]1CC[C@H]2[C@H](C)[C@@H]3CC[C@@H]4[C@@H](C[C@H]5[C@H]4C[C@@H](O)[C@H]4C[C@@H](O)CC[C@@]45C)[C@@H]3CN2C1 | 0.761345787 |
| CN1C2CC(OC(=O)C(CO)c3ccccc3)CC1C(O)C2 | 0.53928155 |
| COc1cc(/C=C/C(N)=O)ccc1O | 0.564575191 |
| CN1[C@@H](CC(=O)c2ccccc2)CCC[C@H]1C[C@H](O)c1ccccc1.Cl | 0.583941672 |
| Oc1cc(O)cc(/C=C/c2c(O)cccc2O)c1 | 0.691896424 |
| CCCC[N+]1(C)[C@@H]2C[C@@H](OC(=O)[C@H](CO)c3ccccc3)C[C@H]1[C@@H]1O[C@@H]12.[Br-] | 0.509925758 |
| COc1cc(-c2c(C)cc(O)cc2O[C@@H]2O[C@H](CO)[C@@H](O)[C@H](O)[C@H]2O)oc(=O)c1 | 0.655678141 |
| Nc1ncnc2[nH]cnc12 | 0.610199614 |
| CN1[C@H]2C[C@H](OC(=O)[C@H](CO)c3ccccc3)C[C@@H]1[C@@H](O)C2 | 0.53928155 |
| COC(=O)C1=CCCN(C)C1 | 0.617261992 |
| CN1[C@@H]2CC[C@H]1CC(OC(=O)[C@H](CO)c1ccccc1)C2 | 0.575644104 |
| COc1cc(O[C@@H]2O[C@H](CO[C@@H]3O[C@H](CO)[C@@H](O)[C@H](O)[C@H]3O)[C@@H](O)[C@H](O)[C@H]2O)c2c(c1)cc(O)c1c(=O)cc(C)oc12 | 0.434114343 |
| C=C1CCC[C@]2(C)CC[C@@H](C(C)(C)O)C[C@@H]12 | 0.633017623 |
| CC(C)(O[C@@H]1O[C@H](CO)[C@@H](O)[C@H](O)[C@H]1O)[C@@H]1CC[C@](O)(CO)[C@H]2CC(=O)[C@@](C)(O)[C@@H]2C1 | 0.604847063 |
| CCOC1c2c(ccc3c2OCO3)-c2ccc3cc4c(cc3c2N1C)OCO4 | 0.50943446 |
| CN1C[C@@H]2C[C@H](C1)c1cccc(=O)n1C2 | 0.530190589 |
| O=c1cc(O)c2ccccc2o1 | 0.510768094 |
| O=c1cccc2n1C[C@@H]1CNC[C@H]2C1 | 0.55266877 |
| CC1=C2C[C@H]3[C@@H](CC(=O)[C@H]4C[C@@H](O)CC[C@@]43C)[C@@H]2CC[C@]12O[C@@H]1C[C@H](C)CN[C@H]1[C@H]2C | 0.815276391 |
| C1CCN2C[C@H]3C[C@H](CN4CCCC[C@@H]34)[C@H]2C1 | 0.564889299 |
| CC1(C)CC[C@]2(C(=O)O)CC[C@]3(C)C(=CC[C@@]4(C)[C@@]3(C)CC[C@H]3C(C)(C)[C@H](O)CC[C@@]34C)[C@]2(C)C1 | 0.894289979 |
| CN1[C@@H](C[C@@H](O)c2ccccc2)CCC[C@H]1C[C@H](O)c1ccccc1 | 0.495413271 |
| O[C@H]1C[C@H]2CC[C@@H](C1)N2 | 0.561490252 |
| COc1cc(O)c2c(c1)O[C@H](c1ccc(O)c(O)c1)CC2=O | 0.600975763 |
| COC(=O)C1=CCCNC1.Cl | 0.633665509 |
| COc1ccc2nccc(C(=O)O)c2c1 | 0.624667658 |
| COC(=O)CC(O)(CCCC(C)(C)O)C(=O)OC1C(OC)=CC23CCCN2CCc2cc4c(cc2C13)OCO4 | 0.415676649 |
| C[C@H](CC[C@@H](O)C(C)(C)O)[C@H]1CC[C@@]2(C)[C@@H]3CC=C4[C@@H](CC[C@H](O)C4(C)C)[C@]3(C)[C@H](O)C[C@]12C | 0.891819629 |
| O=C1C=CC[C@@H]2[C@H]3CCCN4CCC[C@@H](CN12)[C@@H]34 | 0.62516364 |
| COc1cc(C(=O)O)cc(O)c1O | 0.555816649 |
| C=C1C(=O)[C@@]23[C@H]4O[C@H]5O[C@]2(O)[C@@H](O)[C@@H]2C(C)(C)CC[C@H](O)C52[C@@H]3CC[C@@H]14 | 0.746082732 |
| O=C1COc2cc(O)ccc2-c2cc(O)c(O)cc2C1 | 0.652675171 |
| C=C1CCC[C@]2(C)C[C@@H]3OC(=O)C(C)=C3C[C@@H]12 | 0.657236145 |
| COc1c(O)cc2oc3cc(O)c(CC=C(C)C)c(O)c3c(=O)c2c1CCC(C)(C)O | 0.554679401 |
| C=C(C)[C@H](CC=C(C)C)Cc1c(O)cc(OC)c2c1O[C@H](c1ccc(O)cc1O)CC2=O | 0.544846277 |
| CC1OC(OC2CCC3(C)C(CCC4(C)C3C=CC35OCC6(CCC(C)(C)CC63)C(O)CC45C)C2(C)CO)C(O)C(OC2OC(CO)C(O)C(O)C2O)C1O | 0.788042615 |
| CC(C)=CCc1c(-c2ccc(O)cc2O)oc2c3c(cc(O)c2c1=O)OC(C)(C)C=C3 | 0.580571455 |
| COc1ccc(-c2oc3cc(OC)c(OC)c(O)c3c(=O)c2OC)cc1O | 0.558937144 |
| C[C@H](CC(=O)C[C@@H](C)[C@H]1CC(=O)[C@@]2(C)C3=C(C(=O)C[C@]12C)[C@@]1(C)CC[C@H](O)C(C)(C)[C@@H]1C[C@@H]3O)C(=O)O | 0.748821073 |
| O=C(/C=C/c1ccccc1)OC[C@H]1O[C@@H](O[C@@H]2OC=C[C@H]3[C@H](O)[C@@H]4O[C@]4(CO)[C@@H]23)[C@H](O)[C@@H](O)[C@@H]1O | 0.517574766 |
| O=C(O)[C@H]1O[C@@H](Oc2cc3oc(-c4ccc(O)cc4)cc(=O)c3c(O)c2O)[C@H](O)[C@@H](O)[C@@H]1O | 0.53354711 |
| COc1cc(O)c2c(=O)cc(-c3ccccc3)oc2c1 | 0.590303281 |
| CC1(C)C=Cc2cc(-c3coc4cc(O)ccc4c3=O)ccc2O1 | 0.520108438 |
| COc1ccc(-c2cc(=O)c3c(O)cc(O)c(-c4cc(-c5cc(=O)c6c(O)cc(O)cc6o5)ccc4O)c3o2)cc1 | 0.416363025 |
| COc1ccc2c(c1OC)C(OC)N(C)c1c-2ccc2cc3c(cc12)OCO3 | 0.503668387 |
| C=C[C@@]1(C)Cc2occ(C)c2[C@@H]2OC(=O)C(=C)[C@@H]21 | 0.672624368 |
| CC(=O)Oc1ccc(/C=C/c2cc(OC(C)=O)cc(OC(C)=O)c2)cc1 | 0.57632319 |
| C=C1C(=O)[C@]23C[C@H]1CC[C@H]2[C@@]12CO[C@]3(O)[C@@H](O)[C@@H]1C(C)(C)C=CC2=O | 0.609012084 |
| COc1cccc(OC)c1C(=O)OCc1cc(O)ccc1O[C@@H]1O[C@H](CO)[C@@H](O)[C@H](O)[C@H]1O | 0.544744476 |
| C=C1CCC[C@]2(C)C=C3OC(=O)C(C)=C3C[C@@H]12 | 0.626417716 |
| CC(C)=CCC[C@](C)(O)[C@H]1CC[C@]2(C)[C@@H]1[C@H](O)C[C@@H]1[C@@]3(C)CC[C@H](O)C(C)(C)[C@@H]3[C@@H](O)C[C@]12C | 0.907303429 |
| CC1(C)C[C@H]2C3=CC[C@@H]4[C@@]5(C)CC[C@H](O)[C@@](C)(CO)[C@@H]5CC[C@@]4(C)[C@]3(C)C[C@H](O)[C@@]2(CO)[C@@H](O)[C@@H]1O | 0.862985063 |
| COC(=O)c1ccc(O)cc1O | 0.555229832 |
| O=c1oc2c(O)c(O)cc3c(=O)oc4c(O)c(O)cc1c4c23 | 0.604000052 |
| COc1cc2ccc(=O)oc2c(OC)c1OC | 0.67188938 |
| C[C@@H]1O[C@@H](OC[C@H]2O[C@@H](Oc3cc(O)c4c(c3)O[C@H](c3ccc(O)c(O)c3)CC4=O)[C@H](O)[C@@H](O)[C@@H]2O)[C@H](O)[C@H](O)[C@H]1O | 0.46864507 |
| O=c1ccc2cc(O)ccc2o1 | 0.493370147 |
| COc1ccc([C@@H]2OC[C@H]3[C@@H]2CO[C@@H]3c2ccc(O[C@@H]3O[C@H](CO)[C@@H](O)[C@H](O)[C@H]3O)cc2OC)cc1OC | 0.436600582 |
| C[C@@H]1CC[C@]2(C(=O)O)CC[C@]3(C)C(=CC[C@@H]4[C@@]5(C)CC[C@H](O)C(C)(C)[C@@H]5CC[C@]43C)[C@@H]2[C@]1(C)O | 0.871133188 |
| COc1ccc2cc3[n+](cc2c1OC)CCc1cc(O)c(O)cc1-3 | 0.496846354 |
| CCCCC[C@H](O)CC(=O)CCc1ccc(O)c(OC)c1 | 0.593394826 |
| CC(C)=CCC/C(C)=C/Cc1c(O)ccc(C(=O)/C=C/c2ccc(O)cc2)c1O | 0.531669327 |
| CCCCCCCCCCCCCCCCCCCCCCCCCCO | 0.509591032 |
| Nc1ncnc2c1ncn2[C@@H]1O[C@H](COP(=O)(O)O)[C@@H](O)[C@H]1O | 0.673381516 |
| COc1c(-c2ccccc2)oc2c(ccc3occc32)c1=O | 0.56259871 |
| C=C(C)[C@@H]1CCC2=CC(=O)C[C@@H](C)[C@]2(C)C1 | 0.654822173 |
| COC(=O)/C=C/c1ccccc1 | 0.615731738 |
| O=C(/C=C/c1ccc(O)c(O)c1)c1ccc(O[C@@H]2O[C@H](CO)[C@@H](O)[C@H](O)[C@H]2O)c(O)c1O | 0.575730366 |
| Oc1ccc2c(c1)OCO2 | 0.422481161 |
| C=CC1C(OC2OC(CO)C(O)C(O)C2OC(=O)c2c(O)cc(O)cc2-c2cccc(O)c2)OC=C2C(=O)OCCC21 | 0.474842299 |
| COc1c(O)cc2c(c1OC)-c1c(cc(O)c(OC)c1OC)CC(C)C(C)C2 | 0.55165968 |
| C[C@@H]1C(=O)O[C@H]2[C@@H](O)[C@]34[C@@H]5OC(=O)[C@]3(O[C@@H]3OC(=O)[C@H](O)[C@@]34[C@H](C(C)(C)C)[C@H]5O)[C@@]12O | 0.605925871 |
| O=C(O)[C@H]1O[C@@H](Oc2c(O)cc(O)c3c(=O)c(O)c(-c4ccc(O)c(O)c4)oc23)[C@H](O)[C@@H](O)[C@@H]1O | 0.50202734 |
| COc1ccc([C@@H]2CC(=O)c3c(O)cc(O[C@@H]4O[C@H](CO)[C@@H](O)[C@H](O)[C@H]4O)cc3O2)cc1O | 0.564577443 |
| C/C=C/c1cc(OC)c(OC)cc1OC | 0.60293037 |
| COc1cc2c(cc1OC)-c1c(OC)c(OC)cc3c1[C@H](C2)N(C)CC3 | 0.509178217 |
| COc1c(OC)c2nccc3c4ccccc4n(c1=O)c23 | 0.588171885 |
| Oc1cc(O)cc(-c2cc3ccc(O)cc3o2)c1 | 0.582325498 |
| CCOC(=O)C1=CC2(CC)CCCN3CCc4c(n1c1ccccc41)C32 | 0.63245902 |
| COc1cc(O)c2c(=O)c(OC)c(-c3ccc(OC)c(O)c3)oc2c1 | 0.591148953 |
| C[C@@H]1C(=O)O[C@H]2C[C@]34[C@@H]5OC(=O)C3(O[C@@H]3OC(=O)[C@H](O)C34[C@H](C(C)(C)C)[C@H]5O)[C@]21O | 0.624321903 |
| COc1c(OC(C)=O)cc(OC(C)=O)c2c(=O)cc(-c3ccccc3)oc12 | 0.518437563 |
| COc1c(-c2ccc(O)c(O)c2)oc2cc(O)cc(O)c2c1=O | 0.649200755 |
| COc1cc2ccc(=O)oc2c(O[C@@H]2O[C@H](CO)[C@@H](O)[C@H](O)[C@H]2O)c1O | 0.693024989 |
| Nc1nc2ncc(C(=O)O)nc2c(=O)[nH]1 | 0.643026684 |
| COc1c2ccoc2nc2c(O)cccc12 | 0.546922523 |
| CC1(C)[C@H](C(=O)O)CC[C@]1(C)C(=O)O | 0.569666659 |
| N[C@@H](CCC(=O)N[C@@H](CSCc1ccc(O)cc1)C(=O)NCC(=O)O)C(=O)O | 0.560053401 |
| COc1ccc2c(c1)O[C@H]1c3ccc(O)cc3OC[C@@H]21 | 0.492107632 |
| C[C@H](CCC(=O)O)[C@H]1CC(=O)[C@@]2(C)C3=C(C(=O)C[C@]12C)[C@@]1(C)CCC(=O)C(C)(C)[C@@H]1C[C@@H]3O | 0.723560149 |
| COc1cc2oc(-c3ccc(O)c(O)c3)c(OC)c(=O)c2c(O)c1OC | 0.584320103 |
| C=C[C@H]1[C@H](O[C@@H]2O[C@H](CO)[C@@H](O)[C@H](O)[C@H]2O)OC=C(C(=O)OC)[C@H]1CCOC(=O)c1cc(O)c(O)c(O)c1 | 0.423929682 |
| c1cc2c(cc1O[C@H]1OC[C@H]3[C@@H]1CO[C@@H]3c1ccc3c(c1)OCO3)OCO2 | 0.461640552 |
| C/C=C\c1cc(OC)c(OC)cc1OC | 0.60293037 |
| COc1cc(OC)c(CCC(=O)c2ccc(O)cc2)c(OC)c1 | 0.590212212 |
| O=c1c(-c2ccc(O)c(O)c2)coc2cc(O)ccc12 | 0.598532823 |
| CC(C)=CCc1c(O)c(CC=C(C)C)c2oc3c(O)ccc(O)c3c(=O)c2c1O | 0.572455153 |
| C[C@@H]1[C@H](C)[C@@H](c2ccc3c(c2)OCO3)O[C@@H]1c1ccc2c(c1)OCO2 | 0.456117674 |
| COc1cc2cc(Oc3ccc4ccc(=O)oc4c3)c(=O)oc2cc1O | 0.564052924 |
| COc1ccc([C@H]2OC[C@H]3[C@@H]2CO[C@@H]3c2ccc(OC)c(OC)c2)cc1OC | 0.513088077 |
| CC1=C[C@H]2[C@@]3(O)[C@H](C)[C@@H](O)[C@]4(O)[C@H]([C@@H]3C=C(CO)C[C@]2(O)C1=O)C4(C)C | 0.688733838 |
| O=C(/C=C/c1ccc(O)c(O)c1)OC[C@H]1O[C@@H](OCCc2ccc(O)c(O)c2)[C@H](O)[C@@H](O[C@@H]2O[C@H](CO)[C@@H](O)[C@H](O)[C@H]2O)[C@@H]1O | 0.489336993 |
| Cc1cc(O)c2c(=O)c3c(O)cc(O)c4c5c(O)cc(O)c6c(=O)c7c(O)cc(C)c8c1c2c(c34)c(c78)c65 | 0.475795472 |
| C[C@@H]1O[C@@H](Oc2c(-c3cc(O)c(O)c(O)c3)oc3cc(O)cc(O)c3c2=O)[C@H](O)[C@H](O)[C@H]1O | 0.49457185 |
| COc1ccc([C@@H]2CC(=O)c3c(O)cc(OC4OC(COC5OC(C)C(O)C(O)C5O)C(O)C(O)C4O)cc3O2)cc1OC | 0.425093545 |
| CC1=C[C@]23C(=O)[C@@H](C=C(CO)[C@@H](O)[C@]2(O)[C@H]1O)[C@H]1[C@@H](C[C@H]3C)C1(C)C | 0.709826799 |
| C/C=C(/C)C(=O)O[C@H]1C(C)=C[C@]23C(=O)[C@@H](C=C(CO)[C@@H](O)[C@]12O)[C@H]1[C@@H](C[C@H]3C)C1(C)C | 0.596511024 |
| CC(C)=CC(=O)O[C@H]1Cc2cc3ccc(=O)oc3cc2OC1(C)C | 0.592915889 |
| O=c1c(-c2ccc(O)cc2)coc2cc(O)c(O)cc12 | 0.608641434 |
| Oc1cc(O)c2c(c1)OC1(c3ccc(O)c(O)c3)Oc3cc(O)c4c(c3C2C1O)OC(c1ccc(O)c(O)c1)C(O)C4 | 0.45912503 |
| COc1cc(-c2cc(=O)c3c(O)c(OC)c(OC)c(OC)c3o2)cc(OC)c1OC | 0.519738183 |
| CC(=O)O[C@H]([C@@H]1C[C@@H](C)C2=C3C[C@H](O)[C@H]4[C@@]5(C)CCC(=O)C(C)(C)[C@@H]5CC[C@]4(C)[C@@]3(C)C[C@@H]2O1)C(C)(C)O | 0.764766257 |
| C/C(=C\C(=O)CC(C)C(=O)O)[C@@H]1CC(=O)[C@@]2(C)C3=C(C(=O)C[C@]12C)[C@@]1(C)CC[C@H](O)C(C)(C)C1C[C@@H]3O | 0.715678991 |
| COc1cc(-c2oc3cc(OC)c(OC)c(O)c3c(=O)c2OC)ccc1O | 0.558937144 |
| CC(=O)O[C@@H]1[C@H]2[C@@H](OC(=O)Cc3ccccc3)[C@@H](C)C[C@]2(OC(C)=O)C(=O)/C(C)=C\[C@@H]2[C@H](CC[C@]13CO3)C2(C)C | 0.513802172 |
| CC(C)=CCc1c(O[C@@H]2O[C@H](CO)[C@@H](O)[C@H](O)[C@H]2O)cc(O)c2c1O[C@H](c1ccc(O)cc1)[C@@H](O)C2=O | 0.477160759 |
| CC(C)=CCC[C@](C)(O)[C@H]1CC[C@]2(C)[C@@H]1[C@H](O)C[C@@H]1[C@@]3(C)CC[C@H](O)C(C)(C)[C@@H]3CC[C@]12C | 0.902978236 |
| CC(=O)O[C@H]1[C@H]2O[C@]23[C@@H]2CC[C@]4(O)C[C@@H](O)CC[C@]4(C)[C@H]2CC[C@]3(C)[C@H]1c1ccc(=O)oc1 | 0.583855228 |
| COc1ccc(-c2cc(=O)c3c(OC)cc(OC)c(OC)c3o2)cc1OC | 0.523125541 |
| C=C1[C@H](O)C[C@H]2[C@@H](/C=C(\C)C(=O)[C@@]3(O)C[C@H](C)[C@H](O)[C@@H]3[C@H]1O)C2(C)C | 0.703990015 |
| O=C(OCC1=C[C@@H](O)[C@@H]2C=CO[C@@H](O[C@@H]3O[C@H](CO)[C@@H](O)[C@H](O)[C@H]3O)[C@H]12)c1ccc(O)cc1 | 0.57391718 |
| O=c1ccoc2cc(O)ccc12 | 0.456690091 |
| C=C1C(=O)O[C@@H]2CC(C)=C([C@@H](C)CCCOC(C)=O)[C@@H](O)[C@H]12 | 0.498755603 |
| O=C(O)CC(O)(CC(=O)O)C(=O)O | 0.583388595 |
| C[C@H](O)C(=O)O | 0.614402828 |
| CC(C)=CCC12Oc3cc(O)ccc3C1(O)Oc1cc(O)c(C3C=C(C)CC(c4ccc(O)cc4O)C3C(=O)c3ccc(O)cc3O)c(O)c1C2=O | 0.398043826 |
| CN(C)CCc1ccc(O)cc1 | 0.627742596 |
| O=C(O)/C=C/c1ccc2c(c1)OCO2 | 0.481971278 |
| O=c1ccn([C@H]2O[C@@H](CO)[C@H](O)[C@@H]2O)c(=O)[nH]1 | 0.624929512 |
| COc1cc(O)c2c(c1)O[C@H](c1ccc(OC)c(OC)c1)CC2=O | 0.629677954 |
| Cc1cc(O)c2c(c1)Cc1cc(O)cc(O)c1C2=O | 0.593517821 |
| CCCCCc1cc(O)cc(O)c1C(=O)O | 0.711928859 |
| CC(=O)c1c(O)cc(O)cc1O | 0.537607963 |
| O=C(O)/C=C/c1ccc(O)cc1 | 0.590068118 |
| c1csc(-c2ccc(-c3cccs3)s2)c1 | 0.565306941 |
| COc1ccc2cc1Oc1ccc(cc1)CC1c3cc(c(OC)cc3CCN1C)Oc1c(O)c(OC)cc3c1C(C2)N(C)CC3 | 0.392376592 |
| COc1ccc(-c2oc3cc(O)cc(O)c3c(=O)c2OC)cc1 | 0.63112101 |
| COC(=O)[C@]12CCCC(C)(C)[C@@H]1CCc1cc(C(C)C)c(O)c(O)c12 | 0.593014837 |
| CC(C)=CCCC(C)=CCCC(C)=CCO | 0.616691811 |
| COC(=O)c1cc(O)c(OC)c(OC)c1 | 0.657864929 |
| S=C=NCc1ccccc1 | 0.603942401 |
| CC(C)=CCc1cc(C(=O)/C=C/c2ccc(O)cc2)c(O)cc1O | 0.612906393 |
| COC(=O)c1ccc(O)cc1 | 0.584868582 |
| Cl.O[C@H]1CCN2Cc3ccccc3N=C12 | 0.610889707 |
| O=C(O)c1ccco1 | 0.604420178 |
| CC(C)=CCc1cc([C@@H]2CC(=O)c3ccc(O)c(CC=C(C)C)c3O2)ccc1O | 0.581516439 |
| C[C@@H]1CC[C@]2(C(=O)O)CC[C@]3(C)C(=CC[C@@H]4[C@@]5(C)C[C@@H](O)[C@@H](O)C(C)(C)[C@@H]5CC[C@]43C)[C@@H]2[C@]1(C)O | 0.87042676 |
| O=C1C=CC[C@@H]2[C@H]3CCC[N@+]4([O-])CCC[C@@H](CN12)[C@@H]34 | 0.636653121 |
| COc1cc2ccc(=O)oc2c2ccoc12 | 0.547640258 |
| CC(=O)O[C@@H]1CC[C@@]2(C)[C@@H](CC[C@]3(C)[C@@H]2CC=C2[C@@H]4CC(C)(C)CC[C@]4(C)CC[C@]23C)[C@@]1(C)C(=O)O | 0.770837299 |
| CC(C)=CCc1cc2ccc(=O)oc2cc1O | 0.700117881 |
| CN1c2ccccc2C(=O)N2CCc3c([nH]c4ccccc34)[C@H]21 | 0.567521007 |
| CCCCC[C@H](O)/C=C/[C@H]1[C@H](O)CC(=O)[C@@H]1C/C=C\CCCC(=O)O | 0.516028443 |
| CC(C)=CCC[C@H](C)[C@@H]1CC[C@]2(C)C3=C(CC[C@@]12C)[C@@]1(C)CC[C@H](O)C(C)(C)[C@@H]1CC3 | 0.815143274 |
| C=C1CC[C@]2(C)CC[C@]3(C)[C@@H](CC[C@@H]4[C@@]5(C)CC[C@H](O)C(C)(C)[C@@H]5CC[C@]43C)[C@@H]2[C@@H]1C | 0.879436592 |
| C=C(C)[C@@H]1CC[C@]2(C(=O)O)CC[C@]3(C)[C@H](CC[C@@H]4[C@@]5(C)CC[C@@H](O)C(C)(C)[C@@H]5CC[C@]43C)[C@@H]12 | 0.902206477 |
| C=C[C@]1(C)C=C2CC[C@H]3[C@@](C)(CCC[C@@]3(C)C(=O)O)[C@@H]2CC1 | 0.702555988 |
| O=C(O)C=Cc1ccc(O)cc1 | 0.590068118 |
| COc1ccc(-c2oc3c(CC=C(C)C)c(O)cc(O)c3c(=O)c2O[C@@H]2O[C@@H](C)[C@H](O)[C@@H](O)[C@H]2O)cc1 | 0.477668208 |
| NC(=O)c1ccc[n+]([O-])c1 | 0.57873263 |
| CC(C)=CCOc1c2ccoc2c(OCC=C(C)C)c2oc(=O)ccc12 | 0.627840347 |
| COc1ccc(-c2oc3cc(OC)cc(O)c3c(=O)c2OC)cc1 | 0.63104172 |
| CC(C)=CCc1c(O)cc(O)c2c(=O)c(O[C@@H]3O[C@@H](C)[C@H](O)[C@@H](O)[C@H]3O)c(-c3ccc(O)cc3)oc12 | 0.492441093 |
| COc1ccc(-c2cc(=O)c3c(O)c(OC)c(OC)c(OC)c3o2)cc1 | 0.555581904 |
| O=c1c(O[C@@H]2O[C@H](CO)[C@@H](O)[C@H](O)[C@H]2O)c(-c2ccc(O)c(O)c2)oc2cc(O)cc(O)c12 | 0.501438229 |
| C=CCc1cc(OC)c2c(c1)OCO2 | 0.459593454 |
| CC(C)=CCc1c(O)cc(-c2cc3ccc(O)cc3o2)cc1O | 0.547443263 |
| C[C@]1(OC(=O)/C=C/c2ccccc2)C[C@@H](O)[C@]2(O)C=CO[C@@H](O[C@@H]3O[C@H](CO)[C@@H](O)[C@H](O)[C@H]3O)[C@@H]21 | 0.544387277 |
| CC1=C(CCC(C)COC2OC(CO)C(O)C(O)C2O)OC2CC3C4CCC5CC(OC6OC(CO)C(O)C(O)C6OC6OC(CO)C(O)C(O)C6O)CCC5(C)C4CCC3(C)C12 | 0.644248644 |
| C[n+]1c2n(c(=O)c3ccccc31)CCc1c-2[n-]c2ccccc12 | 0.576592051 |
| COC(=O)Nc1ccc(C)c(NC(=O)OC)c1 | 0.697109086 |
| COc1cc(-c2coc3cc4c(c(OC)c3c2=O)OCO4)cc(OC)c1OC | 0.523185396 |
| CC(=O)OCC1=C[C@@H]2OC(=O)C3=CO[C@@H](O[C@@H]4O[C@H](CO)[C@@H](O)[C@H](O)[C@H]4O)[C@H]1[C@@H]32 | 0.579247551 |
| C=C[C@H]1[C@H](O[C@@H]2O[C@H](CO)[C@@H](O)[C@H](O)[C@H]2O)OC=C2C(=O)OC(O)C[C@H]21 | 0.668456841 |
| CC1(C)O[C@H]2[C@H](O)CC(C(=O)O)=C[C@H]2O1 | 0.641383863 |
| COc1c2c(cc3oc(CO[C@@H]4O[C@H](CO)[C@@H](O)[C@H](O)[C@H]4O)cc(=O)c13)O[C@H](C(C)(C)O)C2 | 0.545462016 |
| CC(=O)O[C@@H]1CC[C@]2(C)C3=C(CC[C@H]2C1(C)C)[C@]1(C)CC[C@H]([C@@H](CCC=C(C)C)C(=O)O)[C@@]1(C)CC3 | 0.761397194 |
| CC(=O)OC[C@@H](COC(=O)/C=C/c1ccc(O)cc1)O[C@@H]1O[C@H](CO)[C@@H](O)[C@H](O)[C@H]1O | 0.507075636 |
| COc1cc(OC)c2c(=O)cc(-c3ccccc3)oc2c1OC | 0.593292253 |
| CCOC(=O)C=Cc1ccc(O)c(O)c1 | 0.66991215 |
| O=C(/C=C/c1ccc(O)c(O)c1)O[C@@H]1CC(C(=O)O)=C[C@@H](O)[C@H]1O | 0.672981831 |
| COC(=O)C1=CO[C@@H](O[C@@H]2O[C@H](CO)[C@@H](O)[C@H](O)[C@H]2O)[C@@H]2C(COC(=O)SC)=C[C@H](O)[C@H]12 | 0.548862583 |
| O=c1c(O)c(-c2ccc(O)c(O)c2)oc2cc(O[C@@H]3O[C@H](CO)[C@@H](O)[C@H](O)[C@H]3O)cc(O)c12 | 0.491333783 |
| CCOC(=O)C=Cc1ccccc1 | 0.605681877 |
| CC(C)=CCC1(CC=C(C)C)C(=O)C(O)=C(O)C1=O | 0.565287681 |
| CC(C)=CCc1cc(-c2coc3cc(O)cc(O)c3c2=O)cc(O)c1O | 0.599368771 |
| C=C(C)/C=C/C/C(C)=C/COc1c2ccoc2cc2oc(=O)ccc12 | 0.564942954 |
| O=C1CC(c2ccc(O)cc2)Oc2ccccc21 | 0.596468338 |
| CC1(C)O[C@H]2CC(=O)OC[C@@]23[C@@H]1C(=O)[C@H](O)[C@]1(C)[C@@H]3CC[C@@]2(C)[C@H](c3ccoc3)OC(=O)[C@H]3O[C@@]312 | 0.659588523 |
| COC(=O)[C@@H]1[C@H]2C[C@H]3c4[nH]c5ccccc5c4CCN3C[C@@H]2CC[C@@H]1O.Cl | 0.616179437 |
| COc1ccc(/C=C/C(=O)O)cc1O | 0.558491725 |
| COc1ccc([C@@H]2OC[C@H]3[C@@H]2CO[C@@H]3c2ccc3c(c2)OCO3)cc1OC | 0.467032693 |
| CNCC(O)c1ccc(O)cc1 | 0.570644873 |
| COc1ccc2ccc(=O)oc2c1C[C@@H]1OC1(C)C | 0.637486507 |
| CN(C)Cc1c[nH]c2ccccc12 | 0.592365444 |
| COC(=O)[C@]12CCC(C)(C)C[C@H]1C1=CC[C@@H]3[C@@]4(C)C[C@@H](O)[C@H](O)C(C)(C)[C@@H]4CC[C@@]3(C)[C@]1(C)CC2 | 0.890008662 |
| CN1C2CC(O)CC1C1OC12 | 0.527280175 |
| Cl.Oc1ccc(CC2NCCc3cc(O)c(O)cc32)cc1 | 0.586738011 |
| O=C(/C=C/c1ccc(O)cc1)NCCc1c[nH]c2ccc(O)cc12 | 0.606464469 |
| COc1ccc2c(c1OC)O[C@H]1c3ccc(O)cc3OC[C@@H]21 | 0.556808273 |
| COc1cc(CCO)ccc1O | 0.539817241 |
| COC(=O)/C=C/C(=O)O | 0.57351615 |
| O=C(O)c1ccncc1 | 0.677511002 |
| CCOC(=O)/C=C/c1ccc(O)cc1 | 0.601703593 |
| CC[C@]12CCCN3CCc4c(n(c5ccccc45)C(=O)C1)[C@@H]32 | 0.59047794 |
| COC(=O)c1cc(OC)c(O)c(OC)c1 | 0.65902065 |
| CCCCC/C=C/C=C/C(=O)NCC(C)C | 0.725054374 |
| COc1cc(O)cc(O)c1C(=O)/C=C/c1ccccc1 | 0.674814748 |
| NCCc1c[nH]c2ccccc12 | 0.547037155 |
| CC(C)=C1CC[C@@H](C)CC1=O | 0.609963529 |
| C[C@]1(C(=O)O)CCC[C@]2(C)c3cc(O)ccc3CC[C@@H]12 | 0.575606067 |
| COc1cc(COC(=O)CCCC/C=C/C(C)C)ccc1O | 0.577517154 |
| CCCCCCCCC(=O)NCc1ccc(O)c(OC)c1 | 0.598350392 |
| C/C=C/C=C/C=C\CC/C=C/C(=O)NCC(C)(C)O | 0.629222657 |
| CCC(=O)c1cc2c(cc1OC)OCO2 | 0.560650108 |
| CC[C@H](/C=C/[C@@H](C)[C@H]1CC[C@H]2C3=CC[C@H]4C[C@@H](O)CC[C@]4(C)[C@H]3CC[C@]12C)C(C)C | 0.798167566 |
| COC(=O)c1ccccc1O | 0.563263244 |
| CC12CCC(CC1=O)C2(C)C | 0.52763956 |
| CC1(C)[C@@H]2CC[C@@]1(C)C(=O)C2 | 0.52763956 |
| O=C(O)C=Cc1ccc(O)c(O)c1 | 0.562190329 |
| O=C/C=C/c1ccccc1 | 0.592918772 |
| CCCCCCC[C@H](O)CC(=O)CCc1ccc(O)c(OC)c1 | 0.534513996 |
| O=C(CCCCc1ccc2c(c1)OCO2)N1CCCCC1 | 0.537337848 |
| COc1cc(CNC(=O)CCCCCCC(C)C)ccc1O | 0.55622265 |
| CC(C)C12CCC(C)(CC1)O2 | 0.509946531 |
| CC(C)[C@@H]1CC[C@@H](C)C[C@H]1O | 0.568555404 |
| CC1CC[C@@]2(OC1)O[C@H]1C[C@H]3[C@@H]4CC=C5C[C@@H](O)C[C@@H](O)[C@]5(C)[C@H]4CC[C@]3(C)[C@H]1[C@@H]2C | 0.83683999 |
| CC(=O)O[C@H]1CC[C@@]2(C)[C@H](CC[C@@H]3[C@@H]2[C@H](O)C(=O)[C@]2(C)[C@@H](c4ccc(=O)oc4)CC[C@]32O)C1 | 0.594181654 |
| O=CC=Cc1ccccc1 | 0.592918772 |
| COc1cc(-c2cc(=O)c3c(O)cc(O)cc3o2)cc(OC)c1O | 0.617871656 |
| O=C1Nc2ccccc2C1=C1Nc2ccccc2C1=O | 0.5577747 |
| CC1(C)CC[C@]2(C(=O)O)CC[C@]3(C)C(=CC[C@@H]4[C@@]5(C)CC[C@H](O)[C@@](C)(C=O)[C@@H]5CC[C@]43C)[C@@H]2C1 | 0.801996757 |
| COc1cc([C@@H]2Oc3cc([C@H]4Oc5cc(O)cc(O)c5C(=O)[C@@H]4O)ccc3O[C@H]2CO)ccc1O | 0.527343346 |
| COc1cc([C@@H]2c3cc4c(cc3C[C@H]3COC(=O)[C@@H]32)OCO4)cc(OC)c1O | 0.532797256 |
| CCCCCCCCCC(O)CCCCCCCC(=O)O | 0.542278605 |
| COc1cc(C=CC(=O)O)cc(OC)c1O | 0.673816827 |
| Nc1nc(=O)[nH]c2c1ncn2[C@@H]1O[C@H](CO)[C@@H](O)[C@H]1O | 0.647935449 |
| CC(C)=CCc1c(O)ccc(C(=O)[C@@H]2[C@@H](c3c(O)cc(O)c4c(=O)c(CC=C(C)C)c(-c5ccc(O)cc5O)oc34)C=C(C)C[C@H]2c2ccc(O)cc2O)c1O | 0.381203316 |
| Cc1c([C@H](C)[C@@H]2NC[C@@H](C)C[C@H]2O)ccc2c1C[C@H]1[C@H]2CC=C2C[C@@H](O[C@@H]3O[C@H](CO)[C@@H](O)[C@H](O)[C@H]3O)CC[C@@]21C | 0.628778515 |
| O=C(/C=C/c1ccccc1)N1CCCC1 | 0.643970931 |
| O=Cc1ccc(COCc2ccc(C=O)o2)o1 | 0.634520013 |
| Oc1ncnc2ccccc12 | 0.583690811 |
| COc1cc(C(C)C)ccc1C | 0.574805983 |
| C=C(C)[C@@H]1CC[C@]2(C=O)CC[C@]3(C)[C@H](CC[C@@H]4[C@@]5(C)CC[C@H](O)C(C)(C)[C@@H]5CC[C@]43C)[C@@H]12 | 0.88819747 |
| CC(C)=CCc1cc2c(cc1O)oc(=O)c1c3ccc(O)cc3oc21 | 0.590897256 |
| COc1ccc(CCc2cc(OC)c(OC)c(OC)c2)cc1O | 0.516161416 |
| COc1cc(C(=O)O)ccc1O | 0.550342278 |
| CC(C)c1cc2c(c(O)c1O)[C@@]1(C(=O)O)CCCC(C)(C)[C@@H]1CC2 | 0.589175463 |
| CC(=O)NCCCC(=O)O | 0.651508259 |
| CC1=CCC(C(C)(C)O)CC1 | 0.544406607 |
| CCCCCCCCCCCCCCCCCCOC(=O)[C@@]1(C)CC[C@]2(C)CC[C@]3(C)C(=CC(=O)[C@@H]4[C@@]5(C)CC[C@H](O)C(C)(C)C5CC[C@]43C)[C@H]2C1 | 0.701165963 |
| O=c1ccc2c(O)cc(O)cc2o1 | 0.51394184 |
| C[C@@H]1O[C@@H](OC[C@H]2O[C@@H](Oc3cc(O)c4c(c3)O[C@H](c3ccc(O)cc3)CC4=O)[C@H](O)[C@@H](O)[C@@H]2O)[C@H](O)[C@H](O)[C@H]1O | 0.454878782 |
| O=C(O)/C=C\C(=O)O | 0.60816908 |
| O=C(O)C(O)Cc1ccccc1 | 0.636011142 |
| OCC1=CC(O)C2C=COC(OC3OC(CO)C(O)C(O)C3O)C12 | 0.60519392 |
| CCOC(=O)c1cc(O)c(O)c(O)c1 | 0.582279838 |
| COC(=O)c1c[nH]c2ccccc12 | 0.560561387 |
| O=C(O)C1CCCCN1 | 0.612154719 |
| C[C@@H]1O[C@@H](O[C@H]2[C@H](Oc3cc(O)c4c(=O)cc(-c5ccc(O)c(O)c5)oc4c3)O[C@H](CO)[C@@H](O)[C@@H]2O)[C@H](O)[C@H](O)[C@H]1O | 0.432072919 |
| CC(=O)CCCC(C)CCCC(C)CCCC(C)C | 0.568255724 |
| COc1cc(C=O)cc(OC)c1OC | 0.592614468 |
| COc1cc(C(=O)O)cc(OC)c1O | 0.576218382 |
| CC(C)=CCC/C(C)=C/CC/C(C)=C/CC/C(C)=C/CC/C(C)=C/CC/C(C)=C/CC/C(C)=C/CC/C(C)=C/CC/C(C)=C/CO | 0.505366205 |
| COc1ccc(-c2cc(=O)c3c(O)c(OC)c(O)c(OC)c3o2)cc1 | 0.589016188 |
| CC(C)=CCCC1=CC(=O)c2c(O)ccc(O)c2C1=O | 0.535914818 |
| COc1cc(O)c2c(=O)c(O)c(-c3ccc(OC)c(O)c3)oc2c1 | 0.621598281 |
| CC1OC(OC2C(=O)c3c(O)cc(O)cc3OC2c2ccc(O)c(O)c2)C(O)C(O)C1O | 0.538609821 |
| Oc1cc(O)c2c(c1)O[C@@]1(c3ccc(O)c(O)c3)Oc3cc(O)c4c(c3[C@@H]2[C@H]1O)O[C@H](c1ccc(O)c(O)c1)[C@H](O)C4 | 0.45912503 |
| COC(=O)C1=CO[C@@H](O)[C@@H]2[C@@H](C)[C@@H](O)C[C@H]12 | 0.662872544 |
| CCCCCCCCCCCCC(=O)O | 0.698297824 |
| O=C(C=Cc1ccccc1)c1ccccc1 | 0.659681362 |
| CC1(C)Oc2cc3oc(=O)ccc3cc2CC1O | 0.62678335 |
| CCCCCCCCCCCC(=O)O | 0.674492393 |
| CCOC(=O)c1c(C)cc(O)cc1O | 0.586744943 |
| CC1=CC[C@@](O)(C(C)C)CC1 | 0.546705285 |
| CC1=CC[C@@]23C[C@@H]1C(C)(C)[C@@H]2CC[C@H]3C | 0.605563746 |
| CC1(C)CC[C@]2(C(=O)O)CCC3=C([C@@H](CCl)C[C@H]4[C@@]3(C)CCC3[C@]4(C)C[C@H](O)[C@H](O)[C@]3(C)C(=O)O)[C@H]2C1 | 0.73134583 |
| O=c1[nH]c(=O)c2ccccc2[nH]1 | 0.579661678 |
| CC1=C(C)[C@H]2C3=CC[C@@H]4[C@@]5(C)CC[C@H](O)C(C)(C)[C@@H]5CC[C@@]4(C)[C@]3(C)CC[C@@]2(C(=O)O)CC1 | 0.882255009 |
| Cc1cc2c([nH]c3ccccc32)c2c1OC(C)(C)C=C2 | 0.539044569 |
| COC(=O)C=Cc1ccc(O)c(O)c1 | 0.592184596 |
| COc1ccc(C=O)c(O)c1 | 0.520002865 |
| CC(=O)O[C@H]1C[C@H]2C(=O)O[C@H](c3ccoc3)C[C@@]2(C)[C@@H]2C[C@@H]3C[C@@H](C(=O)O3)[C@@H]12 | 0.612294314 |
| O=c1ccc2cc(OC3OC(CO)C(O)C(O)C3O)c(O)cc2o1 | 0.699088166 |
| CC(=O)NCCc1ccc(O)cc1 | 0.639250354 |
| CC1=C(O)C(=O)C(C)O1 | 0.547646021 |
| O=c1c(O[C@@H]2OC[C@H](O)[C@H](O)[C@H]2O)c(-c2ccc(O)c(O)c2)oc2cc(O)cc(O)c12 | 0.505858713 |
| O=C(O)c1ccc(O)cc1 | 0.589479007 |
| COc1c(O)ccc2c1C(=O)c1ccccc1C2=O | 0.539110196 |
| COc1cc(/C=C/C=O)cc(OC)c1O | 0.62648251 |
| CN1C[C@H](CO)C=C2c3cccc4[nH]cc(c34)C[C@H]21 | 0.591606325 |
| COC(=O)/C=C/c1ccc(O)cc1 | 0.606645153 |
| O=Cc1ccc(O)c(O)c1 | 0.527697966 |
| CC(C)=CCC[C@@](C)(O)[C@H]1CC[C@@]2(C)[C@@H]1[C@H](O)C[C@@H]1[C@@]3(C)CC[C@H](O)C(C)(C)[C@@H]3CC[C@]12C | 0.902978236 |
| COC(=O)c1ccccc1N | 0.573395019 |
| COc1ccc(-c2cc(=O)c3c(O)c(OC)c(O[C@@H]4O[C@H](C(=O)O)[C@@H](O)[C@H](O)[C@H]4O)cc3o2)cc1 | 0.510146935 |
| CC(=O)O[C@@]1(C)C[C@@H](O)[C@]2(O)C=CO[C@@H](O[C@@H]3O[C@H](CO)[C@@H](O)[C@H](O)[C@H]3O)[C@@H]21 | 0.695998987 |
| Oc1cc2c3c(c1)c1cc4c(cc1c[n+]3CC2)OCO4 | 0.493428028 |
| COc1cc2ccc(=O)oc2cc1OC(C)=O | 0.654774852 |
| O=Cc1cc(O)ccc1O | 0.526773265 |
| Cc1cc(O[C@@H]2O[C@H](CO)[C@@H](O)[C@H](O)[C@H]2O)c2c(c1)C(=O)c1cccc(O)c1C2=O | 0.524845275 |
| COC(=O)c1cc(O)c(O)c(O)c1 | 0.561718893 |
| C=C1[C@H]2C[C@H]2[C@]2(C)Cc3occ(C)c3[C@H](O)[C@@H]12 | 0.537249938 |
| CC1(C)C=Cc2c(ccc(C(=O)/C=C/c3ccc(O)cc3)c2O)O1 | 0.582912484 |
| CC(C)(O)[C@H](O)COc1c2occc2cc2ccc(=O)oc12 | 0.673389947 |
| Cc1ccc(C(C)C)c(O)c1 | 0.563676776 |
| COc1cc(O)c2c(=O)c(O[C@@H]3O[C@H](CO[C@@H]4O[C@@H](C)[C@H](O)[C@@H](O)[C@H]4O)[C@@H](O)[C@H](O)[C@H]3O)c(-c3ccc(OC)c(O)c3)oc2c1 | 0.399007572 |
| OC/C=C/C#CC#C/C=C/CCC(CCO)O[C@@H]1O[C@H](CO)[C@@H](O)[C@H](O)[C@H]1O[C@@H]1OC[C@H](O)[C@H](O)[C@H]1O | 0.460011522 |
| COc1cc(O)cc(O)c1C(C)=O | 0.515115167 |
| CC(C)=CCC[C@@H](C)c1ccc(C)c(O)c1 | 0.623425255 |
| C/C=C/c1cc(OC)c2c(c1)C(C)C(c1ccc(O)c(OC)c1)O2 | 0.491614872 |
| COc1ccc2[nH]c(=O)oc2c1 | 0.562210634 |
| COc1ccc(-c2cc(=O)c3c(O)c(OC)c(O[C@@H]4O[C@H](CO)[C@@H](O)[C@H](O)[C@H]4O)cc3o2)cc1 | 0.512908775 |
| CC(=O)Oc1cc(OC(C)=O)c2c(c1)O[C@H](c1cc(OC(C)=O)c(OC(C)=O)c(OC(C)=O)c1)[C@H](OC(=O)c1cc(OC(C)=O)c(OC(C)=O)c(OC(C)=O)c1)C2 | 0.397019791 |
| COC(=O)[C@@]1(C)CC[C@]2(C(=O)O)CC[C@]3(C)C(=CC[C@@H]4[C@@]5(C)C[C@H](O)[C@H](O)[C@@](C)(CO)[C@@H]5CC[C@]43C)[C@@H]2C1 | 0.683792732 |
| CC1(C)CC[C@]2(C(=O)O)CC[C@]3(C)C(=CC[C@@H]4[C@@]5(C)C[C@@H](O)[C@H](O)C(C)(C)[C@@H]5CC[C@]43C)[C@@H]2C1 | 0.889286259 |
| O=C(O)/C=C/c1ccccc1 | 0.583696287 |
| O=C(/C=C/c1ccc(O)c(O)c1)OC1CC(O)(C(=O)O)CC(O)C1O | 0.61575665 |
| O=c1c(O)c(-c2cc(O)c(O)c(O)c2)oc2cc(O)ccc12 | 0.629947252 |
| CC1=CC[C@H]2C[C@@H]1C2(C)C | 0.546273027 |
| C/C=C/c1ccc(O)c(OC)c1 | 0.518591003 |
| CCCC/C=C\CCCCCCCc1cc(=O)c2ccccc2n1C | 0.524773583 |
| COc1ccc(/C=C/C(=O)N2CCCC2)cc1 | 0.644249753 |
| COc1cc2oc(-c3ccccc3)cc(=O)c2c(O)c1OC | 0.56941601 |
| C=C1C(=O)C23C(O)C1CCC2C12COC3(O)C(O)C1C(C)(C)CCC2O | 0.745299593 |
| COc1ccc(-c2coc3cc(O[C@@H]4O[C@H](CO)[C@@H](O)[C@H](O)[C@H]4O)ccc3c2=O)cc1 | 0.619724159 |
| COc1ccc(C(=O)O)cc1 | 0.56734107 |
| CCCCCCCCCCCCCCCc1cccc(O)c1C(=O)O | 0.531230527 |
| O=C(/C=C/c1ccc(O)cc1)OC[C@H]1O[C@@H](Oc2c(-c3ccc(O)cc3)oc3cc(O)cc(O)c3c2=O)[C@H](O)[C@@H](O)[C@@H]1O | 0.451902462 |
| C=C1CCC2CC1C2(C)C | 0.533274357 |
| COc1c(-c2ccccc2)oc2cc(O)cc(O)c2c1=O | 0.619089997 |
| CCCCCCCCCCCCCCO | 0.569497009 |
| CC(C)=CCCc1ccoc1 | 0.55266723 |
| C=C(C(=O)O)[C@@H]1CC[C@@H](C)[C@@H]2CCC(C)=C[C@@H]21 | 0.67314859 |
| CCCCCCCCCCCCCCCCCCCCCCCCCCCCC | 0.497730128 |
| CC1(C)CC[C@]2(C(=O)O)CC[C@]3(C)C(=CC[C@@H]4[C@@]5(C)C[C@H](O)[C@H](O)[C@@](C)(C(=O)O)[C@@H]5CC[C@]43C)[C@@H]2C1 | 0.778200538 |
| C=C1CC[C@]2(C)CC[C@]3(C)[C@H](CC[C@@H]4[C@@]5(C)CC[C@H](OC(C)=O)C(C)(C)[C@@H]5CC[C@]43C)[C@H]2[C@@H]1C | 0.892660263 |
| CCOC(=O)CC(C)=O | 0.617038884 |
| CC1(C)CCC[C@]2(C)[C@H]3CC(=O)O[C@]3(C)CC[C@@H]12 | 0.674023841 |
| COc1cc2c(=O)c(-c3ccc(O)cc3)coc2cc1OC1OC(CO)C(O)C(O)C1O | 0.566561671 |
| O=C1C=C2C=C[C@H]3C[C@]2(O1)[C@H]1CCCCN31 | 0.608045473 |
| CCCCCCCCCCCCCCCCCO | 0.547001659 |
| CC1=C[C@H](O)CC(C)(C)[C@H]1/C=C/C(C)=C/C=C/C(C)=C/C=C/C=C(C)/C=C/C=C(C)/C=C/C1=C(C)C[C@@H](O)CC1(C)C | 0.549087145 |
| c1cc2c(cc1[C@H]1OC[C@@H]3[C@H]1CO[C@H]3c1ccc3c(c1)OCO3)OCO2 | 0.469910411 |
| COc1cc(O)c2c(O)c3c(=O)oc(C)cc3cc2c1 | 0.655429453 |
| OC[C@H]1O[C@@](CO)(OC[C@@]2(O[C@H]3O[C@H](CO)[C@@H](O)[C@H](O)[C@H]3O)O[C@H](CO)[C@@H](O)[C@@H]2O)[C@@H](O)[C@@H]1O | 0.508529624 |
| O=C(O)/C=C\c1ccc2occc2c1O[C@@H]1O[C@H](CO)[C@@H](O)[C@H](O)[C@H]1O | 0.719935489 |
| COC1=CC(=O)O[C@@H](/C=C/c2ccccc2)C1 | 0.693406319 |
| CCCCCCCCCCCCCCCCCCCC(=O)OC | 0.539503195 |
| O=CCCCCCCCC(=O)O | 0.563520854 |
| CCc1c2c(nc3ccccc13)-c1cc3c(c(=O)n1C2)COC(=O)[C@]3(O)CC | 0.57046594 |
| CC[C@@]1(O)C(=O)OCc2c1cc1n(c2=O)Cc2cc3cc(OC)ccc3nc2-1 | 0.586398104 |
| COc1ccc(C[C@H]2COC(=O)[C@]2(O)Cc2ccc(O[C@@H]3O[C@H](CO)[C@@H](O)[C@H](O)[C@H]3O)c(OC)c2)cc1OC | 0.469303305 |
| O=C(O)c1ccc2c(c1)C(=O)c1ccccc1C2=O | 0.670609548 |
| COC(=O)/C=C/c1ccc(O[C@@H]2O[C@H](CO)[C@@H](O)[C@H](O)[C@H]2O)cc1 | 0.707186656 |
| COc1cc(O)cc2oc(-c3ccc(O)c(O)c3)c(O)c(=O)c12 | 0.65236081 |
| CC1(C)CC[C@@]2(CO)C(=C3C=C[C@@H]4[C@@]5(C)CC[C@H](O)[C@@](C)(CO)[C@@H]5CC[C@@]4(C)[C@]3(C)C[C@@H]2O)C1 | 0.862911899 |
| Oc1cc(O)c2c(c1)O[C@H](c1ccc(O)c(O)c1)[C@@H](O)[C@@H]2c1c(O)cc(O)c2c1O[C@H](c1ccc(O)c(O)c1)[C@@H](O)C2 | 0.438105926 |
| Nc1ncc2[nH]cnc2n1 | 0.608031127 |
| C/C1=C\CCC2=C[C@@H](OC2=O)c2c(C)coc2C1 | 0.628752488 |
| COC(=O)c1c2c(c3ccccc3c1O)OC(C)(C)C=C2 | 0.615042173 |
| C=C1C[C@@]2(O)O[C@@]3(C[C@H]2C(C)C)[C@@H](C)CC[C@@H]13 | 0.660492408 |
| COc1cc([C@H]2c3cc(O)c(OC)cc3C[C@@H](CO)[C@@H]2CO)ccc1O | 0.562750933 |
| O=C(/C=C/c1ccc(O)c(O)c1)NCCc1ccc(O)cc1 | 0.728207966 |
| COc1c(O)cc2c(c1O)[C@@H]1O[C@H](COC(=O)c3cc(O)c(O)c(O)c3)[C@@H](O)[C@H](O)[C@H]1OC2=O | 0.511141075 |
| O=C(OC[C@H]1O[C@@H](OC(=O)c2cc(O)c(O)c(O)c2)[C@H](O)[C@@H](OC(=O)c2cc(O)c(O)c(O)c2)[C@@H]1O)c1cc(O)c(O)c(O)c1 | 0.460873927 |
| C=C1CC[C@@]2(C)[C@H]([C@@H]1C)[C@H]1CC[C@@H]3[C@@]4(C)CC[C@H](O)C(C)(C)[C@@H]4CC[C@@]3(C)[C@]1(C)C[C@@H]2O | 0.912179803 |
| CC1(C)C=Cc2c(ccc3ccc(=O)oc23)O1 | 0.595989375 |
| C[C@H]1[C@H](C)CC[C@]2(C(=O)O)CC[C@]3(C)C(=CC[C@@H]4[C@@]5(C)CC[C@@H](O)C(C)(C)[C@@H]5CC[C@]43C)[C@H]12 | 0.886060644 |
| CC1CCC2(C(=O)O)CCC3(C)C(=CCC4C5(C)CC(O)C(O)C(C)(C)C5CCC43C)C2C1(C)O | 0.87042676 |
| CC1(C)O[C@@H]1COc1c2occc2cc2ccc(=O)oc12 | 0.644787287 |
| O=C(C=Cc1ccccc1)OCc1ccccc1 | 0.722710024 |
| C=C(CC[C@@H](C(=O)O)[C@H]1[C@H](O)C[C@@]2(C)C3=CC[C@H]4C(C)(C)[C@@H](O)CC[C@]4(C)C3=CC[C@]12C)C(C)C | 0.827959837 |
| O=c1c(O[C@@H]2O[C@H](CO)[C@H](O)[C@H](O)[C@H]2O)c(-c2ccc(O)cc2)oc2cc(O)cc(O)c12 | 0.551009807 |
| CC(=O)O[C@H]1CC[C@]2(C)C3=C(CC[C@H]2C1(C)C)[C@]1(C)C[C@@H](O)[C@H]([C@@H](CCC=C(C)C)C(=O)O)[C@@]1(C)CC3 | 0.75051038 |
| CC(CCC(O)C(C)(C)O)C1CCC2(C)C3CC=C4C(CCC(O)C4(C)C)C3(C)C(=O)CC12C | 0.8818963 |
| C[C@@H]1CC[C@@H]2C(C(=O)O)=CO[C@@H](O[C@@H]3O[C@H](CO)[C@@H](O)[C@H](O)[C@H]3O)[C@H]12 | 0.735469597 |
| O=c1c(O[C@@H]2O[C@H](CO)[C@@H](O)[C@H](O)[C@H]2O)c(-c2cc(O)c(O)c(O)c2)oc2cc(O)cc(O)c12 | 0.483598466 |
| COc1ccc(-c2oc3cc(O)cc(O)c3c(=O)c2O)cc1O | 0.63617759 |
| COc1cc([C@H]2OC[C@H]3[C@@H]2CO[C@@H]3c2cc(OC)c(O[C@@H]3O[C@H](CO)[C@@H](O)[C@H](O)[C@H]3O)c(OC)c2)cc(OC)c1O | 0.447984749 |
| COc1cc(C(=O)O)cc(OC)c1O[C@@H]1O[C@H](CO)[C@@H](O)[C@H](O)[C@H]1O | 0.683326639 |
| CC1(C)[C@H]2CC[C@]1(C)[C@@H](O)C2 | 0.512554996 |
| CC(C)CCC[C@@H](C)[C@H]1CC[C@H]2C3=CC[C@H]4C[C@H](O)CC[C@]4(C)[C@H]3CC[C@]12C | 0.779785154 |
| CC(=O)N[C@@H](CCCCN)C(=O)O | 0.637736276 |
| Oc1ncnc2[nH]cnc12 | 0.588060292 |
| O=C(O)/C=C(\CC(=O)O)C(=O)O | 0.609969952 |
| C/C=C/CC1=C(C)CC(=O)C1C(=O)OC | 0.596076096 |
| CC1(C)C2CC[C@]3(C)[C@H](C(=O)C=C4[C@@H]5C[C@@](C)(C(=O)O)CC[C@]5(C)CC[C@]43C)[C@@]2(C)CC[C@@H]1O | 0.789223864 |
| O=C1O[C@H]([C@@H](O)CO)C(=O)C1=O | 0.57437235 |
| Cc1cnc(C)cn1 | 0.623941317 |
| O=C(O)Cc1cc(O)ccc1O | 0.575327504 |
| C=C1CC/C=C(/C)CC[C@@H]2[C@@H]1CC2(C)C | 0.615622349 |
| O=C[C@H](O)[C@@H](O)[C@H](O)CO | 0.638577352 |
| CC=Cc1ccc(OC)cc1 | 0.557174165 |
| O=C(O)CCc1ccc(O)c(O)c1 | 0.558672295 |
| O=C(O)C(=O)O | 0.612160769 |
| CC(C)CCC[C@@H](C)[C@H]1CC[C@H]2[C@@H]3CC[C@H]4C[C@@H](O)CC[C@]4(C)[C@H]3CC[C@]12C | 0.811528389 |
| O=C(O)/C=C/c1cccc(O)c1 | 0.581674722 |
| C[S+]([O-])CCCN=C=S | 0.609025018 |
| O=C(O)/C=C/CCCCCCCO | 0.616840569 |
| CCCCCCCCCCCCCCCCC(=O)O | 0.59465656 |
| O=C(O)C1=CO[C@@H](O[C@@H]2O[C@H](CO)[C@@H](O)[C@H](O)[C@H]2O)[C@@H]2C(CO)=CC[C@H]12 | 0.650701559 |
| OC[C@H]1O[C@@](CO)(O[C@H]2O[C@H](CO)[C@@H](O)[C@H](O)[C@H]2O)[C@@H](O)[C@@H]1O | 0.63232109 |
| C[C@H](CCC(=O)O)[C@H]1CC[C@H]2[C@@H]3[C@H](O)C[C@@H]4C[C@H](O)CC[C@]4(C)[C@H]3C[C@H](O)[C@]12C | 0.78562712 |
| CCCCCC/C=C/C=C\CCCCCCCC(=O)O | 0.535338961 |
| C=C(C)C1CC=C(CO)CC1 | 0.552081478 |
| CCCCCCCCCCCCCCCCCCCCCCC(=O)O | 0.545369039 |
| Cn1c(=O)[nH]c2nc[nH]c2c1=O | 0.593237773 |
| O.OC[C@H]1O[C@H](OC[C@H]2O[C@@H](O)[C@H](O)[C@@H](O)[C@@H]2O)[C@H](O)[C@@H](O)[C@H]1O | 0.657225157 |
| C[N+](C)(C)CC(=O)O.[Cl-] | 0.550374995 |
| CC(=O)c1ccc(C)cc1C | 0.55020587 |
| C=C(C)C1CC=C(C)C(O)C1 | 0.557614883 |
| O=Cc1c[nH]c2ccccc12 | 0.564042929 |
| O=C(O)c1c(O)cccc1O | 0.561862612 |
| O=C(O)c1ccc(O)cc1O | 0.551920716 |
| CCCCCCCC/C=C/CCCCCCCC(=O)O | 0.572160088 |
| CSC[C@H]1O[C@@H](n2cnc3c(N)ncnc32)[C@H](O)[C@@H]1O | 0.708966797 |
| CCCCCCCC/C=C\CCCCCCCCCCCCCC(=O)O | 0.544837926 |
| OCCc1ccc(O)cc1 | 0.5662178 |
| O=C(O)C=Cc1ccccc1 | 0.583696287 |
| O=c1cc(-c2ccccc2)oc2ccccc12 | 0.604770909 |
| c1ccc2[nH]ccc2c1 | 0.590210676 |
| CC1(C)O[C@H]2CC(=O)OC[C@]23[C@H]2CC[C@@]4(C)[C@H](c5ccoc5)OC(=O)[C@H]5O[C@]54[C@]2(C)C(=O)C[C@@H]13 | 0.641977705 |
| NCc1ccc(O)cc1 | 0.563798459 |
| O=C(O)CCCCC1CCSS1 | 0.682376452 |
| O=C(O)Cc1ccccc1O | 0.597491885 |
| CC(=O)CCC=C(C)C | 0.608476437 |
| O=C(O)CCCCCCC(=O)O | 0.575072911 |
| O=C(/C=C/c1ccc(O)c(O)c1)O[C@@H]1CC(O)(C(=O)O)C[C@@H](OC(=O)/C=C/c2ccc(O)c(O)c2)C1O | 0.48068717 |
| CC12CCC(=O)C=C1CCC1C2CCC2(C)C1CC[C@]2(O)C(=O)CO | 0.593417401 |
| CC(C(=O)O)C(=O)O | 0.59915159 |
| OC[C@H]1O[C@H](O[C@H]2[C@H](O)[C@H](O)COC2(O)CO)[C@H](O)[C@@H](O)[C@@H]1O | 0.645974418 |
| Nc1ncnc2c1ncn2[C@H]1C[C@H](O)[C@@H](CO)O1.O | 0.69032502 |
| OC[C@@H](O)[C@@H](O)CO | 0.572832525 |
| OCc1ccc(O)cc1 | 0.574369631 |
| COc1cc(C=O)ccc1O | 0.506048849 |
| NC(=O)NCCCCC(N)C(=O)O | 0.610005665 |
| CC(=O)[C@H]1CC[C@H]2[C@@H]3CCC4=CC(=O)CC[C@]4(C)[C@H]3CC[C@]12C | 0.627918382 |
| C=CCCC(=O)O | 0.655925532 |
| Oc1ccc([C@H]2COc3cc(O)ccc3C2)cc1 | 0.517305382 |
| O=C(/C=C/c1ccc(O)c(O)c1)O[C@H]1[C@H](O)C[C@](O)(C(=O)O)C[C@H]1OC(=O)/C=C/c1ccc(O)c(O)c1 | 0.46618018 |
| CCCC(C)C(=O)O | 0.632385377 |
| CCCCCCCCCCCCCCCCCCCCCCCCC(=O)O | 0.537179724 |
| C[C@]12CC[C@@H]3c4ccc(O)cc4CC[C@H]3[C@@H]1CCC2=O | 0.574660181 |
| OC[C@@H](O)[C@@H](O)[C@H](O)[C@@H](O)CO | 0.575057934 |
| Oc1cc(O)c2c(c1)O[C@H](c1ccc(O)c(O)c1)[C@H](O)C2 | 0.597670335 |
| CCC(C)CC(=O)O | 0.63241568 |
| CCCCCCCCCCC/C=C\CCCCC(=O)O | 0.544119772 |
| CC(C)CCC[C@@H](C)[C@H]1CC[C@H]2[C@@H]3CC[C@H]4CCCC[C@]4(C)[C@H]3CC[C@]12C | 0.794716846 |
| CCCCCCCC/C=C\CCCCCCCC(=O)OCC(COC(=O)CCCCCCC/C=C\CCCCCCCC)OC(=O)CCCCCCC/C=C\CCCCCCCC | 0.42922323 |
| CCCCC(CC)COC(=O)c1ccccc1C(=O)O | 0.599520886 |
| C=CC1=C(C)c2cc3[nH]c(cc4nc(cc5[nH]c(cc1n2)c(C)c5C=C)C(C)=C4CCC(=O)O)c(CCC(=O)O)c3C | 0.454350793 |
| COc1ccccc1O | 0.531795642 |
| CC(C)=CCCC(C)C1CCC2(C)C3=C(CCC12C)C1(C)CCC(O)C(C)(C)C1CC3 | 0.815143274 |
| O=C(O)CCc1ccccc1 | 0.625534055 |
| CCC=CCC=CCC=CCC=CCC=CCC=CCCC(=O)O | 0.508375905 |
| CC1=CC(=O)c2ccccc2C1=O | 0.470809483 |
| CC1OC(OC2C(OC3CC4(C)C(CC(O)C5C(C6(C)CCC(C(C)(C)O)O6)CCC54C)C4(C)CCC(O)C(C)(C)C34)OC(CO)C(O)C2O)C(O)C(O)C1O | 0.792906826 |
| C[C@H]1[C@H](C)CC[C@]2(C(=O)O)CC[C@]3(C)C(=CC[C@@H]4[C@@]5(C)CCC(=O)C(C)(C)[C@@H]5CC[C@]43C)[C@H]12 | 0.805334982 |
| Nc1ccn([C@@H]2O[C@H](CO)[C@@H](O)[C@H]2O)c(=O)n1 | 0.667053991 |
| Cc1ccc(C(=O)O)cc1 | 0.598396909 |
| O=c1c(O)c(-c2ccccc2)oc2ccccc12 | 0.560883664 |
| CNC(=O)c1cccnc1 | 0.67238866 |
| NC(Cc1c[nH]c2ccc(O)cc12)C(=O)O | 0.751685526 |
| CC(=O)NCCc1c[nH]c2ccc(O)cc12 | 0.66695157 |
| C[N+]1(C)CCC[C@H]1C(=O)[O-] | 0.5409845 |
| CCCCCCCC/C=C\CCCCCCCCCCCC(=O)O | 0.558314489 |
| C[n+]1cccc(C(=O)[O-])c1 | 0.575016957 |
| O=C(O)CC(O)C(=O)O | 0.592842457 |
| O=C(O)C1=CC(O)C(O)C(O)C1 | 0.611949684 |
| CC(C)=CCC[C@@H](C)CCO | 0.617720567 |
| COc1ccc(C=O)cc1 | 0.527752613 |
| CCCCCCCC/C=C\CCCCCCCC(=O)OCC(O)CO | 0.527444441 |
| O=C1O[C@H]([C@@H](O)CO)C(O)=C1O | 0.635844987 |
| CC(C)CC(=O)O | 0.633896391 |
| O=C(O)CCc1ccc(O)cc1 | 0.595087471 |
| CC(C)[C@@H](C)/C=C/[C@@H](C)[C@H]1CC[C@H]2C3=CC=C4C[C@@H](O)CC[C@]4(C)[C@H]3CC[C@]12C | 0.701635212 |
| CCCCCCCCCCCCCCCCCC=O | 0.545840611 |
| COc1ccc(CC(=O)O)cc1OC | 0.615439464 |
| COc1ccc(CC(=O)O)cc1 | 0.595986702 |
| C=C1CC[C@H]2O[C@]2(C)CC[C@@H]2[C@@H]1CC2(C)C | 0.604130438 |
| O=C(O)Cc1ccc(O)cc1 | 0.608928574 |
| O=C(O)C=Cc1cccc(O)c1 | 0.581674722 |
| O=C(O)c1cc(O)cc(O)c1 | 0.581746974 |
| CCCCCC=CCC=CCCCCCCCC(=O)OCC(COC(=O)CCCCCCCC=CCC=CCCCCC)OC(=O)CCCCCCCC=CCC=CCCCCC | 0.356534428 |
| O=C(O)CCCCCCCCCCCC(=O)O | 0.586662583 |
| COc1cc(C=CC(=O)O)ccc1O | 0.560835475 |
| CCc1ccc(O)cc1 | 0.617996734 |
| CC(CCC(=O)O)CC(=O)O | 0.58384774 |
| CC(C)=CCC[C@H](C)CC=O | 0.60220865 |
| CC(C)=CCC/C(C)=C/CC/C(C)=C/CC[C@]1(C)CCc2c(C)c(O)c(C)c(C)c2O1 | 0.544112034 |
| CC1(O)CCOC(=O)C1 | 0.57460692 |
| COC(=O)c1ccc(O)c(OC)c1 | 0.57095938 |
| CCCCCCCCCCCCCCCCCC(=O)O | 0.56851645 |
| COc1cccc(C(=O)O)c1 | 0.565346061 |
| O=C(/C=C/c1ccc(O)c(O)c1)O[C@H]1[C@H](O)C[C@@](O)(C(=O)O)C[C@H]1O | 0.616991408 |
| CC1C(=O)OC2C(O)C34C5CC(C(C)(C)C)C36C(OC(=O)C6O)OC4(C(=O)O5)C12O | 0.642386282 |
| C/C=C/C(=O)C1=C(C)C=CCC1(C)C | 0.581997535 |
| Cn1cnc(C[C@H](N)C(=O)O)c1 | 0.650832855 |
| CCCCCCCCCCCCCCCC(=O)O | 0.591395885 |
| Cc1cc(O)cc2c1OC(C)(CCCC(C)CCCC(C)CCCC(C)C)CC2 | 0.603485611 |
| CCCCCCCCCCCCCCCCCCCCCCCCCCC(=O)O | 0.53875224 |
| OC[C@H]1O[C@H](O)[C@H](O)[C@@H](O)[C@@H]1O | 0.587858967 |
| COc1cc(CC(=O)O)ccc1O | 0.56073589 |
| O=C(O)c1ccoc1 | 0.622237279 |
| O=C1C[C@@H](c2ccc(O)c(O)c2)Oc2cc(O)cc(O)c21 | 0.629506202 |
| CC(=CCNc1ncnc2[nH]cnc12)CO | 0.712693623 |
| O=C(O)C(=O)c1ccccc1 | 0.557476614 |
| O=C(O)c1cc(O)ccc1O | 0.5673708 |
| O=c1ccn([C@@H]2O[C@H](COP(=O)(O)O)[C@@H](O)[C@H]2O)c(=O)[nH]1 | 0.679260029 |
| OCCc1c[nH]c2ccccc12 | 0.543463066 |
| O=C(CCc1ccc(O)cc1)c1c(O)cc(O)cc1O | 0.693699242 |
| Cc1ccc(C(C)C)cc2c(C)ccc1-2 | 0.52939778 |
| CC(=O)NC(Cc1c[nH]c2ccccc12)C(=O)O | 0.669287119 |
| CC12CCC3c4ccc(O)cc4CCC3C1CC(O)C2O | 0.542960787 |
| COc1cc(C=CC(=O)O)cc(OC)c1OC | 0.689726514 |
| C=C(C)C1CC=C(/C=N/O)CC1 | 0.537973382 |
| CN1c2c([nH]c(N)nc2=O)NCC1CNc1ccc(C(=O)N[C@@H](CCC(=O)O)C(=O)O)cc1 | 0.485732654 |
| CCCCC/C=C/C/C=C/CCCCCCCC(=O)O[C@H](COC(=O)CCCCCCCCCCCCCCC)COP(=O)([O-])OCC[N+](C)(C)C | 0.437763698 |
| CC(C)(C)c1ccc(O)c(C(C)(C)C)c1 | 0.584713538 |
| O=C(O)CCC(=O)O | 0.632686169 |
| CCCCCC=CCC=CCCCCCCCCCC(=O)O | 0.51272952 |
| CCCCCCCCCCCCCC(=O)O | 0.64116828 |
| COc1cc(-c2oc3cc(O)cc(O)c3c(=O)c2O)ccc1O | 0.637045645 |
| COC(=O)Cc1c[nH]c2ccccc12 | 0.629711879 |
| Cc1occc(=O)c1O | 0.596128631 |
| CC(=O)CCC(=O)O | 0.599329083 |
| CCCCCCCCCCCCCCCCCCO | 0.533087181 |
| CC(=O)N[C@@H](C=O)[C@@H](O)[C@H](O)[C@H](O)CO | 0.623595141 |
| Oc1cc(O)c2c(c1)O[C@H](c1ccc(O)c(O)c1)[C@@H](O)C2 | 0.597670335 |
| O=C(O)c1c[nH]c2ccccc12 | 0.565703146 |
| C[C@@H]1C(=O)O[C@H]2C[C@@]34[C@H]5C[C@@H](C(C)(C)C)[C@]36[C@@H](OC(=O)[C@@H]6O)O[C@@]4(C(=O)O5)[C@]21O | 0.646528535 |
| Cc1ncc(C[n+]2csc(CCO)c2C)c(N)n1.Cl.[Cl-] | 0.665496651 |
| COC(=O)CCc1ccccc1 | 0.624338032 |
| Cc1ccc(C=O)c(O)c1 | 0.549346058 |
| Cc1cc2nc3[nH]c(=O)[nH]c(=O)c3nc2cc1C | 0.621809915 |
| O=C(O)[C@@H](O)[C@H](O)[C@H](O)[C@@H](O)C(=O)O | 0.613088779 |
| CC12CCC3c4ccc(O)cc4CCC3C1CCC2O | 0.51986078 |
| O=c1ccc2ccc(O)cc2o1 | 0.509760573 |
| C#C[C@]1(O)CC[C@H]2[C@@H]3CCc4cc(O)ccc4[C@H]3CC[C@@]21C | 0.544687718 |
| Cc1cn([C@H]2C[C@H](O)[C@@H](CO)O2)c(=O)[nH]c1=O | 0.672874139 |
| NCCCNCCCCNCCCN | 0.622072387 |
| O=C(O)C1(O)CC(O)C(O)C(O)C1 | 0.601758423 |
| O=C(O)c1cc(O)c(O)c(O)c1 | 0.560804099 |
| CC(=O)OC1CCC2(C)C(CCC3(C)C2C(=O)C=C2C4C(C)C(C)CCC4(C)CCC23C)C1(C)C(=O)O | 0.763077768 |
| OC[C@H]1O[C@@H](O)[C@H](O)[C@H](O)[C@@H]1O | 0.587858967 |
| O=P(O)(O)O[C@H]1[C@H](OP(=O)(O)O)[C@@H](OP(=O)(O)O)[C@H](OP(=O)(O)O)[C@@H](OP(=O)(O)O)[C@H]1OP(=O)(O)O | 0.486008612 |
| O=Cc1ccc(O)cc1O | 0.514108955 |
| C[C@H](CCC(=O)NCCS(=O)(=O)O)[C@H]1CCC2C3C(CC[C@@]21C)[C@@]1(C)CC[C@@H](O)C[C@H]1C[C@@H]3O | 0.779654709 |
| CCCCCC/C=C/CCCCCCCCCC(=O)O | 0.572160088 |
| O=C(O)c1ccc(CO)o1 | 0.630796532 |
| CC(CCC(=O)O)C1CCC2C3C(O)CC4CC(O)CCC4(C)C3CCC12C | 0.760349915 |
| CCCCCCCCCCCCCCCC(=O)OCC(COC(=O)CCCCCCCCCCCCCCC)OC(=O)CCCCCCCCCCCCCCC | 0.440669981 |
| CC[C@H](CC[C@@H](C)[C@H]1CC[C@H]2[C@@H]3CC[C@H]4C[C@@H](O)CC[C@]4(C)[C@H]3CC[C@]12C)C(C)C | 0.822624714 |
| COc1ccc(/C=C/C(=O)O)cc1 | 0.575784522 |
| O=C(O)CC(=O)C(=O)O | 0.58942272 |
| Cc1cc2nc3c(=O)[nH]c(=O)nc-3n(CC(O)C(O)C(O)CO)c2cc1C | 0.580737778 |
| CCCCCC/C=C\CCCCCCCC(=O)O | 0.590570218 |
| CCCCCCCC/C=C\CCCCCCCCC(=O)O | 0.565670614 |
| O=C(O)[C@@H](O)Cc1ccccc1 | 0.636011142 |
| O=c1[nH]c(=O)c2ncn([C@@H]3O[C@H](CO)[C@@H](O)[C@H]3O)c2[nH]1 | 0.634222379 |
| CO[C@@H]1[C@H](O)[C@@H](CO)O[C@H]1n1cnc2c(N)ncnc21 | 0.646194014 |
| COc1cc(CCC(=O)O)cc(OC)c1OC | 0.705290626 |
| O=C(O)CCCCCCCCCCCCCCC(=O)O | 0.51353402 |
| CC(C)=CCCC(C)(O)C1CCC2(C)C1C(O)CC1C3(C)CCC(OC4OC(CO)C(O)C(O)C4O)C(C)(C)C3CCC12C | 0.888247923 |
| CC(=O)c1ccccc1C | 0.551715681 |
| CC(=O)C=Cc1ccccc1 | 0.600064724 |
| O=C(O)C[C@H](O)C(=O)O | 0.592842457 |
| O=C(O)c1cccc(O)c1 | 0.575006374 |
| NC(=O)c1ccccc1 | 0.610663994 |
| C=Cc1ccc(O)cc1 | 0.600839282 |
| CCCCCCCCCCCCCCCCCCCCCC(=O)O | 0.549079977 |
| O=Cc1ccc(O)cc1 | 0.550253189 |
| CC(=O)c1ccc(O)cc1O | 0.513652661 |
| O=C(C=Cc1ccc(O)c(O)c1)O[C@@H]1[C@H](O)C[C@@](O)(C(=O)O)C[C@H]1OC(=O)C=Cc1ccc(O)c(O)c1 | 0.46618018 |
| OCC1O[C@H](OCC2OC(O)C(O)[C@@H](O)[C@@H]2O)C(O)[C@@H](O)[C@@H]1O | 0.664760679 |
| CCCCC/C=C/C(=O)O | 0.634399025 |
| CC(C)=CCC[C@@H](C)[C@H]1CC[C@@]2(C)C3=C(CC[C@]12C)[C@@]1(C)CC[C@H](O)C(C)(C)[C@@H]1CC3 | 0.815143274 |
| CC(C)c1ccc(C=O)cc1 | 0.586716996 |
| CC[C@H](CC[C@@H](C)[C@H]1CC[C@H]2[C@@H]3CC=C4C[C@@H](O)CC[C@]4(C)[C@H]3CC[C@]12C)C(C)C | 0.840120619 |
| C[C@]12CC[C@H]3[C@@H](CCC4=CC(=O)CC[C@@]43C)[C@@H]1CC[C@@H]2C(=O)CO | 0.583610956 |
| OC[C@@H](O)[C@H](O)[C@H](O)[C@@H](O)CO | 0.575057934 |
| O=C(O)C1CC(O)CN1 | 0.62639115 |
| NC1CCCNC1=O | 0.643234623 |
| CC(=O)Oc1c(C)c(C)c2c(c1C)CC[C@@](C)(CCC[C@H](C)CCC[C@H](C)CCCC(C)C)O2 | 0.605591615 |
| O=C(O)/C=C(/CC(=O)O)C(=O)O | 0.609969952 |
| N#CCc1ccc(O)cc1 | 0.565814123 |
| C=C(C)[C@@H]1CC=C(C)CC1 | 0.550331078 |
| CC1=CC(=O)CC(C)(C)[C@@]1(O)/C=C/C(C)=C\C(=O)O | 0.628178032 |
| COc1cc(C)ccc1O | 0.510231902 |
| COc1cc(/C=C/C(=O)O)ccc1O | 0.560835475 |
| OCC(O)C(O)C(O)CO | 0.581867809 |
| COC(=O)c1ccco1 | 0.606705509 |
| Cc1cn([C@@H]2O[C@H](CO)[C@@H](O)[C@H]2O)c(=O)[nH]c1=O | 0.635270079 |
| O=c1ccc2cc3ccoc3c(O)c2o1 | 0.479332101 |
| Nc1nc(=O)c2ncn([C@H]3C[C@H](O)[C@@H](CO)O3)c2[nH]1 | 0.638309145 |
| CCCCCCCCCCCCCCCCCCCCCCCC(=O)O | 0.537014004 |
| O=C(O)c1cccc(O)c1O | 0.553845205 |
| O=C(O)CCCCCCCCCCCCCCCCC(=O)O | 0.47419029 |
| CCCCCCCCCCCCCCCCCCCC(=O)O | 0.550946214 |
| CCCCC/C=C\C/C=C\C/C=C\CCCCC(=O)O | 0.517888155 |
| O=C(O)CCC(=O)C(=O)O | 0.591106972 |
| N#CCc1c[nH]c2ccccc12 | 0.522659531 |
| O=C(O)c1ccc[nH]1 | 0.652767961 |
| CCCCC/C=C\C/C=C\C/C=C\C/C=C\CCCC(=O)O | 0.483227743 |
| O=C(O)c1ccc(C(=O)O)cc1 | 0.591752014 |
| N[C@@H](CCO)C(=O)O | 0.635581236 |
| CC/C=C\C/C=C\C/C=C\C/C=C\C/C=C\CCCC(=O)O | 0.491319261 |
| CCCCCCCCCCCCCCC(=O)O | 0.595540374 |
| CCCCCCCC/C=C\CCCCCCCC(=O)O | 0.572160088 |
| O=C(O)[C@H]1O[C@@H](Oc2c(-c3ccc(O)c(O)c3)oc3cc(O)cc(O)c3c2=O)[C@H](O)[C@@H](O)[C@@H]1O | 0.50306829 |
| Cc1c[nH]c(=O)[nH]c1=O | 0.658181322 |
| O=c1ccn([C@H]2C[C@H](O)[C@@H](CO)O2)c(=O)[nH]1 | 0.639297369 |
| Nc1ccc(C(=O)O)cc1 | 0.54745208 |
| O=C(O)[C@@H]1CCCCN1 | 0.612154719 |
| CC1=C(C/C=C(\C)CCC[C@H](C)CCC[C@H](C)CCCC(C)C)C(=O)c2ccccc2C1=O | 0.476669602 |
| O=C[C@H](O)[C@H](O)[C@H](O)CO | 0.638577352 |
| OC[C@H](O)C(O)[C@@H](O)CO | 0.581867809 |
| CCCCCCCCCCCCCCCC=O | 0.562403195 |
| CCCCCCCCCCCCCCCCCCCCC(=O)O | 0.558450326 |
| CCCCCCCCCCCCCCCCCCCCCCCCCC(=O)O | 0.535226599 |
| O=C(O)/C=C/C(=O)O | 0.60816908 |
| CC(C)CCC[C@@H](C)[C@H]1CC[C@H]2C3=CC=C4C[C@@H](O)CC[C@]4(C)[C@H]3CC[C@]12C | 0.746564122 |
| O=C(O)CCc1cccc(O)c1 | 0.590640065 |
| CC1=C(/C=C/C(C)=C/C=C/C(C)=C/C=C/C=C(C)/C=C/C=C(C)/C=C/C2=C(C)CCCC2(C)C)C(C)(C)CCC1 | 0.5449026 |
| CC1=CCC(O)(C(C)C)CC1 | 0.546705285 |
| C[N+](C)(C)CC(=O)[O-] | 0.574213167 |
| Nc1ncnc2c1ncn2[C@@H]1O[C@H](COP(=O)(O)OP(=O)(O)OP(=O)(O)O)[C@@H](O)[C@H]1O | 0.574549476 |
| O=c1c(O)cc([C@H]2Oc3cc(O)cc(O)c3C[C@H]2O)cc2c([C@H]3Oc4cc(O)cc(O)c4C[C@H]3O)cc(O)c(O)c12 | 0.460149117 |
| CCCCC/C=C\C/C=C\CCCCCCCC(=O)O | 0.535482191 |
| CN(C)CC(=O)O | 0.658737004 |
| COc1cc(O[C@@H]2O[C@H](CO[C@@H]3O[C@H](CO)[C@@H](O)[C@H](O)[C@H]3O)[C@@H](O)[C@H](O)[C@H]2O)c2c(O)c3c(=O)oc(C)cc3cc2c1 | 0.476331517 |
| CCCCCCCCCCCCO | 0.619479318 |
| CN1CN([C@@H]2O[C@H](CO)[C@@H](O)[C@H]2O)c2nc(N)[nH]c(=O)c21 | 0.590668882 |
| COc1ccc2c(c1O)-c1c(OC)c(OC)cc3c1[C@H](C2)N(C)CC3 | 0.511310782 |
| O=C(O)Cc1c[nH]c2ccccc12 | 0.609101369 |
| O=C(O)C(O)=Cc1ccccc1 | 0.629017198 |
| CCCCCCCCCC=O | 0.613080935 |
| NCCc1ccc(O)cc1 | 0.582099979 |
| O=C(O)[C@@H]1CCN1 | 0.630020401 |
| O=C(O)[C@@H]1C[C@H](O)CN1 | 0.62639115 |
| C=C[C@]12CN(C)[C@@H]3[C@H]4CO[C@H](C[C@H]41)[C@]1(C(=O)N(OC)c4ccccc41)[C@@H]32 | 0.57265692 |
| C[C@@H]1O[C@@H](O[C@H]2C(=O)c3c(O)cc(O)cc3O[C@@H]2c2ccc(O)c(O)c2)[C@H](O)[C@H](O)[C@H]1O | 0.538609821 |
| C=C(C(=O)O)[C@@H]1CC[C@H](C)[C@@H]2CC(=O)C(C)=C2C1 | 0.59442635 |
| COC(=O)[C@@]12OC[C@]34[C@H]([C@@H](O)[C@@H]1O)[C@@]1(C)CC(=O)C(O)=C(C)[C@@H]1C[C@H]3OC(=O)[C@H](OC(=O)C=C(C)C)[C@@H]24 | 0.564516837 |
| COc1cc(CC/C=C/C(=O)CCc2ccc(O)c(OC)c2)ccc1O | 0.55085398 |
| O=c1ccoc2cc(O)cc(O)c12 | 0.464784188 |
| O=c1cc(-c2cc(O)c(O)c(O)c2)oc2cc(O)cc(O)c12 | 0.580858901 |
| O=C1C[C@@H](c2ccc(O)c(O)c2)Oc2cc(O)ccc21 | 0.64146458 |
| C=C(C)[C@@H]1CC=C(C=O)CC1 | 0.592209119 |
| COC1=C(OC)[C@H](O)[C@H](C/C=C(\C)CC/C=C(\C)CCC=C(C)C)[C@@H](C)C1=O | 0.496704286 |
| CC=C(C)C(=O)Oc1c(OC)c(OC)cc2c1-c1c(cc(OC)c(OC)c1OC)CC(C)(O)C(C)C2 | 0.481259965 |
| O=c1c2cc(O)c(O)cc2oc2cc(O)c([C@@H]3O[C@H](CO)[C@@H](O)[C@H](O)[C@H]3O)c(O)c12 | 0.565751663 |
| CC(C)c1cccc(=O)c(O)c1 | 0.55959193 |
| O=C1C[C@@H](O[C@@H]2O[C@H](CO)[C@@H](O)[C@H](O)[C@H]2O)CO1 | 0.72984655 |
| C[S+]([O-])CCCCN=C=S | 0.608840276 |
| COc1cc(OC)c2c(=O)cc(-c3cc(OC)c(OC)c(OC)c3)oc2c1 | 0.54163482 |
| O=C1/C(=C/c2ccc(O)c(O)c2)COc2cc(O)ccc21 | 0.651914183 |
| C1CCN2C[C@@H]3C[C@@H](CN4CCCC[C@@H]34)[C@H]2C1.O.O.O.O.O.O=S(=O)(O)O | 0.583641926 |
| COC(=O)[C@@H](Cc1ccc(O)c(O)c1)OC(=O)/C=C/c1ccc(O)c2c1[C@H](C(=O)O[C@H](Cc1ccc(O)c(O)c1)C(=O)OC)[C@@H](c1ccc(O)c(O)c1)O2 | 0.408460888 |
| CC[C@H]1C2C[C@H]3[C@@H]4N(C)c5ccccc5C45C[C@@H](C2[C@H]5O)N3[C@@H]1O | 0.633214648 |
| COC1=CC(=O)O[C@@H](CCc2ccccc2)C1 | 0.699111124 |
| COc1cc(-c2coc3cc(O)ccc3c2=O)ccc1O | 0.577070738 |
| Cc1c([C@H](C)[C@@H]2NC[C@@H](C)C[C@H]2O)ccc2c1C[C@H]1[C@H]2CC=C2C[C@@H](O)CC[C@@]21C | 0.607941664 |
| CC[C@H]1CN2CC[C@H]1C[C@@H]2[C@@H](O)c1ccnc2ccc(OC)cc12 | 0.523790184 |
| CCN1C[C@]2(COC)CC[C@H](O)C34[C@@H]5C[C@H]6[C@H](O)[C@@H]5[C@](O)(C[C@@H]6OC)[C@@H]([C@H](OC)[C@@H]32)[C@@H]14 | 0.720637749 |
| CCN1C[C@]2(OC(=O)c3ccccc3NC(C)=O)CC[C@H](OC)[C@]34[C@@H]2C[C@@H]([C@@H]13)[C@@]1(O)C[C@H](OC)[C@H]2C[C@@H]4[C@]1(O)[C@H]2OC | 0.649190205 |
| O=C1CCC[C@@H]2[C@H]3CCCN4CCC[C@H](CN12)[C@@H]34 | 0.642430102 |
| COC[C@@H](Cc1ccc(OC)c(OC)c1)[C@@H](COC)Cc1cc(OC)c2c(c1)OCO2 | 0.397109209 |
| COc1cc([C@@H]2c3cc4c(cc3[C@H](O)[C@H]3COC(=O)[C@H]23)OCO4)cc(OC)c1OC | 0.550406049 |
| C[C@@]12CC[C@@H]3[C@@](CC[C@H]4[C@@]3(C)CCC[C@@]4(C)C(=O)O)(CC1=O)C2 | 0.639732241 |
| Cc1c2ccncc2c(C)c2c1[nH]c1ccccc12 | 0.524366168 |
| CC[C@@]1(O)C(=O)OCc2c1cc1n(c2=O)Cc2cc3c(N)cccc3nc2-1 | 0.635151448 |
| CC[C@@]1(O)C(=O)OCc2c1cc1n(c2=O)Cc2cc3c(OC)cccc3nc2-1 | 0.594055594 |
| CC[C@@]1(O)C(=O)OCc2c1cc1n(c2=O)Cc2cc3cc(O)ccc3nc2-1 | 0.62510416 |
| Cc1cc(=O)oc2cc(O)c(O)cc12 | 0.53526489 |
| O=c1c(-c2ccc(O)cc2)coc2cc(O[C@@H]3O[C@H](CO)[C@@H](O)[C@H](O)[C@H]3O)ccc12 | 0.644705615 |
| O=C(O)CC(CC(=O)O)C(=O)O | 0.629488523 |
| CC(=O)O[C@@H]1CC[C@@]2(C)[C@@H](CC[C@]3(C)[C@@H]2CC=C2[C@@H]4[C@@H](C)[C@H](C)CC[C@]4(C)CC[C@]23C)[C@@]1(C)C(=O)O | 0.777477924 |
| COc1cc(O)c2c(=O)c3cc(O)ccc3oc2c1 | 0.628861179 |
| CC1(C)CCC2(CO)C(=C3C=CC4C5(C)CCC(OC6OC(COC7OC(CO)C(O)C(O)C7O)C(OC7OC(CO)C(O)C(O)C7O)C(O)C6O)C(C)(CO)C5CCC4(C)C3(C)CC2O)C1 | 0.703800474 |
| COc1cccc2oc(-c3ccccc3)cc(=O)c12 | 0.618244405 |
| O=C1c2ccccc2C(=O)c2c(O)cccc21 | 0.572600268 |
| O=c1ccc2cc3ccoc3cc2o1 | 0.494197966 |
| COc1ccc(-c2cc(=O)c3c(OC)c(OC)c(OC)c(OC)c3o2)cc1O | 0.510264728 |
| CC(C)=CCc1cc(-c2coc3cc(O)ccc3c2=O)ccc1O | 0.538461107 |
| C[C@H]1[C@H](C)CC[C@]2(C)CC[C@]3(C)C(=CC[C@@H]4[C@@]5(C)CC[C@@H](O)[C@](C)(C(=O)O)C5CC[C@]43C)[C@H]12 | 0.882154394 |
| O=c1cc(-c2ccc(O)c(O)c2)oc2cc(O[C@@H]3O[C@H](CO)[C@@H](O)[C@H](O)[C@H]3O)cc(O)c12 | 0.544465032 |
| C=C(CO)C(=O)O[C@H]1CC(=C)[C@@H]2C[C@H](O)C(=C)[C@@H]2[C@H]2OC(=O)C(=C)[C@@H]21 | 0.584227114 |
| CC[C@]12C=CCN3CC[C@@]4(c5ccc(OC)cc5N(C)C4[C@@](O)(C(=O)OC)[C@@H]1OC(C)=O)[C@@H]32 | 0.474252113 |
| CN1C(=O)[C@@H](O)[C@H](c2ccccc2)[C@@H]1[C@@H](O)c1ccccc1 | 0.552052944 |
| COc1cc([C@@H]2c3cc4c(cc3[C@@H](O)[C@H]3COC(=O)[C@H]23)OCO4)cc(OC)c1O | 0.578994797 |
| COc1cc([C@@H]2c3cc4c(cc3C(=O)[C@H]3COC(=O)[C@H]23)OCO4)cc(OC)c1OC | 0.48209052 |
| COC(=O)c1cc(OC)c2c(c1-c1c(C(=O)OC)cc(OC)c3c1OCO3)OCO2 | 0.51352384 |
| C=C(CC[C@@H](C(=O)O)[C@H]1[C@H](O)C[C@@]2(C)C3=CC[C@H]4C(C)(C)C(=O)CC[C@]4(C)C3=CC[C@]12C)C(C)C | 0.728142815 |
| CC(C)=CC[C@H](O)C1=CC(=O)c2c(O)ccc(O)c2C1=O | 0.519391113 |
| O=C(/C=C/c1ccc(O)c(O)c1)O[C@@H]1C[C@](OC(=O)/C=C/c2ccc(O)c(O)c2)(C(=O)O)C[C@@H](O)[C@H]1O | 0.473955327 |
| O.O.O=c1c(O)c(-c2ccc(O)c(O)c2)oc2cc(O)cc(O)c12 | 0.636442119 |
| COc1ccc(CCC(=O)c2ccc(O)cc2)c(OC)c1 | 0.652534661 |
| CC/C=C\C/C=C\C/C=C\CCCCCCCC(=O)O | 0.518672708 |
| C[C@]12CC[C@H](O)C[C@H]1CC[C@@H]1[C@@H]2C(=O)[C@@H](O)[C@]2(C)[C@@H](c3ccc(=O)oc3)CC[C@]12O | 0.66482482 |
| O=c1ccc2cc(O)c(O)cc2o1 | 0.515088611 |
| C[C@@H]1O[C@@H](O[C@H]2[C@H](Oc3c(-c4ccc(O)cc4)oc4cc(O)cc(O)c4c3=O)O[C@H](CO)[C@@H](O)[C@@H]2O)[C@H](O)[C@H](O)[C@H]1O | 0.438681196 |
| C[C@@H]([C@H](C)O)[C@H](N)C(=O)O | 0.622765955 |
| C/C1=C\C(=O)c2c(C)coc2C/C(C)=C/CC1 | 0.633286223 |
| COc1c(O)cc2c3c1-c1ccccc1C[C@H]3N(C)CC2 | 0.503331268 |
| C=C1CC23C[C@H]4[C@@H]5[C@@]6(C)C[C@H](OC(C)=O)C[C@]57C(N4C6)[C@@]2(O)[C@H](OC(C)=O)[C@H]1[C@@H](O)[C@H]37 | 0.67508945 |
| CC(C)=CC[C@@H](O)C1=CC(=O)c2c(O)ccc(O)c2C1=O | 0.519391113 |
| COc1ccc(C[C@H]2COC(=O)[C@@H]2Cc2ccc(O[C@@H]3O[C@H](CO)[C@@H](O)[C@H](O)[C@H]3O)c(OC)c2)cc1OC | 0.44132894 |
| C[C@@H]1O[C@@H](Oc2cc(O)c3c(=O)c(O[C@@H]4O[C@@H](C)[C@H](O)[C@@H](O)[C@H]4O)c(-c4ccc(O)cc4)oc3c2)[C@H](O)[C@H](O)[C@H]1O | 0.447183155 |
| COc1cc([C@@H]2c3cc4c(cc3[C@H](O)[C@H]3COC(=O)[C@@H]23)OCO4)cc(OC)c1OC | 0.550406049 |
| COc1cc([C@@H]2c3cc4c(cc3[C@H](O)[C@H]3COC(=O)[C@@H]23)OCO4)cc(OC)c1OC | 0.550406049 |
| CC(=O)OC(C)(C)CCC(=O)[C@](C)(O)[C@H]1[C@H](O)C[C@@]2(C)[C@@H]3CC=C4[C@@H](C[C@H](O)[C@@H](O)C4(C)C)[C@]3(C)C(=O)C[C@]12C | 0.731752755 |
| COC1=CC(=O)OC(C=Cc2ccccc2)C1 | 0.693406319 |
| COc1c(OC)c2occc2c2oc(=O)ccc12 | 0.605864426 |
| COc1c(O[C@@H]2O[C@H](CO)[C@@H](O)[C@H](O)[C@H]2O)cc2c(c1OC)-c1ccc(SC)c(=O)cc1[C@@H](NC(C)=O)CC2 | 0.484745393 |
| CC(C)=CCc1c(O)cc2c(c1O)C(=O)C[C@@H](c1ccc(O)cc1)O2 | 0.567635455 |
| CC1(C)[C@@H]2CC[C@@]1(C)[C@@H](O)C2 | 0.512554996 |
| CC1(C)[C@H]2CC[C@]1(C)[C@H](O)C2 | 0.512554996 |
| C=C1[C@H]2C[C@@]3(C4C[C@H]5C6(C4N(CC)C[C@]5(C)CC[C@@H]6O)[C@@H]3CC2=O)[C@@H]1O | 0.793226678 |
| O=C1CC(c2ccccc2)Oc2ccc(O)cc21 | 0.585626076 |
| CN1CCC=C(C(=O)O)C1.Cl | 0.646949524 |
| C=C(C)[C@@H]1[C@H]2OC(=O)[C@@H]1[C@]1(O)C[C@H]3O[C@]34C(=O)O[C@H]2[C@]14C | 0.546380712 |
| COc1c2ccoc2c(OCC=C(C)C)c2oc(=O)ccc12 | 0.642963002 |
| CN1CCc2cc3c(cc2[C@H]1[C@@H]1OC(=O)c2c1ccc1c2OCO1)OCO3 | 0.540526744 |
| O=C1C=C2C=C[C@@H]3C[C@@]2(O1)[C@H]1CCCCN31 | 0.608045473 |
| Cl.O=C(O)C1=CCCNC1 | 0.659918463 |
| COc1cc(O)ccc1C(=O)/C=C/c1ccc(O)cc1 | 0.681371774 |
| CC(C)C1=Cc2ccc3c(c2C(=O)C1=O)CCCC3(C)C | 0.503144583 |
| C=CCc1ccc(OC)c(OC)c1 | 0.602609827 |
| C=C(C)[C@@H]1CCC(C)=C[C@H]1c1c(O)cc(CCC)cc1O | 0.473934457 |
| C=C1[C@H]2C[C@H]2[C@]2(C)Cc3occ(C)c3[C@H](OC(C)=O)[C@@H]12 | 0.613472632 |
| C[C@@]12CCc3occc3[C@H]1CC[C@@]13C[C@@H](CC[C@H]12)[C@@](O)(CO)C3 | 0.582167086 |
| COc1ccc(-c2cc(=O)c3c(OC)c(OC)c(OC)cc3o2)cc1OC | 0.525049073 |
| C[C@@H]1CC[C@@]2(OC1)O[C@H]1C[C@H]3[C@@H]4CC=C5C[C@@H](O)C[C@@H](O)[C@]5(C)[C@H]4CC[C@]3(C)[C@H]1[C@@H]2C | 0.83683999 |
| OC[C@H]1O[C@@H](Oc2c[nH]c3ccccc23)[C@H](O)[C@@H](O)[C@@H]1O | 0.652786943 |
| O=C(C=Cc1ccc(O)c(O)c1)OC(C(=O)O)C(OC(=O)C=Cc1ccc(O)c(O)c1)C(=O)O | 0.493548668 |
| O=C1OCC2OC(O)C(O)C(O)C2OC(=O)c2cc(O)c(O)c(O)c2-c2c(O)c(O)c3oc(=O)c4c(c(O)c(O)c5oc(=O)c2c3c54)-c2c1cc(O)c(O)c2O | 0.386008894 |
| O=C(OC[C@H]1O[C@@H](Oc2c(-c3ccc(O)cc3)oc3cc(O)cc(O)c3c2=O)[C@H](O)[C@@H](O)[C@@H]1O)c1cc(O)c(O)c(O)c1 | 0.442146605 |
| O=C(C=Cc1ccc(O)c(O)c1)OC1C(O)CC(OC(=O)C=Cc2ccc(O)c(O)c2)(C(=O)O)CC1O | 0.46728695 |
| O=C(O)/C=C/c1ccccc1O | 0.589775259 |
| COc1cc(O)c2c(=O)c3c(O)ccc(O)c3oc2c1 | 0.630026741 |
| COc1cc2c(O)c3c(c(-c4ccc5c(c4)OCO5)c2cc1OC)C(=O)OC3 | 0.642602407 |
| CC1(C)CC[C@]2(C(=O)O)CC[C@]3(C)C(=CC[C@@H]4[C@@]5(C)CCC(=O)C(C)(C)[C@@H]5CC[C@]43C)[C@@H]2C1 | 0.79999644 |
| COc1cc(O)cc(O)c1C(=O)/C=C/c1ccc(O)cc1 | 0.698215292 |
| O=c1cc(-c2ccc(O)cc2)oc2cc(O[C@@H]3O[C@H](CO)[C@@H](O)[C@H](O)[C@H]3O)cc(O)c12 | 0.575020042 |
| COc1cc2c(c(OC)c1OC)-c1c(cc3c(c1OC)OCO3)[C@H](OC(=O)c1ccccc1)[C@@](C)(O)[C@@H](C)C2 | 0.507310595 |
| O=C(/C=C/c1ccc(O)c(O)c1)O[C@@H](C(=O)O)[C@@H](OC(=O)/C=C/c1ccc(O)c(O)c1)C(=O)O | 0.493548668 |
| O=C1Cc2ccccc2N1 | 0.496607426 |
| O=C(/C=C/c1ccc(O)c2c1[C@H](C(=O)O)[C@@H](c1ccc(O)c(O)c1)O2)O[C@H](Cc1ccc(O)c(O)c1)C(=O)O | 0.448433218 |
| C[C@]12CCC[C@@]3(COC1=O)[C@@H]1CC[C@@H]4C[C@@]1(CC[C@@H]32)C[C@@]4(C)O | 0.799200991 |
| c1ccc2cccc-2cc1 | 0.566151466 |
| C=C(C)[C@@H]1CC[C@]2(C(=O)O)CC[C@]3(C)[C@H](CC[C@@H]4[C@@]5(C)CC[C@H](OC(=O)CC(C)(C)C(=O)O)C(C)(C)[C@@H]5CC[C@]43C)[C@@H]12 | 0.773723435 |
| O.O=c1cc(-c2ccccc2)oc2cc(O)c(O)c(O)c12 | 0.600827602 |
| CC(=O)c1ccc(O)cc1 | 0.543823618 |
| O=C(CCc1ccc(O)cc1)c1c(O)cc(O[C@@H]2O[C@H](CO)[C@@H](O)[C@H](O)[C@H]2O)cc1O | 0.587486811 |
| COc1ccc(-c2cc(=O)c3c(O)cc(OC)cc3o2)cc1 | 0.61474756 |
| O=C1C[C@@H](c2ccc(O)c(O)c2)Oc2c1ccc(O)c2O | 0.642963514 |
| COc1cc(C=O)cc(OC)c1O | 0.543930208 |
| O=c1cc(-c2ccc(O)c(O)c2)oc2cc(O)c([C@@H]3O[C@H](CO)[C@@H](O)[C@H](O)[C@H]3O)c(O)c12 | 0.521055811 |
| Oc1cc(O)c2c(c1)O[C@@H](c1ccc(O)c(O)c1)[C@H](O)C2 | 0.597670335 |
| C=C(C)[C@@H]1CC[C@]2(C)[C@H](CC=C3[C@@H]4[C@@H](C)[C@H](C)CC[C@]4(C)CC[C@]32C)[C@@]1(C)CCC(=O)O | 0.866366959 |
| CC(C)(O)[C@@H]1Cc2cc3ccc(=O)oc3cc2O1 | 0.628218488 |
| C[C@@H]1O[C@@H](OC[C@H]2O[C@@H](Oc3cc4c(O)cc(O)cc4[o+]c3-c3ccc(O)c(O)c3)[C@H](O)[C@@H](O)[C@@H]2O)[C@H](O)[C@H](O)[C@H]1O.[Cl-] | 0.45316622 |
| CCCCCCCC(=O)CCc1ccc(O)c(OC)c1 | 0.623859675 |
| OCc1ccccc1O[C@@H]1O[C@H](CO)[C@@H](O)[C@H](O)[C@H]1O | 0.705819321 |
| COc1cc(CCC(=O)CC(O)CCc2ccc(O)c(OC)c2)ccc1O | 0.556759246 |
| COc1cc(/C=C/C(=O)NCCc2ccc(O)cc2)ccc1O | 0.660005845 |
| C[C@]12CC[C@H](O)C[C@H]1CC[C@@H]1[C@@H]2[C@H](O)C[C@]2(C)[C@@H](c3ccc(=O)oc3)CC[C@]12O | 0.725354497 |
| COc1cc(OC)c(OC)cc1C=O | 0.592874058 |
| C[C@@]12C[C@@]3(O)O[C@@H](O1)[C@]1(COC(=O)c4ccccc4)[C@H]3C[C@@]12O[C@@H]1O[C@H](COC(=O)c2ccccc2)[C@@H](O)[C@H](O)[C@H]1O | 0.481945918 |
| COc1cc([C@@H]2OC[C@@H]3[C@H]2CO[C@H]3c2ccc(O)c(OC)c2)ccc1O | 0.536178633 |
| Cc1cccc(C(=O)O)c1O | 0.56306588 |
| C=C1CC[C@@H]2[C@](C)(CO[C@@H]3O[C@H](CO)[C@@H](O)[C@H](O)[C@H]3O)CCC[C@@]2(C)[C@@H]1CCC1=CCOC1=O | 0.631528207 |
| Oc1ccc([C@H]2Oc3cc(O)cc(O)c3C[C@H]2O)cc1 | 0.609078115 |
| CC(C)(C)c1cc(O)c(C(C)(C)C)cc1O | 0.624099751 |
| COC(=O)c1ccccc1O[C@@H]1O[C@H](CO[C@@H]2OC[C@@H](O)[C@H](O)[C@H]2O)[C@@H](O)[C@H](O)[C@H]1O | 0.596619082 |
| O=c1c2ccccc2nc2n1CCc1c-2[nH]c2ccccc12 | 0.551138498 |
| Cc1c(O)cc2c(c1C)O[C@](C)(CCC[C@H](C)CCC[C@H](C)CCCC(C)C)CC2 | 0.625173377 |
| CCOC(=O)/C=C/c1ccc(OC)cc1 | 0.690634803 |
| CC(=O)OC(C)(C)CCC(=O)[C@](C)(O)[C@H]1[C@H](O)C[C@@]2(C)[C@@H]3CC=C4[C@@H](C[C@H](O)C(=O)C4(C)C)[C@]3(C)C(=O)C[C@]12C | 0.721901107 |
| COc1cc2ccc(=O)oc2c(OC)c1O | 0.608404284 |
| COc1cc(/C=C/CO)cc(OC)c1O | 0.584713116 |
| CCCCCCC/C=C/C(=O)CCc1ccc(O)c(OC)c1 | 0.563593132 |
| CCCCCCCCC/C=C/C(=O)CCc1ccc(O)c(OC)c1 | 0.503861471 |
| NC(=O)c1ccccc1O | 0.572113324 |
| COc1ccc(C(=O)O)cc1OC | 0.577227958 |
| COc1cc(C[C@H](C)[C@H](C)Cc2ccc3c(c2)OCO3)ccc1O | 0.505937567 |
| COc1cc(/C=C/C(=O)OCCc2ccccc2)ccc1O | 0.695529294 |
| O.Oc1cc(O)c2c(c1)OC(c1ccc(O)c(O)c1)C(O)C2 | 0.611206815 |
| CC(C)c1cc2c(c(O)c1O)[C@@]13CCCC(C)(C)[C@@H]1[C@H](OC3=O)[C@H]2O | 0.665683016 |
| CC(C)=CCC[C@H](C(=O)O)[C@@H]1CC[C@]2(C)C3=C(CC[C@@]12C)[C@@]1(C)CCC(=O)C(C)(C)[C@@H]1CC3 | 0.779852575 |
| Cl.c1cc2c(c3c1CC1c4cc5c(cc4CCN1C3)OCO5)OCO2 | 0.52682211 |
| COc1ccc(-c2cc(=O)c3c(O)c(OC)c(O)cc3o2)cc1 | 0.60648324 |
| C[C@@]12C[C@@]3(O)O[C@@H](O1)[C@]1(COC(=O)c4ccccc4)[C@H]3C[C@@]12O[C@@H]1O[C@H](CO)[C@@H](O)[C@H](O)[C@H]1O | 0.567649667 |
| C=Cc1cc(OC)c(O)c(OC)c1 | 0.53185482 |
| Oc1cc(O)c2c(c1)O[C@H](c1ccc(O)c(O)c1)[C@@H](O)C2 | 0.597670335 |
| O=C(O[C@@H]1Cc2c(O)cc(O)cc2O[C@H]1c1ccc(O)c(O)c1)c1cc(O)c(O)c(O)c1 | 0.580164834 |
| Cc1cc(=O)c2c(O)c3c(cc2o1)OC(C)(C)[C@@H](O)C3 | 0.590050859 |
| C=C1C(=O)O[C@H]2[C@H]1CCC(C)=C1CC[C@@](C)(O)[C@@H]12 | 0.642251189 |
| COc1c2ccoc2c(OC[C@H]2OC2(C)C)c2oc(=O)ccc12 | 0.600422015 |
| C/C1=C\CC/C(C)=C/CC(C)(C)/C=C/C1 | 0.570816742 |
| COc1cccc2c1cc1c3c(cc4c(c32)OCO4)C(=O)N1 | 0.545732632 |
| O=C(O)c1cc(=O)cc(C(=O)O)o1 | 0.553074439 |
| CCC/C=C1\OC(=O)C2=C1CC[C@H](O)[C@H]2O | 0.67219281 |
| C[C@H]1CC[C@@H]2N(C1)C[C@H]1[C@@H]3C[C@H]4[C@@H](C[C@@H](O)[C@H]5C[C@@H](O)CC[C@@]54C)[C@@H]3CC[C@@H]1[C@]2(C)O | 0.788327629 |
| COc1ccc(-c2cc(=O)c3c(OC)cc(OC)cc3o2)cc1 | 0.602284584 |
| O=c1c(-c2ccc(O)cc2)coc2c([C@@H]3O[C@H](CO[C@@H]4OC[C@@H](O)[C@H](O)[C@H]4O)[C@@H](O)[C@H](O)[C@H]3O)c(O)ccc12 | 0.504920547 |
| COc1cc(O)c2c(c1)oc(=O)c1c3cc(O)c(O)cc3oc21 | 0.606455402 |
| CC(C)=CCc1c(O)cc(O)c2c(=O)c(-c3ccc(O)cc3)coc12 | 0.555317804 |
| CC1CCC2(OC1)OC1CC3C4CC=C5CC(OC6OC(CO)C(OC7OC(C)C(OC8OC(C)C(O)C(O)C8O)C(O)C7O)C(O)C6OC6OC(C)C(O)C(O)C6O)CCC5(C)C4CCC3(C)C1C2C | 0.785102261 |
| O=C(O)/C=C/c1ccc(O)c(O)c1/C=C/c1ccc(O)c(O)c1 | 0.66609289 |
| COC(=O)C1=CO[C@@H](O[C@@H]2O[C@H](CO)[C@@H](O)[C@H](O)[C@H]2O)[C@H]2[C@@H]1[C@H](O)C[C@]2(C)OC(C)=O | 0.53780344 |
| C=C1C(=O)[C@]23[C@H](O)[C@H]1CC[C@H]2[C@@]12CO[C@]3(O)[C@@H](O)C1C(C)(C)CC[C@@H]2OC(C)=O | 0.652416284 |
| COC(=O)C1=CO[C@@H](O[C@@H]2O[C@H](CO)[C@@H](O)[C@H](O)[C@H]2O)[C@@H]2[C@H](C)O[C@@H](O)C[C@H]12 | 0.680482304 |
| COc1cc(/C=C/C(=O)NCC(O)c2ccc(O)cc2)ccc1O | 0.665828594 |
| COc1cc(-c2cc(=O)c3c(O)cc(O)cc3o2)ccc1O | 0.612135025 |
| C=C(C)[C@@H]1CC[C@]2(C)CC[C@]3(C)[C@H](CC[C@@H]4[C@@]5(C)CC[C@H](O)C(C)(C)[C@@H]5CC[C@]43C)[C@@H]12 | 0.871233467 |
| C/C=C(/C)C(=O)O[C@@H]1[C@H](OC(C)=O)c2c(ccc3ccc(=O)oc23)OC1(C)C | 0.573205272 |
| COc1cc2c(cc1O)C[C@H]1c3cc(O)c(OC)cc3CC[N@@+]1(C)C2 | 0.498264466 |
| COc1ccc2c(c1OC)C[C@@H]1c3c(cc4c(c3-2)OCO4)CCN1C | 0.497725909 |
| Cc1c(O)c(C)c2c(c1O)C(=O)CC(c1ccc(O)cc1)O2 | 0.586037703 |
| O=c1c(O)c(-c2ccc(O)cc2)oc2c(O)c(O)cc(O)c12 | 0.627531075 |
| C=C(CC[C@@H](C(=O)O)[C@H]1[C@H](O)C[C@@]2(C)C3=C(CC[C@]12C)[C@@]1(C)CC[C@H](OC(C)=O)C(C)(C)[C@@H]1CC3)C(C)C | 0.746622732 |
| CC(C)=CCC/C(C)=C/Cc1c(O)c(CC=C(C)C)c2c(c1O)C(=O)C1=C[C@@H]3C[C@H]4C(C)(C)O[C@@](C/C=C(/C)C(=O)O)(C3=O)[C@@]14O2 | 0.467315104 |
| COc1ccc(-c2cc(=O)c3c(OC)c(OC)c(OC)c(OC)c3o2)cc1 | 0.527750233 |
| O=C(/C=C/c1ccc(O)cc1)CC(=O)/C=C/c1ccc(O)cc1 | 0.584324439 |
| CO[C@H]1[C@H](O)[C@@H](O)[C@H](O)[C@H](O)[C@H]1O | 0.574039782 |
| COc1cc(C[C@@]2(O)C(=O)OC[C@@H]2Cc2ccc(OC)c(OC)c2)ccc1O | 0.648121066 |
| CC(CCC1(O)OC2CC3C4CCC5CC(OC6OC(CO)C(O)C(O)C6OC6OC(CO)C(O)C(O)C6O)CCC5(C)C4CCC3(C)C2C1C)COC1OC(CO)C(O)C(O)C1O | 0.67777729 |
| Cc1cc(=O)c2c(O)c3c(O[C@@H]4O[C@H](CO)[C@@H](O)[C@H](O)[C@H]4O)cc(O)cc3cc2o1 | 0.613163239 |
| COc1cc(C)c2c(=O)cc(C[C@@H](C)O)oc2c1[C@@H]1O[C@H](CO)[C@@H](O)[C@H](O)[C@H]1OC(=O)/C=C/c1ccc(O)cc1 | 0.47788136 |
| CC(C)=CCC/C(C)=C/COc1c2occc2cc2ccc(=O)oc12 | 0.568482797 |
| CC(C)=CCCC(C)=CCOc1ccc2ccc(=O)oc2c1 | 0.647631291 |
| COc1ccc2c(c1)[nH]c1c(C)nccc12 | 0.494843979 |
| O=c1c2cc(O)ccc2oc2cc(O)cc(O)c12 | 0.599441429 |
| COc1ccc(-c2cc(=O)c3c(OC)c(OC)c(OC)cc3o2)cc1 | 0.560040191 |
| COc1cc([C@H]2OC[C@H]3[C@@H]2CO[C@@H]3c2cc(OC)c(OC)c(OC)c2)cc(OC)c1OC | 0.449980955 |
| C=CCC1=C[C@]2(OC)C(=CC1=O)O[C@H](c1ccc(OC)c(OC)c1)[C@H]2C | 0.600595256 |
| Cc1cc2cc3c(C)cc(=O)oc3c(C)c2o1 | 0.550763925 |
| Cc1cc(O)c(C)c2c1O[C@](C)(CCC[C@H](C)CCC[C@H](C)CCCC(C)C)CC2 | 0.623871293 |
| CC(=O)OC1CC(=O)OC(C)(C)C2CC(=O)C3(C)C(CCC4(C)C(c5ccoc5)OC(=O)C5OC543)C12C | 0.651797583 |
| COc1ccc(-c2oc3c(CCC(C)(C)O)c(O[C@@H]4O[C@H](CO)[C@@H](O)[C@H](O)[C@H]4O)cc(O)c3c(=O)c2O)cc1 | 0.473794829 |
| CC(C)=C/C=C1\O[C@H]2CC(C)=CC(=O)[C@H]2[C@@]1(C)O | 0.615870913 |
| N[C@@H](CNC(=O)C(=O)O)C(=O)O | 0.599529544 |
| CC(C)=CCC(OC(=O)C=C(C)C)C1=CC(=O)c2c(O)ccc(O)c2C1=O | 0.528514486 |
| CC1(C)CCC[C@](C)([C@H]2CC[C@]3(C)[C@@H]2[C@H](O)C[C@@H]2[C@@]4(C)CC[C@H](O)C(C)(C)[C@@H]4CC[C@]23C)O1 | 0.900037895 |
| COc1cc(/C=C/C(=O)O[C@@H]2C[C@@](O)(C(=O)O)C[C@@H](O)[C@@H]2O)ccc1O | 0.621203801 |
| O=C1CCc2ccccc2O1 | 0.502860193 |
| COc1cc2ccc(=O)oc2cc1O[C@@H]1O[C@H](CO)[C@@H](O)[C@H](O)[C@H]1O | 0.680691463 |
| O=C(/C=C/c1ccc(O)c(O)c1)N[C@@H](Cc1c[nH]c2ccccc12)C(=O)O | 0.548409545 |
| CC(CC(=O)C[C@@H](C)[C@H]1CC(=O)[C@@]2(C)C3=C(C(=O)C[C@]12C)[C@@]1(C)CCC(=O)C(C)(C)[C@@H]1C[C@@H]3O)C(=O)O | 0.717322493 |
| CC(C)=CCc1c(O)cc(O)c2c1O[C@H](c1ccc(O)cc1)CC2=O | 0.560799518 |
| CC(C)=CCCC(C)=CCCC(C)=CCCC=C(C)CCC=C(C)CCC=C(C)C | 0.517882842 |
| CCCCCc1cc(O)c(C/C=C(\C)CCC=C(C)C)c(O)c1 | 0.547379729 |
| C[C@H]1[C@H](C)CC[C@]2(C)CC[C@]3(C)C(=CC[C@@H]4[C@@]5(C)CC[C@H](O)C(C)(C)[C@@H]5CC[C@]43C)[C@H]12 | 0.849185777 |
| COc1cc(CCC(O)CC(O)CCc2ccc(O)c(OC)c2)ccc1O | 0.560122778 |
| COc1cc(O)c2c(c1)O[C@H](c1cc(O)cc(O)c1)CC2=O | 0.609161681 |
| COc1cc(C[C@H]2COC(=O)[C@@H]2Cc2ccc(O)c(OC)c2)ccc1O | 0.576683503 |
| COc1cc(CC(COC2OC(CO)C(O)C(O)C2O)C(COC2OC(CO)C(O)C(O)C2O)Cc2ccc(O)c(OC)c2)ccc1O | 0.365546469 |
| Cc1cccc2c3c(ccc12)C1=C(C(=O)C3=O)C(C)CO1 | 0.544596555 |
| CC1=CC(=O)c2c(O)cccc2C1=O | 0.469483068 |
| CC(C)=CCCC(C)=CC=CC(C)=CC=CC(C)=CC=CC=C(C)C=CC=C(C)C=CC=C(C)CCC=C(C)C | 0.487358556 |
| O=C(/C=C/c1ccc(O)c(O)c1)c1ccc(O)c(O)c1O | 0.651356835 |
| Oc1cc(O)c2c(c1)O[C@H](c1ccc(O)c(O)c1)[C@H](O)[C@H]2c1c(O)cc(O)c2c1O[C@H](c1ccc(O)c(O)c1)[C@@H](O)C2 | 0.438105926 |
| CC1(C)C2CC[C@]3(C)[C@H](C(=O)C=C4[C@H]5C[C@@](C)(C(=O)O)CC[C@]5(C)CC[C@]43C)[C@@]2(C)CC[C@@H]1O | 0.789223864 |
| O=C(/C=C/c1ccccc1)c1ccc(O)cc1 | 0.679514865 |
| O=C1/C(=C/c2ccc(O)c(O)c2)Oc2cc(O)cc(O)c21 | 0.668490116 |
| O=c1oc2ccccc2cc1O | 0.507083906 |
| C/C=C1/CN2[C@@H]3C[C@@]45c6ccccc6N[C@]4(O3)[C@@H]2C[C@@H]1[C@H]5C(=O)OC | 0.655933531 |
| Cc1cc(=O)oc2c(O)c(O)ccc12 | 0.512535962 |
| COc1ccc(-c2cc(=O)c3c(O)c(OC)c(OC)c(OC)c3o2)cc1OC | 0.518125863 |
| CC(=O)O[C@@H]1CC[C@@]2(C)[C@@H](CC[C@]3(C)[C@@H]2[C@H](O)C=C2[C@@H]4[C@@H](C)[C@H](C)CC[C@]4(C)CC[C@]23C)[C@@]1(C)C(=O)O | 0.790273852 |
| C[C@H]1[C@H](C)CC[C@]2(C)CC[C@]3(C)C(=CC(=O)[C@@H]4[C@@]5(C)CC[C@@H](O)[C@](C)(C(=O)O)[C@@H]5CC[C@]43C)[C@H]12 | 0.7991662 |
| COc1cc([C@@H]2Oc3c(O)cc([C@H]4Oc5cc(O)cc(O)c5C(=O)[C@@H]4O)cc3[C@H]2CO)ccc1O | 0.490032419 |
| O=C(c1c(O)cc(O)cc1O)C(c1coc2cc(O)cc(O)c2c1=O)C(c1ccc(O)cc1)c1ccc(O)cc1 | 0.409735505 |
| C[C@@H]1C[C@@H]([C@@H](O)C(C)(C)O)O[C@H]2C[C@@]3(C)C(=C12)C[C@H](O)[C@H]1[C@@]2(C)CCC(=O)C(C)(C)[C@@H]2CC[C@@]13C | 0.884191394 |
| OCC1OC(OC2OC=CC3C(O)C4OC4(CO)C23)C(O)C(O)C1O | 0.615960503 |
| COc1cc2oc(=O)ccc2cc1O | 0.542460062 |
| COC(=O)[C@@]1(O)C[C@@H](O)[C@H](O)[C@H](OC(=O)/C=C/c2ccc(O)c(O)c2)C1 | 0.570883624 |
| CSC(=O)OCC1=C[C@@H]2OC(=O)C3=CO[C@@H](O[C@@H]4O[C@H](CO)[C@@H](O)[C@H](O)[C@H]4O)[C@H]1[C@@H]32 | 0.587842544 |
| O=c1cc(-c2ccc(O)cc2)oc2c(O[C@@H]3O[C@H](CO)[C@@H](O)[C@H](O)[C@H]3O)c(O)cc(O)c12.OC[C@H]1O[C@@H](O)[C@H](O)[C@@H](O)[C@@H]1O | 0.439668776 |
| COc1cc(-c2cc(=O)c3c(O)c(OC)c(O)cc3o2)cc(OC)c1O | 0.592594395 |
| COc1c(O[C@@H]2O[C@H](CO)[C@@H](O)[C@H](O)[C@H]2O)cc2occ(-c3ccc(O)cc3)c(=O)c2c1O | 0.535181652 |
| O=c1c(O[C@@H]2O[C@H](CO[C@@H]3O[C@H](CO)[C@@H](O)[C@H](O)[C@H]3O)[C@@H](O)[C@H](O)[C@H]2O)c(-c2ccc(O)c(O)c2)oc2cc(O)cc(O)c12 | 0.409824695 |
| C[C@@H]1O[C@@H](O[C@H]2C(=O)c3c(O)cc(O)cc3O[C@@H]2c2ccc(O)cc2)[C@H](O)[C@H](O)[C@H]1O | 0.598583926 |
| COc1c2ccoc2c(OC[C@@H](O)C(C)(C)O)c2oc(=O)ccc12 | 0.633195464 |
| C=C1C[C@@]2(O)O[C@@]3(CC2=C(C)C)[C@@H](C)CC[C@@H]13 | 0.60240567 |
| CC1OC(OCC2OC(OCCc3ccc(O)c(O)c3)C(O)C(OC3OC(C)C(O)C(O)C3O)C2OC(=O)/C=C/c2ccc(O)c(O)c2)C(O)C(O)C1O | 0.423157578 |
| COc1c(C(C)C)cc2c(c1O)[C@@]1(C(=O)O)CCCC(C)(C)[C@@H]1CC2 | 0.572120131 |
| O=C(O)CCCC[C@@H]1SC[C@@H]2NC(=O)N[C@@H]21 | 0.680544589 |
| CC(=O)OC[C@H](Cc1ccccc1)NC(=O)[C@H](Cc1ccccc1)NC(=O)c1ccccc1 | 0.514032612 |
| COc1cc(-c2coc3cc(O)c(OC)c(O)c3c2=O)cc(O)c1OC | 0.594366449 |
| COc1cc2oc(-c3ccc(O)c(O)c3)cc(=O)c2c(O)c1[C@@H]1O[C@H](CO)[C@@H](O)[C@H](O)[C@H]1O | 0.521819978 |
| OC[C@H]1O[C@H](Oc2ccc(O)cc2)[C@H](O)[C@@H](O)[C@@H]1O | 0.671158916 |
| CC(=O)c1cc(O)ccc1O | 0.533859754 |
| COc1ccc(O)cc1OC | 0.53322935 |
| Oc1ccc(OC2CCCCO2)cc1 | 0.458812473 |
| O=C1c2c(O)cccc2C(C2OC(CO)C(O)C(O)C2O)c2cc(CO)cc(O)c21 | 0.59572215 |
| OC[C@H]1O[C@@H](Oc2ccc(O)cc2)[C@H](O)[C@@H](O)[C@@H]1O | 0.671158916 |
| COc1ccccc1C=CC(=O)O | 0.587942884 |
| O=c1c(-c2ccc(O)cc2)coc2c([C@@H]3O[C@H](CO[C@@H]4OC[C@](O)(CO)[C@H]4O)[C@@H](O)[C@H](O)[C@H]3O)c(O)ccc12 | 0.51220356 |
| C[C@@]12C[C@@]3(O)O[C@@H](O1)[C@]1(COC(=O)c4ccc(O)cc4)[C@H]3C[C@@]12O[C@@H]1O[C@H](COC(=O)c2ccccc2)[C@@H](O)[C@H](O)[C@H]1O | 0.477180104 |
| COc1cc(O)c2c(=O)c(OC)c(-c3ccc(OC)c(OC)c3)oc2c1 | 0.579770377 |
| OCC1OC(Oc2ccc3c(c2)OCC2c4cc5c(cc4OC32)OCO5)C(O)C(O)C1O | 0.545420434 |
| COc1cc(O)cc(OC)c1 | 0.576577487 |
| O=C(/C=C/c1ccc(O)cc1)c1ccc(O[C@@H]2O[C@H](CO)[C@@H](O)[C@H](O)[C@H]2O[C@@H]2OC[C@](O)(CO)[C@H]2O)cc1O | 0.470967988 |
| COc1ccc(-c2coc3cc(O)ccc3c2=O)cc1O | 0.580389978 |
| CC(=O)Cc1cc(=O)c2c(C)cc(O)c([C@@H]3O[C@H](CO)[C@@H](O)[C@H](O)[C@H]3O)c2o1 | 0.580136796 |
| COC(=O)[C@@H](Cc1ccc(O)c(O)c1)OC(=O)/C=C/c1ccc(O)c(O)c1 | 0.563155106 |
| O=C1c2c(O)cc(O)cc2O[C@H](c2ccc(O)c(O)c2)[C@H]1O | 0.618513797 |
| CC(=O)CCc1ccccc1 | 0.576743152 |
| CC1(C)C2CC[C@]3(C)[C@H](CC=C4[C@H]5C[C@@]6(C)C(=O)OC6[C@H](O)[C@]5(C)CC[C@]43C)[C@@]2(C)CC[C@@H]1O | 0.896333067 |
| O=C(O)CCc1ccc(O)cc1O | 0.57639498 |
| Cc1cc(=O)c2c(O)c3c(cc2o1)OC(C)(C)[C@@H](O[C@@H]1O[C@H](CO)[C@@H](O)[C@H](O)[C@H]1O)C3 | 0.574502828 |
| O=C(/C=C/c1ccc(O)cc1)NCCCCN(CCCNC(=O)/C=C/c1ccc(O)cc1)C(=O)/C=C/c1ccc(O)cc1 | 0.39303849 |
| COc1cc2c(cc1OC)C1Cc3ccc(OC)c(OC)c3CN1CC2 | 0.463609805 |
| C/C=C1/CN2CCc3c([nH]c4ccccc34)[C@@H]2C[C@@H]1/C(=C/OC)C(=O)OC | 0.621233088 |
| COc1cc2c3c(c1OC)-c1ccccc1C[C@H]3N(C)CC2 | 0.497223155 |
| CC(C)=CCc1c(O)cc2oc3cc(O)c(O)c(CC=C(C)C)c3c(=O)c2c1O | 0.563211693 |
| O=c1c(-c2ccc(O)cc2)coc2c([C@@H]3O[C@H](CO)[C@@H](O)[C@H](O)[C@H]3O)c(O)ccc12 | 0.609351234 |
| COc1cc2c(cc1OC)[C@@H]1Cc3ccc(OC)c(OC)c3CN1CC2 | 0.463609805 |
| COc1ccc2c(c1OC)CN1CCc3cc4c(cc3C1C2)OCO4 | 0.489240612 |
| COc1cc(/C=C/C(=O)OCCc2ccc(O)cc2)ccc1O | 0.693229145 |
| COc1cc([C@@H]2Oc3ccc([C@H]4Oc5cc(O)cc(O)c5C(=O)[C@@H]4O)cc3O[C@H]2CO)ccc1O | 0.527064328 |
| C=C1[C@@H](O)[C@]2(O)OC[C@]34[C@H]2[C@@]2(C)[C@H](O)C(=O)C=C(C)[C@@H]2C[C@H]3OC(=O)C[C@@H]14 | 0.599792866 |
| COC(=O)c1c(C)cc(O)c(C)c1O | 0.556374615 |
| CC(C)c1ccc2c(c1O)CC[C@H]1C3=C(CC[C@]21C)C(=O)OC3 | 0.586480843 |
| COc1ccc(/C=C/C(=O)/C=C(O)/C=C/c2ccc(OC)c(OC)c2)cc1OC | 0.603700652 |
| CC(C)(O)[C@@H]1[C@H]2OC(=O)[C@@H]1[C@]1(O)C[C@H]3O[C@]34C(=O)O[C@H]2[C@]14C | 0.589505577 |
| COc1ccc(-c2coc3cc(O)c(OC)c(O)c3c2=O)cc1 | 0.597028668 |
| O=C1OC(=O)C2C3CCC(O3)C12 | 0.448715077 |
| O=C1OC(=O)C2C3CCC(O3)C12 | 0.448715077 |
| O=C1c2ccccc2-n2c1nc1ccccc1c2=O | 0.598198407 |
| O=c1c(O)c(-c2ccc(O)c(O)c2)oc2cc(O)c(O)c(O)c12 | 0.626542368 |
| COc1c(O)cc2oc(-c3ccc(O)cc3)cc(=O)c2c1O | 0.615933373 |
| CC(=O)C=Cc1ccc(O)c(O)c1 | 0.512228982 |
| CC(C)=CCCC(C)CCO | 0.617720567 |
| COc1ccc(C=CC(=O)O)cc1OC | 0.662941983 |
| COc1ccc(/C=C/c2cc(OC)cc(OC)c2)cc1 | 0.622025427 |
| COc1c(O)cc2c(c1OC)-c1ccc(OC)c(=O)cc1[C@@H](NC(C)=O)CC2 | 0.502751535 |
| COc1cc(C(=O)OC2C3C=COC(OC4OC(CO)C(O)C(O)C4O)C3C3(CO)OC23)ccc1O | 0.48118672 |
| COc1cc([C@H]2OC[C@H]3[C@@H]2CO[C@H]3c2ccc(OC)c(OC)c2)ccc1O | 0.515195619 |
| O=C(c1ccc(O)c(O)c1)c1c(O)cc(O)cc1O | 0.656994997 |
| COc1ccc(-c2coc3cc(O[C@@H]4O[C@H](CO)[C@@H](O)[C@H](O)[C@H]4O)ccc3c2=O)cc1O | 0.542933794 |
| CC(=O)O[C@H]1C[C@]2(O)[C@@H]3CC[C@@H]4C[C@@H](O)CC[C@]4(C)[C@H]3CC[C@]2(C)[C@H]1c1ccc(=O)oc1 | 0.650328911 |
| COc1ccc(CCC(=O)c2c(O)cc(O[C@@H]3O[C@H](CO)[C@@H](O)[C@H](O)[C@H]3O[C@@H]3O[C@@H](C)[C@H](O)[C@@H](O)[C@H]3O)cc2O)cc1O | 0.457349198 |
| CC(=O)c1ccc(O[C@@H]2O[C@H](CO)[C@@H](O)[C@H](O)[C@H]2O)cc1 | 0.708263497 |
| COc1ccc(O)c(C(C)(C)C)c1 | 0.516884142 |
| O=c1c(O[C@@H]2OC[C@@H](O)[C@H](O)[C@H]2O)c(-c2ccc(O)c(O)c2)oc2cc(O)cc(O)c12 | 0.505858713 |
| O=C(/C=C/c1ccc(O)c(O)c1)O[C@@H]1C[C@@](OC(=O)/C=C/c2ccc(O)c(O)c2)(C(=O)O)C[C@@H](O)[C@@H]1O | 0.473955327 |
| COc1cc(O)c2c(=O)cc(-c3ccccc3)oc2c1OC | 0.581029509 |
| CCOC(=O)C=Cc1ccc(O)c(OC)c1 | 0.736874532 |
| C[S+]([O-])CCCCC(=NOS(=O)(=O)O)S[C@@H]1O[C@H](CO)[C@@H](O)[C@H](O)[C@H]1O | 0.618531565 |
| C[C@]12C[C@@H](O)[C@@H]3C[C@@]1(O[C@@H]1O[C@H](CO)[C@@H](O)[C@H](O)[C@H]1O)[C@]3(COC(=O)c1ccccc1)C(=O)O2 | 0.533551002 |
| Cc1cc(O)c(C(C)C)cc1O | 0.501233461 |
| O=c1c(O[C@@H]2O[C@H]([C@H](O)CO)[C@H](O)[C@H]2O)c(-c2ccc(O)c(O)c2)oc2cc(O)cc(O)c12 | 0.490347985 |
| O=C1C[C@@H](c2ccc(O[C@@H]3O[C@H](CO)[C@@H](O)[C@H](O)[C@H]3O)cc2)Oc2cc(O)ccc21 | 0.675435815 |
| CC1(C)CCC2(C(=O)O)C(O)CC3(C)C(=CCC4C5(C)CCC(O)C(C)(C)C5CCC43C)C2C1 | 0.883130956 |
| C=C1C(=O)O[C@H]2C[C@@H](C)[C@@H]3CCC(=O)[C@@]3(C)[C@@H](OC(C)=O)[C@H]12 | 0.576384122 |
| CCC1CN2CCC3(C(=O)Nc4ccccc43)C2CC1C(=COC)C(=O)OC | 0.521786019 |
| CCN1C[C@]2(C)CC[C@H](O)C34C1C(C[C@@H]32)[C@@]1(O)C[C@H](OC)[C@H]2C[C@@H]4[C@@H]1[C@H]2O | 0.747278998 |
| COc1cc(CCc2cc(OC)c(O)c(OC)c2)ccc1O | 0.522827063 |
| COc1ccc(/C=C/C(=O)c2ccc3c(c2O)C=CC(C)(C)O3)cc1 | 0.611647358 |
| c1cc2c(c3c1CC1c4cc5c(cc4CCN1C3)OCO5)OCO2 | 0.454582901 |
| C=CCc1cc(OC)c(O[C@@H](C)[C@H](O)c2ccc(O)c(OC)c2)c(OC)c1 | 0.602573615 |
| O=C(/C=C/c1ccc(O)c(O)c1)OCCc1ccccc1 | 0.745470077 |
| COC(=O)C1=CO[C@@H](C)[C@H]2CN3CC[C@]4(C(=O)Nc5ccccc54)[C@@H]3C[C@H]12 | 0.561011307 |
| C[C@H]1O[C@@H](O[C@H]2[C@H](Oc3c(-c4ccc(O)cc4)oc4cc(O)cc(O)c4c3=O)O[C@@H](CO)[C@H](O)[C@H]2O)[C@H](O)[C@@H](O)[C@@H]1O | 0.438681196 |
| O=C(N[C@@H](Cc1ccccc1)C(=O)N[C@H](CO)Cc1ccccc1)c1ccccc1 | 0.592405036 |
| C=C1CCC2C(C)(CO)C(O)CCC2(C)C1CCC1=CCOC1=O | 0.674322023 |
| COc1ccc(-c2cc(=O)c3c(O)cc(O)c(-c4cc(-c5cc(=O)c6c(O)cc(OC)cc6o5)ccc4OC)c3o2)cc1 | 0.436569708 |
| COc1ccc(-c2coc3cc(O)cc(O)c3c2=O)cc1O | 0.629156899 |
| C[S+]([O-])C=CCCN=C=S | 0.582248485 |
| OC[C@H]1O[C@@H](Oc2cc3c(O)cc(O)cc3[o+]c2-c2ccc(O)c(O)c2)[C@H](O)[C@@H](O)[C@@H]1O.[Cl-] | 0.549652112 |
| COc1cc(CCC(C)=O)ccc1O | 0.562258186 |
| CC(C)[C@]12O[C@H]1[C@@H]1O[C@]13[C@]1(O[C@H]1C[C@H]1C4=C(CC[C@@]13C)C(=O)OC4)[C@@H]2O | 0.661568277 |
| CCC(C)=CCCC(C)(O)C1CCC2(C)C3CC(OC4OC(CO)C(O)C(O)C4OC4OC(C)C(O)C(O)C4O)C4C(C)(C)C(O)CCC4(C)C3CC(O)C12 | 0.818036364 |
| COc1cc2c(c(OC)c1OC)-c1c(cc3c(c1OC)OCO3)CC(C)C(C)(O)C2OC(=O)c1ccccc1 | 0.501219928 |
| CC1=C(C)C(=O)O[C@@H]([C@](C)(O)[C@H]2CC[C@H]3[C@@H]4[C@@H]5O[C@@H]5[C@@]5(O)CC=CC(=O)[C@]5(C)[C@H]4CC[C@]23C)C1 | 0.687208753 |
| COc1cccc2c1cc([N+](=O)[O-])c1c(C(=O)O)cc3c(c12)OCO3 | 0.599629877 |
| COc1c(C)c(O)cc2c1C(=O)c1ccccc1C2=O | 0.54336091 |
| COc1c(/C=C/C(=O)c2ccc(O)c(CC=C(C)C)c2)ccc(O)c1O | 0.56293797 |
| O=C(/C=C/c1ccc(O[C@@H]2O[C@H](CO)[C@@H](O)[C@H](O)[C@H]2O[C@@H]2OC[C@](O)(CO)[C@H]2O)cc1)c1ccc(O)cc1O | 0.477478405 |
| COc1c(O)c(C)cc2c1C(=O)c1c(O)cccc1C2=O | 0.512463398 |
| CC1(C)CC[C@]2(C)CC=C3[C@]4(C)CC[C@H]5C(C)(C)[C@@H](O)CC[C@]5(C)[C@H]4CC[C@@]3(C)[C@@H]2C1 | 0.830058687 |
| CC1=C(CO)C(=O)O[C@@H]([C@@H](C)[C@H]2CC[C@H]3[C@@H]4C[C@H]5O[C@]56[C@@H](O)C=CC(=O)[C@]6(C)[C@H]4CC[C@]23C)C1 | 0.641575938 |
| C=C(C)[C@@H]1CCC2=CCC[C@@H](C)[C@]2(C)C1 | 0.604763905 |
| C/C=C(/C)C(=O)O[C@H]1[C@@H](OC(C)=O)c2c(ccc3ccc(=O)oc23)OC1(C)C | 0.573205272 |
| COc1cc2c(cc1OC)C(Cc1ccc(Oc3cc(CC4c5cc(OC)c(OC)cc5CCN4C)ccc3O)cc1)N(C)CC2 | 0.388345518 |
| CCCCCCCCCCCCCCCCCCCCCCCCCCCCCCC | 0.482006851 |
| COc1ccc(CCc2cc(OC)c(O)c(OC)c2)cc1OC | 0.500935599 |
| C[C@@H]1O[C@@H](O[C@H]2[C@H](Oc3cc(O)c(C(=O)CCc4ccc(O)cc4)c(O)c3)O[C@H](CO)[C@@H](O)[C@@H]2O)[C@H](O)[C@H](O)[C@H]1O | 0.454868542 |
| COc1cc2c(cc1O)C(=O)[C@@H](C)[C@@H](C)[C@@H]2c1ccc(OC)c(OC)c1 | 0.562137243 |
| CC(=O)OCC1=C[C@H](O)[C@@H]2C(C(=O)O)=CO[C@@H](O[C@@H]3O[C@H](CO)[C@@H](O)[C@H](O)[C@H]3O)[C@H]12 | 0.573434389 |
| O=c1cc(-c2ccc(O)c(O)c2)oc2cc(O)ccc12 | 0.616047491 |
| COc1c(O)cc2oc3cc(O)c4c(c3c(=O)c2c1CC=C(C)C)OC(C)(C)CC4 | 0.576780051 |
| C[C@@H]1[C@H]2c3cc4c(cc3O[C@@]35OCOC3=CC(=O)[C@H](C[C@H]1C)[C@@H]25)OCO4 | 0.559244007 |
| CC1=CC(=O)[C@@H](O)[C@@]2(C)[C@H]1CC(=O)[C@@]1(C)[C@H]3C(=O)O[C@H]([C@@H]3C)[C@H](O)[C@@H]12 | 0.636906346 |
| C[C@@H]1O[C@@H](O[C@@H]2[C@@H](O)[C@H](OCCc3ccc(O)c(O)c3)O[C@H](COC(=O)/C=C/c3ccc(O)c(O)c3)[C@H]2O)[C@H](O)[C@H](O)[C@H]1O | 0.497983387 |
| C=C(C)C(=O)O[C@H]1CC2=C[C@@H](C/C(C)=C\[C@H]3OC(=O)C(=C)[C@H]13)OC2=O | 0.551017798 |
| O=C(/C=C/c1ccc(O)c(O)c1)O[C@]1(C(=O)O)C[C@@H](O)[C@H](O)[C@H](O)C1 | 0.611658627 |
| COc1cc2oc(=O)ccc2cc1CC=C(C)C | 0.653005548 |
| COc1cc(O)c2c(=O)c(-c3ccc(O)cc3)coc2c1 | 0.608523151 |
| C=CCC1=C[C@]2(OC)C(=O)C(OC)(OC)[C@H]1[C@@H]1C(OC)(OC)C(=O)C(OC)=C[C@@]12CC=C | 0.514819793 |
| COC[C@@H](Cc1ccc(OC)c(OC)c1)[C@@H](COC)Cc1ccc(OC)c(OC)c1 | 0.415298903 |
| O=c1c(OC2OC(CO)C(O)C(O)C2O)c(-c2ccc(O)cc2)oc2cc(O)cc(O)c12 | 0.551009807 |
| C=C1[C@H](OC(=O)c2ccccc2)C[C@H]2[C@@H](/C=C(\C)C(=O)[C@@]3(OC(C)=O)C[C@H](C)[C@H](OC(=O)c4ccccc4)[C@@H]3[C@H]1OC(C)=O)C2(C)C | 0.532858437 |
| COC(=O)[C@]1(O)C[C@@H](O)[C@@H](O)[C@H](OC(=O)C=Cc2ccc(O)c(O)c2)C1 | 0.570883624 |
| COc1c(O[C@@H]2O[C@H](CO)[C@@H](O)[C@H](O)[C@H]2O)cc2oc(-c3ccc(O)cc3)cc(=O)c2c1O | 0.532746695 |
| COc1ccc(/C=C/C(=O)c2c(O)cc(O[C@@H]3O[C@H](CO[C@@H]4O[C@@H](C)[C@H](O)[C@@H](O)[C@H]4O)[C@@H](O)[C@H](O)[C@H]3O)cc2OC)cc1O | 0.449206919 |
| C=C(C)[C@H](O)[C@@H](O)C[C@@H](C)C1=C2C[C@H](O)[C@H]3[C@@]4(C)CCC(=O)C(C)(C)[C@@H]4CC[C@]3(C)[C@@]2(C)CC1 | 0.888442696 |
| C=CC1CNC(=S)O1 | 0.606680894 |
| C=C1CC[C@@H]2[C@](C)(COC(=O)CCC(=O)O)[C@H](OC(=O)CCC(=O)O)CC[C@@]2(C)[C@@H]1/C=C/C1=CCOC1=O | 0.424061867 |
| COC(=O)C1=CO[C@@H](O[C@@H]2O[C@H](CO)[C@@H](O)[C@H](O)[C@H]2O)[C@@H]2C(CO)=CC[C@H]12 | 0.659801152 |
| O=c1c2cc(O)c(O)cc2oc2c([C@@H]3O[C@H](CO)[C@@H](O)[C@H](O)[C@H]3O)c(O)cc(O)c12 | 0.565772179 |
| CC(C)=C1C/C=C(/C)CC/C=C(/C)CC1=O | 0.652487843 |
| COC(=O)C[C@](O)(CCC(C)(C)O)C(=O)O[C@@H]1C(OC)=C[C@]23CCCN2CCc2cc4c(cc2[C@H]13)OCO4 | 0.422453683 |
| COC(=O)C1=COC(O[C@@H]2O[C@H](CO)[C@@H](O)[C@H](O)[C@H]2O)C(O)(CC(=O)O)C1CC(=O)O | 0.527912438 |
| COC1=C[C@]23CCCN2CCc2cc4c(cc2[C@@H]3[C@@H]1O)OCO4 | 0.51052253 |
| CN1Cc2c(ccc3c2OCO3)[C@@H]2[C@H]1c1cc3c(cc1C[C@@H]2O)OCO3 | 0.514257858 |
| COc1cc2ccc(=O)oc2c(OC)c1O[C@H]1O[C@H](CO)[C@@H](O)[C@H](O)[C@H]1O | 0.657816876 |
| O=c1ccc2ccccc2o1 | 0.555395525 |
| CC(C)[C@@H]1[C@H]2OC(=O)[C@@H]1[C@@H]1CC[C@@H]3CN(C)[C@H]2[C@@]31C | 0.730084219 |
| O=C1CC(c2ccccc2)Oc2cc(O)c(O)c(O)c21 | 0.602857871 |
| O=C(CCc1ccc(O)cc1)c1c(O)cc(O)cc1O[C@@H]1O[C@H](CO)[C@@H](O)[C@H](O)[C@H]1O | 0.586660705 |
| C=C(C)[C@H](CC=C(C)C)Cc1c(O)cc(O)c2c1O[C@H](c1ccc(O)cc1OC)CC2=O | 0.546311121 |
| COc1ccc(-c2cc(=O)c3c(O)cc(O)cc3o2)cc1O | 0.612265233 |
| CC[C@@H]1[C@H]2CCCCN3[C@H]2[C@@H](C[C@H]3[C@@H]2C[C@H](C)C(=O)O2)[C@H]2[C@@H]1OC(=O)[C@H]2C | 0.663473264 |
| CC(=O)OC1C(OCCc2ccc(O)c(O)c2)OC(COC2OC(CO)C(O)C(O)C2O)C(OC(=O)/C=C/c2ccc(O)c(O)c2)C1OC1OC(C)C(O)C(O)C1O | 0.380315302 |
| COc1cc2c(cc1OC)C1Cc3ccc(OC)c(OC)c3CN1CC2.Cl | 0.535292762 |
| C=C[C@H]1CN2CCc3c([nH]c4ccccc34)[C@H]2C[C@@H]1/C(=C\OC)C(=O)OC | 0.600658493 |
| OC1c2ccccc2-c2ccccc21 | 0.445360926 |
| COc1cc(OC)c2c(=O)cc(-c3ccc(O)c(O)c3)oc2c1 | 0.593864567 |
| CCOC(=O)Oc1c(OC)cc(C(=O)O[C@@H]2C[C@@H]3CN4CCc5c([nH]c6cc(OC)ccc56)[C@H]4C[C@@H]3[C@H](C(=O)OC)[C@H]2OC)cc1OC | 0.378330764 |
| COc1cc2c(cc1OC)[C@@H]1Cc3ccc(OC)c(OC)c3CN1CC2 | 0.463609805 |
| C=C[C@H]1[C@H](O[C@@H]2O[C@H](CO)[C@@H](O)[C@H](O)[C@H]2O)OC=C(C(=O)O)[C@H]1CC(=O)O | 0.596543434 |
| O=c1oc2cc(O)cc(O)c2c2oc3cc(O)c(O)cc3c12 | 0.575081203 |
| Cc1cc(O)c2c(c1)C(=O)c1cccc(O[C@@H]3O[C@H](CO)[C@@H](O)[C@H](O)[C@H]3O)c1C2=O | 0.52247785 |
| Cc1c(C)c2c(c(C)c1O)CC[C@@](C)(CCC[C@H](C)CCC[C@H](C)CCCC(C)C)O2 | 0.624063506 |
| Cc1cc(C(C)(C)C)c(O)c(C(C)(C)C)c1 | 0.64064453 |
| C=C1C(=O)OC23C1CCC(C)C2CCC1(C)OC13 | 0.676698404 |
| CC(=O)/C=C/c1ccccc1 | 0.600064724 |
| OC[C@H]1O[C@@H](Oc2cc(O)cc(/C=C/c3ccc(O)cc3)c2)[C@H](O)[C@@H](O)[C@@H]1O | 0.653314248 |
| Cc1coc2c1C(=O)C(=O)c1c-2ccc2c(C)cccc12 | 0.528870732 |
| O=c1cc(-c2ccc(Oc3c(O)cc4oc(-c5ccc(O)cc5)cc(=O)c4c3O)cc2)oc2cc(O)cc(O)c12 | 0.414984677 |
| CCCCCC/C=C\CCCCCCCc1cccc(O)c1C(=O)O | 0.511777278 |
| COc1cc([C@H]2Oc3c(OC)cc4ccc(=O)oc4c3O[C@@H]2CO)ccc1O | 0.607715662 |
| CC1=C(CCC2=CCOC2=O)[C@]2(C)CC[C@@H](O)[C@@](C)(CO)[C@H]2CC1 | 0.653979369 |
| C=C[C@@]1(C)CC(=O)[C@]2(O)[C@@]3(C)[C@@H](O)CCC(C)(C)[C@@H]3[C@H](OC(C)=O)[C@H](O)[C@@]2(C)O1 | 0.630186572 |
| COc1cc(C[C@@H](CO)[C@H](CO)Cc2ccc(O)c(OC)c2)ccc1O | 0.570534615 |
| COc1cc(O[C@@H]2O[C@H](CO)[C@@H](O)[C@H](O)[C@H]2O)c2c(c1)C(=O)c1cc(C)cc(O)c1C2=O | 0.487630217 |
| COCC12CCC(OC)C34C5CC6(O)C(OC)C(O)C(OC(C)=O)(C5C6OC(=O)c5ccccc5)C(C(OC)C13)C4N(C)C2 | 0.59119952 |
| O=C(CCc1ccc(O)cc1)c1c(O)cc(O)c([C@@H]2O[C@H](CO)[C@@H](O)[C@H](O)[C@H]2O)c1O | 0.585815169 |
| COc1cc([C@H]2Oc3c(OC)cc(CCCO)cc3[C@@H]2CO)ccc1O | 0.531677094 |
| COc1cc(O)c2c(c1)O[C@H](c1ccccc1)CC2=O | 0.597242253 |
| C[C@@H]1CC[C@]2(C(=O)O)CC[C@]3(C)C(=CC[C@@H]4[C@@]5(C)CC[C@H](O)[C@](C)(C(=O)O)[C@@H]5CC[C@]43C)[C@@H]2[C@]1(C)O | 0.774033871 |
| CCC1CC2CN3CCc4c([nH]c5ccccc45)C(C(=O)OC)(C2)C13.O=C(O)C(O)C(O)C(=O)O | 0.52224374 |
| Oc1ccc(O)c(O)c1 | 0.519368147 |
| C/C=C(/C)C(=O)O[C@H]1[C@@H](OC(=O)CC(C)C)c2c(ccc3ccc(=O)oc23)OC1(C)C | 0.570605486 |
| CC(=O)OCC12C(OC(C)=O)C(OC(C)=O)C3C(OC(C)=O)C14OC3(C)COC(=O)c1cccnc1CCC(C)C(=O)OC(C(OC(=O)c1ccoc1)C2OC(C)=O)C4(C)O | 0.499144281 |
| C/C=C(\C)C(=O)OC1c2cc3c(c4c2C2(CO4)C(=O)C(OC)=C(OC)C=C2CC(C)C1C)OCO3 | 0.483499272 |
| COc1ccc(C(C)=O)c(O)c1O | 0.497942065 |
| COc1ccc2cc1Oc1ccc(cc1)C[C@H]1c3cc(c(OC)cc3CCN1C)Oc1c(OC)c(OC)cc3c1[C@H](C2)N(C)CC3 | 0.4102144 |
| O=C1C[C@@H](c2ccc(O)cc2)Oc2cc(O)ccc21 | 0.609352549 |
| C[C@@]12C[C@H]3OC(=O)[C@@H]1CO[C@]14O[C@@]5([C@H]2C1=O)[C@@]3(C)OC(=O)[C@@]5(O)CC[C@H]1[C@H]4C[C@H]2O[C@]23CC=CC(=O)[C@]13C | 0.635609413 |
| CC1CCC(C(C)C)C(O)C1 | 0.568555404 |
| CC/C=C\C[C@H]1C(=O)CC[C@@H]1CC(=O)O | 0.618810996 |
| C/C=C(/C)C(=O)O[C@@H]1[C@H](OC(=O)CC(C)C)c2c(ccc3ccc(=O)oc23)OC1(C)C | 0.570605486 |
| COc1cc(C2Oc3ccc(C4Oc5cc(O)cc(O)c5C(=O)C4O)cc3OC2CO)ccc1O | 0.527064328 |
| COc1cc(O)c(C(=O)/C=C/c2ccc(O)cc2)cc1CC=C(C)C | 0.590995154 |
| COc1ccc2c(c1)O[C@@H]1c3ccc(O)cc3OC[C@H]21 | 0.492107632 |
| O=C1C[C@@H](c2ccc(O)cc2)Oc2cc(O)ccc21 | 0.609352549 |
| O=C(c1ccc(O)cc1)c1c(O)cc(O)cc1O | 0.701198643 |
| CC(C)=CCc1cc2c(cc1O)O[C@H](c1ccc(O)cc1)CC2=O | 0.557757304 |
| Cn1cc(C[C@H](N)C(=O)O)c2ccccc21 | 0.699604441 |
| O=S(=O)([O-])O.c1c2c(cc3c1OCO3)-c1cc3ccc4c(c3c[n+]1CC2)OCO4 | 0.535334044 |
| c1c2c(cc3c1OCO3)-c1cc3ccc4c(c3c[n+]1CC2)OCO4 | 0.468495637 |
| COc1ccc(C(=O)/C=C/c2ccc(O)cc2)c(O)c1CC=C(C)C | 0.602782994 |
| COc1ccc2c3c([nH]c2c1)C(C)NCC3 | 0.481886762 |
| C=C1CC[C@@H]2[C@](C)(CO)CCC[C@@]2(C)[C@@H]1CCC1=CCOC1=O | 0.686578509 |
| COc1ccc(/C=C/C(=O)c2c(O)cc(OC)cc2OC)cc1 | 0.699155114 |
| OC[C@H]1O[C@@H](OC/C=C/c2ccccc2)[C@H](O)[C@@H](O)[C@@H]1O | 0.672731469 |
| COc1cc2oc(-c3ccccc3)cc(=O)c2c(OC)c1OC | 0.600323503 |
| CC1(C)OC(=O)C=C[C@]2(C)[C@H]3CC[C@@]4(C)[C@H](c5ccoc5)OC(=O)[C@H]5O[C@]54[C@]3(C)C(=O)C[C@@H]12 | 0.62130272 |
| CCCCCCCCCCCCCCC(O)C(O)C(N)CO | 0.508040035 |
| CCCCCCCc1cc(=O)c2ccccc2n1C | 0.540956751 |
| C=C[C@@H]1C2=CCOC(=O)C2=CO[C@H]1O[C@@H]1O[C@H](CO)[C@@H](O)[C@H](O)[C@H]1O | 0.700513779 |
| CC1(C)Oc2cc3oc(-c4cc(O)cc(O[C@@H]5OC[C@@H](O)[C@H](O)[C@H]5O)c4)cc3cc2CC1O | 0.500364178 |
| COc1c(OC(C)=O)cc(O)c2c(=O)cc(-c3ccccc3)oc12 | 0.585077901 |
| O=c1ccc2ccc3occc3c2o1 | 0.465008763 |
| CCCCCC/C=C\CCCCCCCCCC(=O)O | 0.572160088 |
| COC(=O)c1c2c(c3ccccc3c1O)OC(C)(C)CC2 | 0.602661712 |
| COc1cccc(O)c1O | 0.502370609 |
| CC1=CC(=O)[C@@H](O)[C@]2(C)[C@H]3[C@@H](O)[C@H](O)[C@@]4(CO)OC[C@@]35[C@@H](C[C@@H]12)OC(=O)[C@H](O)[C@]54O | 0.560150563 |
| C=C1C(=O)OC2C1CCC(=C)C1CCC(=C)C12 | 0.655883554 |
| CCCCCc1cc(O)cc(O)c1 | 0.574283986 |
| C=CCSC[C@H](NC(=O)CC[C@H](N)C(=O)O)C(=O)O | 0.691400651 |
| COc1cc(CCC(O)CCCCc2ccccc2)ccc1O | 0.510496187 |
| COc1cc(/C=C/C(=O)NCCc2c[nH]c3ccc(O)cc23)ccc1O | 0.604929225 |
| O=C(C=Cc1ccc(O)cc1)OCC1OC(Oc2c(-c3ccc(O)cc3)oc3cc(O)cc(O)c3c2=O)C(O)C(O)C1O | 0.451902462 |
| CCOCc1ccc(O)cc1 | 0.545051433 |
| CC(C)(O)C1CCC(C)(C2C(O)CC3(C)C4CC(O)C5C(C)(C)C(OC6OCC(O)C(O)C6OC6OC(CO)C(O)C(O)C6O)CCC56CC46CCC23C)O1 | 0.784461886 |
| O=c1cc(-c2ccccc2)oc2cc(O)cc(O)c12 | 0.590393516 |
| OCC1=C[C@@H](O)[C@]2(O[C@@H]3O[C@H](CO)[C@@H](O)[C@H](O)[C@H]3O)C=CO[C@@H](O[C@@H]3O[C@H](CO)[C@@H](O)[C@H](O)[C@H]3O)[C@H]12 | 0.494676395 |
| COc1ccc(-c2cc(=O)c3c(O)cc(O[C@@H]4O[C@H](CO)[C@@H](O)[C@H](O)[C@H]4O)cc3o2)cc1O | 0.531667403 |
| O=C1CCC[C@@H]2[C@H]3CCC[N+]4([O-])CCC[C@H](CN12)[C@@H]34 | 0.651251961 |
| O=c1c(-c2ccc(O)c(O)c2)coc2c([C@@H]3O[C@H](CO)[C@@H](O)[C@H](O)[C@H]3O)c(O)ccc12 | 0.566169644 |
| COc1cc2c(c(O)c1OC)-c1c(cc(OC)c(OC)c1OC)C[C@H](C)[C@H](C)C2 | 0.534716016 |
| O=c1cc(-c2ccccc2)oc2ccc3ccccc3c12 | 0.554823601 |
| CCCCC/C=C\C/C=C\CCCCCCCC(=O)OC | 0.515899546 |
| CC1=NCCc2c1[nH]c1cc(O)ccc21.Cl | 0.612841794 |
| COc1ccc([C@@H]2CC(=O)c3c(O)cc(O[C@@H]4O[C@H](CO)[C@@H](O)[C@H](O)[C@H]4O[C@@H]4O[C@@H](C)[C@H](O)[C@@H](O)[C@H]4O)cc3O2)cc1O | 0.439985295 |
| OC[C@H]1O[C@@H](Oc2cc3c(O)cc(O)cc3[o+]c2-c2ccc(O)c(O)c2)[C@H](O)[C@@H](O)[C@H]1O.[Cl-] | 0.549652112 |
| COC(=O)C1=CO[C@@H](O[C@@H]2O[C@H](CO[C@@H]3O[C@H](CO)[C@@H](O)[C@H](O)[C@H]3O)[C@@H](O)[C@H](O)[C@H]2O)[C@@H]2C(CO)=CC[C@H]12 | 0.473655336 |
| O=C(/C=C/c1ccc(O)c(O[C@@H]2O[C@H](CO)[C@@H](O)[C@H](O)[C@H]2O)c1)O[C@H](Cc1ccc(O)c(O)c1)C(=O)O | 0.467421927 |
| O=C(CCc1ccccc1)C[C@@H](O)/C=C/c1ccccc1 | 0.622933494 |
| COc1cc2c3c(c1O)-c1ccccc1C[C@@H]3N(C)CC2 | 0.492835839 |
| CC(C)(O)[C@H](O)COc1c2ccoc2cc2oc(=O)ccc12 | 0.67968079 |
| COc1cc(/C=C/C(=O)O[C@H]2[C@H](O)[C@@H](CO)O[C@@]2(CO)O[C@H]2O[C@H](CO)[C@@H](O)[C@H](O)[C@H]2O)cc(OC)c1O | 0.459421256 |
| CCCCCCCC/C=C\CCCCCCCC(=O)OC | 0.532846779 |
| O=c1cc(-c2ccc(O)cc2)oc2c([C@@H]3O[C@H](CO)[C@@H](O)[C@H](O)[C@H]3O)c(O)cc(O)c12 | 0.554714393 |
| COc1cc2c(c(OC)c1OC)-c1c(cc3c(c1OC)OCO3)[C@H](OC(C)=O)[C@H](C)[C@H](C)C2 | 0.502756812 |
| COc1cc(/C=C/C=O)ccc1O | 0.556758641 |
| Oc1cc(O)c2cc(O)c(-c3ccc(O)c(O)c3)[o+]c2c1.[Cl-] | 0.660854403 |
| COc1cc(O[C@@H]2O[C@H](CO[C@@H]3OC[C@@H](O)[C@H](O)[C@H]3O)[C@@H](O)[C@H](O)[C@H]2O)c2c(=O)cc(-c3ccc(O)cc3)oc2c1 | 0.437220147 |
| O=c1c(-c2ccccc2)coc2ccccc12 | 0.583237144 |
| COc1ccc(CC2c3cc(Oc4cc(CC5c6cc(O)c(OC)cc6CCN5C)ccc4O)c(OC)cc3CCN2C)cc1 | 0.399032877 |
| COCc1c(CO)cnc(C)c1O | 0.647792976 |
| COc1ccc(-c2cc(=O)c3c(O)cc(O[C@@H]4O[C@H](CO)[C@@H](O)[C@H](O)[C@H]4O[C@@H]4O[C@@H](C)[C@H](O)[C@@H](O)[C@H]4O)cc3o2)cc1O | 0.419673033 |
| CC1=C(C=O)C(C)(C)CC=C1 | 0.570429058 |
| COC(=O)/C(C)=C/C=C/C(C)=C/C=C/C=C(C)/C=C/C=C(\C)C(=O)OC | 0.480071682 |
| COc1cc([C@H]2OC[C@H]3[C@@H]2CO[C@@H]3c2ccc(O[C@@H]3O[C@H](CO)[C@@H](O)[C@H](O)[C@H]3O)c(OC)c2)ccc1O | 0.428541004 |
| O=c1cc(-c2ccc(O)cc2)oc2cc(O)c([C@@H]3O[C@H](CO)[C@@H](O)[C@H](O)[C@H]3O)c(O)c12 | 0.564480925 |
| COc1c(O[C@@H]2O[C@H](CO)[C@@H](O)[C@H](O)[C@H]2O)cc2oc(-c3ccc(O)c(O)c3)cc(=O)c2c1O | 0.519116201 |
| COc1cc(-c2oc3cc(O)cc(O)c3c(=O)c2OC2O[C@H](CO[C@@H]3O[C@@H](C)[C@H](O)[C@@H](O)[C@H]3O)[C@H](O)[C@H](O)[C@H]2O)ccc1O | 0.42780203 |
| C=C[C@]12CN(C)[C@@H]3C4CO[C@H](C[C@H]41)[C@]1(C(=O)Nc4ccccc41)[C@@H]32 | 0.60430154 |
| O=C(O)[C@H]1O[C@@H](Oc2cc(O)c3c(=O)cc(-c4ccccc4)oc3c2)[C@H](O)[C@@H](O)[C@@H]1O | 0.613270226 |
| CC1=CCC(C(C)C)=CC1 | 0.574628718 |
| COc1cc(C(=O)O)cc(OC)c1OC | 0.654200325 |
| CCCOC(=O)c1cc(O)c(O)c(O)c1 | 0.662682325 |
| CC1=C(C)C(=O)C(CCC(C)(O)CCCC(C)CCCC(C)CCCC(C)C)=C(C)C1=O | 0.492076923 |
| CC(=O)OCCc1ccc(O)c(O)c1 | 0.614590352 |
| C=CCc1ccc(OC)c(-c2ccc(OC)c(CC=C)c2)c1 | 0.53693628 |
| COc1cc(-c2oc3cc(O)cc(O)c3c(=O)c2O[C@@H]2O[C@H](CO)[C@@H](O)[C@H](O)[C@H]2O[C@@H]2O[C@@H](C)[C@H](O)[C@@H](O)[C@H]2O)ccc1O | 0.41500287 |
| COc1cc(O)c2c(=O)c(OC)c(-c3ccc(O)c(OC)c3)oc2c1 | 0.591148953 |
| COc1ccc(-c2oc3cc(OC)c(OC)c(O)c3c(=O)c2OC)cc1OC | 0.514986445 |
| CCOC(=O)C(C)=O | 0.606095434 |
| COc1cc(-c2oc3cc(O)cc(O)c3c(=O)c2O[C@@H]2O[C@H](CO[C@@H]3O[C@@H](C)[C@H](O)[C@@H](O)[C@H]3O)[C@@H](O)[C@H](O)[C@H]2O)ccc1O | 0.42780203 |
| Cc1cc(O)cc(OC2O[C@H](CO)[C@@H](O)[C@H](O)[C@H]2O)c1 | 0.694311557 |
| Cc1nc(C)c(C)nc1C | 0.600850515 |
| COc1cc(O)c2c(=O)cc(-c3ccc(O)c(O)c3)oc2c1 | 0.604804042 |
| O=c1cc(-c2ccc(O)cc2)oc2c([C@@H]3O[C@H](CO)[C@@H](O)[C@H](O)[C@H]3O)c(O)c([C@@H]3OC[C@@H](O)[C@H](O)[C@H]3O)c(O)c12 | 0.419842236 |
| O=Cc1c(O)cc(O)c(C=O)c1O | 0.535508731 |
| CS(=O)(=O)O.NCCCN(CCCCNCCCNC(=O)CCc1ccc(O)c(O)c1)C(=O)CCc1ccc(O)c(O)c1 | 0.35478437 |
| COC(=O)C1=CO[C@@H](O[C@@H]2O[C@H](CO)[C@@H](O)[C@H](O)[C@H]2O)[C@@H]2C(CO)=C[C@@H](O)[C@H]12 | 0.658372097 |
| CC(=O)O[C@@H]1C[C@H]2C(C)(C)C(=O)C=C[C@]2(C)[C@H]2CC[C@@]3(C)[C@H](c4ccoc4)OC(=O)[C@H]4O[C@]43[C@@]21C | 0.675420461 |
| C=C1C(=O)O[C@@H]2/C=C(/C)[C@@H](OC(C)=O)C/C=C(/COC(C)=O)C[C@@H](OC(=O)/C(C)=C/CO)[C@@H]12 | 0.43986592 |
| COc1ccc(-c2cc(=O)c3ccccc3o2)cc1OC | 0.605868222 |
| Cc1cc2c(C(C)C)c(O)c(O)c(C=O)c2c(O)c1-c1c(C)cc2c(C(C)C)c(O)c(O)c(C=O)c2c1O | 0.404712335 |
| CC(C)=CCC[C@@H](C(=O)O)[C@H]1CC[C@@]2(C)C3=CC[C@H]4C(C)(C)[C@@H](O)CC[C@]4(C)C3=CC[C@]12C | 0.805150277 |
| CCCCC/C=C/C/C=C/CCCCCCCc1cccc(O)c1C(=O)O | 0.493587119 |
| CC1(C)CC[C@]2(C)CC=C3[C@]4(C)CC[C@H]5C(C)(C)C(=O)CC[C@]5(C)[C@H]4CC[C@@]3(C)[C@@H]2C1 | 0.799491034 |
| COc1ccc(-c2coc3cc(O[C@@H]4O[C@H](CO[C@@H]5OC[C@@H](O)[C@H](O)[C@H]5O)[C@@H](O)[C@H](O)[C@H]4O)c(OC)c(O)c3c2=O)cc1 | 0.421523992 |
| COc1cc(/C=C/C(=O)O[C@H]2[C@H](O)[C@@H](CO)O[C@@]2(CO)O[C@H]2O[C@H](COC(=O)c3ccc(O)cc3)[C@@H](O)[C@H](O)[C@H]2O)cc(OC)c1O | 0.39696644 |
| COc1cc(/C=C/C(=O)O[C@H]2[C@H](O)[C@@H](CO)O[C@@]2(CO)O[C@H]2O[C@H](CO)[C@@H](O)[C@H](O)[C@H]2O)cc(OC)c1OC | 0.453115826 |
| O=Cc1cccc(O)c1 | 0.566108845 |
| COc1cc(/C=C/C(=O)O[C@H]2[C@H](O)[C@@H](CO)O[C@@]2(CO)O[C@H]2O[C@H](CO)[C@@H](O)[C@H](O)[C@H]2O)ccc1O | 0.489620843 |
| O=C(/C=C/c1ccc(O)c(O)c1)OC[C@H]1O[C@@H](OCCc2ccc(O)c(O)c2)[C@H](O)[C@@H](O)[C@@H]1O | 0.562599332 |
| O=C1CC(c2ccccc2)Oc2ccccc21 | 0.588195326 |
| O=C(O)c1cc2c(c3c1c([N+](=O)[O-])cc1ccccc13)OCO2 | 0.626630795 |
| O=C1C[C@H](c2ccc(O)cc2)Oc2cc(O[C@@H]3O[C@H](CO)[C@@H](O)[C@H](O)[C@H]3O)cc(O)c21 | 0.599497491 |
| COc1c2c(cc3c1-c1c(cc4c(c1OC)OCO4)CC(C)C(C)C3)OCO2 | 0.536843231 |
| COc1cc(O)c2c(=O)cc(-c3ccc(O)cc3)oc2c1 | 0.605102393 |
| O=c1cc(-c2ccccc2)oc2c(O)c(O)cc(O)c12 | 0.613558319 |
| O=C1c2ccccc2C(=O)c2ccccc21 | 0.590384985 |
| COc1ccc2c(c1OC)CN(C)CCc1cc3c(cc1C(=O)C2)OCO3 | 0.519615021 |
| CCN1C[C@]2(COC)CC[C@H](OC)C34C1C(C[C@@H]32)[C@@]1(O)C[C@H](OC)[C@H]2C[C@@H]4[C@@H]1[C@H]2O | 0.728049732 |
| O=C1CC(c2ccc(O)cc2)Oc2cc(O)cc(O)c21 | 0.625509927 |
| C=C1C[C@@]23CC[C@H]4[C@@](C)(CCC[C@@]4(C)C(=O)O)[C@@H]2CC[C@@H]1C3 | 0.721258182 |
| CC1(C)OC1COc1c2ccoc2cc2oc(=O)ccc12 | 0.656228666 |
| COc1cc(OC)c2c(=O)cc(-c3ccccc3)oc2c1 | 0.622836777 |
| C/C1=C\CC[C@H](C)C(=O)C[C@@H](C(C)C)C(=O)C1 | 0.584628088 |
| O=c1cc(-c2ccccc2)oc2cc(O)ccc12 | 0.580593158 |
| COc1cc2oc(=O)ccc2c(OC)c1O | 0.608906133 |
| CC(C=O)=CC=CC(C)=CC=CC=C(C)C=CC=C(C)C=CC1=C(C)CCCC1(C)C | 0.551999337 |
| O=c1ccc2c(O)c3ccoc3cc2o1 | 0.497807959 |
| CCC1=N[C@H]2C[C@@]3(C(=O)N(OC)c4ccccc43)[C@H]3C[C@@H]1[C@@H]2CO3 | 0.564686996 |
| COc1cc2c(=O)c3c(O)c([C@@H]4O[C@H](CO[C@@H]5OC[C@](O)(CO)[C@H]5O)[C@@H](O)[C@H](O)[C@H]4O)c(O)cc3oc2cc1O | 0.430050051 |
| CC1=C[C@@]2(O)O[C@@]3(CC2=C(C)C)[C@@H](C)CC[C@@H]13 | 0.582137123 |
| C=CCC12C=C(OC)C(=O)C(OC)(OC)C1C1C(C=CC)=CC2(OC)C(=O)C1(OC)OC | 0.508706912 |
| CC(=O)OC(C)(C)C1CC=C(C)CC1 | 0.644082485 |
| Oc1ccc(/C=C/c2cc(O)cc3c2[C@@H](c2cc(O)cc(O)c2)[C@H](c2ccc(O)cc2)O3)cc1 | 0.499381749 |
| COc1ccc(-c2cc(=O)c3c(O)c(OC)c(OC)cc3o2)cc1O | 0.56011848 |
| O=C1c2ccccc2C(=O)c2c1ccc(O)c2O | 0.547623806 |
| O=C(/C=C/c1ccc(O)c2oc(-c3ccc(O)c(O)c3)cc12)O[C@H](Cc1ccc(O)c(O)c1)C(=O)O | 0.441957178 |
| COc1cc(C2Oc3ccc(C4Oc5cc(O)cc(O)c5C(=O)[C@@H]4O)cc3OC2CO)ccc1O | 0.527064328 |
| COC1=CC(=O)O[C@@H](CCc2ccc3c(c2)OCO3)C1 | 0.595893695 |
| COc1ccc(/C=C/c2cc(O)cc(O)c2)cc1O[C@@H]1O[C@H](CO)[C@@H](O)[C@H](O)[C@H]1O | 0.645114044 |
| Cc1ccc2nc(C)ccc2c1 | 0.559634911 |
| COc1cc2c3c(c1OC)-c1ccccc1C[C@H]3NCC2.Cl | 0.52648276 |
| C/C1=C/CC[C@]23O[C@@H]2[C@@H](OC3=O)c2c(C)coc2C1 | 0.61880855 |
| O=C(O)c1ccc2c(c1)OCO2 | 0.463653645 |
| CCCCCCCCCCCCCCCC(=O)NC(C)C | 0.566269029 |
| O=c1c(O)c(-c2ccc(O)c(O)c2)oc2cc(O[C@@H]3O[C@H](CO)[C@@H](O)[C@H](O)[C@H]3O)c(O)c(O)c12 | 0.51471982 |
| C/C(=C\C(=O)O)C(=O)O | 0.608262988 |
| C=C(C)[C@@H]1CC[C@]2(C(=O)O)CC[C@]3(C)[C@H](CC[C@@H]4[C@@]5(C)CC[C@H](O)[C@@](C)(CO)[C@@H]5CC[C@]43C)[C@@H]12 | 0.892896581 |
| O=C(/C=C/c1ccc(O)cc1)c1c(O)cc(O)cc1O | 0.690283984 |
| CC1(C)[C@H]2CC=C(C=O)[C@@H]1C2 | 0.54621202 |
| OCc1ccc(O[C@@H]2O[C@H](CO)[C@@H](O)[C@H](O)[C@H]2O)cc1 | 0.700377213 |
| COc1cc(O[C@@H]2O[C@H](CO[C@@H]3O[C@H](CO)[C@@H](O)[C@H](O)[C@H]3O)[C@@H](O)[C@H](O)[C@H]2O)c2c(O)c(C(C)=O)c(C)cc2c1 | 0.489282495 |
| O=c1cc(-c2ccccc2)oc2c([C@@H]3OC[C@H](O)[C@H](O)[C@H]3O)c(O)c([C@@H]3O[C@H](CO)[C@@H](O)[C@H](O)[C@H]3O)c(O)c12 | 0.442264421 |
| COc1cc(/C=C/C(=O)O)cc(OC)c1OC | 0.689726514 |
| CCCCCCCCCC(=O)OCC | 0.66800857 |
| C=C[C@H]1CNCC[C@H]1CCC(=O)c1ccnc2ccc(OC)cc12.Cl | 0.621094615 |
| O=c1cc(CCc2ccccc2)oc2ccccc12 | 0.598801601 |
| O=c1nc(CCCO)[nH]c2ccccc12 | 0.672893186 |
| CCCCCCCCCCCCCCCCCC(=O)NCc1ccccc1 | 0.500477231 |
| COc1ccc(/C=C/C(=O)c2c(OC)cc(OC)cc2OC)cc1 | 0.646354275 |
| COc1ccc(-c2coc3c([C@@H]4O[C@H](CO)[C@@H](O)[C@H](O)[C@H]4O)c(O)ccc3c2=O)cc1 | 0.622885028 |
| COc1ccc(C(C)=O)cc1O | 0.507609103 |
| CNc1ccccc1C(=O)OC | 0.593240633 |
| C1=Cc2cccc3cccc1c23 | 0.47503227 |
| C[C@@H]1O[C@@H](OCc2cc(O)c3c(c2)[C@H]([C@@H]2O[C@H](CO)[C@@H](O)[C@H](O)[C@H]2O)c2cccc(O)c2C3=O)[C@H](O)[C@H](O)[C@H]1O | 0.460859806 |
| OCc1ccc(O)cn1 | 0.674435555 |
| C/C(=C\C=C\[C@@]1(C)OC(=O)[C@]23CC=C(C(=O)O)CC[C@]2(O)[C@H]1CC3)C(=O)O | 0.627842847 |
| Oc1cc(O)c(Br)c(O)c1Br | 0.675661055 |
| CCOC(=O)/C=C/c1ccc(O)c(O)c1 | 0.66991215 |
| C[C@@H]1O[C@@H](OC[C@H]2O[C@@H](OCCc3ccc(O)c(O)c3)[C@H](O)[C@@H](OC(=O)/C=C/c3ccc(O)c(O)c3)[C@@H]2O)[C@H](O)[C@H](O)[C@H]1O | 0.497926727 |
| COc1cc2c(cc1OC)OC(C)(C)C=C2 | 0.593117032 |
| C=C(CC[C@@H](C(=O)O)[C@H]1[C@H](O)C[C@@]2(C)C3=CC[C@@H](C(=C)C)[C@](C)(CCC(=O)O)C3=CC[C@]12C)C(C)C | 0.630099855 |
| COc1ccc(/C=C/CO)cc1 | 0.562003634 |
| COC(=O)[C@H]1O[C@@H](Oc2cc3oc(-c4ccc(O)cc4)cc(=O)c3c(O)c2O)[C@H](O)[C@@H](O)[C@@H]1O | 0.515677382 |
| COc1ccc2ccc(=O)oc2c1 | 0.544826831 |
| C=C[C@@]12CN(C)[C@H]3C[C@]14C(=Nc1ccccc14)[C@H]1C[C@@H]2[C@@H]3CO1 | 0.582694112 |
| COC(=O)[C@]1(O)C[C@@H](O)[C@@H](O)[C@H](OC(=O)/C=C/c2ccc(O)c(O)c2)C1 | 0.570883624 |
| C/C=C/C=C/C=C/CC/C=C/C(=O)NCC(C)(C)O | 0.629222657 |
| CC1=CC(=O)C=C(C)C1=O | 0.565963487 |
| c1cc2c(cc1[C@H]1OC[C@H]3[C@@H]1CO[C@@H]3c1ccc3c(c1)OCO3)OCO2 | 0.469910411 |
| COc1cc2c(cc1OC)C1=Cc3ccc(OC)c(OC)c3CN1CC2 | 0.487099894 |
| CCC(=O)c1cc(OC)c(O)c(OC)c1 | 0.630373975 |
| C[C@H]1CC[C@@H]2N(C1)C[C@H]1[C@@H]3C[C@H]4[C@@H](C[C@H](O)[C@H]5C[C@@H](O)CC[C@@]54C)[C@@H]3CC[C@@H]1[C@]2(C)O | 0.788327629 |
| COc1ccc(-c2oc3c(CCC(C)(C)O)c(O)cc(O)c3c(=O)c2O)cc1 | 0.626388681 |
| CC(CC(=O)CC(C)C1CC(=O)C2(C)C3=C(C(=O)C(O)C12C)C1(C)CCC(=O)C(C)(C)C1CC3=O)C(=O)O | 0.710148775 |
| CC1=CC(=O)C(C(C)C)CC1 | 0.607965903 |
| COc1ccc(C[C@@H]2C(=O)OC[C@H]2Cc2ccc(O[C@@H]3O[C@H](CO)[C@@H](O)[C@H](O)[C@H]3O)c(OC)c2)cc1OC | 0.436120607 |
| CC1OC(OC2CCC3(C)C(CCC4(C)C3C=CC3=C5CC(C)(C)CCC5(CO)C(O)CC34C)C2(C)CO)C(O)C(OC2OC(CO)C(O)C(O)C2O)C1O | 0.801531498 |
| O=C1CCC[C@@H]2[C@H]3CCCN4CCC[C@@H](CN12)[C@H]34 | 0.642430102 |
| COc1c2c(cc3oc(C)cc(=O)c13)O[C@H](C(C)(C)O)C2 | 0.584420406 |
| CCCC(=O)c1ccccc1 | 0.602054998 |
| CCOC(C)OCCc1ccccc1 | 0.619567516 |
| CCCCCCCCC=CCCCCCCCC(=O)OCC | 0.550470338 |
| CCCCCCCc1cc(=O)c2ccccc2[nH]1 | 0.593407365 |
| O=C(O)c1cc(O)c2c(c1)C(=O)c1cccc(O[C@@H]3O[C@H](CO)[C@@H](O)[C@H](O)[C@H]3O)c1C2=O | 0.491258801 |
| CCCCCCCC1CCC(=O)O1 | 0.625961779 |
| O=C(O)CCCCCCC[C@@H](O)[C@@H](O)CCCCCCO | 0.553385045 |
| Cc1cc(O)cc(O)c1 | 0.533721164 |
| C/C1=C\[C@@H]2[C@H](C/C=C(/CO)[C@@H](O)[C@@H]3[C@@H](O)[C@@H](C)C[C@]3(O)C1=O)C2(C)C | 0.690121829 |
| COc1c2occc2c(OCC=C(C)C)c2ccc(=O)oc12 | 0.642572377 |
| COc1ccc2oc(-c3ccccc3)cc(=O)c2c1 | 0.619476986 |
| COC1=CC(=O)OC(C=Cc2ccc3c(c2)OCO3)C1 | 0.654168885 |
| CCCC[C@@H]1OC(=O)C2=C1CCC=C2 | 0.586113117 |
| CC(=O)O[C@H]1CC(C)(C)C(=C=C/C(C)=C/C=C/C(C)=C/C=C/C=C(C)/C=C/C=C(\C)C(=O)C[C@@]23O[C@]2(C)C[C@@H](O)CC3(C)C)[C@](C)(O)C1 | 0.458030316 |
| CC(=O)OC(C)(C)[C@@H]1Cc2c(ccc3ccc(=O)oc23)O1 | 0.601160082 |
| O=c1c2cc(O)ccc2oc2cc(O)c([C@@H]3O[C@H](CO)[C@@H](O)[C@H](O)[C@H]3O)c(O)c12 | 0.59169351 |
| O=C(O)[C@H](O)c1ccccc1 | 0.599499794 |
| COc1cc(-c2coc3cc(O)c(OC)c(O)c3c2=O)ccc1O | 0.61786465 |
| COc1cc(-c2cc(=O)c3c(O)cc(O[C@@H]4O[C@H](CO)[C@@H](O)[C@H](O)[C@H]4O[C@@H]4OC[C@](O)(CO)[C@H]4O)cc3o2)ccc1O | 0.419945928 |
| O=C(CCCCCCCCc1ccc(O)cc1)c1c(O)cccc1O | 0.565988163 |
| COc1cc([C@@H]2OC[C@@H]3[C@H]2CO[C@H]3c2cc(OC)c(O[C@@H]3O[C@H](CO)[C@@H](O)[C@H](O)[C@H]3O)c(OC)c2)cc(OC)c1O | 0.447984749 |
| CCCCCCCCCCCCCCOC(C)=O | 0.567945896 |
| C=C(C)C1CCC2(C)CC1c1c(O)c3c(c(CC=C(C)C)c1O2)OC12C(=CC4CC1C(C)(C)OC2(CC=C(C)C(=O)O)C4=O)C3=O | 0.577751382 |
| C=C1CC[C@H]2[C@@H](/C=C(/C)C(=O)[C@@]3(OC(C)=O)C[C@H](C)[C@H](OC(=O)Cc4ccccc4)[C@@H]3[C@H]1OC(C)=O)C2(C)C | 0.499387126 |
| O=C(O)Cc1cc(O)ccc1O[C@@H]1O[C@H](CO)[C@@H](O)[C@H](O)[C@H]1O | 0.74773335 |
| O=C(C=Cc1ccc(O)cc1)O[C@H]1[C@H](O)C[C@](O)(C(=O)O)C[C@H]1O | 0.669453256 |
| C=C1C(=O)O[C@@H]2C[C@@H](C)[C@@H]3[C@@H](OC(=O)C(C)C)C[C@@]4(C)O[C@@]34C[C@H]12 | 0.653010058 |
| Nc1cc2ccccc2oc1=O | 0.505414094 |
| CN[C@@H](Cc1c[nH]c2ccccc12)C(=O)O | 0.708696719 |
| CC1(C)CC[C@]2(C)CC[C@]3(C)C(=C2C1)CC[C@@H]1[C@@]2(C)CCC(=O)C(C)(C)[C@@H]2CC[C@]13C | 0.805382961 |
| C=C1CC[C@H]2[C@@H](/C=C(/C)C(=O)[C@@]3(OC(C)=O)C[C@H](C)[C@H](OC(=O)c4ccccc4)[C@@H]3[C@H]1OC(C)=O)C2(C)C | 0.54424521 |
| CC1(C)CCC2(C(=O)O)CCC3(C)C(=CCC4C5(C)CCC(O)C(C)(C)C5C(O)CC43C)C2C1 | 0.894885218 |
| CC(=O)CC/C=C(\C)CC/C=C(\C)CCC=C(C)C | 0.572747655 |
| C/C(=C\CNc1ncnc2nc[nH]c12)COC1O[C@H](CO)[C@@H](O)[C@H]1O | 0.645183631 |
| COc1cc(CNC(=O)CCCCCCCC(C)C)ccc1O | 0.552326032 |
| COc1ccc(C(=O)O)c(O)c1 | 0.528563048 |
| O=c1c(-c2ccc(O[C@@H]3O[C@H](CO)[C@@H](O)[C@H](O)[C@H]3O)cc2)coc2c([C@@H]3O[C@H](CO)[C@@H](O)[C@H](O)[C@H]3O)c(O)ccc12 | 0.484467038 |
| CC(CC(=O)C[C@@H](C)[C@H]1CC(=O)[C@@]2(C)C3=C(C(=O)[C@@H](O)[C@]12C)[C@@]1(C)CC[C@H](O)C(C)(C)[C@@H]1C[C@@H]3O)C(=O)O | 0.716672008 |
| COc1ccc2c(OC)c3ccoc3nc2c1OC | 0.593862167 |
| O=C1c2c(O)cc(O[C@@H]3O[C@H](CO)[C@@H](O)[C@H](O)[C@H]3O)cc2O[C@H](c2ccc(O)c(O)c2)[C@H]1O | 0.533780663 |
| CCCCCCCCCCCCCCCCCCCCCCCCCCCCCCO | 0.500453291 |
| COc1cc(O)c2c(=O)c(O[C@@H]3O[C@H](CO)[C@@H](O)[C@H](O)[C@H]3O)c(-c3ccc(O[C@@H]4O[C@H](CO)[C@@H](O)[C@H](O)[C@H]4O)cc3)oc2c1 | 0.423981859 |
| COc1cc2c(c(OC)c1OC)-c1ccc(OC)c(=O)cc1[C@@H](N(C)C(C)=O)CC2 | 0.459916539 |
| O=c1cc(-c2ccc(O)c(O)c2)oc2ccccc12 | 0.595511684 |
| O=C(NCCc1ccccc1)c1ccccc1 | 0.698636779 |
| COc1cc(CC(CO)c2cc(CCCO)cc(OC)c2O)ccc1O | 0.583195105 |
| CCCCCCCCCCCCCCCCCCCCCCCCCCCCCC(=O)O | 0.525201074 |
| COC(=O)c1ccc(O)cn1 | 0.650144043 |
| COc1cc2oc(=O)ccc2c(OC)c1C[C@@H](O)C(C)(C)OC | 0.646368465 |
| COc1cc(C)c2c(=O)c(-c3ccccc3)coc2c1 | 0.592574942 |
| COC1c2c(ccc3c2OCO3)-c2ccc3cc4c(cc3c2N1C)OCO4 | 0.513631305 |
| CC(=O)O[C@H]1C[C@H]2CC[C@]1(C)C2(C)C | 0.593097597 |
| COc1cc2c(=O)c3c(O)c([C@@H]4O[C@H](CO)[C@@H](O)[C@H](O)[C@H]4O)c(O)cc3oc2cc1O | 0.553833689 |
| O=c1c2ccccc2nc2n1CC[C@@H]2O | 0.545018448 |
| COC(=O)C1=CC[C@@]23CC[C@@H]([C@@](C)(/C=C/C=C(\C)C(=O)O[C@@H]4O[C@H](CO)[C@@H](O)[C@H](O)[C@H]4O)OC2=O)[C@@]3(OC(C)=O)CC1 | 0.476979622 |
| COc1cc2oc(=O)ccc2c(OC)c1/C=C/C(C)=O | 0.679872584 |
| C[C@@]12C[C@@]3(O)O[C@@H](O1)[C@]1(COC(=O)c4ccc(O)cc4)[C@H]3C[C@@]12O[C@@H]1O[C@H](CO)[C@@H](O)[C@H](O)[C@H]1O | 0.576353625 |
| C/C=C1/[C@H](O[C@@H]2O[C@H](CO)[C@@H](O)[C@H](O)[C@H]2O)OC=C(C(=O)OC)[C@H]1CC(=O)O | 0.590467986 |
| C[C@@H]1O[C@@H](OC[C@H]2O[C@@H](Oc3c(-c4ccc(O)cc4)oc4cc(O)cc(O)c4c3=O)[C@H](O)[C@@H](O)[C@@H]2O)[C@H](O)[C@H](O)[C@H]1O | 0.435120142 |
| CCCCCCCCCCCCCCC(=O)OC | 0.558881358 |
| C/C=C(/C)C(=O)O | 0.614580127 |
| COc1cc(CCC(=O)O)ccc1O | 0.589938931 |
| O=C(/C=C/c1ccc(O)cc1)c1c(O)cc(O)cc1O | 0.690283984 |
| CC(C)C1=C[C@H]2[C@@H](CC[C@]2(C)O)[C@](C)(O)CC1 | 0.570475569 |
| O=c1ccc2ccc(Oc3cc4cc(O)c(O[C@@H]5O[C@H](CO)[C@@H](O)[C@H](O)[C@H]5O)cc4oc3=O)cc2o1 | 0.480624522 |
| CC1=C[C@H](O)[C@@H](O)[C@]2(C)[C@H]3[C@@H](O)[C@H](O)[C@@]4(C)OC[C@@]35[C@@H](C[C@@H]12)OC(=O)[C@H](O)[C@]54O | 0.691039563 |
| O=C(O[C@@H]1[C@@H](Oc2c(-c3ccc(O)c(O)c3)oc3cc(O)cc(O)c3c2=O)O[C@@H](CO)[C@@H](O)[C@H]1O)c1cc(O)c(O)c(O)c1 | 0.418010637 |
| COc1c(O)c(=O)n2c3ccccc3c3ccnc1c32 | 0.580749952 |
| CC(=O)OC1C(=O)C2(C)C(O)CC3OCC3(OC(C)=O)C2C(OC(=O)c2ccccc2)C2(O)CC(O)C(C)=C1C2(C)C | 0.521608389 |
| O=C(O)c1cc2c(c3c1c([N+](=O)[O-])cc1ccc(O)cc13)OCO2 | 0.610022922 |
| COc1c(O)cc2c(c1O)C(=O)C[C@H](c1ccc(O)cc1)O2 | 0.604804793 |
| C=C[C@@H](O)C#CC#C[C@@H](O)/C=C\CCCCCCC | 0.64030962 |
| CC1(C)[C@H]2CC[C@]1(C)C(=O)C2 | 0.52763956 |
| CCCCCCCCCCCC(C)=O | 0.643927501 |
| COc1c2c(cc3oc(CO)cc(=O)c13)O[C@H](C(C)(C)O)C2 | 0.603082315 |
| CC1(C)[C@H](OC(=O)c2ccccc2)CC[C@]2(C)C3=CC[C@@]4(C)[C@@H]5C[C@](C)(COC(=O)c6ccccc6)CC[C@]5(C)CC[C@]4(C)C3=CC[C@@H]12 | 0.650881296 |
| COc1cc(OC)cc(OC)c1 | 0.574543496 |
| CC(=O)Oc1ccc(-c2oc3cc(OC(C)=O)cc(O)c3c(=O)c2OC(C)=O)cc1 | 0.481635717 |
| CCCCCC=CCC=CCCCCCCCCO | 0.520956583 |
| COc1cc(OC)c2ccc(=O)oc2c1 | 0.603021982 |
| O=C1c2c(O)cccc2[C@@H]([C@@H]2O[C@H](CO)[C@@H](O)[C@H](O)[C@H]2O)c2cc(CO)cc(O)c21 | 0.59572215 |
| O=c1cc(CCc2ccccc2)oc2c1[C@H](O)[C@@H](O)[C@@H](O)[C@@H]2O | 0.562032454 |
| C[N+]12CCc3cc4c(cc3C1(O)Cc1ccc3c(c1C2)OCO3)OCO4 | 0.492966361 |
| COc1cc2c(=O)c(-c3ccc(O)cc3)coc2cc1O[C@@H]1O[C@H](CO[C@@H]2OC[C@@H](O)[C@H](O)[C@H]2O)[C@@H](O)[C@H](O)[C@H]1O | 0.434906087 |
| C/C=C(\C)C(=O)O[C@H]1[C@@H]2[C@H](C)C(=O)O[C@@H]2C[C@@H](C)[C@@H]2C=CC(=O)[C@]21C | 0.593550555 |
| O=C(/C=C/c1ccc(O)c(O)c1)O[C@H]1[C@H](O[C@@H]2O[C@H](CO)[C@@H](O)[C@H](O)[C@H]2O)[C@@H](O)[C@H](OCCc2ccc(O)c(O)c2)O[C@@H]1CO | 0.489232362 |
| CC(C)c1ccc(CO)cc1 | 0.537003176 |
| OCC(O)c1ccccc1 | 0.60403311 |
| CCCCCCCCCC(C)=O | 0.643813631 |
| COC(=O)[C@@H](Cc1ccc(O)c(O)c1)OC(=O)/C=C/c1ccc(O)c2c1[C@H](C(=O)O[C@H](Cc1ccc(O)c(O)c1)C(=O)O)[C@@H](c1ccc(O)c(O)c1)O2 | 0.406894962 |
| C[C@H]1[C@@H](O)CC[C@@H]2[C@]1(C)CC[C@H]1[C@@]2(C)CC[C@@]2(C)[C@@H]3CC(C)(C)CC[C@]3(C)CC[C@]12C | 0.854790014 |
| CC(O)[C@H](C)[C@H](N)C(=O)O | 0.622765955 |
| CC1CCC2C(C)C3CCC4C(CC5C4CC(=O)C4CC(OC6OC(CO)C(O)C(O)C6O)CCC45C)C3CN2C1 | 0.730051105 |
| CC(=O)OC/C1=C/C[C@H]2[C@@H](/C=C(/C)C(=O)[C@@]3(OC(C)=O)C[C@H](C)[C@H](OC(=O)c4ccccc4)[C@@H]3[C@H]1OC(C)=O)C2(C)C | 0.524476333 |
| COc1ccc(-c2cc(=O)c3c(O)cc(O[C@@H]4O[C@H](CO)[C@@H](O)[C@H](O)[C@H]4O)cc3o2)cc1 | 0.591760267 |
| C[C@@H]1CC[C@@]2(OC1)O[C@H]1C[C@H]3[C@@H]4CCC5=CC(=O)CC[C@]5(C)[C@H]4CC(=O)[C@]3(C)[C@H]1[C@@H]2C | 0.706781726 |
| COc1cc(C[C@@H](CO)[C@](O)(CO)Cc2ccc(O)c(OC)c2)ccc1O | 0.567287604 |
| COc1cc(CCc2ccccc2O)cc(OC)c1OC | 0.562193555 |
| CC(=O)C(=O)c1ccccc1 | 0.565817698 |
| CC(C)=CCCC(C)=CCCC(C)=CCCC1(C)CCc2cc(O)c(C)c(C)c2O1 | 0.549515173 |
| CC(C)[C@@H](C)C[C@@H](O)[C@](C)(O)[C@H]1CC[C@@]2(O)C3=CC(=O)[C@@H]4C[C@@H](O)[C@@H](O)C[C@]4(C)[C@H]3CC[C@]12C | 0.846928348 |
| COC(=O)C1=CO[C@@H](O[C@@H]2O[C@H](CO)[C@@H](O)[C@H](O)[C@H]2O)[C@H]2[C@@H]1C=C[C@@]2(O)CO | 0.659278905 |
| CC1(C)CCc2c(cc3occ(-c4ccc(O)cc4)c(=O)c3c2O)O1 | 0.559804158 |
| COc1cc2c3c(c1OC)Oc1ccc(cc1)C[C@@H]1c4c(cc(OC)c(OC)c4Oc4ccc(cc4)C[C@H]3N(C)CC2)CCN1C | 0.419739744 |
| COc1cc2c(=O)c3c(O)c([C@@H]4O[C@H](CO)[C@@H](O)[C@H](O)[C@H]4O[C@@H]4OC[C@](O)(CO)[C@H]4O)c(O)cc3oc2cc1O | 0.42972453 |
| CCCCC/C=C/C=C/C=O | 0.634538539 |
| C[C@H]1[C@H]2[C@H](O[C@@H]3O[C@H](CO)[C@@H](O)[C@H](O)[C@H]3O)OC=C(C(=O)O)[C@H]2C[C@@H]1O | 0.700239237 |
| CC(=O)O[C@H]1C[C@@]23C[C@@](C)(O)[C@H](CC[C@H]2[C@](C)(O)[C@H]2[C@@H]4O[C@@H]4C(C)(C)[C@]12O)[C@@H]3O | 0.720065328 |
| CC[C@H]1CN2CC[C@@]3(C(=O)Nc4ccccc43)[C@@H]2C[C@@H]1/C(=C\OC)C(=O)OC | 0.521786019 |
| CCC1CCC(=O)O1 | 0.576742114 |
| CCCCCCCCCCCCCCCCCCCC | 0.497489728 |
| COc1cc2c(c(OC)c1OC)C(=O)c1c(cc(C)c(O)c1OC)C2=O | 0.515870199 |
| C=C1C(=O)O[C@@H]2C=C(C)[C@@H](OC(C)=O)CC=C(COC(C)=O)C[C@@H](OC(=O)/C(C)=C/CO)[C@@H]12 | 0.43986592 |
| CC1=CC(=O)C(=C(C)C)C[C@@]2(O)[C@H]1CC[C@]2(C)O | 0.607829054 |
| C/C=C(\C)C(=O)OC/C=C(\C)CCC=C(C)C | 0.748148546 |
| Cc1cncc(C)n1 | 0.629431768 |
| C[C@@H]1O[C@@H](O[C@@H]2[C@@H](O)[C@H](O)[C@@H](CO)O[C@H]2c2c(O)cc(O)c3c(=O)cc(-c4ccc(O)cc4)oc23)[C@H](O)[C@H](O)[C@H]1O | 0.435319254 |
| C=C[C@]1(C)C[C@]2(O)OC(=O)C(C)=C2C[C@H]1C(=C)C | 0.741664136 |
| C=C[C@H]1[C@H](O[C@@H]2O[C@H](CO)[C@@H](O)[C@H](O)[C@H]2O)OC=C2C(=O)OCC[C@H]21 | 0.67631952 |
| CC(=O)OC/C=C/C#CC#C/C=C/c1ccco1 | 0.767617411 |
| C[C@@]12C[C@@]3(O)O[C@@H](O1)[C@]1(COC(=O)c4ccccc4)[C@H]3C[C@@]12O[C@@H]1O[C@H](COC(=O)c2ccc(O)cc2)[C@@H](O)[C@H](O)[C@H]1O | 0.474692195 |
| COc1cc(O)c2c(=O)c(O)c(-c3ccc(O)cc3)oc2c1 | 0.63272655 |
| CS/C=C/C(=O)NCCO[C@@H]1O[C@H](CO)[C@@H](O)[C@H](O)[C@H]1O | 0.793719932 |
| COc1ccc(O)cc1 | 0.508527888 |
| CC1OC(OCC2OC(OCCc3ccc(O)c(O)c3)C(O)C(O)C2OC(=O)C=Cc2ccc(O)c(O)c2)C(O)C(O)C1O | 0.49499704 |
| C/C=C1/C[C@@H](C)[C@@](C)(O)C(=O)OCC2=CCN3CC[C@@H](OC1=O)[C@@H]23 | 0.533180378 |
| CC(C)=CCc1c(O)ccc2c1O[C@H](c1ccc(O)cc1)CC2=O | 0.568183659 |
| O=C(O)C1=CO[C@@H](O[C@@H]2O[C@H](CO)[C@@H](O)[C@H](O)[C@H]2O)[C@H]2[C@@H]1C=C[C@]2(O)CO | 0.651609473 |
| COc1cc2c(cc1O)[C@@H](Cc1ccc(O)cc1)[N+](C)(C)CC2 | 0.486380281 |
| CCCCC/C=C\C/C=C\CCCCCCCC(=O)OCC | 0.504730857 |
| COc1cc(O)c2c(=O)c(O)c(-c3ccc(OC)c(OC)c3)oc2c1 | 0.588496055 |
| Cc1ccc2oc(=O)ccc2c1 | 0.513596436 |
| O=C1C[C@@H](c2cc(O)c(O)c(O[C@@H]3O[C@H](CO)[C@@H](O)[C@H](O)[C@H]3O)c2)Oc2cc(O)cc(O)c21 | 0.54670177 |
| CC1(C)CCc2c(ccc3ccc(=O)oc23)O1 | 0.590522361 |
| CC1(C)CCCC(C)(C2CCC3(C)C2C(O)CC2C4(C)CCC(O)C(C)(C)C4C(O)CC23C)O1 | 0.87996721 |
| C=C(CC[C@@H](C(=O)O)[C@H]1CC[C@@]2(C)C3=CC[C@H]4C(C)(C)[C@@H](OC(C)=O)CC[C@]4(C)C3=CC[C@]12C)C(C)C | 0.727172228 |
| CC1(C)CC[C@@]2(C(=O)O)CCC3=C(CC[C@@H]4[C@@]5(C)C[C@H](O)[C@H](O)[C@@](C)(C(=O)O)[C@@H]5CC[C@@]34C)[C@@H]2C1 | 0.708269363 |
| C[C@]12CC[C@H](O)C[C@@]1(O)CC[C@@H]1[C@@H]2CC[C@]2(C)[C@@H](C3=CC(=O)OC3)CC[C@]12O | 0.783022843 |
| O=Cc1ccc(O[C@@H]2O[C@H](CO)[C@@H](O)[C@@H](O)[C@H]2O)cc1 | 0.71354403 |
| CC(=O)c1c[nH]c2ccccc12 | 0.55680685 |
| C[C@@H]1O[C@@H](OC[C@H]2O[C@@H](OCCc3ccc(O)c(O)c3)[C@H](O)[C@@H](O)[C@@H]2O)[C@H](O)[C@H](O)[C@H]1O | 0.523664241 |
| C=C1C(=O)OC2C1CCC(=C)C1CCC(=C)C12 | 0.655883554 |
| COc1cc2c(c(OC)c1OC)-c1c3cc4c(c1OCC(C)C(C)(O)C(=O)OC2C(C)(O)C(C)C3)OCO4 | 0.523999224 |
| COc1cc2c(cc1O)C[C@@H]1NCCc3cc(O)c(OC)c-2c31 | 0.498153023 |
| COC(=O)c1nccc2c1[nH]c1ccccc12 | 0.593777658 |
| Nc1nc(=O)c(O[C@@H]2O[C@H](CO)[C@@H](O)[C@H](O)[C@H]2O)c(N)[nH]1 | 0.667213367 |
| O=c1cc(-c2ccc(O)cc2)oc2c([C@@H]3O[C@H](CO)[C@@H](O[C@@H]4O[C@H](CO)[C@@H](O)[C@H](O)[C@H]4O)[C@H](O)[C@H]3O)c(O)cc(O)c12 | 0.42834317 |
| COc1cc2occc2c2oc(=O)ccc12 | 0.548866583 |
| CCCCCCCCCCCCCCCCCCCCCC | 0.498027173 |
| COc1cc(O)c(C(C)=O)c(OC)c1C | 0.608458532 |
| CC1(C)O[C@H]2CC(=O)OC[C@@]23[C@H]1CC(=O)[C@@]1(C)C2=CC(=O)O[C@@H](c4ccoc4)[C@]2(C)CC[C@@H]13 | 0.585319947 |
| CC/C=C\CC1=C(C)CCC1=O | 0.591295336 |
| COC[C@]12CN(C)C3C4[C@H](OC)[C@H]1C3([C@@H](OC)C[C@H]2O)[C@@H]1C[C@@]2(O)[C@H](OC(=O)c3ccccc3)[C@@H]1[C@]4(O)[C@@H](O)[C@@H]2OC | 0.649496973 |
| CC1(C)CCCC2(C)OC(=O)C=C12 | 0.529369577 |
| O=C(O)C(Cc1ccc(O)cc1)C(=O)O | 0.679345651 |
| COc1ccc2cc1Oc1ccc(cc1)C[C@@H]1c3cc(c(OC)cc3CCN1C)Oc1c(OC)c(OC)cc3c1[C@@H](C2)N(C)CC3 | 0.4102144 |
| O=C(/C=C/c1ccc(O)c(O)c1)OC[C@@H](O)CO[C@@H]1O[C@H](CO)[C@@H](O)[C@H](O)[C@H]1O | 0.664399316 |
| Cc1c(O)c(C)c2c(c1O)C(=O)[C@H](Cc1ccc3c(c1)OCO3)CO2 | 0.568855483 |
| C=C(C)C1CCC(C)(O)CC1.CC(C)=C1CCC(C)(O)CC1.CC1=CCC(C(C)(C)O)CC1 | 0.566404753 |
| Cc1cc(=O)c2c(O)c3c(cc2o1)O[C@H](C(C)(C)O[C@@H]1O[C@H](CO)[C@@H](O)[C@H](O)[C@H]1O)C3 | 0.568010773 |
| C=C1C(=O)O[C@@H]2C[C@@]3(C)CCCC(=C)[C@]3(O)C[C@H]12 | 0.599950419 |
| COC(C)(C)[C@H](O)[C@@H](O)C[C@@H](C)C1=C2C[C@H](O)[C@H]3[C@@]4(C)CCC(=O)C(C)(C)[C@@H]4CC[C@]3(C)[C@@]2(C)CC1 | 0.889869407 |
| C=C1CC[C@H]2[C@@H](/C=C(/C)C(=O)[C@@]3(OC(C)=O)C[C@H](C)[C@H](OC(=O)c4cccnc4)[C@@H]3[C@H]1OC(C)=O)C2(C)C | 0.539210876 |
| C=C1C(=O)O[C@H]2[C@H]1CC[C@]1(C)O[C@@]13CC[C@@](C)(O)[C@H]23 | 0.652543659 |
| Cc1ccc(C=O)s1 | 0.600217987 |
| COc1cc2c(cc1OC)-c1cc3ccc(OC)c(O)c3c[n+]1CC2 | 0.488547959 |
| C=C1CC[C@H]2[C@@H](/C=C(/C)C(=O)[C@@]3(O)C[C@H](C)[C@H](O)[C@@H]3[C@H]1O)C2(C)C | 0.698750868 |
| N#C/C=C1/C=C[C@@H](O)[C@H](O)[C@H]1O[C@@H]1O[C@H](CO)[C@@H](O)[C@H](O)[C@H]1O | 0.658293027 |
| C=C1[C@H](OC(=O)/C=C(\C)CC)C[C@@H](C(C)C)[C@@H]2[C@@H]([C@@H](C)OC(C)=O)C(=O)C[C@@H]12 | 0.641580877 |
| C=C(C(=O)O)c1ccccc1 | 0.638498159 |
| C=C(C)C(=O)Cc1c(OC)ccc2ccc(=O)oc12 | 0.720967905 |
| O=Cc1coc2ccccc12 | 0.477931582 |
| Cc1cccc2c3c(ccc12)C=C(C(C)C)C(=O)C3=O | 0.522907673 |
| COc1ccc([C@@H]2CC(=O)c3c(O)cc(OC)cc3O2)cc1 | 0.597499892 |
| CC1(C)[C@H](O)CC[C@@]2(C)[C@@H]3CC[C@](C)([C@@H](O)CO)C=C3CC[C@H]12 | 0.694742953 |
| COc1cc(/C=C/c2ccccc2)cc(OC)c1 | 0.621955794 |
| CC(C)=CCCC(C)=CC=O | 0.602629739 |
| C[N+](C)(C)CCc1ccc(O)cc1 | 0.59443987 |
| COc1c(O[C@@H]2O[C@H](CO)[C@@H](O)[C@H](O)[C@H]2O)cc2c(c1O)C(=O)c1c(cc(C)c(O)c1OC)C2=O | 0.417127097 |
| COc1ccc(-c2cc(=O)c3ccccc3o2)cc1 | 0.60872901 |
| COc1cc(C=CC(=O)CC(=O)C=Cc2ccc(O)cc2)ccc1O | 0.554492081 |
| NC(=O)/C=C/c1ccccc1 | 0.596268748 |
| Cc1cc(O)cc(O[C@@H]2O[C@H](CO[C@@H]3O[C@H](CO)[C@@H](O)[C@H](O)[C@H]3O)[C@@H](O)[C@H](O)[C@H]2O)c1 | 0.543572449 |
| COc1cc(O)c2c(c1)O[C@H](c1ccc(O)c(O)c1)[C@@H](O)C2=O | 0.642860169 |
| COc1ccc(/C=C/C(=O)c2ccccc2)cc1OC | 0.685768384 |
| O=C(/C=C/c1ccc(O)cc1)O[C@H](CO)CO[C@@H]1O[C@H](CO)[C@@H](O)[C@H](O)[C@H]1O | 0.672699434 |
| O=C(O)C[C@@H](O)C(=O)O | 0.592842457 |
| COc1cc(O)cc2c1cc([N+](=O)[O-])c1c(C(=O)O)cc3c(c12)OCO3 | 0.576096993 |
| CCC(=O)c1ccc(O[C@@H]2O[C@H](CO)[C@@H](O)[C@H](O)[C@H]2O)c(OC)c1 | 0.65349956 |
| CC1NC(=O)C2CCCN2C1=O | 0.461905835 |
| CCCCCC1CCCC(=O)O1 | 0.614552401 |
| C=C(C)C(CC=C(C)C)Cc1c(O)cc(O)c2c1OC(c1ccc(O)cc1OC)CC2=O | 0.546311121 |
| CC1=CC=CC(C)(C)CC1=O | 0.581307876 |
| CCCCCCCCCCCCCC(=O)OC | 0.573344422 |
| O=C(N[C@@H](Cc1ccccc1)C(=O)O)c1ccccc1 | 0.668212136 |
| CCCCCCCC/C=C\CCCCCCCCCCCC(N)=O | 0.552409094 |
| COc1ccc(O)c(C(C)=O)c1 | 0.527397098 |
| COc1cc2c[n+](C)c3c4cc5c(cc4ccc3c2cc1OC)OCO5 | 0.490264392 |
| COc1ccc(C(C)=O)c(O[C@@H]2O[C@H](CO[C@@H]3OC[C@H](O)[C@H](O)[C@H]3O)[C@@H](O)[C@H](O)[C@H]2O)c1 | 0.530489896 |
| CCCCCCCCCCCCCCCCCCCCO | 0.518705756 |
| O=C(/C=C/c1ccc(O)cc1)OC[C@@H](O)CO[C@@H]1O[C@H](CO)[C@@H](O)[C@H](O)[C@H]1O | 0.681293184 |
| COc1cccc(OC)c1C(=O)CCc1ccc(O)cc1 | 0.631336584 |
| COc1cc([C@@H]2OC[C@@H]3[C@H]2CO[C@H]3c2cc(OC)c(O)c(OC)c2)cc(OC)c1O | 0.498491158 |
| CC(=O)O[C@@]12CO[C@@H]1C[C@H](O)[C@@]1(C)C(=O)[C@H](O)C3=C(C)[C@@H](O)C[C@@](O)([C@@H](OC(=O)c4ccccc4)[C@H]21)C3(C)C | 0.495631821 |
| COC1=CC(=O)C(OC)=CC1=O | 0.549974555 |
| COc1c(O[C@@H]2O[C@H](CO)[C@@H](O)[C@H](O)[C@H]2O)cc2c(c1OC)-c1ccc(OC)c(=O)cc1[C@@H](NC(C)=O)CC2 | 0.386797574 |
| C/C=C(\C)C(=O)OC1c2c(ccc3ccc(=O)oc23)OC(C)(C)C1OC(=O)/C(C)=C/C | 0.551829398 |
| CN1CC[C@]23C(=O)C[C@H]4C(=CCO[C@H]5CC(=O)N(c6c(O)cccc62)[C@H]3[C@H]54)C1 | 0.507079834 |
| COc1cc(CNC(=O)CCCCCC(C)C)ccc1O | 0.590315442 |
| CC(=O)O[C@@H]1C[C@@H](C)[C@](O)(CCc2ccoc2)[C@@]2(C)CCCC(C)(C)[C@H]12 | 0.616301344 |
| O=C(/C=C/c1ccc(O)c(O)c1)c1ccc(O[C@@H]2O[C@H](CO)[C@@H](O)[C@H](O)[C@H]2O)cc1O | 0.59583499 |
| C[C@@]12C[C@H](c3ccoc3)O[C@@]13C[C@H](OC3=O)[C@H]1[C@H]2C[C@@H]2C[C@H]1C(=O)O2 | 0.555634963 |
| CC1(C(O)CO)C=C2CCC3C(C)(C)C(OC4OC(CO)C(O)C(O)C4O)CCC3(C)C2CC1 | 0.731841924 |
| COc1cc(C(C)=O)cc(OC)c1O | 0.547243113 |
| CC1(C)CC(=O)CC(C)(C)N1 | 0.638385394 |
| COc1ccc([C@@H]2CC(=O)c3c(O)cc(O[C@@H]4O[C@H](CO)[C@@H](O)[C@H](O)[C@H]4O)cc3O2)cc1 | 0.599509803 |
| C=C[C@H]1[C@H](O[C@@H]2O[C@H](CO)[C@@H](O)[C@H](O)[C@H]2O)OC=C2C(=O)OCC[C@]21O | 0.67070496 |
| COc1ccc2ccc(=O)oc2c1OC | 0.571679403 |
| COC(=O)[C@@H](Cc1ccc(O)c(O)c1)OC(=O)[C@H]1c2c(/C=C/C(=O)O[C@H](Cc3ccc(O)c(O)c3)C(=O)O)ccc(O)c2O[C@@H]1c1ccc(O)c(O)c1 | 0.411824277 |
| C[C@@H]1O[C@@H](Oc2cc(O)c3c(=O)c(O[C@@H]4O[C@H](CO)[C@@H](O)[C@H](O)[C@H]4O)c(-c4ccc(O)cc4)oc3c2O)[C@H](O)[C@H](O)[C@H]1O | 0.430254764 |
| Cc1nc2c(o1)c1c3c(ccc1c1occ(C)c21)C(C)(C)CCC3 | 0.521623916 |
| O=c1c2c3c(ccc2cc2n1CCc1cc4c(cc1-2)OCO4)OCO3 | 0.52731555 |
| CCCCCCCCCCCCCCCCCCCCCCCC | 0.49204224 |
| CC[C@H](CC[C@@H](C)[C@H]1CC[C@H]2[C@@H]3CC=C4C[C@@H](OC(C)=O)CC[C@]4(C)[C@H]3CC[C@]12C)C(C)C | 0.863538672 |
| CC(=O)OC(C)(C)/C=C/C(=O)[C@](C)(O)[C@H]1[C@H](O)C[C@@]2(C)[C@@H]3CC=C4[C@@H](C[C@H](O)[C@@H](O)C4(C)C)[C@]3(C)C(=O)C[C@]12C | 0.697983395 |
| OC[C@H]1O[C@@H](O[C@@H]2OCC[C@H]3[C@H](O)[C@@H]4O[C@]4(CO)[C@@H]23)[C@H](O)[C@@H](O)[C@@H]1O | 0.633220853 |
| COc1ccc([C@@H]2COc3cc(O[C@@H]4O[C@H](CO)[C@@H](O)[C@H](O)[C@H]4O)ccc3C2)c(O)c1OC | 0.529078325 |
| CCN1C[C@]2(COC)[C@H](O)C[C@H](OC)C34C1C([C@H](OC)[C@@H]32)[C@@]1(OC(C)=O)C[C@H](OC)[C@@]2(O)C[C@@H]4[C@@H]1[C@H]2OC(=O)c1ccccc1 | 0.607998009 |
| C/C=C(/C)C(=O)OC(=O)/C(C)=C\C | 0.585709638 |
| CC1(C)[C@H]2O[C@H]2[C@H]2[C@](C)(O)[C@@H]3CC[C@@H]4[C@@H](O)[C@@]3(C[C@@H](O)[C@@]21O)C[C@@]4(C)O | 0.705986268 |
| O=C(/C=C/c1ccc(O)c(O)c1)O[C@H]1[C@H](O)[C@@H](O)[C@H](OCCc2ccc(O)c(O)c2)O[C@@H]1CO[C@@H]1O[C@H](CO)[C@@H](O)[C@H](O)[C@H]1O | 0.490729758 |
| COc1cc(C)cc(OC)c1O | 0.487754512 |
| Oc1cc(O)c2c(c1)O[C@H](c1cc(O)c(O)c(O)c1)[C@@H](O)C2 | 0.565078832 |
| CC(C)=CCc1c(-c2ccc(O)cc2O)oc2c(CC=C(C)C)c(O)cc(O)c2c1=O | 0.520004562 |
| COc1ccc(/C=C/CO)cc1OC | 0.573357566 |
| COc1ccc(C=O)cc1O | 0.503162564 |
| CCCCCCCCCCCCCCCCCCCCCCC(=O)OC | 0.522841178 |
| CC(C)[C@@H]1CC[C@@H](C)C[C@@H]1O | 0.568555404 |
| CC(=CCCC1(CO)CCC2(C)C(CCC3C4(C)CCC(OC5OC(CO)C(O)C(O)C5OC5OC(CO)C(O)C(O)C5O)C(C)(COC5OC(CO)C(O)C(O)C5O)C4CCC32C)C1O)CO | 0.657055564 |
| C=CCc1ccc(O)c(O)c1 | 0.570422144 |
| O=c1c(O[C@@H]2O[C@H](CO)[C@@H](O)[C@H](O)[C@H]2O[C@@H]2O[C@H](CO)[C@@H](O)[C@H](O)[C@H]2O)c(-c2ccc(O)c(O)c2)oc2cc(O)cc(O)c12 | 0.401007881 |
| COC(=O)C1=CO[C@@H]2O[C@@H]3C[C@H]1[C@H]2[C@H](C)O3 | 0.656023426 |
| COc1ccc(C(N)=O)cc1OC | 0.57608037 |
| COc1ccc(C(C)=O)c(OC2OC(COC3OCC(O)(CO)C3O)C(O)C(O)C2O)c1 | 0.548784121 |
| O=C1C=C2C=CC(O)C(O)C2O1 | 0.545058834 |
| COc1cccc(OC)c1 | 0.539896552 |
| O=c1cc(-c2ccc(O)cc2)oc2cc(O)c([C@@H]3O[C@H](CO)[C@@H](O)[C@H](O)[C@H]3O[C@@H]3O[C@H](CO)[C@@H](O)[C@H](O)[C@H]3O)c(O)c12 | 0.436732307 |
| Oc1cc(O)c2c(c1)O[C@@H](c1cc(O)c(O)c(O)c1)[C@H](O)C2 | 0.565078832 |
| CC(C=O)Cc1ccc(C(C)(C)C)cc1 | 0.597469412 |
| Oc1ccc(Cc2ccc(O)cc2)cc1 | 0.554312081 |
| Oc1cc(O)c(Br)c(O)c1 | 0.604209706 |
| O=C(/C=C/C=C/c1ccccc1)CCc1ccccc1 | 0.675380491 |
| CC(=O)CCC=C(C)CCC=C(C)C | 0.642607541 |
| COc1cc(-c2coc3cc(O[C@@H]4O[C@H](CO)[C@@H](O)[C@H](O)[C@H]4O)c(OC)c(O)c3c2=O)cc(O)c1OC | 0.460640594 |
| CC1[C@@]23CC[C@H]4[C@@](C)(CC[C@@]5(C)[C@@H]6C[C@](C)(C(=O)O)CC[C@]6(C)CC[C@]45C)[C@@H]2C[C@@H](O)[C@]1(O)OC3 | 0.889384676 |
| CCCCCCCC/C=C\CCCCCCCC(=O)O[C@H](COC(=O)CCCCCCCCCCCCCCC)COP(=O)([O-])OCC[N+](C)(C)C | 0.430434829 |
| O=C1C[C@@H](c2ccccc2)Oc2cc(O[C@@H]3O[C@H](CO)[C@@H](O)[C@H](O)[C@H]3O)cc(O)c21 | 0.639516553 |
| CCCCCCCCCCCCCCCCCC(=O)OC | 0.525886408 |
| C=C(CC[C@@H](C(=O)O)[C@H]1[C@H](O)C[C@@]2(C)C3=CC[C@@H](C(=C)C)[C@](C)(CCC(=O)OC)C3=CC[C@]12C)C(C)C | 0.614624637 |
| C=CC(C)(CCC=C(C)C)OC(C)=O | 0.668226206 |
| O=C(O)CC(O)(CC(=O)OCc1ccc(O[C@@H]2O[C@H](CO)[C@@H](O)[C@H](O)[C@H]2O)cc1)C(=O)O | 0.538026219 |
| COc1cc(O[C@@H]2O[C@H](CO)[C@@H](O)[C@H](O)[C@H]2O)cc2cc(C)c(C(C)=O)c(O)c12 | 0.685833214 |
| O=C(/C=C/c1ccc(O)c(O)c1)O[C@@H]1C[C@](O)(C(=O)O)C[C@@H](OC(=O)/C=C/c2ccc(O)c(O)c2)[C@H]1O | 0.48068717 |
| C[C@H]1CC[C@@H]2N(C1)C[C@H]1[C@@H]3C[C@H]4[C@@H](CC(=O)[C@H]5C[C@@H](O)CC[C@@]54C)[C@@H]3CC[C@H]1[C@]2(C)O | 0.783051254 |
| C[C@@H]1O[C@@H](OC[C@H]2O[C@@H](OCCc3ccc(O)c(O)c3)[C@H](OC(=O)/C=C/c3ccc(O)c(O)c3)[C@@H](O)[C@@H]2O)[C@H](O)[C@H](O)[C@H]1O | 0.49091261 |
| OC[C@@H]1O[C@@H](OC[C@H]2O[C@@H](OC/C=C/c3ccccc3)[C@H](O)[C@@H](O)[C@@H]2O)[C@H](O)[C@H]1O | 0.568594065 |
| O=C(O)c1ccc(=O)oc1 | 0.574129319 |
| CCCCCCCCCCCCCCCCCC | 0.521166659 |
| Cc1cc(O)c2c(=O)c3c(O)cc(O)c4c5c(O)cc(O)c6c(=O)c7c(O)cc(C)cc7c(c65)c(c2c1)c34 | 0.481509376 |
| O=C(c1ccc(O)cc1)c1c(O)cc(O)c([C@@H]2O[C@H](CO)[C@@H](O)[C@H](O)[C@H]2O)c1O | 0.622854535 |
| O=c1c(O)c(-c2ccccc2)oc2cc(O)ccc12 | 0.600073385 |
| C=C1CC[C@@H]2[C@](C)(CO)[C@H](O)CC[C@@]2(C)[C@@H]1C/C=C1/C(=O)OC[C@@H]1O | 0.65658649 |
| CC(=O)c1cnccn1 | 0.685725732 |
| O=C(/C=C/c1ccc(O)cc1)NCC(O)c1ccc(O)cc1 | 0.700149174 |
| Oc1cc(O)c(Cl)c(O)c1 | 0.55123503 |
| CC(=O)C1(O)CCC2(O)C3(O)CC=C4CC(O)CCC4(C)C3CC(OC(=O)c3ccc(O)cc3)C12C | 0.575110674 |
| CC(C)CCCC(C)CCCC(C)CCCCC(C)CCCC(C)CCCC(C)C | 0.62466534 |
| COc1cc(-c2oc3cc(O)cc(O)c3c(=O)c2O[C@@H]2O[C@H](CO)[C@@H](O)[C@H](O)[C@H]2O)ccc1O | 0.515026106 |
| COc1ccc(CCC(C)=O)cc1 | 0.573647065 |
| O=C(OC[C@H]1O[C@@H](Oc2ccc(/C=C/c3cc(O)cc(O)c3)cc2)[C@H](O)[C@@H](O)[C@@H]1O)c1cc(O)c(O)c(O)c1 | 0.509355561 |
| CO[C@H]1[C@@H]2C=CO[C@@H](O[C@@H]3O[C@H](CO)[C@@H](O)[C@H](O)[C@H]3O)[C@@H]2[C@@]2(CO)O[C@@H]12 | 0.630321354 |
| CC(C)=CCCC(C)CC=O | 0.60220865 |
| O=C(O)c1ccc(O)c(O)c1O | 0.580465307 |
| C[C@H]1C(=O)C[C@@H]2C(C(=O)OCCc3ccc(O)cc3)=CO[C@@H](O[C@@H]3O[C@H](CO)[C@@H](O)[C@H](O)[C@H]3O)[C@@H]21 | 0.411978033 |
| COc1cc(-c2oc3c(OC)c(O)cc(O)c3c(=O)c2O[C@@H]2O[C@H](CO)[C@@H](O)[C@H](O)[C@H]2O[C@@H]2O[C@H](CO)[C@@H](O)[C@H](O)[C@H]2O)ccc1O | 0.40065554 |
| O=C1C[C@@H](c2ccc(O)c(O)c2)Oc2c1ccc(O[C@@H]1O[C@H](CO)[C@@H](O)[C@H](O)[C@H]1O)c2O | 0.589376598 |
| CC(=O)OCC=Cc1ccccc1 | 0.631358166 |
| CC(C)CCC[C@@H](C)[C@H]1CC[C@@]2(C)C3=C(CC[C@]12C)[C@@]1(C)CC[C@H](O)C(C)(C)[C@@H]1CC3 | 0.831642947 |
| CC(C)=CCC[C@](C)(O)[C@@H]1CC=C(C)CC1 | 0.61380313 |
| COc1ccc2cc3n(c(=O)c2c1OC)CCc1cc2c(cc1-3)OCO2 | 0.551874543 |
| CC/C=C\CCOC(=O)CCCCC | 0.625300254 |
| CC(C)=CCCC(C)(OC1OC(CO)C(O)C(O)C1O)C1CCC2(C)C1C(O)CC1C3(C)CC(O)C(OC4OC(CO)C(O)C(O)C4OC4OC(CO)C(O)C(O)C4O)C(C)(C)C3CCC12C | 0.719980944 |
| C=C(C(=O)O[C@H]1C[C@H]2CC[C@@H](C1)N2C)c1ccccc1 | 0.584681823 |
| COc1cc(O)cc(OC)c1CCC(=O)c1ccc(O)cc1 | 0.609582691 |
| CCCCCCCCCCCCCCCCCCCCCCCCO | 0.511074618 |
| OC[C@H]1O[C@@H](O[C@@H]2[C@@H](O)[C@H](O[C@@H]3[C@@H](O)[C@@H](O)O[C@H](CO)[C@H]3O)O[C@H](CO)[C@H]2O)[C@H](O)[C@@H](O)[C@@H]1O | 0.531647832 |
| CC1(C)[C@H](OC(=O)c2ccccc2)CC[C@]2(C)C3=C([C@@H](O)C[C@@H]12)[C@@]1(C)CC[C@@]2(C)CC[C@@](C)(COC(=O)c4ccccc4)C[C@H]2[C@]1(C)CC3 | 0.672889155 |
| CC=Cc1ccc(OC)cc1 | 0.557174165 |
| C=C(C[C@@H](O)[C@](C)(O)[C@H]1CC[C@@]2(O)C3=CC(=O)[C@@H]4C[C@@H](O)[C@@H](O)C[C@]4(C)[C@H]3CC[C@]12C)C(C)C | 0.831039246 |
| CC/C=C\C/C=C\C/C=C\CCCCCCCC(=O)OC | 0.4831948 |
| Cc1ccc(C=O)o1 | 0.570164777 |
| C[C@@H]1[C@@H](C)[C@H](c2ccc3c(c2)OCO3)O[C@@H]1c1ccc2c(c1)OCO2 | 0.456117674 |
| C[C@@H]1[C@H]2[C@H](O[C@@H]3O[C@H](CO)[C@@H](O)[C@H](O)[C@H]3O)OC=C(C(=O)O)[C@H]2C[C@@H]1O | 0.700239237 |
| COc1cc(C)c2c(c1C=O)Oc1c(c(C)c(O)c3c1C(O)OC3=O)OC2=O | 0.546124031 |
| C/C1=C\C[C@]2(C(C)C)CC[C@@](C)(O2)[C@@H](O)CC/C(C)=C/CC1 | 0.596841852 |
| CC(C)c1cc2c(c(O)c1O)C13CCCC(C)(C)C1C(OC3=O)C2=O | 0.551537837 |
| O=C(/C=C/c1ccc(O)c(O)c1)O[C@@H](C(=O)O)[C@@H](O)C(=O)O | 0.65076112 |
| C=C[C@H](O)C/C(=N/OS(=O)(=O)O)S[C@@H]1O[C@H](CO)[C@@H](O)[C@H](O)[C@H]1O | 0.711676072 |
| CCCCCCCCCCCCCCCCCCCCCCCC(=O)OC | 0.523831551 |
| COc1cc2c(c3ccccc13)OC(C)(C)CC2 | 0.494799863 |
| CC1=CC(=O)[C@@H](O)[C@]2(C)[C@H]3[C@@]4(O)OC[C@@]35[C@@H](C[C@@H]12)OC(=O)[C@H](O)[C@@]5(O)[C@@]1(CO1)[C@H]4O | 0.582206501 |
| COc1cc([C@@H]2c3cc4c(cc3[C@H](O)[C@H]3COC(=O)[C@H]23)OCO4)cc(OC)c1O | 0.578994797 |
| CC(=O)O[C@H]1C(C)=C[C@]23C(=O)[C@H](C=C(C)[C@@H](OC(=O)c4ccccc4)[C@]12O)[C@H]1[C@@H](C[C@H]3C)C1(C)C | 0.596316285 |
| O=c1cc(-c2ccc(O)c(O)c2)oc2cc(O[C@@H]3O[C@H](CO)[C@@H](O)[C@H](O)[C@H]3O)c(O)c(O)c12 | 0.522118656 |
| CC1(C)C[C@H]2C3=CC[C@@H]4[C@@]5(C)CC[C@H](O)[C@](C)(CO)[C@@H]5CC[C@@]4(C)[C@]3(C)C[C@@H](O)[C@@]2(CO)[C@@H](O)[C@@H]1O | 0.862985063 |
| C=C1[C@H]2C[C@@]3(C4C[C@H]5C6(C4N(CC)C[C@]5(C)CC[C@@H]6O)[C@@H]3C[C@H]2O)[C@@H]1O | 0.796598819 |
| COc1c2c(cc3c1OCO3)CCN(C)C2 | 0.521919026 |
| CC[C@H](CC[C@@H](C)[C@H]1CC[C@H]2[C@@H]3CCC4=CC(=O)CC[C@]4(C)[C@H]3CC[C@]12C)C(C)C | 0.822268698 |
| CC1=CC(=O)CC(C)(C)C1(O)C=C/C(C)=C\C(=O)O | 0.628178032 |
| COc1c(C(C)C)cc(O)c2c1CC[C@H]1C3=C(CC[C@]21C)C(=O)OC3 | 0.554132279 |
| C[N+](C)(C)CCO.[OH-] | 0.570247332 |
| COc1c(O[C@@H]2O[C@H](CO[C@@H]3OC[C@@H](O)[C@H](O)[C@H]3O)[C@@H](O)[C@H](O)[C@H]2O)cc2occ(-c3ccc(O)cc3)c(=O)c2c1O | 0.423668652 |
| COc1cc2c(cc1OC)-c1cc3ccc4c(c3c(=O)n1CC2)OCO4 | 0.550792812 |
| C=C1CC23CCC4C(C)(C(=O)O)CCCC4(C)C2CCC1(OC1OC(CO)C(O)C(O)C1OC1OC(CO)C(O)C(O)C1O)C3 | 0.720046954 |
| COc1cc(O)cc(OC)c1OC | 0.573806069 |
| CC(CC(=O)C[C@@H](C)[C@H]1CC(=O)[C@@]2(C)C3=C(C(=O)[C@@H](O)[C@]12C)[C@@]1(C)CC[C@H](O)C(C)(C)[C@@H]1CC3=O)C(=O)O | 0.691081331 |
| COc1cc2oc(-c3ccc(O)cc3)cc(=O)c2c(O)c1[C@@H]1O[C@H](CO)[C@@H](O)[C@H](O)[C@H]1O[C@@H]1O[C@H](CO)[C@@H](O)[C@H](O)[C@H]1O | 0.42590384 |
| CC(C)(O)[C@@H](O)COc1c2ccoc2cc2oc(=O)ccc12 | 0.67968079 |
| CCCCOC(=O)[C@]1(O)C[C@@H](O)[C@@H](O)[C@H](OC(=O)/C=C/c2ccc(O)c(O)c2)C1 | 0.567727864 |
| CC(=O)NCc1ccccc1 | 0.67921491 |
| CCC(C)C1NC(=O)C(C)NC1=O | 0.578485831 |
| COc1cc(C(C)=O)ccc1O[C@@H]1O[C@H](CO)[C@@H](O)[C@H](O)[C@H]1O | 0.678421853 |
| C[C@]12C[C@@H](O)[C@@H]3C[C@@]1(O[C@@H]1O[C@H](COC(=O)c4ccccc4)[C@@H](O)[C@H](O)[C@H]1O)[C@]3(COC(=O)c1ccccc1)C(=O)O2 | 0.495883283 |
| COc1cc([C@@H]2c3cc4c(cc3C(=O)[C@H]3COC(=O)[C@@H]23)OCO4)cc(OC)c1OC | 0.48209052 |
| CC(=O)c1c(O)c(C)c(O)c2c1OC1=CC(=O)/C(=C(/C)N)C(=O)[C@@]12C | 0.531417389 |
| Cc1cc(=O)oc2c([N+](=O)[O-])c(O)ccc12 | 0.645883279 |
| CCCCCCCCCCCCCCCCCCCO | 0.524409726 |
| COc1ccc(O)c(C(C)(C)C)c1 | 0.516884142 |
| CCCCCCCCCCCCCCCCCCC(=O)O | 0.546536264 |
| CC(=CCNc1ncnc2nc[nH]c12)CO | 0.712693623 |
| CC1(C)CCC2(C)CCC3(C)C(=CCC4C5(C)CCC(O)C(C)(C(=O)O)C5CCC43C)C2C1 | 0.874397697 |
| COc1cc2c(c(OC)c1OC)-c1c(cc3c(c1OC)OCO3)C[C@H](C)[C@H](C)[C@H]2O | 0.540930948 |
| COc1cc2c(c(OC)c1)C(=O)OC2 | 0.547879217 |
| C=C(C)[C@H]1CC=C(C)C(=O)C1 | 0.609530532 |
| C[N+](C)(C)C(Cc1c[nH]c2ccccc12)C(=O)[O-] | 0.638755718 |
| O=C1NC(=O)C(Br)=C1Br | 0.664188048 |
| O=C(/C=C/c1ccccc1)c1ccccc1O | 0.675916057 |
| CC1=C(C=O)C(C)(C)CCC1 | 0.580680674 |
| O=c1cc(CCc2ccccc2)oc2c1[C@H](O)[C@@H](O)[C@H](O)[C@H]2O | 0.562032454 |
| COc1cccc(O)c1C(=O)O | 0.541177396 |
| CC(=O)OCC=C(C)CCC=C(C)CCC=C(C)C | 0.621650164 |
| CC(C)[C@@H]1CC[C@@H](C)CC1=O | 0.607719218 |
| C=CCc1ccc(OC)cc1 | 0.574367326 |
| COc1c2ccoc2c(O)c2oc(=O)ccc12 | 0.588345214 |
| C[C@H]1[C@H](C)CC[C@]2(C)CC[C@]3(C)C(=CC(=O)[C@@H]4[C@@]5(C)CC[C@H](O)C(C)(C)[C@@H]5CC[C@]43C)[C@H]12 | 0.887371508 |
| COc1ccc2c(c1OC)O[C@H]1c3ccc(O[C@@H]4O[C@H](CO)[C@@H](O)[C@H](O)[C@H]4O)cc3OC[C@@H]21 | 0.529473476 |
| COc1cc(/C=C/C(=O)OC[C@H]2O[C@@H](O[C@@H]3OC=C[C@H]4[C@H](O)[C@@H]5O[C@]5(CO)[C@@H]34)[C@H](O)[C@@H](O)[C@@H]2O)ccc1O | 0.501738724 |
| COc1cc(O)c2c(=O)cc(C)oc2c1 | 0.534110706 |
| CC(=O)O[C@H]1Cc2c(cc3oc(C)cc(=O)c3c2O)OC1(C)C | 0.523554673 |
| CCCCCCc1cccs1 | 0.610478138 |
| COc1cc(O)c(C(=O)/C=C/c2ccccc2)c(O)c1 | 0.675552562 |
| O=C(/C=C/c1ccccc1)O[C@H]1C[C@](O)(C(=O)O)C[C@H](O)[C@H]1O | 0.655284062 |
| O=C1C(=Cc2ccc(O)c(O)c2)Oc2cc(O)cc(O)c21 | 0.668490116 |
| O=C(CCc1ccccc1)C[C@H](O)CCc1ccccc1 | 0.578539576 |
| Cc1cc(C)c(O)c(C)c1 | 0.520686382 |
| O=C1C[C@@H](c2ccc(O)cc2)Oc2cc(O[C@@H]3O[C@H](CO)[C@@H](O)[C@H](O)[C@H]3O)ccc21 | 0.650496004 |
| CCCCCCCCCCCCCCCC(=O)OCC | 0.56762036 |
| CCN1C[C@]2(C)CC[C@H](OC)C34C1C1(OCO[C@@]15C[C@H](OC)[C@H]1C[C@]3(O)C5C1OC)[C@@H](OC(C)=O)[C@@H]42 | 0.716769473 |
| COc1ccc(-c2oc3c(CC=C(C)C)c(O[C@@H]4O[C@H](CO)[C@@H](O)[C@H](O)[C@H]4O)cc(O)c3c(=O)c2O)cc1 | 0.461747556 |
| COc1c2ccoc2c(OCC(O)C(C)(C)O)c2oc(=O)ccc12 | 0.633195464 |
| Cc1ccc2c(c1)C(=O)c1ccccc1C2=O | 0.608666189 |
| c1cc2c(cc1[C@H]1OC[C@H]3[C@@H]1CO[C@H]3c1ccc3c(c1)OCO3)OCO2 | 0.469910411 |
| CC(=O)O[C@@H]1C(=O)C2=C(C(=O)C[C@H]3C(C)(C)C(=O)CC[C@]23C)[C@]2(C)C(=O)C[C@H]([C@H](C)CC(=O)CC(C)C(=O)O)[C@@]12C | 0.664904962 |
| CCCCCC1C(=O)CCC1CC(=O)OC | 0.625882859 |
| CCCCCCCCCCCCCCCO | 0.550367279 |
| N#C[C@H](O[C@@H]1O[C@H](CO[C@@H]2O[C@H](CO)[C@@H](O)[C@H](O)[C@H]2O)[C@@H](O)[C@H](O)[C@H]1O)c1ccccc1 | 0.535865677 |
| O=c1cc(-c2ccc(O)cc2)oc2cc(O[C@@H]3O[C@H](CO)[C@@H](O)[C@H](O)[C@H]3O[C@@H]3OC[C@](O)(CO)[C@H]3O)cc(O)c12 | 0.447651169 |
| CCCCCCCCCCCO | 0.619086523 |
| CC12CCC(C1)C(C)(C)C2O | 0.512666603 |
| COc1cc2oc(=O)ccc2c(OC)c1C[C@H](O)C(C)(C)O | 0.689218838 |
| Cc1c(O)cc2c(c1O)C(=O)c1ccc(O)c(O)c1C2=O | 0.564506467 |
| O=c1oc2c(O)cc(O)cc2c2oc3cc(O)c(O)cc3c12 | 0.582818001 |
| COc1ccc2c3c([nH]c2c1)C(C)=NCC3 | 0.479507164 |
| COC(=O)C1=COC(O[C@@H]2O[C@H](CO)[C@@H](O)[C@H](O)[C@H]2O)/C(=C/C(=O)O)C1CC(=O)OCCc1ccc(O)c(O)c1 | 0.406539687 |
| C/C=C1/CN2[C@H]3Cc4c([nH]c5ccccc45)[C@]2(O)C[C@@H]1[C@@]3(CO)C(=O)OC | 0.662063491 |
| CC1=C(O)C(=O)CO1 | 0.575292316 |
| COC(=O)Cc1ccc(O)cc1O | 0.583360474 |
| C=C1C(=O)O[C@@H]2CC(C)=C([C@@H](C)CCCO)[C@@H](O)[C@H]12 | 0.650081302 |
| CCOc1cc(C=O)ccc1O | 0.529186651 |
| CCCCC(C)C(=O)O | 0.662348991 |
| CC(=O)OCc1ccco1 | 0.605871296 |
| CC1=CC(=O)CC(C)(C)C1=O | 0.513620945 |
| O=C(O)[C@H]1CC[C@H](O)CC1 | 0.574551443 |
| C[C@@H]1O[C@@H](Oc2c(O)cc3c(=O)oc4c(O)c(O)cc5c(=O)oc2c3c45)[C@H](O)[C@H](O)[C@H]1O | 0.532131484 |
| O=Cc1ccc(O[C@@H]2O[C@H](CO)[C@@H](O)[C@H](O)[C@H]2O)cc1 | 0.71354403 |
| COc1cc(-c2coc3cc4c(c(O)c3c2=O)OCO4)cc(O)c1OC | 0.546956965 |
| COc1cc(CCCO)cc(OC)c1O | 0.606721461 |
| CC(C)=CCNc1ncnc2[nH]cnc12 | 0.643892777 |
| C=C1CCC2(C(C)C)CC12 | 0.546532083 |
| O=C1c2ccccc2C(=O)c2cc(O)ccc21 | 0.592297076 |
| C/C=C/C#CC#CC(O)C(/C=C/CCCO)O[C@@H]1O[C@H](CO)[C@@H](O)[C@H](O)[C@H]1O | 0.630588772 |
| CCCCCCCCCCCCCC | 0.572589307 |
| O=c1ccc2cc(O)c(O[C@@H]3O[C@H](CO)[C@@H](O)[C@H](O)[C@H]3O)cc2o1 | 0.702649747 |
| C=C(C)[C@@H](O)Cc1c(OC)cc2oc(=O)ccc2c1OC | 0.687372602 |
| COc1ccc(C(=O)O)c(O)c1O | 0.535916237 |
| CC(=O)O[C@@]1(C)CC[C@]2(O)C=CO[C@@H](O[C@@H]3O[C@H](CO)[C@@H](O)[C@H](O)[C@H]3O)[C@@H]21 | 0.694182093 |
| CCN1C[C@]2(COC)CC[C@H](O)C34C1C(O)([C@@H](OC)[C@@H]32)[C@@]1(O)C[C@H](OC)[C@H]2C[C@@H]4[C@@H]1[C@H]2OC | 0.709987826 |
| CC(=O)c1ccc(O[C@@H]2O[C@H](CO)[C@@H](O)[C@H](O)[C@H]2O)c(O)c1O | 0.719423106 |
| O=C1CCC[C@H]2[C@@H]3CCCN4CCC[C@@H](CN12)[C@H]34 | 0.642430102 |
| OCC1=C[C@@H](O)[C@]2(O)C=CO[C@@H](O[C@@H]3O[C@H](CO)[C@@H](O)[C@H](O)[C@H]3O)[C@H]12 | 0.610783242 |
| Cc1cc(O)c2c(c1)C(=O)c1cc(O[C@@H]3O[C@H](CO)[C@@H](O)[C@H](O)[C@H]3O)cc(O)c1C2=O | 0.482860192 |
| C[C@@H]1CCC=C2C(=O)C[C@H]3OOC(C)(C)[C@H]3[C@]21C | 0.640394812 |
| COc1cc(OC)cc(C(C)=O)c1 | 0.604198237 |
| CC(C)=CCC[C@]1(C)CC[C@]2(C)[C@H]3CC[C@@]4(C)[C@@H](CCC(=O)[C@@H]4C)[C@]3(C)CC[C@@]2(C)C1 | 0.801775683 |
| CCCCCCCCCCCCCCCCC(=O)OC | 0.553070062 |
| COC1=C[C@@H]2[C@@H]3Cc4ccc(OC)c(O)c4[C@]2(CC[N+]3(C)[O-])CC1=O | 0.620967135 |
| CCCCCCCC/C=C\CCCCCCCCCC(=O)O | 0.549909728 |
| COc1ccc(C(C)=O)c(O[C@@H]2O[C@H](CO)[C@@H](O)[C@H](O)[C@H]2O)c1 | 0.687421341 |
| COc1cc(-c2oc3cc(O)cc(OC)c3c(=O)c2O)ccc1O | 0.631867557 |
| CCCCCCC(O)CC=CCCCCCCCC(=O)O | 0.530666465 |
| O=C(/C=C/c1ccc(O)c2c1[C@H](C(=O)O[C@H](Cc1ccc(O)c(O)c1)C(=O)O)[C@@H](c1ccc(O)c(O)c1)O2)O[C@H](Cc1ccc(O)c(O)c1)C(=O)O | 0.408985433 |
| CC1(C)C=Cc2c(ccc(C(=O)/C=C/c3ccc(O)cc3)c2O)O1 | 0.582912484 |
| CCC/C=C1\OC(=O)C2=C1CC[C@@H](O)[C@H]2O | 0.67219281 |
| COc1ccc(C2CC(=O)c3ccccc3O2)cc1 | 0.599997435 |
| COc1cc(C)cc(OC)c1 | 0.544625016 |
| C[C@@H]1O[C@@H](O[C@@H]2C(=O)c3c(O)cc(O)cc3O[C@@H]2c2ccc(O)cc2)[C@H](O)[C@H](O)[C@H]1O | 0.598583926 |
| C[C@@H]1CCCC2=CC(=O)[C@@H]3[C@@H](C3(C)C)[C@]21C | 0.678081892 |
| Cc1cc(O[C@@H]2O[C@H](CO)[C@@H](O)[C@H](O)[C@H]2O)c2c(c1)C(=O)c1cc(O)cc(O)c1C2=O | 0.481809648 |
| CN1C2CCC1CC(=O)C2 | 0.587215236 |
| CC(C)=CC[C@@H](OC(=O)C(C)C)C1=CC(=O)c2c(O)ccc(O)c2C1=O | 0.526317466 |
| O[C@H]1[C@H]2c3cc4c(cc3CN3CC[C@H](C[C@@H]1O)[C@H]23)OCO4 | 0.518116542 |
| C1CNCCNCCNCCN1 | 0.599577715 |
| O=c1c(O)c(-c2ccc(O)cc2O)oc2cc(O)cc(O)c12 | 0.623963979 |
| CC(=O)Nc1cccc(O)c1 | 0.605760346 |
| C[C@@H]1CC[C@H]2[C@@H](C)C(=O)O[C@@H]3O[C@@]4(C)CC[C@@H]1[C@]32O4 | 0.658131116 |
| C[C@]12CC[C@H](O)C[C@H]1CC[C@@H]1[C@@H]2C(=O)[C@H](O)[C@]2(C)[C@@H](c3ccc(=O)oc3)CC[C@]12O | 0.66482482 |
| CCOC(=O)CC(C)O | 0.654356501 |
| COc1ccc2c(OC)c3ccoc3nc2c1 | 0.556065491 |
| CC[C@@]12C=CCN3CC[C@@]4(C(=C(C(=O)OC)C1)Nc1ccccc14)[C@H]32 | 0.638867834 |
| O=C(/C=C/c1ccc(O)cc1)O[C@@H]1C[C@](O)(C(=O)O)C[C@@H](O)[C@H]1O | 0.666651711 |
| COc1c(O)cc2c(c1O)C(=O)c1c(cc(C)c(O)c1OC)C2=O | 0.552908294 |
| COc1cc(C(=O)NCCO)ccc1O | 0.691971988 |
| O=c1c(O[C@@H]2O[C@H](CO)[C@@H](O)[C@H](O)[C@H]2O[C@@H]2OC[C@@H](O)[C@H](O)[C@H]2O)c(-c2ccc(O)cc2)oc2cc(O)cc(O)c12 | 0.433696482 |
| Cc1cc(=O)oc2cc(O)ccc12 | 0.513155707 |
| COC(=O)[C@H]1O[C@@H](Oc2cc3oc(-c4ccc(OC)cc4)cc(=O)c3c(O)c2OC)[C@H](O)[C@@H](O)[C@@H]1O | 0.49476189 |
| C=C(C)[C@@H]1CC=C(C)C(=O)C1 | 0.609530532 |
| O=C(/C=C/c1ccc(O)cc1)c1ccc(O[C@@H]2O[C@H](CO)[C@@H](O)[C@H](O)[C@H]2O)cc1O | 0.671145927 |
| COc1cc2oc(=O)ccc2c(OC)c1CC=C(C)C | 0.705551221 |
| CC(=O)OC[C@@]12CC[C@@H](C)[C@](C)(CCC3=CC(=O)OC3)[C@H]1C[C@H](O)C=C2CO | 0.572765354 |
| O=C(C=Cc1ccccc1)OCC=Cc1ccccc1 | 0.72666712 |
| O=C(O)[C@H]1O[C@@H](Oc2cc(-c3cc(=O)c4c(O)cc(O)cc4o3)ccc2O)[C@H](O)[C@@H](O)[C@@H]1O | 0.505997759 |
| CC1(C)CC[C@]2(C(=O)O)[C@H](O)C[C@]3(C)C(=CC[C@@H]4[C@@]5(C)CC[C@H](O)[C@@](C)(C=O)[C@@H]5CC[C@]43C)[C@@H]2C1 | 0.784044917 |
| O=c1cc(-c2ccccc2)oc2cc(O[C@@H]3O[C@H](CO[C@@H]4O[C@H](CO)[C@@H](O)[C@H](O)[C@H]4O)[C@@H](O)[C@H](O)[C@H]3O)cc(O)c12 | 0.468733045 |
| CCCCCCCCCCCC(=O)O.CCCCCCCCCCCC(=O)O.CCCCCCCCCCCC(=O)OCC(O)CO | 0.446372116 |
| C[N+](C)(C)CCc1ccc(O)c(O)c1 | 0.581362292 |
| COc1ccc([C@H]2Oc3cc(O)cc(O)c3C(=O)[C@H]2[C@@H]2C(=O)c3c(O)cc(O)cc3O[C@@H]2c2ccc(OC)cc2)cc1 | 0.403658347 |
| COc1ccc(C(=O)O)c(O)c1OC | 0.576191786 |
| O=c1c2cc(O)ccc2oc2cc(O)c([C@@H]3O[C@H](CO)[C@@H](O)[C@H](O)[C@H]3O[C@@H]3OC[C@](O)(CO)[C@H]3O)c(O)c12 | 0.454615838 |
| CC(C)[C@@H](C)CC[C@@H](C)[C@H]1CCC2=C3CC[C@H]4C[C@@H](O)CC[C@]4(C)[C@H]3CC[C@@]21C | 0.793699117 |
| O=C(O)C[C@H](C(=O)O)[C@H]1c2c(cc(O)c(O)c2O)C(=O)O[C@@H]1C(=O)O | 0.655282995 |
| C[C@]12CC[C@H](O)C[C@@]1(O)CC[C@@H]1[C@@H]2CC[C@]2(C)[C@@H](c3ccc(=O)oc3)CC[C@]12O | 0.673813803 |
| COC(=O)c1cccnc1 | 0.645415765 |
| O=C(/C=C/c1ccc(O)c(O)c1)O[C@@H]1[C@@H](O)[C@H](OCCc2ccc(O)c(O)c2)O[C@H](CO[C@@H]2O[C@H](CO)[C@@H](O)[C@H](O)[C@H]2O)[C@H]1O | 0.489753195 |
| CO[C@H]1[C@H](O)[C@@H](O)[C@@H](O)[C@@H](O)[C@@H]1O | 0.574039782 |
| COc1cc(C=CC(=O)NCCCCN)ccc1O | 0.771365259 |
| COc1c(O[C@@H]2O[C@H](C(=O)O)[C@@H](O)[C@H](O)[C@H]2O)cc2oc(-c3ccccc3)cc(=O)c2c1O | 0.552327899 |
| CC1(C)CC[C@]2(C(=O)O)[C@H](O)C[C@]3(C)C(=CC[C@@H]4[C@@]5(C)C[C@H](O)[C@H](O)C(CO)(CO)[C@@H]5CC[C@]43C)[C@@H]2C1 | 0.792363298 |
| C[C@]12CC[C@H](O)C[C@H]1CC[C@@H]1[C@@H]2CC[C@]2(C)[C@@H](c3ccc(=O)oc3)[C@@H](O)[C@H]3O[C@]132 | 0.735258074 |
| O=C(O)c1cc(O)c2c(c1)C(C1c3cc(CO)cc(O)c3C(=O)c3c(OC4OC(CO)C(O)C(O)C4O)cccc31)c1cccc(OC3OC(CO)C(O)C(O)C3O)c1C2=O | 0.446720854 |
| CC(=O)O[C@@H](C[C@@H](C)C1=C2C[C@H](O)[C@H]3[C@@]4(C)CCC(=O)C(C)(C)[C@@H]4CC[C@]3(C)[C@@]2(C)CC1)[C@H]1OC1(C)C | 0.79719434 |
| CCCCCCCCCCCCCCCC(=O)OCC(O)COC(=O)CCCCCCCCCCCCCCC | 0.379517783 |
| O=C1C(=C2Nc3ccccc3C2=O)Nc2ccccc21 | 0.562737539 |
| COc1cc2c(cc1O)CCN1Cc3c(ccc(OC)c3OC)C[C@H]21 | 0.489974102 |
| COc1cc(-c2cc(=O)c3c(O)c(OC)c(O)cc3o2)cc(O)c1OC | 0.59454752 |
| CCCCC1OC(=O)c2ccccc21 | 0.530492865 |
| CC(=O)OCC(COC1OC(CO)C(O)C(O)C1O)OC(=O)C=Cc1ccc(O)cc1 | 0.515531991 |
| CCCCCc1ccco1 | 0.567504355 |
| COc1cc2ccc(=O)oc2cc1OC | 0.574035108 |
| O=c1c(O[C@@H]2O[C@H](CO)[C@@H](O)[C@H](O)[C@H]2O)c(-c2ccc(O)cc2)oc2c(O[C@@H]3O[C@H](CO)[C@@H](O)[C@H](O)[C@H]3O)c(O)cc(O)c12 | 0.428613209 |
| OC[C@@H](O)[C@@H](O)[C@@H](O)[C@@H](O)CO | 0.575057934 |
| C=C(C)[C@@H]1CC=C2C(=CC[C@]3(C)[C@@H]([C@@H](CCC=C(C)C)C(=O)O)[C@H](O)C[C@@]23C)[C@@]1(C)CCC(=O)O | 0.611682246 |
| CC(=O)O[C@]12CCC(C(=O)O)=CC[C@]13CC[C@H]2[C@@](C)(/C=C/C=C(\C)C(=O)O)OC3=O | 0.58629445 |
| COc1ccc(O[C@@H]2O[C@H](CO)[C@@H](O)[C@H](O)[C@H]2O)cc1 | 0.690798847 |
| C=Cc1ccc(O)c(OC)c1 | 0.529549037 |
| NCc1c[nH]c2ccccc12 | 0.553433905 |
| COCCc1ccc(O)cc1 | 0.562067337 |
| O=C(O)c1ccc(O)c(O)c1 | 0.559366917 |
| CC(=O)O[C@@H]1C[C@H]2CC[C@]1(C)C2(C)C | 0.593097597 |
| CCCCCCCCCCCCCC(C)=O | 0.57586421 |
| COc1ccc(-c2cc(=O)c3c(O)c(OC)c(O)c(OC)c3o2)cc1 | 0.589016188 |
| C[C@]12CC[C@H]3[C@@H](CC[C@@H]4C[C@@H](O)CC[C@@]43CO)[C@@]1(O)CC[C@@H]2c1ccc(=O)oc1 | 0.689120464 |
| O=c1cc(-c2ccccc2)oc2c([C@@H]3O[C@H](CO)[C@@H](O)[C@H](O)[C@H]3O)c(O)c([C@@H]3OC[C@H](O)[C@H](O)[C@H]3O)c(O)c12 | 0.44568239 |
| COc1cc(O)ccc1CCC(=O)c1ccc(O)cc1 | 0.6535562 |
| CC1=CC(=O)[C@H]2C[C@@H]1C2(C)C | 0.536448995 |
| CCN1C[C@]2(COC)CC[C@H](OC)C34C1C([C@H](OC)[C@@H]32)[C@@]1(OC(C)=O)C[C@H](OC)[C@@]2(O)C[C@@H]4[C@@H]1[C@H]2OC(=O)c1ccc(OC)cc1 | 0.619711977 |
| COc1ccc(CO)cc1OC | 0.571759298 |
| COc1cc(/C=C/C(=O)NCCc2ccc(O)cc2)cc(OC)c1O | 0.646432783 |
| C=CC1=C(C)c2cc3[nH]c(c4c5nc(cc6[nH]c(cc1n2)c(C)c6CC)C(C)=C5C(=O)C4)C(CCC(=O)O)[C@@H]3C | 0.53969016 |
| O=CN1C[C@@H]2C[C@H](C1)c1cccc(=O)n1C2 | 0.532429735 |
| CCCCCC/C=C\CCCCCCCCC(=O)O | 0.581508993 |
| CC(C)=CC[C@H](OC(=O)C=C(C)C)C1=CC(=O)c2c(O)ccc(O)c2C1=O | 0.528514486 |
| CNCc1ccccc1 | 0.633612883 |
| O=C(CCCCCCCCc1ccc(O)c(O)c1)c1c(O)cccc1O | 0.561990349 |
| CC(=O)O[C@]12CCC(C)=CC[C@]13CC[C@H]2[C@@](C)(/C=C/C=C(\C)C(=O)O[C@@H]1O[C@H](CO)[C@@H](O)[C@H](O)[C@H]1O)OC3=O | 0.490807916 |
| OC[C@H]1O[C@@H](Oc2c(O)cc(O)cc2/C=C/c2ccc(O)cc2)[C@H](O)[C@@H](O)[C@@H]1O | 0.635084749 |
| Cc1cc(=O)c2c(O)c3c(O[C@@H]4O[C@H](CO[C@@H]5O[C@H](CO)[C@@H](O)[C@H](O)[C@H]5O)[C@@H](O)[C@H](O)[C@H]4O)cc(O)cc3cc2o1 | 0.441815236 |
| CC(C)=CCc1c2ccoc2c(O)c2oc(=O)ccc12 | 0.616680678 |
| C[C@@H](O)CCc1ccc(O)cc1 | 0.539894704 |
| Cc1c(O)c(C)c2occ(Cc3ccc4c(c3)OCO4)c(=O)c2c1O | 0.556145052 |
| O=C1CC(c2ccc(O)cc2)Oc2cc(O)c([C@@H]3O[C@H](CO)[C@@H](O)[C@H](O)[C@H]3O)c(O)c21 | 0.591526424 |
| CC(=O)O[C@@H](C[C@@H](C)C1=C2C[C@H](O)[C@H]3[C@@]4(C)CCC(=O)C(C)(C)[C@@H]4CC[C@]3(C)[C@@]2(C)CC1=O)[C@H]1OC1(C)C | 0.77854518 |
| CC(C)=CCc1c2c(c(O)c3c1O[C@]14C(=C[C@@H]5C[C@H]1C(C)(C)O[C@@]4(C/C=C(/C)C(=O)O)C5=O)C3=O)C=CC(C)(C)O2 | 0.538693269 |
| COc1ccc2oc(=O)cc(C)c2c1 | 0.579309287 |
| O=CCCc1ccccc1 | 0.585328147 |
| CC(C)CCCC(C)CCCC(C)CCCC(C)C | 0.603444134 |
| COc1cc(C=CC(=O)O[C@@H]2C[C@](O)(C(=O)O)C[C@@H](O)[C@H]2OC(=O)C=Cc2ccc(O)c(O)c2)ccc1O | 0.477081823 |
| N[C@@H](Cn1ccc(=O)c(O)c1)C(=O)O | 0.655780257 |
| O=c1ccn([C@@H]2O[C@H](CO)[C@@H](O)[C@@H]2O)c(=O)[nH]1 | 0.624929512 |
| Oc1c(Br)c(O)c(Br)c(O)c1Br | 0.664529337 |
| CC1=CC(=O)CC(C)(C)C1 | 0.577867107 |
| CC(=O)Oc1ccc(-c2coc3cc4c(c(O)c3c2=O)C=CC(C)(C)O4)cc1 | 0.572436532 |
| CCCCCCCCCCCCCCCCCCC(=O)OC | 0.525488666 |
| COC1=C[C@@]23CCN(C)[C@@H](Cc4ccc(OC)c(O)c42)C3=CC1=O | 0.54828653 |
| COc1cc(O)cc(O)c1CCC(=O)c1ccc(O)cc1 | 0.648532701 |
| O=c1cc(-c2ccc(O)cc2)oc2ccccc12 | 0.592026783 |
| CC(=O)O[C@@H]1C[C@H]2C(=O)C=C3[C@H](CC[C@]4(C)[C@@H]([C@@](C)(O)[C@H](O)CCC(C)(C)O)CC[C@@]34O)[C@@]2(C)C[C@@H]1O | 0.68948281 |
| CCCCCCCCCCCCCCCCCCCCCCC | 0.497966719 |
| C[C@@]12O[C@H]3[C@@H](O[C@H](CO)[C@@H](O)[C@@H]3O)O[C@@]13C[C@H]1C(=O)C[C@@H](O2)[C@]13COC(=O)c1ccccc1 | 0.545413234 |
| COC1=C(C)C(=O)O[C@]12C(=O)C(C)=C1CCCCN3[C@H]([C@@H]4C[C@H](C)C(=O)O4)CC[C@@]132 | 0.564242659 |
| COc1cc(C=O)ccc1OC(C)=O | 0.541269764 |
| CC(=O)O[C@H]1CC[C@@]2(C)[C@H](CC[C@@H]3[C@@H]2CC[C@]2(C)[C@@H]4[C@H](C[C@@H]32)O[C@]2(CC[C@@H](C)CO2)[C@H]4C)C1 | 0.877160057 |
| O=Cc1c(O)cc(O)cc1O | 0.531882208 |
| O=c1c(-c2ccc(O[C@@H]3O[C@H](CO)[C@@H](O)[C@H](O)[C@H]3O)cc2)coc2cc(O)cc(O)c12 | 0.57234147 |
| COc1cc2c(cc1O)-c1cc3ccc(OC)c(OC)c3c[n+]1CC2 | 0.483290128 |
| COC(=O)C1=CO[C@@H](O[C@@H]2O[C@H](CO)[C@@H](O)[C@H](O)[C@H]2O)[C@@H]2[C@@]3(C)O[C@H]3[C@@H](O)[C@]12O | 0.647598541 |
| CC1CCC2(OC1)OC1CC3C4CC=C5CC(O)CCC5(C)C4CCC3(C)C1C2C | 0.838599664 |
| NCc1ccccc1O | 0.557506754 |
| COc1c(CC=C(C)C)c(O)cc2oc(=O)c3c4ccc(O)cc4oc3c12 | 0.624165746 |
| O=C1c2ccccc2C(=O)c2c1cc(O)c(O)c2O | 0.54413383 |
| CC(C)CC1NC(=O)C2CCCN2C1=O | 0.514664556 |
| O=C1c2c(O)cc(O)cc2O[C@@H](c2ccc(O)cc2)[C@H]1[C@@H]1C(=O)c2c(O)cc(O)cc2O[C@@H]1c1ccc(O)cc1 | 0.397944393 |
| C/C=C(/C)C(=O)O[C@H]1c2cc(OC)c(OC)c(OC)c2-c2c(cc(OC)c(OC)c2OC)C[C@H](C)[C@]1(C)O | 0.498498835 |
| CC1=C2C[C@H]3CC[C@@H](C)[C@]2(CC1=O)C3(C)C | 0.609072205 |
| COc1cc2c(cc1OC)C(=O)NCC2 | 0.577697626 |
| Cn1c(=O)c2c(c3ccccc31)OC(C)(C)C=C2 | 0.555565373 |
| CC(=O)OC/C1=C\[C@H]2[C@@H](OC(=O)/C=C/c3ccccc3)[C@@H](C)C[C@]2(OC(C)=O)C(=O)/C(C)=C/[C@@H]2[C@H](CC1)C2(C)C | 0.442956567 |
| CCCCCCCCCC(C)O | 0.616637669 |
| CC(N)CC(=O)O | 0.617523492 |
| O=C1c2ccc(O)cc2OC[C@@H]1c1ccc(O)cc1 | 0.615557735 |
| COc1ccc(-c2cc(=O)c3c(O)c(OC)c(OC)cc3o2)cc1OC | 0.55278186 |
| COc1cc(/C=C/C(=O)OCc2ccc(O[C@@H]3O[C@H](CO)[C@@H](O)[C@H](O)[C@H]3O)cc2)ccc1O[C@@H]1O[C@H](CO)[C@@H](O)[C@H](O)[C@H]1O | 0.486518465 |
| O=c1c(-c2ccc3c(c2)OCO3)coc2cc(O)ccc12 | 0.572836946 |
| CCOC(=O)Cc1ccc(O)cc1 | 0.588431424 |
| CCCCCCCCCCCCCC(=O)OC(C)C | 0.577470465 |
| CN1C2CCC1CC(O)C2 | 0.567088238 |
| Cl.NCCc1ccccc1 | 0.602668733 |
| C=C1C=CC(C(C)C)CC1 | 0.566786321 |
| Oc1cc(O)c2ccccc2c1 | 0.487302501 |
| C=CC1(C)CCC(C(C)(C)O)O1 | 0.554433005 |
| CC(C)[C@@H](C)/C=C/[C@@H](C)[C@H]1CC[C@H]2C3=C[C@@H]4O[C@@]45C[C@@H](O)CC[C@]5(C)[C@H]3CC[C@]12C | 0.822887618 |
| COc1cc(CO)cc(OC)c1O | 0.530929768 |
| COc1cc(O)cc(CCc2ccccc2O)c1 | 0.571700933 |
| O=C(O[C@@H]1Cc2c(O)cc(O)cc2O[C@H]1c1cc(O)c(O)c(O)c1)c1cc(O)c(O)c(O)c1 | 0.536369054 |
| C=CC(C)(CCC=C(C)CCC=C(C)C)OC(C)=O | 0.692296202 |
| O=C1CCCCCCCCCCCCCC1 | 0.606084342 |
| COC[C@@]12CC[C@H](OC)C34C(C([C@H](OC)[C@@H]31)[C@]1(O)[C@@H]3[C@H]4C[C@@](O)([C@@H]3O)[C@@H](OC)[C@@H]1O)N(C)C2 | 0.694169553 |
| CC(C)[C@H]1CC[C@@H](C)CC1=O | 0.607719218 |
| CCC(=O)c1ccccc1O | 0.556929351 |
| C=C1C(=O)[C@@]23C(=O)O[C@H]4CCC(C)(C)[C@H]5[C@H](O)OC[C@@]45[C@@H]2CC[C@@H]1C3O | 0.63754446 |
| COc1ccc(C(=O)OCCc2ccc(O)cc2)cc1 | 0.704611464 |
| CC(=O)OC(C)(C)CC(=O)O[C@@H](CC=C(C)C)C1=CC(=O)c2c(O)ccc(O)c2C1=O | 0.465460748 |
| CC(C)(O)[C@H]1Cc2cc3ccc(=O)oc3cc2O1 | 0.628218488 |
| C[C@@H]1CC[C@@]2(OC1)O[C@H]1C[C@H]3[C@@H]4C[C@H](O)[C@H]5C[C@@H](O)CC[C@]5(C)[C@H]4CC[C@]3(C)[C@H]1[C@@H]2C | 0.809890085 |
| CCCCCC(C)C(=O)O | 0.632881524 |
| COc1ccn(C)c(=O)c1C#N | 0.606299106 |
| O=c1c(O)c(-c2ccc(O)cc2)oc2cc(O[C@@H]3O[C@H](CO)[C@@H](O)[C@H](O)[C@H]3O)cc(O)c12 | 0.554566862 |
| CC1OC(OC2C(O)C(OCCc3ccc(O)c(O)c3)OC(CO)C2OC(=O)C=Cc2ccc(O)c(O)c2)C(O)C(OC2OCC(O)(CO)C2O)C1O | 0.431131986 |
| O=c1c(O)c(-c2c(O)cccc2O)oc2cc(O)cc(O)c12 | 0.634424349 |
| CC(C)=CCCC(C)(O)C1CCC2(C)C1C(O)CC1C3(C)CCC(OC4OC(CO)C(O)C(O)C4OC4OC(CO)C(O)C(O)C4O)C(C)(C)C3CCC12C | 0.812009579 |
| COc1ccc2c(C)c3[n+](cc2c1OC)CCc1cc2c(cc1-3)OCO2.[Cl-] | 0.493279314 |
| CCCCCCCCCCCCCCCCCCCCCC(=O)OC | 0.528375032 |
| O=C(O)CC(=O)OC[C@H]1O[C@@H](Oc2cc(O)c3c(=O)cc(-c4ccc(O)cc4)oc3c2)[C@H](O)[C@@H](O)[C@@H]1O | 0.454320227 |
| Cc1ccc2[nH]c3ccccc3c2c1 | 0.523273772 |
| CC12CCC(O)CC1CCC1C2CCC2(C)C(c3ccc(=O)oc3)CC3OC312 | 0.743121524 |
| O=C1C[C@@H](c2ccc(O[C@@H]3O[C@H](CO)[C@@H](O)[C@H](O)[C@H]3O[C@@H]3OC[C@](O)(CO)[C@H]3O)cc2)Oc2cc(O)ccc21 | 0.472177757 |
| O=C(O)C1CCN1 | 0.630020401 |
| C=C(C)[C@@H]1CC[C@]2(C)CC[C@]3(C)[C@H](CC[C@@H]4[C@@]5(C)CC[C@H](OC(=O)CCCCCCCCCCCCCCC)C(C)(C)[C@@H]5CC[C@]43C)[C@@H]12 | 0.821544248 |
| CN1[C@@H]2CC[C@H]1C[C@@H](O)C2 | 0.567088238 |
| COc1ccc(-c2cc(=O)c3c(O)cc(O[C@@H]4O[C@H](CO)[C@H](O)[C@H](O)[C@H]4O)cc3o2)cc1 | 0.591760267 |
| C/C=C/c1ccc(OC)c(OC)c1 | 0.554650803 |
| COC(=O)/C=C/c1ccc(OC)c(O)c1 | 0.669542719 |
| CN1CCC[C@H]1C(=O)O | 0.602549812 |
| COc1cc(/C=C/c2cc(O)cc(O)c2)ccc1O[C@@H]1O[C@H](CO)[C@@H](O)[C@H](O)[C@H]1O | 0.641411984 |
| CCCCCCCCCCC(=O)OC | 0.646093268 |
| CC1CC[C@@]2(OC1)O[C@H]1C[C@H]3[C@@H]4CCC5CC(O)CC[C@]5(C)[C@H]4CC[C@]3(C)[C@H]1[C@@H]2C | 0.823430393 |
| C=C(C)[C@@H]1CC[C@@H](C)C[C@H]1O | 0.55304376 |
| C=CC(=C)CCC=C(C)C | 0.609310471 |
| COc1c2c(cc3oc(C)cc(=O)c13)O[C@H](C(C)(C)O[C@@H]1O[C@H](CO[C@@H]3OC[C@](O)(CO)[C@H]3O)[C@@H](O)[C@H](O)[C@H]1O)C2 | 0.44520893 |
| COc1cc(/C=C/c2cc(O)cc(O[C@@H]3O[C@H](CO)[C@@H](O)[C@H](O)[C@H]3O)c2)ccc1O[C@@H]1O[C@H](CO)[C@@H](O)[C@H](O)[C@H]1O | 0.477270711 |
| C/C=C/C(=O)C1=C(C)CCCC1(C)C | 0.604933744 |
| C[C@H]1CCCC(=O)CCC/C=C/c2cc(O)cc(O)c2C(=O)O1 | 0.536152505 |
| O=C(O)C/C=C(\CC(=O)O)C(=O)O | 0.604677392 |
| COc1cc([C@@H]2c3cc4c(cc3[C@H](O)[C@@H](CO)[C@H]2CO)OCO4)cc(OC)c1OC | 0.506982624 |
| COc1ccc(C2COc3cc(O[C@@H]4O[C@H](CO)[C@@H](O)[C@H](O)[C@H]4O)ccc3C2)c(O)c1OC | 0.529078325 |
| C[C@@H]1O[C@@H](O[C@@H]2C(=O)c3c(O)cc(O)cc3O[C@H]2c2ccc(O)c(O)c2)[C@H](O)[C@H](O)[C@H]1O | 0.538609821 |
| CC1(C)CCC2(C(=O)O)CCC3(C)C(=CCC4C5(C)CCC(O)C(C)(CO)C5CCC43C)C2C1 | 0.878153447 |
| COc1cc(CCC(=O)CCCCc2ccccc2)ccc1O | 0.548579945 |
| O=c1cc(-c2ccc(O)c(O)c2)oc2c([C@@H]3O[C@H](CO)[C@@H](O)[C@H](O)[C@H]3O)c(O)cc(O)c12 | 0.504602146 |
| O=C1CCCCCCCCCCCCCCO1 | 0.631340862 |
| COc1ccc(-c2coc3cc(OC)cc(O)c3c2=O)cc1 | 0.621868329 |
| O=c1cc(-c2ccccc2)c2ccc(O)c(O)c2o1 | 0.629529645 |
| CC(=O)OC/C=C(/C)CCC=C(C)C | 0.668390453 |
| CC1=CC[C@@H](C(C)(C)O)CC1 | 0.544406607 |
| CC(C)(O)[C@@H]1Cc2c(cc3oc(CO)cc(=O)c3c2O)O1 | 0.577150568 |
| O=C(O)C(O)C(O)C(=O)O | 0.587087206 |
| CCCCCCCCCCCCCCC | 0.533783842 |
| C=C(O)C(=O)CC | 0.660064269 |
| CCOc1ccc2ccc(=O)oc2c1 | 0.590851146 |
| CC1(C)Oc2cc3oc(=O)ccc3cc2C[C@@H]1O | 0.62678335 |
| CC(=O)O[C@H]([C@@H](O)C[C@@H](C)C1=C2C[C@H](O)[C@H]3[C@@]4(C)CCC(=O)C(C)(C)[C@@H]4CC[C@]3(C)[C@@]2(C)CC1)C(C)(C)O | 0.76197514 |
| CC(C)(O)[C@@H]1CC[C@](C)([C@H]2[C@@H](O)C[C@@]3(C)[C@@H]4C[C@H](O)[C@H]5C(C)(C)[C@@H](O)CC[C@]5(C)C4=CC[C@]23C)O1 | 0.872689911 |
| COc1cc(C[C@H]2COC(=O)[C@@]2(Cc2ccc(O)c(OC)c2)O[C@@H]2O[C@H](CO)[C@@H](O)[C@H](O)[C@H]2O)ccc1O | 0.482781558 |
| CCCCCCCC(=O)NCc1ccc(O)c(OC)c1 | 0.638075083 |
| COc1cc2c(cc1OC)-c1c3c(cc4c1[C@@H](C2)N(C)CC4)OCO3 | 0.506907447 |
| OC[C@H]1O[C@@](CO)(O[C@H]2O[C@H](CO[C@@H]3O[C@H](CO)[C@@H](O)[C@H](O)[C@H]3O)[C@@H](O)[C@H](O)[C@H]2O)[C@@H](O)[C@@H]1O | 0.524047775 |
| COc1ccc(-c2oc3c(OC)c(OC)c(OC)c(OC)c3c(=O)c2OC)cc1OC | 0.475081796 |
| CCCCCC/C=C\CCCCCCCC(=O)OC | 0.558732358 |
| O=C1c2c(O)cc(O[C@@H]3O[C@H](CO)[C@@H](O)[C@H](O)[C@H]3O)cc2O[C@H](c2ccc(O)cc2)[C@H]1O | 0.565097092 |
| CC(=O)CCc1ccc(O[C@@H]2O[C@H](CO)[C@@H](O)[C@H](O)[C@H]2O)cc1 | 0.670554553 |
| Cc1cc(O)c2c(c1)C(=O)c1cc(O)cc(O[C@@H]3O[C@H](CO)[C@@H](O)[C@H](O)[C@H]3O)c1C2=O | 0.481809648 |
| CCCCCCCCCCCCC | 0.573601196 |
| CC1(C)OC1COc1c2occc2cc2ccc(=O)oc12 | 0.644787287 |
| C[C@@H]1O[C@@H](O[C@@H]2[C@@H](O)[C@H](OCCc3ccc(O)c(O)c3)O[C@H](CO)[C@H]2O)[C@H](O)[C@H](O)[C@H]1O | 0.516846905 |
| COc1c2c(cc3oc(C)cc(=O)c13)O[C@H](C(C)(C)O[C@@H]1O[C@H](CO)[C@@H](O)[C@H](O)[C@H]1O)C2 | 0.564521056 |
| c1ccc(CNc2ncnc3nc[nH]c23)cc1 | 0.683435766 |
| C/C(=C/C(=O)CC(C)C(=O)O)C1CC(O)C2(C)C3=C(C(=O)CC12C)C1(C)CCC(=O)C(C)(C)C1CC3O | 0.717253329 |
| O=C(CCO)c1ccc(O)cc1 | 0.59594085 |
| COc1ccc(C(=O)O)cc1O | 0.555774367 |
| O=c1cc(-c2ccc(O)c(O)c2)oc2c([C@@H]3O[C@H](CO)[C@@H](O)[C@H](O)[C@H]3O[C@H]3O[C@@H](CO)[C@@H](O)[C@@H](O)[C@@H]3O)c(O)cc(O)c12 | 0.383128706 |
| CCCCCCCCCCCC(=O)OC | 0.665317865 |
| O=c1ccc2ccc(O[C@@H]3O[C@H](CO)[C@@H](O)[C@H](O)[C@H]3O)cc2o1 | 0.721012375 |
| CCCCCCCCCCCCCCCC | 0.525530182 |
| COc1cccc(OC)c1O | 0.527702459 |
| CC(C)=CCOc1cc2oc(=O)ccc2cc1O | 0.693899313 |
| CCOC(=O)C1C(C=C(C)C)C1(C)C | 0.664753615 |
| COC(=O)C1=CO[C@@H](O[C@@H]2O[C@H](CO)[C@@H](O)[C@H](O)[C@H]2O)[C@@H]2[C@@H](C)CC(=O)[C@]12O | 0.565121261 |
| COC(=O)C1=CO[C@@H](O[C@@H]2O[C@H](CO)[C@@H](O)[C@H](O)[C@H]2O)[C@@H]2C(CO)=C[C@H](O)[C@H]12 | 0.658372097 |
| COC[C@@]12CC[C@H](OC)C34C(C([C@H](OC)[C@@H]31)[C@]1(O)[C@@H]3[C@H]4C[C@@](O)([C@@H]3OC(=O)c3ccccc3)[C@@H](OC)[C@@H]1O)N(C)C2 | 0.640856243 |
| CC(=O)c1ccc(C)cc1 | 0.563788926 |
| O=C(CCCCCCCCc1ccccc1)c1c(O)cccc1O | 0.567396975 |
| CC(C)=CC(=O)OC(C)(C)C1Cc2cc3ccc(=O)oc3cc2O1 | 0.586437428 |
| OC[C@H]1O[C@@H](Oc2c(O)cc(O)cc2C=Cc2ccc(O)cc2)[C@H](O)[C@@H](O)[C@@H]1O | 0.635084749 |
| CNCCc1cc(OC)c(OC)c2c1ccc1ccccc12.Cl | 0.518703511 |
| COc1cc2c(cc1O)CC[N+](C)(C)[C@@H]2Cc1ccc(O)cc1 | 0.484377076 |
| CC1=C(/C=C/C(C)=C/C=C/C(C)=C/CO)C(C)(C)CCC1 | 0.570991434 |
| COc1c2ccoc2cc2oc(C)cc(=O)c12 | 0.59937223 |
| CO[C@@H]1C[C@H](O[C@H]2CC[C@@]3(C)[C@H](CC[C@@]45CC[C@H](C6=CC(=O)OC6)[C@@](C)(CC[C@@H]43)C5=O)C2)O[C@H](C)[C@@H]1O | 0.712293902 |
| COc1cc2oc(=O)cc(-c3ccccc3)c2cc1O | 0.624608541 |
| COC(=O)C1=CO[C@@H](O[C@H]2O[C@@H](CO)[C@H](O)[C@@H](O)[C@@H]2O)[C@H]2[C@@H]1C(=O)C[C@@H]2C | 0.607316913 |
| C[C@@H]1O[C@@H](Oc2c(-c3ccc(O)cc3)oc3cc(O)cc(O)c3c2=O)[C@H](O)[C@H](O[C@@H]2O[C@H](CO)[C@@H](O)[C@H](O)[C@H]2O)[C@H]1O | 0.444025462 |
| COc1ccc(C2COc3cc(O)ccc3C2)c(O)c1OC | 0.555494582 |
| COc1ccccc1OCC(O)CO | 0.56035629 |
| C[C@]12CC[C@H](O)C[C@H]1CC[C@@H]1[C@@H]2[C@H](O)C(=O)[C@]2(C)[C@@H](c3ccc(=O)oc3)CC[C@]12O | 0.662553229 |
| COc1ccc([C@@H]2CC(=O)c3c(O)cc(O)cc3O2)cc1 | 0.617654696 |
| CCCCCCCCCCCCCCCCCCCCCCCCCCCCO | 0.512791152 |
| COc1ccc(-c2coc3cc(O)ccc3c2=O)cc1 | 0.598388894 |
| CCC12CCCN3CCc4c(n(c5ccccc45)C(O)(C(=O)OC)C1)C32 | 0.610765664 |
| C=Cc1cc(O)cc2ccc3c(C)c(O)ccc3c12 | 0.523605698 |
| O=C1CC(c2ccccc2)Oc2cc(O)cc(O)c21 | 0.603076666 |
| O=C1c2c(O)cc(O)cc2O[C@H](c2ccc(O)cc2)[C@H]1O | 0.652994192 |
| CC1=CC(=O)C(C(C)C)=CC1=O | 0.520448367 |
| CSC(=O)OCC1=C[C@H](O)[C@@H]2C(C(=O)O)=CO[C@@H](O[C@@H]3O[C@H](CO)[C@@H](O)[C@H](O)[C@H]3O)[C@H]12 | 0.588097742 |
| CCCCCCCCCCCCCCCCCCCCCCCCCCCC(=O)O | 0.541681928 |
| CC(=O)c1ccc(C)cc1O | 0.536163711 |
| O=c1ccocc1O | 0.594112503 |
| C=C1C(=O)O[C@H]2C[C@@H](C)[C@@H]3CC[C@H](O)[C@@]3(C)C[C@H]12 | 0.653509316 |
| O=c1c2c(O)cc(O)cc2oc2c(O)ccc(O)c12 | 0.584086305 |
| O=c1c(O[C@@H]2O[C@H](CO)[C@@H](O)[C@H](O)[C@H]2O[C@@H]2O[C@H](CO)[C@@H](O)[C@H](O)[C@H]2O)c(-c2ccc(O)cc2)oc2cc(O)cc(O)c12 | 0.429843076 |
| Cc1cc(O)ccc1O | 0.535479594 |
| O=C1CC(c2ccccc2)Oc2cc(O)ccc21 | 0.584147282 |
| O=C1c2ccccc2C(=O)c2cc(CO)ccc21 | 0.608403555 |
| COc1cc(O)c2c(=O)c(OC)c(-c3ccc(O)cc3)oc2c1 | 0.621946571 |
| CCN1C[C@]2(COC)CC[C@H](O)C34C1C([C@H](OC)[C@@H]32)[C@]1(O)[C@H]2[C@@H](O)[C@H](C[C@H]24)[C@@H](OC)[C@@H]1O | 0.704541519 |
| C=C1CC[C@@H]2[C@H]([C@@H]3[C@H](C)CC[C@@H]13)C2(C)C | 0.633754445 |
| COc1cc(O)c2c(c1)O[C@H](c1ccc(OC)c(O)c1)CC2=O | 0.629287232 |
| C=C[C@H]1[C@H](O[C@@H]2O[C@H](CO)[C@@H](O)[C@H](O)[C@H]2O)OC=C(C(=O)OC)[C@H]1CC(=O)O | 0.581748678 |
| Oc1cc(O)cc(CCc2ccccc2)c1 | 0.599784634 |
| COC(=O)[C@H]1O[C@@H](Oc2cc3oc(-c4ccccc4)cc(=O)c3c(O)c2O)[C@H](O)[C@@H](O)[C@@H]1O | 0.510993581 |
| O[C@]12CO[C@H](c3ccc4c(c3)OCO4)[C@H]1CO[C@@H]2c1ccc2c(c1)OCO2 | 0.534369177 |
| CCCCCc1cc(O)c2c(c1)OC(C)(CCC=C(C)C)C=C2 | 0.567659013 |
| CO[C@H]1c2cc(C(C)C)c(O)c(O)c2[C@@]23CCCC(C)(C)[C@@H]2[C@@H]1OC3=O | 0.645409312 |
| COc1ccc2cc3[n+](cc2c1O)CCc1cc2c(cc1-3)OCO2.[Cl-] | 0.495228268 |
| COc1c(O[C@@H]2O[C@H](C(=O)O)[C@@H](O)[C@H](O)[C@H]2O)cc2oc(-c3ccc(O)cc3)cc(=O)c2c1O | 0.507533898 |
| CC(=O)O[C@@H]1C[C@@H]2CC[C@@]1(C)C2(C)C | 0.593097597 |
| CC(C)=CCC/C(C)=C/CC/C(C)=C/COc1ccc2ccc(=O)oc2c1 | 0.574067178 |
| COC(=O)c1ccccc1NC(=O)c1c[nH]c2ccccc12 | 0.592282743 |
| CC(=O)O[C@H]1C[C@@]2(C)[C@@H](C[C@H]1O)C(=O)C=C1[C@@H]2CC[C@]2(C)[C@@H]([C@@](C)(O)[C@H](O)CCC(C)(C)O)CC[C@@]12O | 0.68948281 |
| COc1c(/C=C/C(=O)c2ccc(O)cc2)ccc(O)c1O | 0.666152947 |
| Oc1cc2c(cc1O)[C@@H]1c3ccc(O)c(O)c3OC[C@]1(O)C2 | 0.593943126 |
| C/C=C(/C)C(=O)O[C@H]1c2cc(OC)c(OC)c(OC)c2[C@@H]2C(=C[C@H](C)[C@]1(C)O)C=C1OCOC1=C2OC | 0.492853545 |
| CC1(C)CC[C@]2(C)CC[C@]3(C)C(=CC[C@@H]4[C@@]5(C)CC[C@H](O)C(C)(C)[C@@H]5CC[C@]43C)[C@@H]2C1 | 0.831783947 |
| Nc1nc(O)nc2nc[nH]c12 | 0.595089328 |
| Cc1cc(O)c2c(c1)C(=O)c1cccc(O)c1C2=O | 0.541467782 |
| COc1c2occc2c(OC)c2c(=O)cc(C)oc12 | 0.605665342 |
| C[C@@H]1C(=O)OC[C@H](O)[C@H]1C[C@@H](O)[C@](C)(O)[C@H]1CCC2(O)C3=CC(=O)[C@@H]4C[C@@H](O)[C@@H](O)C[C@]4(C)[C@H]3CC[C@]12C | 0.651426403 |
| COC(=O)/C=C/c1cc(O)ccc1O | 0.617603205 |
| COc1cc(O)ccc1/C=C/C(=O)c1ccc(O)cc1 | 0.703632081 |
| OC[C@H]1O[C@@H](n2cnc3c(NCc4ccc(O)cc4)ncnc32)[C@H](O)[C@@H]1O | 0.66808285 |
| CC=CCCCCCCC(CCCCCCC)OC(=O)c1ccccc1O | 0.468871721 |
| COc1cc(-c2ccccc2)cc(OC)c1O | 0.601732701 |
| CC(C)=CCc1cc2c(=O)cc(-c3ccc(O)cc3)oc2cc1O | 0.53826002 |
| O=C1c2cc(CO)cc(O)c2C(=O)c2c(O[C@@H]3O[C@H](CO)[C@@H](O)[C@H](O)[C@H]3O)cccc21 | 0.493511373 |
| CC1OC(OC2C(OCCc3ccc(O)c(O)c3)OC(CO)C(O)C2OC(=O)C=Cc2ccc(O)c(O)c2)C(O)C(O)C1O | 0.488438652 |
| COc1cc([C@@H]2[C@H]3CO[C@@]4(O)C(=O)[C@@H]2C=C([C@H]2Oc5cc(O)cc(O)c5C(=O)[C@@H]2O)[C@@H]34)ccc1O | 0.457113952 |
| O=c1c2cc(O[C@@H]3O[C@H](CO)[C@@H](O)[C@H](O)[C@H]3O)c(O)cc2oc2cc(O)c([C@@H]3O[C@H](CO)[C@@H](O)[C@H](O)[C@H]3O)c(O)c12 | 0.428191329 |
| C=C[C@H]1CN2CC[C@]3(C(=O)Nc4ccccc43)[C@@H]2C[C@@H]1/C(=C\OC)C(=O)OC | 0.528837322 |
| O=c1c(O[C@@H]2O[C@@H](CO)[C@H](O)[C@H]2O)c(-c2ccc(O)c(O)c2)oc2cc(O)cc(O)c12 | 0.494162275 |
| COc1ccc([C@H]2OC[C@H]3[C@@H]2CO[C@@H]3c2cc(OC)c(OC)c(OC)c2)cc1OC | 0.490526383 |
| COc1cc(/C=C/C(=O)O[C@H]2[C@H](O)[C@@H](CO)O[C@@]2(CO)O[C@@H]2O[C@H](COC(=O)c3ccc(O)cc3)[C@@H](O)[C@H](O)[C@H]2O)cc(OC)c1OC | 0.384349391 |
| Oc1ccc(CCc2cc(O)cc(O)c2)cc1 | 0.577278015 |
| CC(C)[C@@H]1NC(=O)CNC(=O)[C@H](C)NC(=O)[C@H](Cc2c[nH]c3ccccc23)NC(=O)[C@H](C)NC1=O | 0.450880089 |
| O=c1c(-c2ccc(O)cc2)coc2cc(O[C@@H]3O[C@H](CO)[C@@H](O)[C@H](O)[C@H]3O)cc(O)c12 | 0.577943855 |
| O=c1oc2cc(O)ccc2c2oc3cc(O)ccc3c12 | 0.607291548 |
| CC(=O)OCC12C(OC(C)=O)C(OC(C)=O)C3C(OC(C)=O)C14OC3(C)COC(=O)c1cccnc1CCC(C)C(=O)OC(C(OC(=O)c1ccccc1)C2OC(C)=O)C4(C)O | 0.500857141 |
| CC(=O)OCC1=CO[C@@H](OC(=O)CC(C)C)[C@H]2C1=C[C@H](OC(=O)CC(C)(C)OC(C)=O)[C@]21CO1 | 0.442402412 |
| C[C@]12CC[C@H](O)C[C@H]1CC[C@@H]1[C@@H]2CC[C@]2(C)[C@@H](c3ccc(=O)oc3)CC[C@]12O | 0.701122053 |
| CC(=O)c1ccccc1O | 0.554037535 |
| O=C(O)[C@H]1O[C@@H](Oc2cc(O)c3c(=O)cc(-c4ccc(O)c(O)c4)oc3c2)[C@H](O)[C@@H](O)[C@@H]1O | 0.511958217 |
| COc1ccc(-c2coc3cc(OC)c(OC)cc3c2=O)cc1 | 0.586785644 |
| O=C(O)[C@H]1O[C@@H](Oc2cc(O)c3c(=O)cc(-c4ccc(O)cc4)oc3c2)[C@H](O)[C@@H](O)[C@@H]1O | 0.548311568 |
| O=C(/C=C/c1ccc(O)c(O)c1/C=C/c1ccc(O)c(O)c1)O[C@H](Cc1ccc(O)c(O)c1)C(=O)O | 0.472411048 |
| COc1ccc(-c2cc(=O)c3c(O)cc(O)c(-c4cc(-c5cc(=O)c6c(O)cc(O)cc6o5)ccc4OC)c3o2)cc1 | 0.443838219 |
| COC(=O)[C@H]1[C@H]2C[C@@H]3c4[nH]c5ccccc5c4CCN3C[C@H]2C[C@@H](OC(=O)c2cc(OC)c(OC)c(OC)c2)[C@@H]1OC | 0.449386598 |
| O=c1cc(-c2ccc(O)cc2)oc2c([C@@H]3OC[C@@H](O)[C@H](O)[C@H]3O)c(O)c([C@@H]3O[C@H](CO)[C@@H](O)[C@H](O)[C@H]3O)c(O)c12 | 0.418865674 |
| O=c1cc(-c2ccc(O)cc2)oc2c([C@@H]3O[C@H](CO)[C@@H](O)[C@H](O)[C@H]3O)c(O)c([C@@H]3O[C@H](CO)[C@@H](O)[C@H](O)[C@H]3O)c(O)c12 | 0.425183878 |
| CC(C)=CCC/C(C)=C/CC/C(C)=C/CC/C(C)=C/CO | 0.49484868 |
| C=CCSC[C@H](N)C(=O)O | 0.687443883 |
| CCCC=C1OC(=O)C2=C1CCC1C3C=C4C(=O)OC(=CCCC)C4(CC3)C21 | 0.48969086 |
| CC(=O)OCCC(C)CCC=C(C)C | 0.684786979 |
| CCCCCC/C=C\CCCCCCCc1cccc(O)c1 | 0.466283268 |
| COc1cc(O)c(C(=O)/C=C/c2ccccc2)c(OC)c1 | 0.692158932 |
| CCN1C[C@]2(OC(=O)c3ccccc3NC(C)=O)CC[C@H](OC)[C@]34C1[C@](O)(C[C@H]23)[C@@]1(O)C[C@H](OC)[C@H]2C[C@@H]4[C@]1(O)[C@H]2OC | 0.565418193 |
| COc1cc2c(c(OC)c1OC)-c1c(cc3c(c1OC)OCO3)C[C@H](C)[C@H](C)C2 | 0.510007464 |
| COc1cc2c(=O)c(-c3ccc(O)cc3)coc2cc1O | 0.58102932 |
| CC1=C(C=O)C(C)(C)C[C@H](O[C@@H]2O[C@H](CO)[C@@H](O)[C@H](O)[C@H]2O)C1 | 0.662580129 |
| COc1ccc([C@@H]2COc3cc(O)ccc3C2)c(O)c1OC | 0.555494582 |
| COc1ccccc1C=CC=O | 0.567049368 |
| CCNC(=O)CC[C@H](N)C(=O)O | 0.649321324 |
| C=C1CC[C@@H]2[C@](C)(CO)[C@H](O)CC[C@@]2(C)[C@@H]1CCC1=CCOC1=O | 0.674322023 |
| CC(=O)OCC=C(C)CCC=C(C)C | 0.668390453 |
| CC(=O)[C@]1(O)CC[C@@]2(O)[C@]1(C)[C@H](OC(=O)/C=C(\C)C(C)C)C[C@@H]1[C@@]3(C)CC[C@H](O)CC3=CC[C@]12O | 0.618614253 |
| CCCC/C=C\CCCCCCCC(=O)O | 0.665400016 |
| CCCCCCCC/C=C\CCCCCCC(O)C(=O)O | 0.543661058 |
| COc1cc([C@@H]2c3cc4c(cc3[C@@H](O)[C@H]3COC(=O)[C@H]23)OCO4)cc(OC)c1OC | 0.550406049 |
| CC[C@H]1CN2CCc3c([nH]c4ccccc34)[C@H]2C[C@@H]1/C(=C\OC)C(=O)OC | 0.604427019 |
| C=C1CCC[C@]2(C)C[C@]3(O)OC(=O)C(C)=C3C[C@@H]12 | 0.638495789 |
| CC(=O)C=CC1=C(C)CCCC1(C)C | 0.628480308 |
| C=C(C)[C@H](CC=C(C)C)Cc1c(O)cc(O)c2c1O[C@H](c1ccc(O)cc1O)CC2=O | 0.578559368 |
| C=C[C@](C)(O)CC[C@@H]1[C@@]2(C)CCCC(C)(C)[C@@H]2CC[C@@]1(C)O | 0.667876876 |
| COc1cc2c(cc1OC)-c1cc3ccc(OC)c(OC)c3c(=O)n1CC2 | 0.555623101 |
| CC(C)=CC(O)C/C(C)=C/COc1c2ccoc2cc2oc(=O)ccc12 | 0.650436004 |
| COc1ccc([C@@H]2CC(=O)c3c(O)cc(O[C@@H]4O[C@H](CO[C@@H]5O[C@@H](C)[C@H](O)[C@@H](O)[C@H]5O)[C@@H](O)[C@H](O)[C@H]4O)cc3O2)cc1 | 0.438365575 |
| CC(C)C(C)C(O)C(O)C(C)C1CCC2C3COC(=O)C4CC(O)C(O)CC4(C)C3CCC12C | 0.835951235 |
| COc1c(C)c(O)cc2[o+]c(-c3ccccc3)ccc12.[O-][Cl+3]([O-])([O-])[O-] | 0.642415218 |
| COc1cc(O)c(C(=O)/C=C/c2ccc(O)cc2)c(OC)c1 | 0.712261667 |
| COc1cc2ccc(=O)oc2c(O)c1O | 0.609911182 |
| COc1cc(CO)ccc1O | 0.526184893 |
| CC(C)(O)/C=C/C(=O)[C@](C)(O)[C@H]1[C@H](O)C[C@@]2(C)[C@@H]3CC=C4[C@@H](C[C@H](O)C(=O)C4(C)C)[C@]3(C)C(=O)C[C@]12C | 0.706487652 |
| COc1ccc([C@H]2O[C@@H](c3ccc(OC)c(OC)c3)[C@H](C)[C@@H]2C)cc1OC | 0.501103329 |
| COc1c(O)cc2occ(-c3ccc(O)cc3)c(=O)c2c1O | 0.621338757 |
| CC(C)[C@@H](C)[C@@H](O)[C@H](O)[C@@H](C)[C@H]1CC[C@H]2[C@@H]3COC(=O)[C@H]4C[C@H](O)[C@H](O)C[C@]4(C)[C@H]3CC[C@]12C | 0.835951235 |
| CC1(C)C2=CCCC(C)(C)[C@]23CC[C@H]1C3 | 0.584707744 |
| COc1ccc(/C=C/c2cc(O)cc(O[C@@H]3O[C@H](CO)[C@@H](O)[C@H](O)[C@H]3O)c2)cc1O | 0.648468933 |
| CCCCCCCCCCCCCC[C@@H](O)[C@@H](O)[C@@H](N)CO | 0.508040035 |
| C=C[C@]1(C)CC[C@@H](C(=C)C)C[C@H]1C(=C)C | 0.629351342 |
| CC(C)(O)CCC(=O)[C@](C)(O)[C@H]1[C@H](O)C[C@@]2(C)[C@@H]3CC=C4[C@@H](C[C@H](O)[C@@H](O)C4(C)C)[C@]3(C)C(=O)C[C@]12C | 0.728186143 |
| COc1ccc(C2CC(=O)c3c(O)cc(OC4OC(CO)C(O)C(O)C4OC4OC(C)C(O)C(O)C4O)cc3O2)cc1 | 0.435966021 |
| COc1ccc(/C=C/C(=O)c2ccc(OC)c3c2OC(C)(C)C=C3)cc1 | 0.651316506 |
| CC1(C)CCC[C@]2(C)[C@@H]1CC[C@@]13C[C@@H](CC[C@H]12)[C@@](O)(CO)C3 | 0.694175818 |
| COc1cc2oc(=O)ccc2c(OC)c1C[C@@H](O)C(C)(C)O | 0.689218838 |

**Supplementary Table 5.** The docking scores of the compounds

| smiles | docking_score |
| --- | --- |
| CC1(C)CC[C@]2(C)CC[C@]3(C)C(=CC[C@@H]4[C@@]5(C)CC[C@H](O)C(C)(C)[C@@H]5CC[C@]43C)[C@@H]2C1 | -9.035 |
| C=C1CC[C@@]2(C)[C@H]([C@@H]1C)[C@H]1CC[C@@H]3[C@@]4(C)CC[C@H](O)C(C)(C)[C@@H]4CC[C@@]3(C)[C@]1(C)C[C@@H]2O | -8.886 |
| C[C@H]1[C@H](C)CC[C@]2(C)CC[C@]3(C)C(=CC[C@@H]4[C@@]5(C)CC[C@H](O)C(C)(C)[C@@H]5CC[C@]43C)[C@H]12 | -8.802 |
| CC1(C)C[C@@H](O)[C@]2(C)CC[C@]3(C)C(=CC[C@@H]4[C@@]5(C)CC[C@H](O)[C@](C)(CO)[C@@H]5CC[C@]43C)[C@@H]2C1 | -8.734 |
| C=C1CC[C@@]2(OC1)O[C@H]1C[C@H]3[C@@H]4CC=C5C[C@@H](O)C[C@@H](O)[C@]5(C)[C@H]4CC[C@]3(C)[C@H]1[C@@H]2C | -8.64 |
| C=C(C)[C@@H]1CC[C@]2(C)CC[C@]3(C)[C@H](CC[C@@H]4[C@@]5(C)CCC(=O)C(C)(C)[C@@H]5CC[C@]43C)[C@@H]12 | -8.626 |
| C[C@H]1[C@@H](O)CC[C@@H]2[C@]1(C)CC[C@H]1[C@@]2(C)CC[C@@]2(C)[C@@H]3CC(C)(C)CC[C@]3(C)CC[C@]12C | -8.626 |
| C=C(C)[C@@H]1CC[C@]2(C)CC[C@]3(C)[C@H](CC[C@@H]4[C@@]5(C)CC[C@H](O)C(C)(C)[C@@H]5CC[C@]43C)[C@@H]12 | -8.505 |
| CC1(C)[C@@H](O)CC[C@]2(C)[C@H]3CC=C4[C@H]5C[C@@]6(C)C(=O)O[C@H]6[C@H](O)[C@]5(C)CC[C@@]4(C)[C@]3(C)CC[C@@H]12 | -8.473 |
| C[C@@H]1CC[C@]2(C(=O)O)CC[C@]3(C)C(=CC[C@@H]4[C@@]5(C)CC[C@H](O)C(C)(C)[C@@H]5CC[C@]43C)[C@@H]2[C@]1(C)O | -8.389 |
| CC(=O)O[C@H]1CC[C@]2(C)[C@H]3C(=O)C=C4[C@@H]5[C@@H](C)[C@H](C)CC[C@]5(C)CC[C@@]4(C)[C@]3(C)CC[C@H]2C1(C)C | -8.374 |
| C[C@@H]1CC[C@@]2(OC1)O[C@H]1C[C@H]3[C@@H]4C[C@H](O)[C@H]5C[C@@H](O)CC[C@]5(C)[C@H]4CC[C@]3(C)[C@H]1[C@@H]2C | -8.372 |
| C[C@H]1[C@H](C)CC[C@]2(C)CC[C@]3(C)C(=CC(=O)[C@@H]4[C@@]5(C)CC[C@H](O)C(C)(C)[C@@H]5CC[C@]43C)[C@H]12 | -8.312 |
| CC1(C)CC[C@]2(C)CC=C3[C@]4(C)CC[C@H]5C(C)(C)[C@@H](O)CC[C@]5(C)[C@H]4CC[C@@]3(C)[C@@H]2C1 | -8.276 |
| C[C@@H]1C[C@@H]([C@@H](O)C(C)(C)O)O[C@H]2C[C@@]3(C)C(=C12)C[C@H](O)[C@H]1[C@@]2(C)CCC(=O)C(C)(C)[C@@H]2CC[C@@]13C | -8.272 |
| COC(=O)[C@]12CCC(C)(C)C[C@H]1C1=CC[C@@H]3[C@@]4(C)C[C@@H](O)[C@H](O)C(C)(C)[C@@H]4CC[C@@]3(C)[C@]1(C)CC2 | -8.259 |
| CC(=O)O[C@H]1CC[C@@]2(C)[C@H](CC[C@@H]3[C@@H]2CC[C@]2(C)[C@@H]4[C@H](C[C@@H]32)O[C@]2(CC[C@@H](C)CO2)[C@H]4C)C1 | -8.23 |
| C[C@@H]1CC[C@@]2(OC1)O[C@H]1C[C@H]3[C@@H]4CC=C5C[C@@H](O)C[C@@H](O)[C@]5(C)[C@H]4CC[C@]3(C)[C@H]1[C@@H]2C | -8.205 |
| CC1(C)CC[C@]2(C)CC[C@]3(C)C(=C2C1)CC[C@@H]1[C@@]2(C)CCC(=O)C(C)(C)[C@@H]2CC[C@]13C | -8.195 |
| CC(C)=CCC[C@]1(C)CC[C@]2(C)[C@H]3CC[C@@]4(C)[C@@H](CCC(=O)[C@@H]4C)[C@]3(C)CC[C@@]2(C)C1 | -8.189 |
| C=C1CC[C@]2(C)CC[C@]3(C)[C@H](CC[C@@H]4[C@@]5(C)CC[C@H](OC(C)=O)C(C)(C)[C@@H]5CC[C@]43C)[C@H]2[C@@H]1C | -8.183 |
| C[C@H]1CC[C@@]2(OC1)O[C@H]1C[C@H]3[C@@H]4CC=C5C[C@@H](O)C[C@@H](O)[C@]5(C)[C@H]4CC[C@]3(C)[C@H]1[C@@H]2C | -8.18 |
| C=C(C)[C@@H]1CC[C@]2(C=O)CC[C@]3(C)[C@H](CC[C@@H]4[C@@]5(C)CC[C@H](O)C(C)(C)[C@@H]5CC[C@]43C)[C@@H]12 | -8.167 |
| CC(C)[C@@H](C)/C=C/[C@@H](C)[C@H]1CC[C@H]2C3=C[C@@H]4O[C@@]45C[C@@H](O)CC[C@]5(C)[C@H]3CC[C@]12C | -8.129 |
| CC1=C2C[C@H]3[C@@H](CC(=O)[C@H]4C[C@@H](O)CC[C@@]43C)[C@@H]2CC[C@]12O[C@@H]1C[C@H](C)CN[C@H]1[C@H]2C | -8.125 |
| C[C@H]1[C@H](C)CC[C@]2(C(=O)O)CC[C@]3(C)C(=CC[C@@H]4[C@@]5(C)CC[C@H](O)C(C)(C)[C@@H]5CC[C@]43C)[C@H]12 | -8.117 |
| CC1(C)CC[C@]2(CO)CC[C@]3(C)C(=CC[C@@H]4[C@@]5(C)CC[C@H](O)C(C)(C)[C@@H]5CC[C@]43C)[C@@H]2C1 | -8.104 |
| C=C1CC[C@]2(C)CC[C@]3(C)[C@@H](CC[C@@H]4[C@@]5(C)CC[C@H](O)C(C)(C)[C@@H]5CC[C@]43C)[C@@H]2[C@@H]1C | -8.097 |
| CC(C)=CCC[C@@H](C)[C@@H]1CC[C@@]2(C)C3=C(CC[C@]12C)[C@]1(C)CC[C@H](O)C(C)(C)[C@H]1CC3 | -8.092 |
| C[C@H]1[C@H](C)CC[C@]2(C(=O)O)CC[C@]3(C)C(=CC[C@@H]4[C@@]5(C)CCC(=O)C(C)(C)[C@@H]5CC[C@]43C)[C@H]12 | -8.089 |
| C[C@H]1[C@H](C)CC[C@]2(C)CC[C@]3(C)C(=CC[C@@H]4[C@@]5(C)CC[C@@H](O)[C@](C)(C(=O)O)[C@H]5CC[C@]43C)[C@H]12 | -8.056 |
| C[C@H](C[C@H](O)[C@H]1OC1(C)C)C1=C2C[C@H](O)[C@H]3[C@@]4(C)CCC(=O)C(C)(C)[C@@H]4CC[C@]3(C)[C@@]2(C)CC1 | -8.044 |
| C=C(C)[C@H](O)[C@@H](O)C[C@@H](C)C1=C2C[C@H](O)[C@H]3[C@@]4(C)CCC(=O)C(C)(C)[C@@H]4CC[C@]3(C)[C@@]2(C)CC1 | -8.025 |
| CC(C)=CCC[C@@](C)(O)[C@H]1CC[C@@]2(C)[C@@H]1[C@H](O)C[C@@H]1[C@@]3(C)CC[C@H](O)C(C)(C)[C@@H]3CC[C@]12C | -8.018 |
| CC(C)=CCC[C@@H](C)[C@H]1CC[C@@]2(C)C3=C(CC[C@]12C)[C@@]1(C)CC[C@H](O)C(C)(C)[C@@H]1CC3 | -8.003 |
| CC[C@H](CC[C@@H](C)[C@H]1CC[C@H]2[C@@H]3CC=C4C[C@@H](O)CC[C@]4(C)[C@H]3CC[C@]12C)C(C)C | -7.986 |
| C[C@@H]1O[C@@H](O[C@H]2CC[C@@]3(C)[C@@H](CC[C@@]4(C)[C@@H]3C=CC3=C5CC(C)(C)CC[C@@]5(CO)[C@H](O)C[C@@]34C)[C@@]2(C)CO)[C@@H](O)[C@H](O[C@@H]2O[C@@H](CO)[C@H](O)[C@@H](O)[C@@H]2O)[C@H]1O | -7.982 |
| C=C(C)[C@@H]1CC[C@]2(C(=O)O)CC[C@]3(C)[C@H](CC[C@@H]4[C@@]5(C)CCC(=O)C(C)(C)[C@@H]5CC[C@]43C)[C@@H]12 | -7.957 |
| CC1(C)CC[C@@]2(CO)C(=C3C=C[C@@H]4[C@@]5(C)CC[C@H](O)[C@@](C)(CO)[C@@H]5CC[C@@]4(C)[C@]3(C)C[C@@H]2O)C1 | -7.954 |
| CC[C@H](CC[C@@H](C)[C@H]1CC[C@H]2[C@@H]3CC=C4C[C@@H](O[C@@H]5O[C@H](CO)[C@@H](O)[C@H](O)[C@H]5O)CC[C@]4(C)[C@H]3CC[C@]12C)C(C)C | -7.947 |
| C[C@H]1CC[C@@]2(OC1)O[C@H]1C[C@H]3[C@@H]4CC[C@@H]5C[C@H](O)CC[C@]5(C)[C@H]4CC[C@]3(C)[C@H]1[C@@H]2C | -7.947 |
| CC1(C)CC[C@]2(C(=O)O)CC[C@]3(C)C(=CC[C@@H]4[C@@]5(C)CC[C@H](O)C(C)(C)[C@H]5CC[C@]43C)[C@H]2C1 | -7.936 |
| C[C@H](C[C@H](O)[C@@H](O)C(C)(C)O)C1=C2C[C@H](O)[C@H]3[C@@]4(C)CCC(=O)C(C)(C)[C@@H]4CC[C@]3(C)[C@@]2(C)CC1 | -7.935 |
| C[C@@H]1CC[C@]2(NC1)O[C@@H]1C[C@@H]3[C@H]4CC[C@@H]5C[C@@H](O)CC[C@]5(C)[C@H]4CC[C@@]3(C)[C@H]1[C@H]2C.Cl | -7.927 |
| CC1(C)CC[C@]2(C(=O)O)CC[C@]3(C)C(=CC[C@@H]4[C@@]5(C)CC[C@H](O)[C@@](C)(C=O)[C@@H]5CC[C@]43C)[C@@H]2C1 | -7.926 |
| C[C@H]1[C@H](C)CC[C@]2(CO)CC[C@]3(C)C(=CC[C@@H]4[C@@]5(C)CC[C@H](O)C(C)(C)[C@@H]5CC[C@]43C)[C@H]12 | -7.909 |
| CC1(C)CC[C@@]2(C)CC[C@]3(C)C(=CC[C@H]4[C@@]5(C)CCC(=O)C(C)(C)[C@H]5CC[C@@]43C)[C@@H]2C1 | -7.902 |
| CC1(C)CC[C@]2(C(=O)O)CC[C@@]3(C)C(=CC[C@H]4[C@@]5(C)CC[C@H](O)C(C)(C)[C@@H]5[C@H](O)C[C@]43C)[C@@H]2C1 | -7.899 |
| COC(C)(C)[C@H](O)[C@@H](O)C[C@@H](C)C1=C2C[C@H](O)[C@H]3[C@@]4(C)CCC(=O)C(C)(C)[C@@H]4CC[C@]3(C)[C@@]2(C)CC1 | -7.845 |
| C[C@@H]1CC[C@]2(C(=O)O)CC[C@]3(C)C(=CC[C@@H]4[C@@]5(C)C[C@@H](O)[C@@H](O)C(C)(C)[C@@H]5CC[C@]43C)[C@@H]2[C@]1(C)O | -7.84 |
| C[C@@H]1CC[C@@]2(OC1)O[C@@H]1C[C@@H]3[C@@H]4CC=C5C[C@H](O)CC[C@@]5(C)[C@@H]4CC[C@]3(C)[C@@H]1[C@H]2C | -7.825 |
| C[C@@H]1CC[C@@]2(NC1)O[C@@H]1C[C@H]3[C@H]4CC[C@H]5C[C@H](O)CC[C@]5(C)[C@H]4CC[C@]3(C)[C@@H]1[C@H]2C | -7.816 |
| C=C(CC[C@@H](C(=O)O)[C@H]1CC[C@@]2(C)C3=C(CC[C@]12C)[C@@]1(C)CC[C@H](O)C(C)(C)[C@@H]1CC3)C(C)C | -7.809 |
| CC[C@H](CC[C@@H](C)[C@H]1CC[C@H]2[C@@H]3CC[C@H]4C[C@@H](O)CC[C@]4(C)[C@H]3CC[C@]12C)C(C)C | -7.809 |
| CC(C)=CCC[C@H](C)[C@@H]1CC[C@]2(C)C3=C(CC[C@@]12C)[C@@]1(C)CC[C@H](O)C(C)(C)[C@@H]1CC3 | -7.805 |
| C[C@H](CC[C@H](O)C(C)(C)O)[C@H]1CC[C@@]2(C)[C@@H]3CC=C4[C@@H](CC[C@H](O)C4(C)C)[C@@]3(C)C(=O)C[C@]12C | -7.802 |
| CC1=C(C)[C@H]2C3=CC[C@@H]4[C@@]5(C)CC[C@H](O)C(C)(C)[C@@H]5CC[C@@]4(C)[C@]3(C)CC[C@@]2(C(=O)O)CC1 | -7.801 |
| C=C(C)[C@@H]1CC[C@]2(C(=O)O)CC[C@]3(C)[C@H](CC[C@@H]4[C@@]5(C)CC[C@@H](O)C(C)(C)[C@@H]5CC[C@]43C)[C@@H]12 | -7.798 |
| C[C@H](CC[C@@H](O)C(C)(C)O)[C@H]1CC[C@@]2(C)[C@@H]3CC=C4[C@@H](CC[C@H](O)C4(C)C)[C@]3(C)[C@H](O)C[C@]12C | -7.797 |
| CC1(C)CCC[C@](C)([C@H]2CC[C@]3(C)[C@@H]2[C@H](O)C[C@@H]2[C@@]4(C)CC[C@H](O)C(C)(C)[C@@H]4CC[C@]23C)O1 | -7.777 |
| C/C=C(/CC[C@@H](C)[C@H]1CC[C@H]2[C@@H]3CC=C4C[C@@H](O)CC[C@]4(C)[C@H]3CC[C@]12C)C(C)C | -7.771 |
| C[C@H]1[C@H](C)CC[C@]2(C(=O)O)CC[C@]3(C)C(=CC[C@@H]4[C@@]5(C)CC[C@@H](O)C(C)(C)[C@@H]5CC[C@]43C)[C@H]12 | -7.758 |
| C=C(C)[C@@H]1CC[C@]2(C(=O)O)CC[C@]3(C)[C@H](CC[C@@H]4[C@@]5(C)CC[C@H](O)C(C)(C)[C@@H]5CC[C@]43C)[C@@H]12 | -7.713 |
| C=C(C)[C@@H]1CC[C@]2(CO)CC[C@]3(C)[C@H](CC[C@@H]4[C@@]5(C)CC[C@H](O)C(C)(C)[C@@H]5CC[C@]43C)[C@@H]12 | -7.712 |
| CC1(C)CC[C@]2(C(=O)O)[C@H](O)C[C@]3(C)C(=CC[C@@H]4[C@@]5(C)CC[C@H](O)[C@@](C)(CO)[C@@H]5CC[C@]43C)[C@@H]2C1 | -7.711 |
| CC(C)(O)[C@@H]1CC[C@](C)([C@H]2[C@@H](O)C[C@@]3(C)[C@@H]4C[C@H](O)[C@H]5C(C)(C)[C@@H](O)CC[C@]5(C)C4=CC[C@]23C)O1 | -7.702 |
| C=C(C)[C@@H]1CC[C@]2(C(=O)O)CC[C@]3(C)[C@H](CC[C@@H]4[C@@]5(C)CC[C@H](O)[C@@](C)(CO)[C@@H]5CC[C@]43C)[C@@H]12 | -7.698 |
| CC(C)CCC[C@@H](C)[C@H]1CC[C@@]2(C)C3=C(CC[C@]12C)[C@@]1(C)CC[C@H](O)C(C)(C)[C@@H]1CC3 | -7.678 |
| CC(=O)O[C@H]1CC[C@]2(C)[C@H]3CC=C4[C@@H]5CC(C)(C)CC[C@]5(C)CC[C@@]4(C)[C@]3(C)CC[C@H]2C1(C)C | -7.647 |
| C[C@H]1[C@@]23CC[C@H]4[C@@](C)(CC[C@@]5(C)[C@@H]6C[C@](C)(C(=O)O)CC[C@]6(C)CC[C@]45C)[C@@H]2C[C@@H](O)[C@]1(O)OC3 | -7.624 |
| CC(C)=CCC[C@@](C)(O)[C@@H]1CC[C@]2(C)[C@@H]1[C@@H](O)C[C@@H]1[C@]3(C)CC[C@@H](O[C@H]4O[C@H](CO)[C@H](O)[C@@H](O)[C@@H]4O[C@H]4O[C@H](CO)[C@H](O)[C@H](O)[C@H]4O)C(C)(C)[C@H]3CC[C@@]12C | -7.622 |
| CC[C@H](CC[C@@H](C)[C@H]1CC[C@H]2[C@@H]3CC=C4C[C@@H](OC(C)=O)CC[C@]4(C)[C@H]3CC[C@]12C)C(C)C | -7.617 |
| CC1(C)CC[C@]2(C(=O)O)CC[C@]3(C)C(=CC[C@@H]4[C@@]5(C)C[C@@H](O)[C@H](O)C(C)(C)[C@@H]5CC[C@]43C)[C@@H]2C1 | -7.616 |
| CC(C)(O)[C@@H]1CC[C@](C)([C@H]2[C@@H](O)C[C@@]3(C)[C@@H]4C[C@H](O)[C@H]5C(C)(C)[C@@H](O)CC[C@@]56C[C@@]46CC[C@]23C)O1 | -7.614 |
| CC1(C)CCC[C@@](C)([C@@H]2CC[C@@]3(C)[C@@H]2[C@H](O)C[C@@H]2[C@]4(C)CC[C@H](O)C(C)(C)[C@@H]4[C@H](O)C[C@@]23C)O1 | -7.531 |
| CC1(C)CC[C@]2(C(=O)O)CC[C@]3(C)C(=CC[C@@H]4[C@@]5(C)C[C@@H](O)[C@H](O)[C@@](C)(CO)[C@@H]5CC[C@]43C)[C@@H]2[C@@H]1O | -7.497 |
| CC1(C)CC[C@@]2(C(=O)O)[C@H](C1)C1=CC[C@H]3[C@@]4(C)CC[C@H](O)C(C)(C)[C@H]4CC[C@@]3(C)[C@]1(C)C[C@@H]2O | -7.483 |
| CC(C)=CCC[C@@H](C(=O)O)[C@H]1CC[C@@]2(C)C3=CC[C@H]4C(C)(C)[C@@H](O)CC[C@]4(C)C3=CC[C@]12C | -7.47 |
| C[C@H]1[C@H](C)CC[C@]2(C(=O)O)CC[C@]3(C)C(=CC[C@@H]4[C@@]5(C)C[C@@H](O)[C@H](O)C(C)(C)[C@@H]5CC[C@]43C)[C@H]12 | -7.458 |
| C[C@H]1CC[C@@]2(C(=O)O)CC[C@]3(C)C(=CC[C@H]4[C@@]5(C)C[C@H](O)[C@@H](O)C(C)(C)[C@H]5CC[C@]43C)[C@H]2[C@]1(C)O | -7.458 |
| C[C@H]1[C@H](C)CC[C@]2(C(=O)O)CC[C@]3(C)C(=CC[C@@H]4[C@@]5(C)C[C@@H](O)[C@H](O)[C@@](C)(CO)[C@@H]5CC[C@]43C)[C@H]12 | -7.402 |
| CC1(C)C[C@H]2C3=CC[C@@H]4[C@@]5(C)CC[C@H](O)[C@](C)(CO)[C@@H]5CC[C@@]4(C)[C@]3(C)C[C@@H](O)[C@@]2(CO)[C@@H](O)[C@@H]1O | -7.397 |
| CC1(C)CC[C@]2(C(=O)O)CC[C@]3(C)C(=CC[C@@H]4[C@@]5(C)C[C@@H](O)[C@H](O)[C@@](C)(CO)[C@@H]5CC[C@]43C)[C@@H]2C1 | -7.384 |
| CC1(C)C[C@H]2C3=CC[C@@H]4[C@@]5(C)CC[C@H](O)[C@@](C)(CO)[C@@H]5CC[C@@]4(C)[C@]3(C)C[C@H](O)[C@@]2(CO)[C@@H](O)[C@@H]1O | -7.349 |
| C=C(C)[C@@H]1CC[C@]2(C)[C@H](CC=C3[C@@H]4[C@@H](C)[C@H](C)CC[C@]4(C)CC[C@]32C)[C@@]1(C)CCC(=O)O | -7.343 |
| CCC(C)=CCC[C@@](C)(O)[C@@H]1CC[C@]2(C)[C@H]1[C@H](O)C[C@H]1[C@H]2C[C@@H](O[C@H]2O[C@@H](CO)[C@@H](O)[C@@H](O)[C@@H]2O[C@H]2O[C@H](C)[C@@H](O)[C@H](O)[C@@H]2O)[C@H]2C(C)(C)[C@H](O)CC[C@]12C | -7.337 |
| C[C@@H]1CC[C@]2(C(=O)O)CC[C@]3(C)C(=CC[C@@H]4[C@@]5(C)CC[C@H](O)[C@@](C)(CO)[C@@H]5CC[C@]43C)[C@@H]2[C@]1(C)O | -7.33 |
| CC1(C)CC[C@]2(C)CC[C@]3(C)C(=CC[C@H]4[C@@]5(C)CC[C@H](O)[C@](C)(C(=O)O)[C@H]5CC[C@]43C)[C@H]2C1 | -7.314 |
| CC1(C)CC[C@]2(C(=O)O)CC[C@]3(C)C(=CC[C@@]4(C)[C@@]3(C)CC[C@H]3C(C)(C)[C@H](O)CC[C@@]34C)[C@]2(C)C1 | -7.31 |
| C=C(CC[C@@H](C(=O)O)[C@H]1[C@H](O)C[C@@]2(C)C3=CC[C@H]4C(C)(C)[C@@H](O)CC[C@]4(C)C3=CC[C@]12C)C(C)C | -7.304 |
| C=C(C)[C@@H]1CC[C@]2(C)CC[C@]3(C)[C@H](CC[C@@H]4[C@@]5(C)CC[C@H](OC(=O)CCCCCCCCCCCCCCC)C(C)(C)[C@@H]5CC[C@]43C)[C@@H]12 | -7.272 |
| CC(C)CCC[C@@H](C)[C@H]1CC[C@H]2[C@@H]3CC[C@H]4C[C@@H](O)CC[C@]4(C)[C@H]3CC[C@]12C | -7.183 |
| CC[C@H](CC[C@@H](C)[C@H]1CC[C@H]2[C@@H]3CCC4=CC(=O)CC[C@]4(C)[C@H]3CC[C@]12C)C(C)C | -7.157 |
| C[C@@H]1[C@@H](C)CC[C@@]2(C(=O)O)CC[C@@]3(C)C(=CC[C@H]4[C@@]5(C)C[C@H](O)[C@H](O)[C@@](C)(CO)[C@@H]5[C@@H](O)C[C@@]43C)[C@@H]12 | -7.155 |
| CC1(C)CC[C@]2(C(=O)O)CC[C@@]3(C)C(=CC[C@@H]4[C@]5(C)CC[C@H](O)[C@](C)(CO)[C@@H]5CC[C@]43C)[C@@H]2C1 | -7.104 |
| CC(C)=CCC[C@@](C)(O)[C@H]1CC[C@]2(C)[C@@H]1[C@H](O)C[C@@H]1[C@@]3(C)CC[C@H](O)C(C)(C)[C@@H]3[C@@H](O)C[C@]12C | -7.103 |
| CC(C)=CCC[C@](C)(O)[C@H]1CC[C@]2(C)[C@@H]1[C@H](O)C[C@@H]1[C@@]3(C)CC[C@H](O)C(C)(C)[C@@H]3[C@@H](O)C[C@]12C | -7.091 |
| C=C(C[C@@H](O)[C@](C)(O)[C@H]1CC[C@@]2(O)C3=CC(=O)[C@@H]4C[C@@H](O)[C@@H](O)C[C@]4(C)[C@H]3CC[C@]12C)C(C)C | -6.997 |
| CC(C)(O)[C@H]1CC[C@@](C)([C@H]2[C@@H](O)C[C@@]3(C)[C@@H]4C[C@H](O)[C@H]5C(C)(C)[C@@H](O)CC[C@@]56C[C@@]46CC[C@]23C)O1 | -6.985 |
| CC(C)=CCC[C@](C)(O)[C@H]1CC[C@@]2(C)[C@@H]1[C@H](O)C[C@@H]1[C@]3(C)CC[C@H](O[C@H]4O[C@H](CO)[C@H](O)[C@H](O)[C@@H]4O)C(C)(C)[C@H]3CC[C@@]12C | -6.885 |
| CC(C)=CCC[C@](C)(O)[C@H]1CC[C@]2(C)[C@@H]1[C@H](O)C[C@@H]1[C@@]3(C)CC[C@H](O)C(C)(C)[C@@H]3CC[C@]12C | -6.8 |
| CC(C)[C@@H](C)C[C@@H](O)[C@](C)(O)[C@H]1CC[C@@]2(O)C3=CC(=O)[C@@H]4C[C@@H](O)[C@@H](O)C[C@]4(C)[C@H]3CC[C@]12C | -6.374 |
| CC(C)[C@@H](C)[C@@H](O)[C@H](O)[C@@H](C)[C@H]1CC[C@H]2[C@@H]3COC(=O)[C@H]4C[C@H](O)[C@H](O)C[C@]4(C)[C@H]3CC[C@]12C | -6.006 |
| CC(C)[C@@H](C)[C@H](O)[C@@H](O)[C@@H](C)[C@@H]1CC[C@@H]2[C@@H]3COC(=O)[C@@H]4C[C@H](O)[C@@H](O)C[C@@]4(C)[C@@H]3CC[C@@]21C | -5.838 |
